# Modeling the Sensitivity of Field Surveys for Detection of Environmental DNA (eDNA)

**DOI:** 10.1371/journal.pone.0141503

**Published:** 2015-10-28

**Authors:** Martin T. Schultz, Richard F. Lance

**Affiliations:** Environmental Laboratory, Engineer Research and Development Center, United States Army Corps of Engineers, Vicksburg, Mississippi, United States of America; West Chester University of Pennsylvania, UNITED STATES

## Abstract

The environmental DNA (eDNA) method is the practice of collecting environmental samples and analyzing them for the presence of a genetic marker specific to a target species. Little is known about the sensitivity of the eDNA method. Sensitivity is the probability that the target marker will be detected if it is present in the water body. Methods and tools are needed to assess the sensitivity of sampling protocols, design eDNA surveys, and interpret survey results. In this study, the sensitivity of the eDNA method is modeled as a function of ambient target marker concentration. The model accounts for five steps of sample collection and analysis, including: 1) collection of a filtered water sample from the source; 2) extraction of DNA from the filter and isolation in a purified elution; 3) removal of aliquots from the elution for use in the polymerase chain reaction (PCR) assay; 4) PCR; and 5) genetic sequencing. The model is applicable to any target species. For demonstration purposes, the model is parameterized for bighead carp (*Hypophthalmichthys nobilis*) and silver carp (*H*. *molitrix*) assuming sampling protocols used in the Chicago Area Waterway System (CAWS). Simulation results show that eDNA surveys have a high false negative rate at low concentrations of the genetic marker. This is attributed to processing of water samples and division of the extraction elution in preparation for the PCR assay. Increases in field survey sensitivity can be achieved by increasing sample volume, sample number, and PCR replicates. Increasing sample volume yields the greatest increase in sensitivity. It is recommended that investigators estimate and communicate the sensitivity of eDNA surveys to help facilitate interpretation of eDNA survey results. In the absence of such information, it is difficult to evaluate the results of surveys in which no water samples test positive for the target marker. It is also recommended that invasive species managers articulate concentration-based sensitivity objectives for eDNA surveys. In the absence of such information, it is difficult to design appropriate sampling protocols. The model provides insights into how sampling protocols can be designed or modified to achieve these sensitivity objectives.

## Introduction

Aquatic organisms shed DNA into the environment with bodily excretions such as feces, urine, sperm, and eggs. This DNA, called environmental DNA (eDNA), can be extracted from an environmental sample and analyzed using polymerase chain reaction (PCR) to determine whether or not a genetic marker unique to the target species is present in the sample. Positive results may also indicate presence of the target species at the sampled site. There is growing interest in using eDNA to monitor for invasive species and threatened or endangered species [1–4]. However, relatively little emphasis has been placed on understanding the potential errors associated with the eDNA method [5–8]. Methods and models designed to help investigators understand and communicate the false positive and false negative rates of eDNA surveys are needed to reduce the risk that faulty inference leads to errors in conservation management [8–12].

The eDNA method is a diagnostic test for the presence of the target marker in the monitored water body. The null hypothesis is that the target marker concentration in the water body is equal to zero and the alternate hypothesis is that the target marker concentration is greater than zero. Detection of the target marker in one or more environmental samples collected during a monitoring event is a positive result that falsifies the null hypothesis. Detection of the target marker in a sample may also indicate presence of the target species in the monitored water body; however, this conclusion requires additional information about the environmental system and the target species that are not addressed in this paper. Errors are classified as either false positive or false negative. False positive results can occur if DNA from a non-target species is mistaken for the genetic marker (taxon-specific DNA locus) of the target species or if environmental samples are contaminated. False negative results can occur if too few copies of a target marker are captured in a water sample, the target marker copies degrade in the sample following collection, or the laboratory assays are improperly executed [9]. Some errors can be controlled by using appropriate sampling protocols. In particular, the false negative results attributed to capturing too few target markers can be controlled by adjusting one or more parameters of the sampling protocol, including sample volume, number of samples, and number of PCR replicates. Most studies do not document the basis for sampling protocols used in eDNA surveys [13]. However, several authors report that sampling protocols are often based on ad-hoc criteria such as personal preference, availability of sampling equipment, and familiarity with past practice [13–15]. This can be attributed to the novelty of the eDNA method and the lack of information about what effect sample collection, processing, and analysis methods may have on eDNA detection rates. There is a need for models and other methods that will help investigators design sampling protocols that achieve target levels of sensitivity. Sensitivity is the probability that a target marker will be detected during a monitoring event if that marker is present in the monitored water body. Mathematically, sensitivity is the complement of the false negative rate. By understanding the sensitivity of eDNA surveys, investigators will be better able to interpret, communicate, and compare the results of eDNA surveys.

The model of sensitivity described in this paper is parameterized for bighead carp (*Hypophthalmichthys nobilis*) and silver carp (*H*. *molitrix*), two species of Asian carp that have become widespread in the Mississippi River basin since they were first introduced during the 1970s [16]. Populations of Asian carp in the Illinois River threaten to invade the Laurentian Great Lakes via the Chicago Area Waterway System (CAWS) [17]. Were these fish to become established in the Great Lakes, they could harm native fish populations. To help reduce the probability that bighead carp and silver carp might gain access to the Great Lakes via the CAWS, the United States Army Corps of Engineers (USACE) operates a set of electric fish barriers at Romeoville, Illinois, just upstream of the confluence of the Chicago Sanitary and Ship Canal with the Des Plaines River. Since 2009, federal and state agencies have monitored for Asian carp eDNA at locations between the electrical fish barriers and Lake Michigan.

The aim of this study is to model the sensitivity of eDNA monitoring surveys in aquatic habitats and simulate the effect of potential changes in a sampling protocol on that sensitivity. These changes include increasing the number of water samples, increasing the water sample volume, and increasing the number of PCR replicates. The experimental hypothesis is that some changes in sampling protocol will lead to larger increases in sensitivity than others. Sensitivity is estimated by developing a model that simulates sample collection, processing, and analysis. The model is implemented using Monte Carlo simulation assuming a sampling protocol consistent with that used for Asian carp in the CAWS. Results show that eDNA surveys can have a high false negative rate at low target marker concentrations and that this false negative rate can be attributed to processing and division of the water samples prior to PCR. The model provides a means to estimate the false negative rate of an eDNA monitoring survey, provides insights into why false negative rates may be high, and provides a means to quantify the effect of proposed changes in sampling protocol on the false negative rate.

This study emphasizes the effect of sampling protocol on the sensitivity of eDNA field surveys. However, factors other than sampling protocol may also influence the sensitivity of eDNA field surveys. In particular, a sampling protocol may exhibit more or less sensitivity at a particular site depending on environmental conditions that influence target marker concentration at the time of sampling. For example, high stream flows may dilute target marker concentrations, making a sampling protocol less sensitive during high flow periods. The sensitivity of an eDNA field survey may also be influenced by water temperature and pH because these can affect target marker degradation rates. All else equal, sensitivity will tend to be lower when environmental conditions are more conducive to degradation because target marker concentrations will tend to be lower. Although this study does not address factors that influence target marker concentration, these factors could be accounted for by modeling target marker concentrations as a function of environmental factors that influence target marker concentration at the sampling site.

While this particular application of the modeling approach is developed for field surveys that use PCR to detect bighead and silver carp eDNA in the CAWS, the modeling approach and conclusions described in this paper are applicable to a broad class of eDNA field surveys. This includes those that use quantitative PCR and droplet digital PCR, which are becoming more popular than conventional PCR [14, 18–19]. The modeling approach developed in this paper can be adapted for these other sampling and analysis protocols and, although the specific numerical results would differ in these other applications of the approach, the insight that processing and division of water samples leads to high false negative rates at low target marker concentrations will be an important one regardless of what procedures are used to analyze water samples.

The modeling approach makes it possible for researchers and managers to design and compare sampling protocols based on the relative ability of those protocols to detect the target marker at a given environmental concentration. Simulating the probability that a field survey could detect the target marker if that target marker were present at the given concentration also helps environmental managers to interpret negative survey results. For example, a negative survey result based on a sampling and analysis protocol capable of detecting the target marker at ten copies/L with a probability of 0.95 can be given more weight than a negative result based on a field sampling and analysis protocol that is only capable of detecting a target marker at 100 copies/L with an equivalent probability. The model makes it possible to compare the sensitivity of two or more eDNA sampling protocols in the absence of information about the actual field concentration. This situation confronts most, if not all, eDNA field surveys.

## Materials and Methods

### Collection and Analysis of Environmental Samples

The methods used to collect and analyze water samples for the presence of bighead and silver carp eDNA in the CAWS from 2011 through 2014 are described in a Quality Assurance Project Plan (QAPP) (S1 File). All water samples were collected from public use portions of the CAWS; therefore, no specific permissions were required for collection. No endangered or protected species were involved. Sample collection methods are similar to those described by Jerde *et al*. [3], who report the results of eDNA monitoring in the CAWS from June 2009 through August 2010. Water samples are collected by dipping the mouth of a 2 L bottle just below the water surface. Samples are filtered through one or more 1.5 μm glass fiber filters, which are then packed on dry ice for shipment to a laboratory. DNA is extracted and isolated from the filter. The resulting DNA elution, which is comprised of purified DNA in 100 μl of sterile deionized water, is then stored at -20°C for subsequent analysis.

Analysis of samples proceeds by extracting eight 1μl aliquots from the elution to supply template DNA for eight PCR replicates. The genetic markers for bighead and silver carp used in this study are those described by Jerde *et al*. [3]. These PCR assays are strictly a test for the presence or absence of the genetic marker. As outlined in the QAPP, if none of the replicates tests positive for the target marker, the water sample is classified as negative. If one or more replicates from a water sample tests positive for the target marker, then one of the positive replicates is selected for DNA sequencing to confirm that the nucleotide sequence of the amplicon closely matches known DNA sequences from the target species. If so, the water sample is classified as positive. If not, then the sample is classified as negative.

### Probability of Detecting eDNA in Environmental Samples

The sensitivity of the eDNA method is modeled as a function of ambient target marker concentration in the monitored water body. The model of sensitivity accounts for five steps of sample collection and analysis: 1) collection of a filtered water sample from the source; 2) extraction of DNA from the filter and isolation in a purified elution; 3) removal of aliquots from the elution for use in the PCR assay; 4) PCR; and 5) genetic sequencing to confirm positive PCR assays.

### Collection of a filtered water sample from the source

The mean number of target markers captured in a water sample can be calculated from the target marker concentration and the sample volume: $\mu_{N_{S}}=C_{M}\cdot V_{S}$. The variable $\mu_{N_{S}}$ is the mean number of target marker copies in a monitoring sample, *C*_*M*_ is the target marker concentration in the water body (copies/L), and *V*_*S*_ is the volume of the monitoring sample (L). The number of copies in repeated samples of the same size taken from a common source can be modeled using a Poisson distribution if the copies are randomly distributed in the source, the source is homogenous, and the samples are independent [20]. Using a Poisson distribution, Eq 1 is the probability mass function for the number of copies of the target marker in a water sample:

$$p\left[N_{S}|\mu_{N_{S}}\right]=\frac{{\left(\mu_{N_{S}}\right)}^{N_{S}}\cdot exp\left(-\mu_{N_{S}}\right)}{N_{S}!}$$ (1)

The term $p\left[N_{S}|\mu_{N_{S}}\right]$ is the probability of observing *N*_*S*_ copies of the target marker in a random sample from a well-mixed water body. The variable $\mu_{N_{S}}$ is the mean number of target markers in the monitored water body and the parameter of the distribution. The term $exp\left(-\mu_{N_{S}}\right)$ is the probability that the sample contains no copies of the target marker.

Water samples are stored in a cooler with ice, maintained at approximately 4°C, and processed within sixteen hours of collection. Samples are processed by filtering the water through a 1.5 μm glass fiber filter to trap eDNA. The filters are then packed on dry ice and shipped to a laboratory for processing and analysis.

### Extraction and purification of eDNA from a filtered sample

The DNA is extracted from the filter at the laboratory using a PowerWater^®^ DNA Isolation Kit (MO BIO Laboratories, Inc., Carlsbad, CA), which is specifically designed to extract DNA from water samples. The DNA extract is eluted with 100 μl sterile deionized water. The number of DNA copies in this elution is calculated: *N*_*E*_ = ϕ∙*N*_*S*_. The variable *N*_*E*_ is the number of target markers in the final elution and ϕ is the efficiency of the filtration and extraction processes. This term, ϕ, also captures any losses of eDNA from the sample that may have occurred because of degradation during storage and shipping.

Capture and extraction methods can have a significant impact on recovery of DNA from environmental samples [13, 21], and the efficiency of methods used to extract DNA from CAWS water samples is believed to be low. Uncertainty in extraction efficiency is represented here as a triangular distribution with a lower bound of 0, a median of 0.15 and an upper bound of 0.3. This represents a general consensus of individuals responsible for carrying out the analysis of CAWS water samples at the USACE Engineer Research and Development Center (ERDC). This estimate is consistent with the results of studies that have measured extraction efficiency. For example, Eichmiller *et al*. [22] report an overall mean extraction efficiency of 11 percent using the QIAamp DNA Stool Mini Kit (Qiagen, Hilden, Germany). Mumy and Findlay [23] evaluated four commercial DNA extraction kits and report mean extraction efficiencies ranging from 1.4 percent to 28.3 percent.

The concentration of the target marker in the elution is expressed in copies/μl, and is calculated assuming a 100 μl elution volume: $C_{E}=N_{E}\cdot V_{E}^{-1}$, where *C*_*E*_ is the concentration of target marker in the elution (copies/μl), *N*_*E*_ is the number of target markers in the elution, and *V*_*E*_ is the initial elution volume (μl).

### Sampling of the DNA elution

One or more 1 μl aliquots are extracted from each elution to serve as template DNA for PCR assays. As these aliquots are pipetted out of the elution, the number of target marker copies captured in each aliquot, *N*_*R*_, will vary as a result of random sampling error. Provided the elution is well-mixed and the volume of the replicate is small relative to the volume of the elution, this variability can be described using a Poisson distribution, which has one parameter equal to the mean or expected concentration of the elution, $\mu_{N_{R}}$, as in Eq 2:

$$p\left[N_{R}|\mu_{N_{R}}\right]=\frac{{\left(\mu_{N_{R}}\right)}^{N_{R}}\cdot exp\left(-\mu_{N_{R}}\right)}{N_{R}!}$$ (2)

The variable $\mu_{N_{R}}$ is the mean concentration of the target marker in the elution and is equal to *C*_*E*_.

### Probability of fluorescence on an agarose gel

The fewer the copies of a target marker input into a PCR, the less the chance that the PCR primers will encounter and bind with those target markers during the reaction and the less the chance that an adequate number of amplicons will be produced such that a visible band of fluorescence can be observed on an agarose gel. The target marker almost always represents a miniscule fraction of the total pool of eDNA in a sample. If target marker concentrations are low, this is one means by which false negative results can occur. False negative results can also be caused by the presence of inhibitors in the sample. Inhibition was not measured during the CAWS field surveys and is not considered to be a factor influencing sensitivity in this application of the simulation model. However, where PCR inhibition is prevalent, the model will tend to overestimate sensitivity. Inhibition can be accounted for by reducing the expected number of target marker copies input into PCR by some amount that reflects measured levels of PCR inhibition.

A set of experiments was conducted using serial dilutions of bighead and silver carp target markers to assess the relationship between target marker copy number and the production of observable fluorescence on an agarose gel. Experimental results are summarized in S1 Table. In general, the greater the expected number of copies in replicate aliquots drawn from serial dilutions, the larger the fraction of replicates that produced visible fluorescence. A gamma density function fit to experimental results using the method of moments characterizes uncertainty in the minimum number of copies required to produce visible fluorescence. The parameters of the distribution are $\alpha_{F}={\bar{x}}^{2}/s^{2}$ and $\beta_{F}=s^{2}/\bar{x}$, where $\bar{x}$ and *s* are the mean and standard deviation of expected target marker counts in 1 *μ*l aliquots that produced visible fluorescence. Parameter values are summarized in Table 1. The gamma density function was chosen because it is appropriate for non-negative quantities that are asymmetrically distributed and it exhibited a superior ability to fit the experimental results when compared with alternative distributions.

**Table 1:** Parameters of the Gamma Density Functions Fit to Results of the Fluorescence Experiment.

Parameter	Bighead carp	Silver carp
*α*_*F*_	1.885	2.092
*β*_*F*_	1.486	2.238

Integration of the fitted gamma density function yields a continuous probability distribution that describes the probability of visible fluorescence as a function of the copy number in an aliquot of sample used in the PCR assay. No closed form solution exists for the gamma density function. Therefore, the probability of fluorescence given the expected number of target marker copies contained in the 1 μl aliquots of elution input into PCR, *p*[*F*|*N*_*R*_], is obtained by numerical integration of the gamma density function as shown in Eq 3:

$$p\left[F|N_{R}\right]=\int_{0}^{N_{R}}\frac{N_{R}^{\left(\alpha_{F}-1\right)}\cdot exp\left(\frac{\alpha_{F}}{\beta_{F}}\right)}{\beta_{F}^{\alpha_{F}}\cdot \Gamma \left(\alpha_{F}\right)}dN_{R}$$ (3)

**Fig 1:**
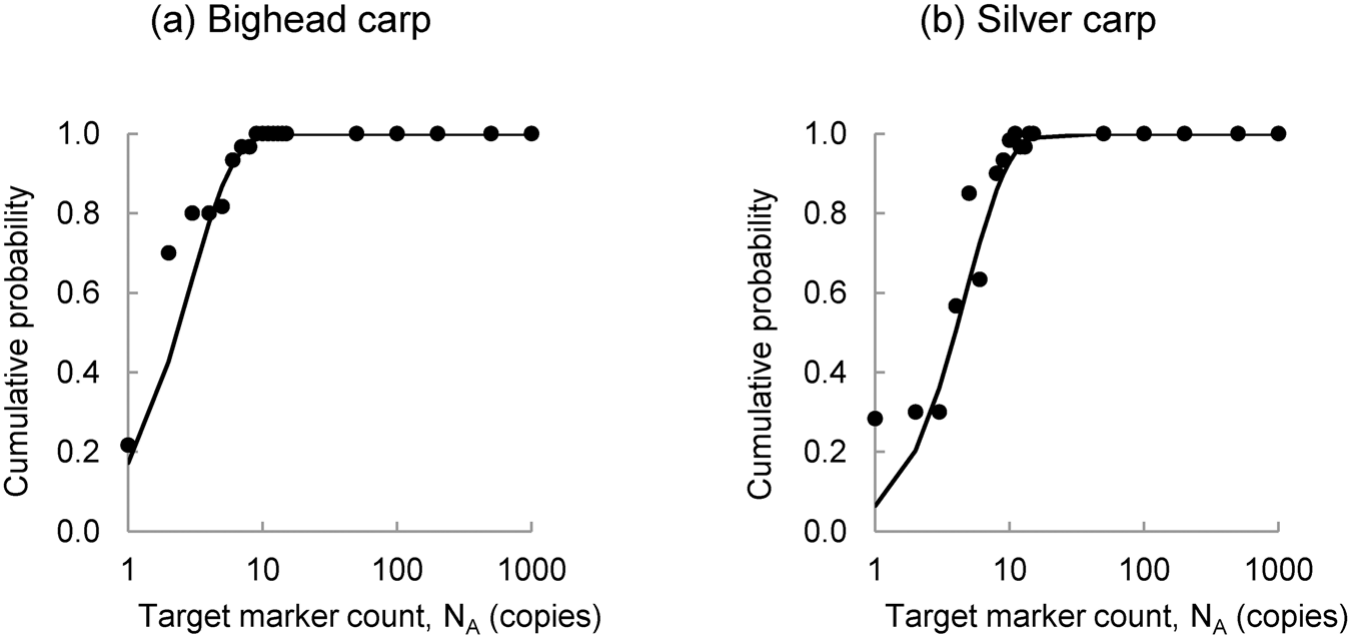
Gamma Distribution Functions Characterizing Probability of Fluorescence. These gamma distribution functions characterize the probability of the event that fluorescence is observed on an agarose gel given the number of target marker copies in a 1 μl aliquot of extraction elution used in PCR for (a) bighead carp and (b) silver carp.

*F* is the event that a replicate PCR produces visible fluorescence. The gamma distribution functions are illustrated in Fig 1. These results show that the PCR assay can detect very small quantities of eDNA. For example, when two copies of the bighead carp target marker are present in a 1 μl PCR replicate, the probability of observing fluorescence is 0.43. When three copies are present, the probability of observing fluorescence is 0.63. The probability of observing fluorescence is slightly lower for the silver carp target marker. When four copies of the silver carp target marker are present in the a 1 μl PCR replicate, the probability of observing fluorescence is 0.5. These results are consistent with the results reported by Jerde *et al*. [3], DeJean *et al*. [24], and Wilcox *et al*. [19].

### Probability of successfully sequencing PCR products

If the presence of the target marker is indicated by visible fluorescence in one or more of the PCR replicates, then the replicate emitting the strongest signal is selected for nucleotide sequencing. The cycle-sequencing reaction is based on the Sanger method [25]. As with PCR, the sequencing reaction is more likely to be successful if the number of amplicons input to the reaction is high. The number of amplicons available for sequencing is in most cases an increasing function of the copy number input into PCR.

**Fig 2:**
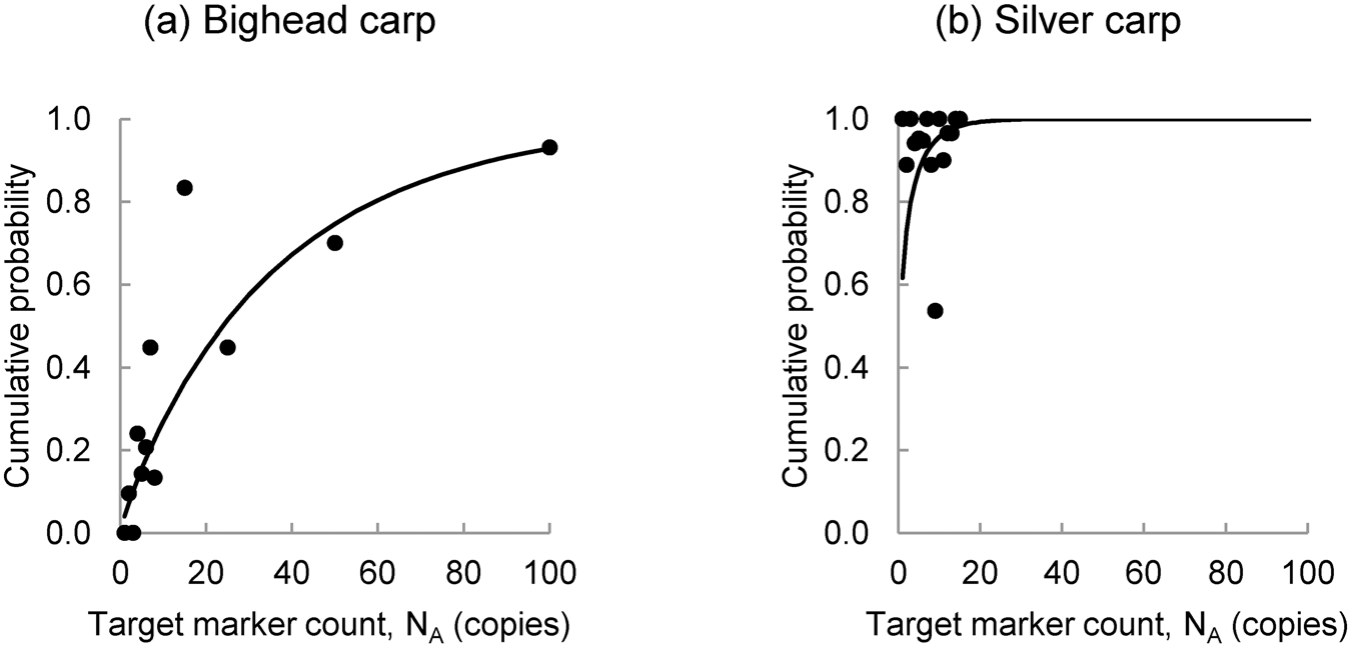
Gamma Distribution Functions Characterizing Probability of Successful Sequencing. These gamma probability distribution functions characterize the probability of the event that PCR amplicons are successfully sequenced given the initial number of target marker copies in a 1 μl aliquot of extraction elution used in PCR for (a) bighead carp and (b) silver carp.

A set of experiments was conducted using serial DNA dilutions of bighead and silver carp DNA to assess the probability that genetic sequencing would confirm the identity of the target markers. Confirmation required at least a 95 percent sequence match between an amplicon sequence and known sequences for the target species. Experimental results are summarized in S2 Table. The greater the initial expected number of copies in the replicate aliquots drawn from serial dilutions, the larger the fraction of replicates that were successfully sequenced. As with the analysis of fluorescence, the probability is characterized using a gamma density function that is fit to experimental results using the method of moments. The moments are calculated from the expected number of target marker copies input into PCR assays that culminated in a target species match. The probability of successfully sequencing a PCR replicate, *p*[*S*|*N*_*R*_], is obtained by numerical integration of the gamma density function. The parameters of the gamma density function, α_*S*_ and β_*S*_, are summarized in Table 2 and the fitted gamma distribution functions are illustrated in Fig 2. As with the analysis of fluorescence, the gamma distributions were selected from a set of alternative distributions appropriate for non-negative, skewed quantities based on their superior ability to fit the experimental results.

**Table 2:** Parameters of Gamma Density Functions Fit to Results of the Sequencing Experiment.

Parameter	Bighead carp	Silver carp
*α*_*S*_	0.877	0.289
*β*_*S*_	41.631	6.968

The bighead carp target marker is more difficult to sequence than the silver carp target marker. This is reflected by the slope of the fitted distribution for bighead carp when compared to the fitted distribution for silver carp (Fig 2) and by the relatively high value of β_S_ for bighead carp (Table 2). Both probability distributions in Fig 2 are shown over target marker counts from 0 to 100, which is the point at which most PCR replicates containing bighead carp target markers could be successfully sequenced. However, experiments on the silver carp target marker were only carried out on a range of expected target marker counts from 1 to 15, as indicated by the points in Fig 2b. As a result, Fig 2b may give the impression that the gamma distribution is not appropriate for silver carp. However, when the fits are evaluated using the root mean squared error (RMSE), the gamma distribution fits just as well for silver carp as it does for bighead carp. The RMSE is 0.164 for bighead carp and 0.167 for silver carp.

### Probability of target marker detection

The probabilities of fluorescence and successful sequencing can be multiplied to estimate the overall probability that a PCR replicate tests positive for the target marker because they are independent given the number of target markers in the replicate. The probability of a positive replicate given the target marker concentration in the monitored water body is calculated as in Eq 4:

$$p\left[R|C_{M}\right]=\underset{N_{R}}{\sum^{\;}}p\left[N_{R}|\mu_{N_{R}}\right]\cdot p\left[F|N_{R}\right]\cdot p\left[S|N_{R}\right]$$ (4)

The term $p\left[N_{R}|\mu_{N_{R}}\right]$ is the probability that some number of target marker copies is present as template in a PCR replicate and is a function of *C*_*M*_. A sample test consists of multiple replicate PCRs. The greater the number of replicates run, the greater the probability of observing at least one positive PCR. Let *A* be the event that at least one PCR in a set of *k* replicate PCRs tests positive for the target marker. Then, the probability that the sample tests positive can be calculated as follows: *p*[*A*|*C*_*M*_] = 1–(1–*p*[*R*|*C*_*M*_])^*k*^. This function expresses the probability of detecting target species eDNA in a single water sample. Multiple water samples are collected during a monitoring event, and a similar function can be used to describe the probability of detecting eDNA in the water body: *p*[*E*|*C*_*M*_] = 1–(1–*p*[*A*|*C*_*M*_])^*N*^. The term *p*[*E*|*C*_*M*_] is the conditional sensitivity of the eDNA monitoring event and *N* is the number of water samples taken from the monitored water body. The false negative rate for the monitoring event is the complement of *p*[*E*|*C*_*M*_].

The model simulates sensitivity of the eDNA monitoring event as a function of target marker concentration in the water body. The model is implemented by defining the parameters of a sampling protocol, including the number and volume of water samples, the volume to which the DNA extract is diluted (elution volume), and the number of PCR replicates. The simulation is accomplished by sampling from probability distributions characterizing uncertainty in selected variables assuming an ambient concentration of the target marker in the monitored water body, *C*_*M*_. Five sources of uncertainty are considered in this simulation, including: 1) *N*_*S*_, the number of target marker copies captured in a raw water sample; 2) ϕ, filtration and extraction efficiency; 3) *N*_*A*_, the number of target marker copies captured in an aliquot of the DNA extraction elution; 4) *F*, fluorescence, and 5) *S*, successful sequencing of positive PCRs. Results are generated for each potential target marker concentration using Monte Carlo simulation, with 50,000 samples drawn at each potential target marker concentration. The baseline field sampling protocol is characterized by ten 2 L water samples and eight PCR replicates. This characterization is consistent with the CAWS sampling protocol, but any other sampling protocol could also be considered.

## Results

**Fig 3:**
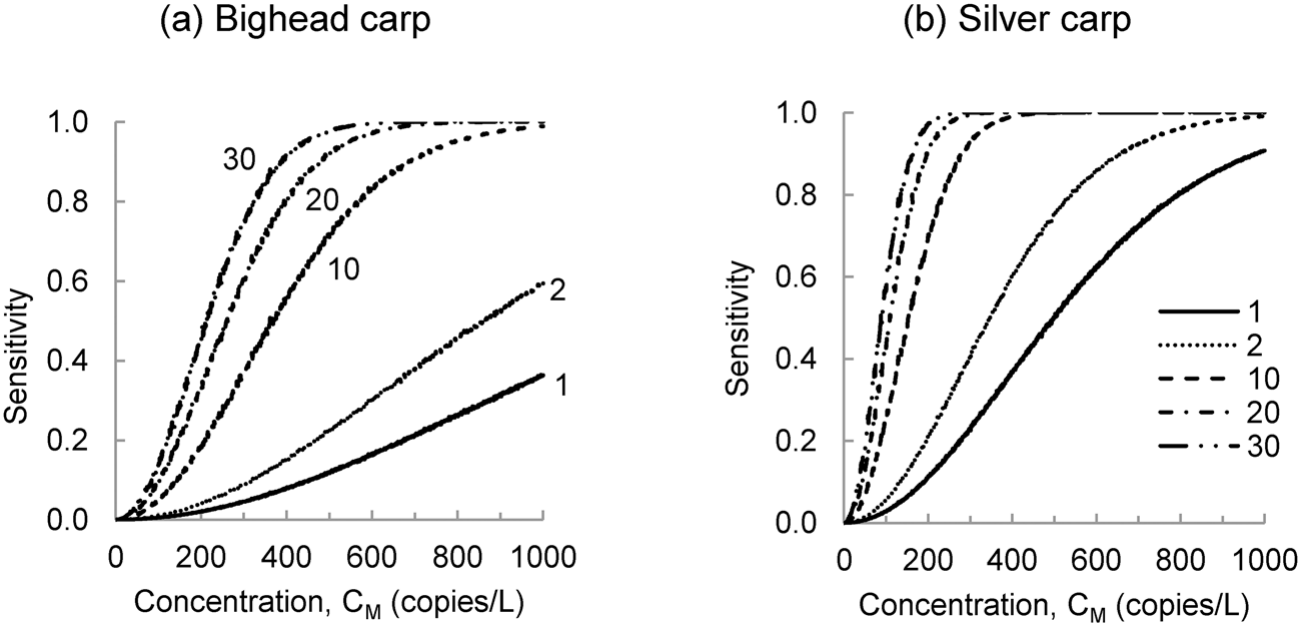
Probability of detecting bighead carp and silver carp eDNA during a monitoring event. The probability of detecting the (a) bighead carp and (b) silver carp target marker is shown for monitoring events with 1, 2, 10, 20 and 30 water samples. At low concentrations in the environment, a large number of samples may be needed to detect the target marker with a high level of confidence.

**Fig 4:**
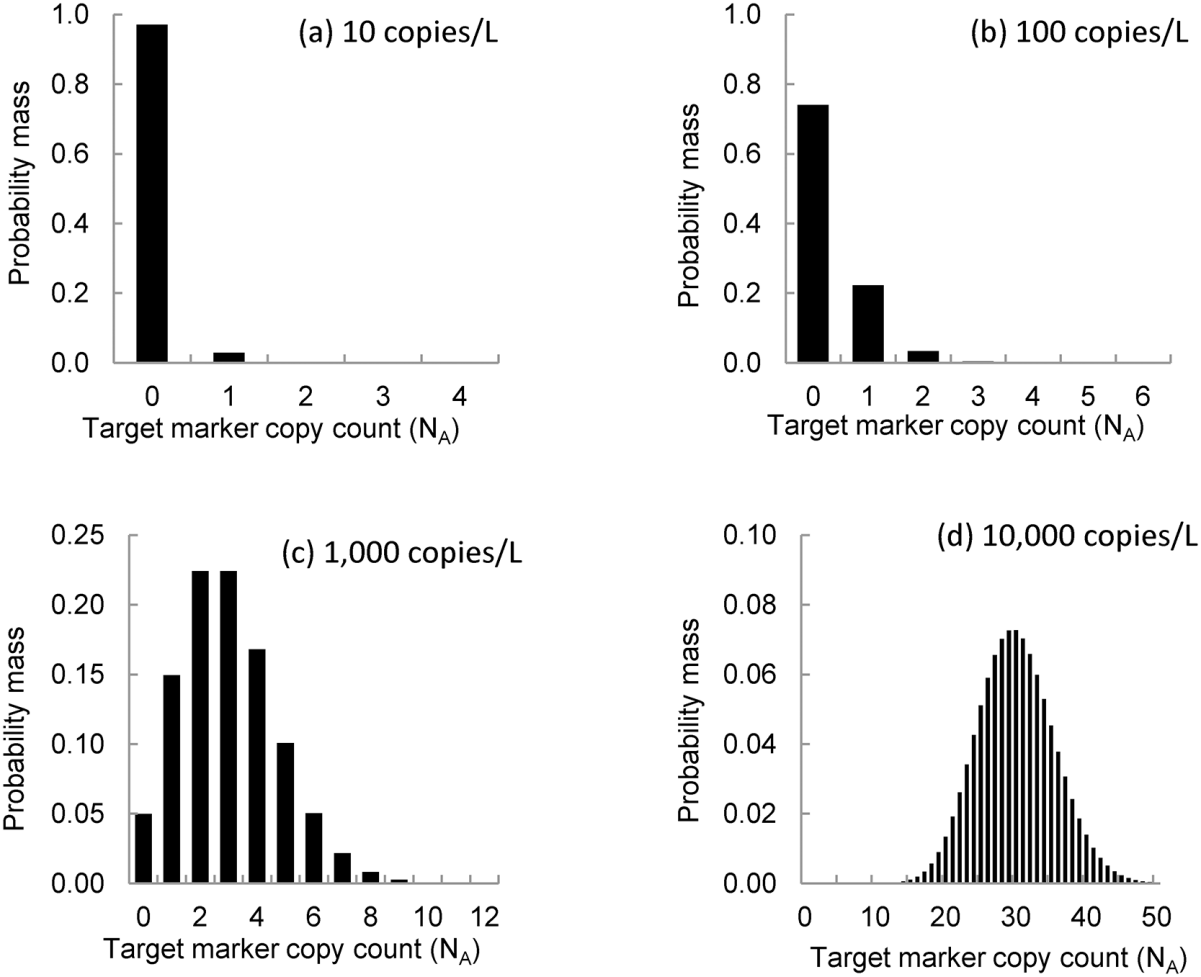
Simulated frequency of target marker counts in 1 μl aliquots. The figure shows uncertainty in the number of target markers in aliquots drawn from a 100 μl extraction elution created by processing a 2 L water sample collected from a water body with an environmental concentration equal to (a) 10 copies/L, (b) 100 copies/L, (c) 1,000 copies/L, and (d) 10,000 copies/L.

Sensitivity is the probability that the target marker will be detected during a monitoring event if it is present in the monitored water body. The probability of detection increases with the concentration and the number of water samples, as shown in Fig 3. At lower target marker concentrations, eDNA monitoring events can have a high false negative rate because the target marker counts in PCR replicates are low. For example, at an ambient target marker concentration of 10 copies/L, 97 percent of PCR replicates will contain no copies of the target marker (Fig 4a). At an ambient concentration of 100 copies/L, 74 percent of PCR replicates will contain no copies of the target marker and 22 percent of PCR replicates will contain only one copy of the target marker (Fig 4b). Even at ambient concentrations of 1,000 copies/L, 65 percent of PCR replicates will contain no more than three copies of the target marker (Fig 4c). These low target marker counts can be attributed to processing of the raw water sample and division of the extraction elution.

**Fig 5:**
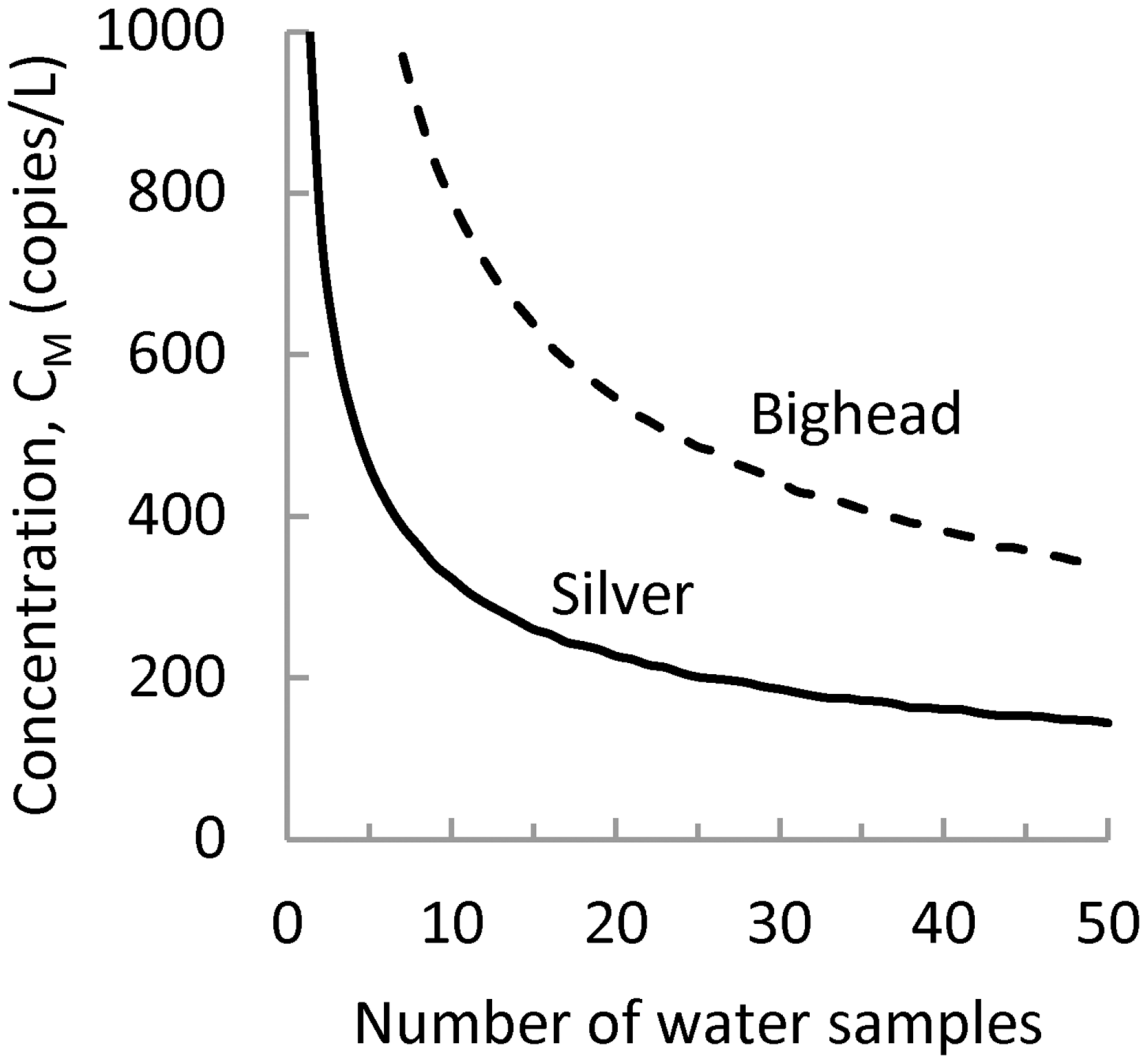
Concentration of the target marker that can be detected with probability 0.95. The figure shows that the minimum concentration that is detectable with probability 0.95 decreases as the number of samples used in the baseline sampling protocol increases.

In general, ambient target marker concentrations will not be known at the time that eDNA monitoring surveys are designed. Therefore, it may be useful to design sampling protocols to achieve a desired sensitivity given a minimum target marker concentration. For example, Fig 5 plots the minimum bighead and silver carp target marker concentrations that can be detected using the baseline sampling protocol assuming a sensitivity goal of 0.95. If ten samples are taken, this sampling protocol can detect, with probability 0.95, a minimum target marker concentration of 789 copies/L for the bighead carp and 322 copies/L for the silver carp. These minimum concentrations could be further reduced by increasing the number of water samples. For example, if the number of water samples is increased to 50, the sampling protocol can detect, with probability 0.95, bighead carp target marker concentrations at 342 copies/L and silver carp target marker concentrations at 143 copies/L (Fig 5). There are diminishing returns to increasing the number of samples. As the number of samples increases, the minimum target marker concentration decreases at a slower rate.

**Fig 6:**
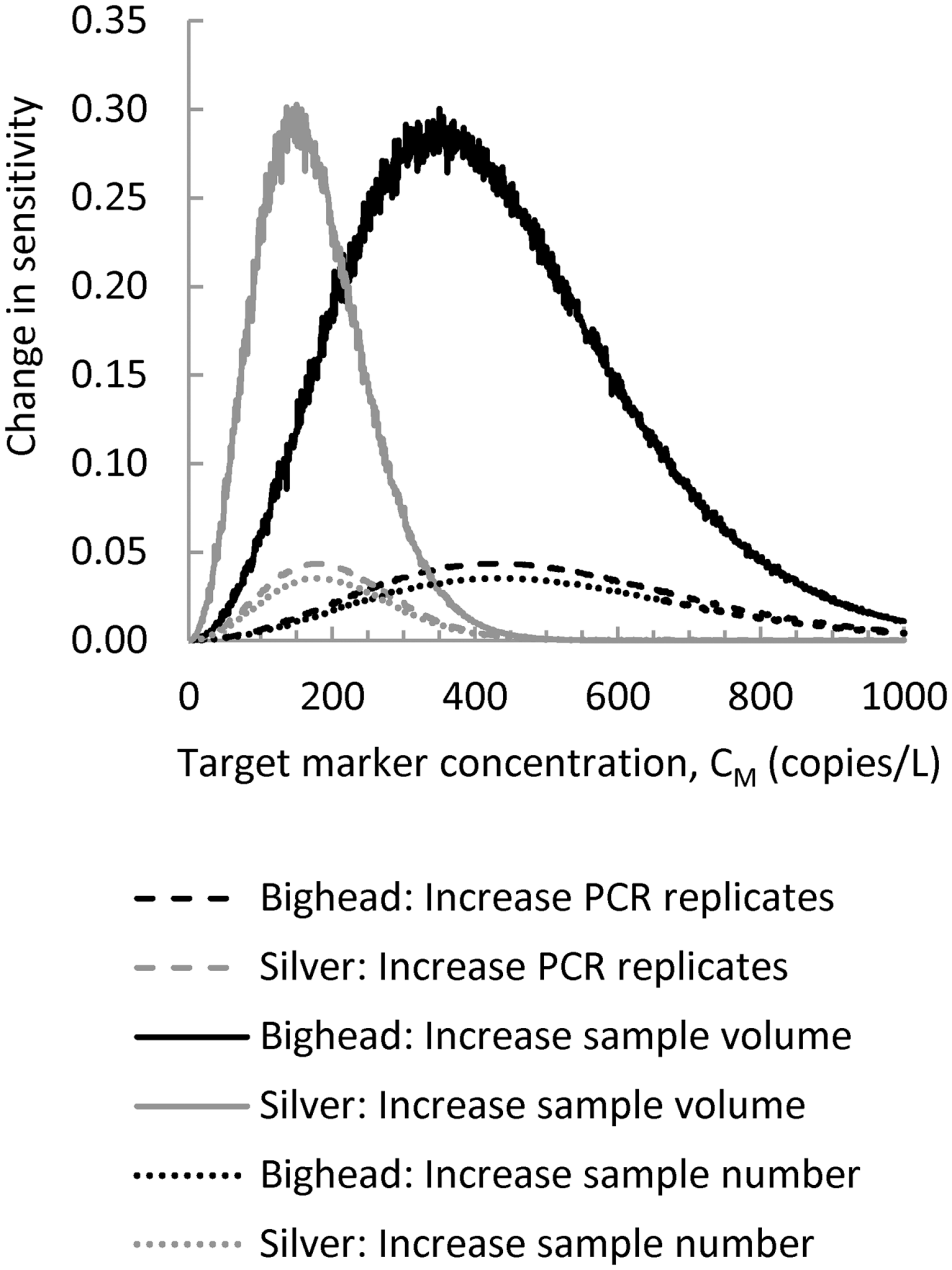
Change in sensitivity caused by a one unit increase in selected parameters of the baseline sampling protocol. The change in sensitivity caused by a change in sampling protocol will depend in part on the environmental concentration of the target marker in the monitored water body and on the target marker.

There are three basic strategies to increase sensitivity: 1) increase the number of water samples; 2) increase the volume of water samples; or 3) increase the number of PCR replicates. The potential benefits of a proposed change in the sampling protocol can be evaluated using the difference in sensitivity between the proposed and baseline sampling protocol. The benefit of a unit increase in each parameter of the baseline sampling protocol is summarized in Fig 6. Results show that effects on sensitivity occur over a limited range of target marker concentration and that this varies by marker. The largest potential improvement in sensitivity is obtained by increasing water sample volume from 2 L to 3 L. Improvements in sensitivity greater than 0.05 only occur between bighead target marker concentrations of 90–780 copies/L and silver carp target marker concentrations of 40–320 copies/L. Benefits of this strategy, measured in terms of an increase in sensitivity, reach a maximum of about 0.3 when target marker concentrations are approximately 350 and 150 copies/L for bighead carp and silver carp, respectively.

**Fig 7:**
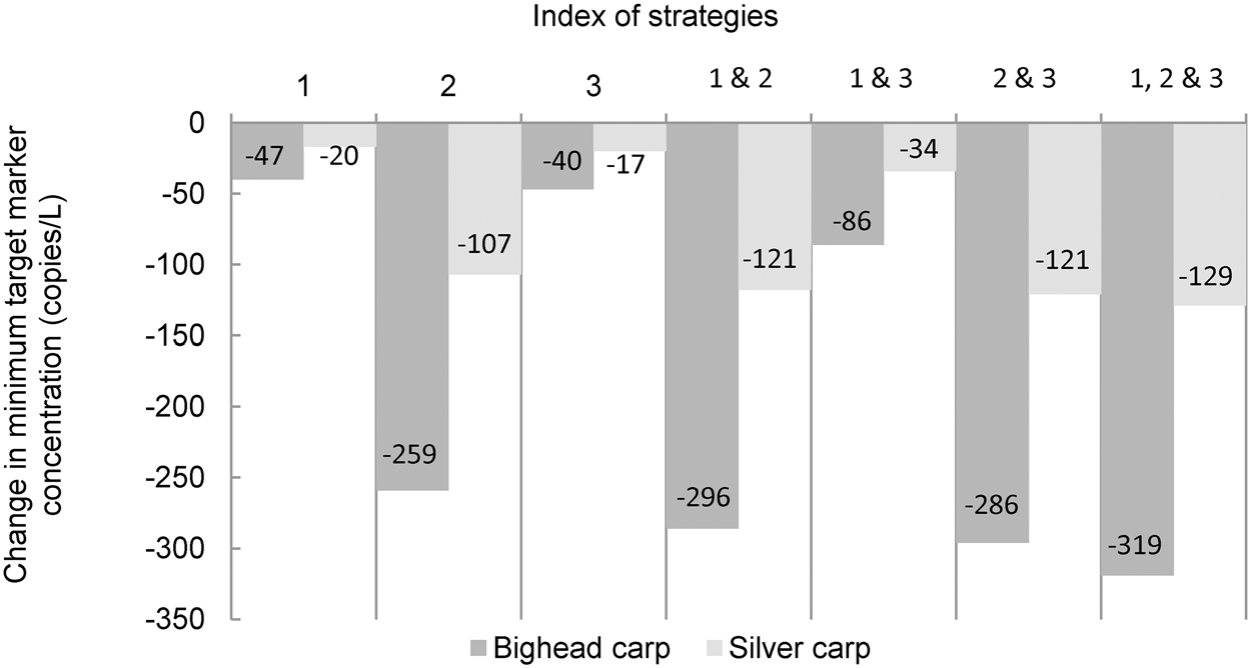
Reduction in the minimum target marker concentration that can be detected with probability 0.95. The figure shows to what extent changes in the baseline sampling protocol can reduce the target marker concentration that can be detected with probability 0.95. Changes in sampling protocol are as follows: 1) increase the number of samples from 10 to 11; 2) increase sample volume from 2 L to 3 L; and 3) increase the number of PCR replicates from eight to nine. The benefits of composite strategies (e.g., 1 & 2) are subadditive.

Changes in sampling protocol can be evaluated either in terms of an increase in sensitivity given the target marker concentration in the water body or a decrease in the minimum concentration that can be detected with a given sensitivity. The latter may be preferred because investigators will not know the ambient target marker concentration. For example, consider the baseline sampling protocol and a sensitivity goal of 0.95. The benefits of unit increases in replicate number, sample volume, and sample number are summarized in Fig 7. Among these three basic strategies, an increase in sample volume from 2 L to 3 L yields the largest reduction in minimum concentration that can be detected with probability 0.95. This strategy reduces the minimum bighead carp target marker concentration by 259 copies/L, from 789 copies/L to 530 copies/L, and reduces the minimum silver carp target marker concentration by 107 copies/L, from 322 copies/L to 215 copies/L. Fig 7 also shows that the effects of combined strategies, those that include increases in more than one sampling program parameter, tend to be subadditive. The most effective combined strategies include a unit increase in sample volume.

## Discussion

The simulation model enables investigators to estimate the sensitivity of an eDNA monitoring event and determine how proposed changes in sampling protocol may affect that sensitivity. Investigators should articulate the sensitivity of eDNA sampling protocols so that resource managers can better interpret results of surveys that fail to detect target species eDNA. An eDNA survey may fail to detect eDNA in a water body either because it is not present or because it is present at a concentration that is difficult to detect using the sampling protocol. Estimates of sensitivity developed using this simulation model enable resource managers to evaluate how likely it is that a particular sampling protocol would detect a target marker if that target marker were present at a specific concentration. It is particularly important to communicate the sensitivity of an eDNA survey when the results of that survey will be used to make important conservation management decisions.

Other studies have estimated the sensitivity of PCR as a means of detecting a target species genetic marker in processed environmental samples [3, 23, 26, 27]. The present study differs from these because it explores the sensitivity of the eDNA method to target marker concentrations in the environment. Other studies have explored the sensitivity of the eDNA method as a means of detecting a target species in aquatic habitats using species occupancy models [6, 10]. The present study differs from these because its emphasis is on detection of the target marker rather than detection of the target species, and because sensitivity is evaluated using a model that describes the process of sample collection and analysis rather than a species occupancy model. The model is implemented to simulate sensitivity of an eDNA survey at different target marker concentrations and to evaluate sampling protocols to detect the genetic marker of a target species. Ficetola *et al*. [11] have evaluated the sensitivity of sampling protocols using a species occupancy model to assess the sensitivity of metabarcoding studies. Like Ficetola *et al*. [11], the present study shows that increasing the number of replicates increases sensitivity, but the optimal number of replicates depends on the target marker concentration rather than the detection probability of taxa.

When using the eDNA method to detect a target species in waters where the presence of that species has not otherwise been established, it is reasonable to anticipate that the target species density may be low and that, as a result, eDNA concentrations will also be low. This reasoning is supported by studies that have documented a correlation between amphibian target species density and eDNA concentrations in ponds and streams [6, 8, 28, 29]. When target marker concentrations are low, processing of water samples and division of extraction elutions can yield PCR replicates with such low copy numbers that PCR may fail to detect the eDNA marker. Therefore, investigators should anticipate that eDNA monitoring surveys may have a high false negative rate where target species densities are low.

Results of model simulation show that PCR assays for the bighead carp target marker used in this study have a higher false negative rate than those for the silver carp target marker. This difference can be attributed to characteristics of the primers. The forward primer for the bighead carp eDNA marker, HN203-F, has a calculated melting temperature (T_m_) of 39.9°C. This is much lower than the melting temperature of the reverse primer HN498R-R, T_m_ = 58.4°C (Primer3Plus, [30]) and the annealing temperatures (T_a_) of PCR and sequencing, T_a_ = 50°C and T_a_ = 60°C, respectively. As a result, the bond between the forward primer and its template may be unstable during PCR and sequencing [31]. Both HN203-F and HN498R-R also exhibit a high degree of 3ʹ end self-complementarity. The Primer3 local alignment scores are 5.0 and 4.0 for these primers, respectively (Primer3Plus, [30]). High self-complementarity can lead to the primer binding to itself instead of the template, which reduces PCR and sequencing efficiency [32]. Other than self-complementarity in the reverse primer, the silver carp marker does not have these same design issues.

False negative rates obtained using simulation results are sensitive to assumptions about how the target marker is distributed in the water body. The Poisson distribution is appropriate if target markers are randomly distributed in the water column and the samples are independent. In general, these conditions will hold if that water body is well mixed and the samples are small relative to the volume of the water body from which they are taken. However, these conditions may not hold if there is spatial variability in the concentration. Spatial variability in the target marker concentration can be caused by target species habitat selection, hydrologic complexity, and persistence of eDNA within mitochondria, cells, or tissue fragments. The spatial extent over which the well-mixed assumption will hold will vary widely from system to system, but in general, the larger the spatial extent of a water body and the greater the variability in habitat types within the water body, the less likely it seems that the well-mixed assumption will hold. If the distribution of target markers in the water column is clumped rather than random, the number of target markers in water samples may tend to follow a negative binomial distribution rather than a Poisson distribution [21, 33, 34].

Under a negative binomial distribution, it is expected that the sensitivity of an eDNA monitoring event would be lower because a larger fraction of water samples would contain no target markers. With respect to designing sampling protocols for eDNA monitoring, the implication of having a negative binomial distribution is that a larger number of water samples will need to be collected to achieve a particular sensitivity goal. However, water samples that contain at least one target marker will contain a larger number of markers. Higher target marker counts in water samples translate into higher mean target marker counts in PCR replicates and an increased probability of fluorescence and successful sequencing.

This study has proposed that sensitivity goals can be expressed in terms of the minimum target marker concentration that can be detected with a desired probability, and that potential improvements in sampling protocol can be evaluated in terms of the potential reduction in that minimum target marker concentration. Some improvements will be more effective than others. Results of this study show that, among the potential improvements in sampling protocol considered in this paper, increases in sample volume yield the greatest potential benefit. The potential benefits of collecting samples with larger volumes are real, but so are the challenges associated with collecting larger samples. For example, when water samples are turbid or rich with algae, filtering and extracting DNA from large water samples can be difficult. Therefore, the costs of implementing a proposed change in sampling protocol should be considered in addition to the benefits. In this case, the same benefits might be achieved more cost-effectively by concentrating DNA extractions from several smaller water samples into a single DNA elution volume. Ultimately, the optimal sampling protocol will balance sensitivity, feasibility, and cost.

Several strategies are available to help reduce the false negative rate of an eDNA survey. Options include increasing the number of PCR replicates, increasing sample volume, increasing the number of samples, reducing elution volume in extraction, increasing extraction efficiency, and increasing the volume of elution in PCR replicates. The effectiveness of any one of these strategies will be difficult to estimate because it requires prior knowledge of the target marker concentration in the monitored water body and these will not be known when monitoring samples are collected. However, if invasive species are being targeted beyond the geographic limits of their known range, it is reasonable to assume that ambient concentrations of the target marker will be low. Therefore, sampling protocols should be designed to detect low target marker concentrations with a high probability. The model described in this study can be used to describe the effectiveness of a sampling protocol in terms of the minimum concentration that can be detected with the desired probability and to investigate how proposed changes in a sampling protocol might influence the sensitivity of an eDNA survey.

## Supporting Information

S1 File Quality Assurance Project Plan. (PDF)

S1 Table Fraction of PCR replicates causing fluorescence on an agarose gel. (PDF)

S2 Table Fraction of PCR replicates successfully sequenced for target markers. (PDF)
