# Supplementary material for: Modeling the Sensitivity of Field Surveys for Detection of Environmental DNA (eDNA)
Source: PLoS One. 2015 Oct 28;10(10):e0141503. doi: 10.1371/journal.pone.0141503 (PMC4624909; doi:10.1371/journal.pone.0141503)
Supplement: S1 File — (PDF) [file pone.0141503.s001.pdf]

DEPARTMENT OF THE ARMY
U.S. ARMY ENGINEER DIVISION, GREAT LAKES AND OHIO RIVER
CORPS OF ENGINEERS
550 MAIN STREET, ROOM 10032
CINCINNATI, OH 45202-3222

May 22, 2012

Dear Asian Carp Regional Coordinating Committee,

The Quality Assurance Project Plan (QAPP) for the Environmental DNA (eDNA) Monitoring of Invasive Asian Carp in the Chicago Area Waterway System (CAWS) outlines the detailed procedures for the planning, collection, filtering, processing, and reporting of eDNA samples. This document is the result of collaboration between USACE biologists and geneticists, and builds upon the initial protocols developed by researchers at the University of Notre Dame. This document has also undergone a technical review by scientists at Argonne National Laboratory, and many of their comments contributed to the document. The team acknowledges that there may be uncertainties with the methods outlined in this QAPP due to the evolving nature of the eDNA technology, the diverse nature of the aquatic environment from which the samples are taken, and the small amount of source biological material. The team recognizes these issues and will assess evolving information moving forward. The team will update this QAPP on a periodic basis to refine the methods and address uncertainties as new information becomes available.

In light of the urgency of ongoing eDNA sampling and evaluations, this QAPP focused on the main criteria of aligning and meeting the stated objectives of monitoring for genetic material in the CAWS.

Issues that are not addressed in this QAPP are:

1) Statistically defensible sampling protocol:
   The sampling protocol is currently designated by the Asian Carp Regional Coordinating Committee's Monitoring and Rapid Response Workgroup (MRRWG). This workgroup has created a Monitoring Plan with specific objectives to monitor for the genetic presence of Asian carp in designated areas of the CAWS. The two sampling approaches used by the MRRWG (and that are described in the QAPP) are targeted and transect sampling at specified locations chosen by the MRRWG. However, it is recommended that future versions of the Monitoring Plan be developed utilizing consistent scientific practices so that a statistically defensible plan can be implemented.

2) eDNA Testing Methodology:
   While the USACE team has compiled a detailed process for sampling/filtering and testing, the process outlined in this QAPP should not be considered final. The rigorous testing required to standardized collection sampling and analytical methods should be undertaken in order to confirm the precision of the eDNA methodology. Due to the high level of public concern regarding the possible spread of Asian carps into the Great Lakes via Lake Michigan, it is recommended additional testing and analysis necessary to develop a standardized method be undertaken in the near future. This additional testing is being conducted by the interagency eDNA Calibration Study team, comprised of scientists from the U.S. Army Corps of Engineers, U.S. Geological Survey, and U.S. Fish and Wildlife Service.

We expect that as our knowledge regarding eDNA continues to grow, the ability to refine the methodology will as well. Therefore, this QAPP will be updated on a periodic basis to reflect the progress made in calibrating eDNA as a monitoring tool, as well as any testing to validate the eDNA methodology. All results from the eDNA calibration study that will enhance or improve the methods for eDNA sampling, filtering, or processing will be implemented by modification of this QAPP. All results from the eDNA calibration study that will better inform sampling strategy will be sent to the MRRWG with a USACE endorsement to implement the recommendation.

Any questions regarding the content of the QAPP should be directed to Mr. Rich Hancock, LRD, eDNA Project Lead, at Richard.A.Hancock@usace.army.mil.

/S/

RICHARD A. HANCOCK, SES
Director
Regional Business Directorate

QUALITY ASSURANCE PROJECT PLAN

eDNA MONITORING OF INVASIVE ASIAN CARP IN THE CHICAGO AREA WATERWAY SYSTEM

Prepared for:

U.S. Army Corps of Engineers
Chicago District
Chicago, Illinois

May 2012

US Army Corps
of Engineers ®

TABLE OF CONTENTS

Section No. **Page No.**

SECTION 1 **1-1**

1. PROJECT DESCRIPTION AND PERSONNEL REQUIREMENTS **1-1**
1.1 Background 1-1
1.2 General Requirement 1-1
1.3 Project History 1-2
1.4 Objective 1-2
1.5 Project Personnel 1-2
1.6 Reporting 1-4

SECTION 2 **1-1**

2. SAMPLE COLLECTION **2-1**
2.1 Pre-Trip Planning and Site Selection 2-1
2.1.1 Purpose 2-1
2.1.2 Pre-trip Planning Procedure 2-1
2.1.3 Field equipment 2-3
2.1.4 Site Selection Procedure (in the field) 2-4
2.2 Boat And Equipment Preparation 2-7
2.2.1 Purpose 2-7
2.2.2 Equipment Procedure 2-7
2.2.3 Boat Preparation Procedure 2-8
2.3 Sample Collection Procedure 2-9
2.3.1 Purpose 2-9
2.3.2 Water Collection Procedure 2-9

3. SAMPLE FILTERING **3-12**
3.1 Purpose 3-12
3.2 Filtering Procedure 3-12
3.3 Laboratory Preparation 3-13
3.4 Sample Preparation 3-13
3.5 Filtering the Equipment Control 3-13
3.6 Filtering the Sample 3-14
3.7 Preparing and Filtering A Positive Equipment Control 3-16
3.8 Equipment and Work Area Cleaning After Filtering Each Sample 3-16

4. SAMPLE SHIPMENT **4-1**
4.1 Purpose 4-1
4.2 Shipping Procedure 4-1

5. DNA ASSAYS 5-1
5.1 General Quality Assurance and Chain-of-Custody Considerations.. 5-1
5.2 Quality Control for Sample Custodian Procedure and Storage 5-3
5.3 Physical Separation of Pre-PCR and Post-PCR Assay Stages 5-3
5.3.1 The eDNA Extraction Room 5-3
5.3.2 Pre-PCR Room 5-3
5.3.3 Product Analysis Room 5-4
5.4 Receipt of Filters 5-4
5.4.1 Source 5-4
Water samples have been taken in the field according to Section 2 and filtered according to Section 3. Filters have been shipped to ERDC and personnel assigned to the ERDC eDNA Team have either received or picked-up shipping boxes from the ERDC Receiving Office. 5-4
5.5 DNA Extraction from Filters 5-5
5.5.1 Source 5-5
5.5.2 DNA Extraction Quality Assurance and Chain of Custody 5-5
5.5.3 Procedure 5-6
5.6 PCR Amplification of eDNA Samples.. 5-8
5.6.1 Purpose 5-8
5.6.2 Source 5-8
5.6.3 PCR Quality Assurance and Chain of Custody 5-8
5.6.4 Procedure 5-9
5.7 Gel Electrophoresis of eDNA PCR Assays... 5-11
5.7.1 Purpose 5-11
5.7.2 Source 5-12
5.7.3 Gel Electrophoresis Assurance and Chain of Custody 5-12
5.7.4 Option A Procedure 5-12
5.7.5 Option B Procedure 5-13
5.8 eDNA Gel Documentation and Storage 5-14
5.8.1 Purpose 5-14
5.8.2 Source 5-14
5.8.3 Gel Documentation and Storage Assurance and Chain of Custody 5-14
5.8.4 Procedure 5-14
5.9 Gel Interpretation 5-15
5.9.1 Purpose 5-15
5.9.2 Source 5-15
5.9.3 Gel Interpretation Quality Assurance and Chain of Custody 5-15
5.10 Confirmation of Positive Results.. 5-15
5.10.1 Purpose 5-15
5.10.2 Source 5-15
5.10.3 Gel Interpretation Assurance and Chain of Custody 5-16
5.10.4 Procedure 5-16
5.11 Communication of eDNA Assay Results from ERDC to USACE 5-18

5.11.1 Purpose 5-18
5.11.2 Source 5-18
5.11.3 Quality Control 5-18
5.11.4 Procedure 5-18

6. INTERNAL QUALITY CONTROL CHECKS 6-21
6.1 Laboratory Quality Control Evaluation Criteria 6-21

7. SPECIFIC ROUTINE PROCEDURES TO ASSESS DATA PRECISION, ACCURACY, AND COMPLETENESS 7-1
7.1 Field Measurements 7-1
7.2 Laboratory Data 7-1

8. CORRECTIVE ACTIONS 8-1

9. PREVENTATIVE MAINTENANCE PROCEDURES 9-1
9.1 Field Equipment/Instruments 9-1
9.2 Laboratory Instruments 9-1

10. PERFORMANCE AND SYSTEM AUDITS 10-1
10.1 Field Audits........ 10-1
10.2 Laboratory Audits........ 10-1

TABLE OF CONTENTS (Continued)

LIST OF TABLES

Table No. **Page**

Table 2-1. Descriptions of sample reaches, designation of sample groups, and basic number of samples to be collected during sampling trips. 2-6
Table 2-2. Boat launch information. 2-6
Table 2-3. Lockmaster contact information for Illinois River and CAWS locks........................... 2-7
Table 5-1. List of critical components for eDNA Processing, and component information for ERDC. No vendor is listed for generally available supplies.. 5-19

LIST OF FIGURES

Figure No.

Figure 2-1. General locations of sample reaches within the Chicago Area Waterway System. Specific reach locations and descriptions are found in Table 2-1. The specific sampling locations will be chosen by the MWWRG and/or the sampling agencies working in partnership. Specific sampling locations and the rationale for those locations will follow the general protocol given above. Each specific sampling location will be identified using a GPS device and the location and characteristics will be recorded on the logs given in the Exhibits ……………………………………………………………………………… 2-5

LIST OF EXHIBITS

Exhibit No.

1 Form 1 – Field Collection Summary Datasheet

2 Form 2 – Field Sample Summary Datasheet

3 Form 3 – Chain of Custody Record

4 Sample Receipt Checklist

5 Refrigerator Logs

6 PCR Log

7 QAPP Compliance Certification Statement

LIST OF APPENDICES

Appendix	
A	Staff Assignments
B	Monitoring and Rapid Response Work Group (MRRWG) Monitoring Plan
C	Sampling Plans for the Current MRRWG eDNA Sampling Strategy
D	Data Management

ACRONYMS/ABBREVIATIONS

CAWS	Chicago Area Waterway System
COC	Chain of custody
CSO	combined sewer overflow
CSSC	Chicago Sanitary and Ship Canal
DI	deionized (water)
DNA	Deoxyribonucleic acid
dNTP	deoxyribonucleotide triphosphates, also known simply as nucleotides
eDNA	environmental Deoxyribonucleic acid
GPS	Global Positioning System
LDB	left descending bank
LRC	Chicago District, USACE
MRRWG	Monitoring and Rapid Response Work Group
MSDS	Material Safety Data Sheets
PCR	polymerase chain reaction
QAPP	Quality Assurance Project Plan
RDB	right descending bank
Taq	*Thermus aquaticus*
USACE	United States Army Corps of Engineers
UV	ultraviolet

SECTION 1

1. PROJECT DESCRIPTION AND PERSONNEL REQUIREMENTS

1.1 Background

Invasive aquatic nuisance species pose a major threat to aquatic ecosystems worldwide. Within Illinois, the manmade Chicago Sanitary & Ship Canal (CSSC), constructed in the early 1900s, provided an unnatural portal for invasive species dispersal between the geologically separated Mississippi River and Great Lakes drainage basins. In 2002, in an effort to curtail the spread of invasive species between the two basins, the U.S. Army Corps of Engineers (USACE), constructed a dispersal barrier system within the CSSC. The primary objective of the barrier system when initiated was to stop the dispersal of the invasive round goby into the Mississippi River basin; however, once the project was completed, it was found that the round goby had already surpassed the barrier. Since then, a new threat to the Great Lakes from the Mississippi River basin has become the primary objective of the dispersal barrier system. Invasive Asian carps, including bighead carp (*Hypophthalmichthys nobilis*) and silver carp (*H. molitrix*) have been steadily dispersing upstream through the Mississippi, Illinois, and Des Plaines Rivers. Their potential dispersal through the dispersal barrier system within the CSSC poses a potential threat to the Great Lakes ecosystem. In the past, traditional fishery techniques were used to detect the leading edge of the Asian carp population; however, this method was somewhat ineffective at targeting these species at low densities. The University of Notre Dame, with funding from the USACE, developed a method that detected “environmental” DNA (eDNA) left behind in the aquatic system by the targeted species. Environmental DNA enters the system through a variety of mechanisms, some of which include sloughing of external epidermal cells into the water, sloughing of internal epidermal cells into feces and into the water, and as tissue residues following injury or predation. The detection of eDNA in water samples is based on whole DNA extraction from filtered particulate organic and inorganic matter found in the water and polymerase chain reaction (PCR) assays for species-specific mitochondrial DNA markers. Use of this method is to provide detection of Asian carp at low densities and to serve as an early detection system of the spread of Asian carp into previously uninhabited areas. As USACE takes over the lead on eDNA monitoring from the University of Notre Dame, a Quality Assurance Project Plan detailing the eDNA monitoring process, including methodologies and quality controls, has been requested from the U.S. Army Engineer Research and Development Center (ERDC).

1.2 General Requirement

The Chicago District (LRC) requires a Quality Assurance Project Plan (QAPP) for eDNA monitoring. Full-scale eDNA monitoring commenced in September 2010. Based on consultation with experts in processing of litigable DNA evidence (which applies to eDNA monitoring), a final, comprehensive QAPP was not in place within that time frame. Instead a provisional QAPP was used during the first year of eDNA monitoring, to be followed later by a finalized version under

a different scope of work. This allows for inclusion of any beneficial technical or strategic modifications that become apparent during the first year of effort.

1.3 Project History

The University of Notre Dame, Department of Biological Sciences, Center for Aquatic Conservation, prepared a Standard Operating Procedure (SOP) in 2010. The SOP provided details regarding eDNA monitoring protocol and was given to USACE in May 2010 in compliance with Cooperative Ecosystem Study Unit agreement #W912HZ-08-2-0014, modification P00007. On 15 and 16 December 2009, a technical and quality systems audit of the Center for Aquatic Conservation Lodge Laboratory at the University of Notre Dame was conducted by USEPA. The laboratory audit report dated 5 February 2010 was provided to USACE in addition to the eDNA monitoring protocol. These documents served as the basis for this provisional QAPP.

1.4 Objective

The objective of this QAPP is to provide detailed procedures for Asian carp eDNA sample collection, sample processing (including filtering, DNA extraction, PCR, biomarker analysis, DNA sequencing), data reporting, and quality control/quality assurance procedures for 2011, to ensure that data are as technically defensible, consistent, and usable as possible. The specific goals and objectives of sampling are currently directed by the Asian Carp Regional Coordinating Committee's (ACRCC) Monitoring and Rapid Response Plan.

1.5 Project Personnel

The eDNA monitoring project must have personnel appointed to the following positions:

- eDNA Program Manager
- Sampling Quality Assurance Specialist
- Filtering Leader
- Filtering Quality Assurance Specialist
- DNA Processing Leader
- DNA Processing Quality Assurance Specialist
- Data Documentation & Reporting Specialist
- Supporting Agency contacts.

The minimal responsibilities of the above positions are detailed below. Specific personnel assigned to the project are listed in Appendix A.

eDNA Program Manager: Responsible for ensuring water samples for eDNA monitoring are collected and provided to the filtering team. Responsible for reporting results to Filtering and DNA Processing Team Leaders, as well as other designated USACE personnel and the Federal Executives of the ACRCC.

Sampling Quality Assurance Specialist: Responsible for knowing all quality assurance/quality control (QA/QC) measures for eDNA sampling efforts. Advises eDNA Program Manager on any potential QA/QC problems. Reviews procedures, field logs, data collection methodology;

ensures that all agencies participating in sampling are conforming to procedures. Recommends corrective actions for non-conformities. Coordinates sampling crews for collection of samples.

Filtering Leader: Responsible for filtering water samples for eDNA monitoring and providing those filter samples to eDNA Processing team. Responsible for reporting results to Sampling and DNA Processing Team Leaders, as well as other designated USACE personnel.

Filtering Quality Assurance Specialist: Responsible for knowing all QA/QC measures for filtering efforts. Advises Filtering Leader on any potential QA/QC problems. Reviews procedures, laboratory logs, and documentation for filtering; ensures all personnel are conforming to procedures. Recommends corrective actions for non-conformities.

DNA Processing Leader: Responsible for processing eDNA filter samples through extraction, PCR, and sequencing. Responsible for reporting results to Sampling and Filtering Team Leaders, as well as other designated USACE personnel.

DNA Processing Quality Assurance Specialist: Responsible for knowing all QA/QC measures for eDNA processing efforts. Advises eDNA processing Leader on any potential QA/QC problems. Reviews procedures, laboratory logs, and documentation for DNA processing; ensures all personnel are conforming to procedures. Recommends corrective actions for non-conformities.

Data Documentation & Reporting Specialist: Assists DNA Processing Leader in maintaining shared database for eDNA sample processing. Performs data completeness and data verification checks, and ensures that all data are documented completely (completed by Sampling and Filtering Leads).

Assigned Project Leaders and Specialists: Others serving on the project may include researchers, technicians, and budgetary personnel. Sampling may employ personnel from other agencies in coordinated efforts. All personnel must meet a minimum standard for training and/or experience before independently conducting any portion of the eDNA monitoring protocol. The supporting agency contacts are given in Appendix A. Minimum personnel training requirements are given below.

Personnel Training Requirements

Minimum training and/or experience requirements for the different major components of the eDNA monitoring protocol are detailed below.

Boat Operator:

- Must meet USACE boat operator requirements as a minimum. USACE Boat Operator requirements are listed in Engineer Manual 385-1-1.

Sampling:

- A BA/BS degree or its equivalent in biology or related field of study, or

- At least 2 years of specialized postsecondary training or an associate degree in applied science or science-related technology, or
- A high school diploma or its equivalent and a minimum of 2 years professional experience in biology-related field.
- First aid and/or boating safety course.
- Minimum 1 year experience in collecting field samples for biological analyses.

Filtering:

- A BA/BS degree or its equivalent in biology or related field of study, or
- At least 2 years of specialized postsecondary training or an associate degree in applied science or science-related technology, or
- A high school diploma or its equivalent and a minimum of 2 years professional experience in biology-related field.
- First aid training.
- Facility-specific safety training.
- Minimum one semester college level laboratory experience, plus eDNA-specific training.

DNA Processing:

- A minimum BA/BS degree or its equivalent in biology or related area and successful completion of college course work (graduate or undergraduate level) covering the subject areas of biochemistry, genetics, and molecular biology (molecular genetics, recombinant DNA technology) or other subjects that provide a basic understanding of the foundation of DNA analysis, as well as course work and/or training in PCR amplification as it applies to eDNA analysis.
- A minimum of 6 months of general DNA laboratory experience, including experience with DNA extraction and PCR. Additionally, 2 weeks of training on Asian carp eDNA protocols.
- Successful completion of a qualifying test demonstrating effective execution of eDNA-type assays before beginning independent work on the project.

1.6 Reporting

All agencies, including USACE offices, need to submit resumes (Curriculum Vitaes) for proposed staff, to be reviewed and approved by LRC and the eDNA Program Manager. Those documents will be kept with the project file. For each sampling event, specific staff members conducting the various activities will be documented (e.g., on the field sampling log). That information will also become part of the project file.

SECTION 2

2. SAMPLE COLLECTION

Prior to any field sampling work, all field employees must review this quality assurance plan and acknowledge the procedures and processes to be followed for every sample and every event. Field employees will acknowledge their understanding and intent to comply by signing the certification form given as Exhibit 7. Field employees will also review the sampling safety plan (separate document) and participate in a safety briefing.

Prior to any filtering or analysis work, all laboratory employees must review this quality assurance plan and acknowledge the procedures and processes to be followed for every sample and every event. Laboratory employees will acknowledge their understanding and intent to comply by signing the certification form given as Exhibit 7. Laboratory employees will also review the laboratory safety plan (separate document) and participate in a safety briefing.

2.1 Pre-Trip Planning and Site Selection

2.1.1 Purpose

Accurate planning of a general collection site is necessary to effectively manage the time of crews collecting samples, as well as to ensure complete and correct sampling procedures are used. Sampling will occur within the Chicago Area Waterway System (CAWS), which includes but is not limited to the following named waters: Illinois River, Des Plaines River, CSSC, Chicago River, Bubbly Creek, North Branch of the Chicago River, North Shore Channel, Cal-Sag Channel, Little Calumet River North Leg, Little Calumet River South Leg, Grand Calumet River, Lake Calumet, and the Calumet River. Reaches to be sampled within the CAWs have been designated (Figure 2-1) and a general description and location (river mile) of each reach is given in Table 2-1. Overall, the reaches to be sampled and number of samples to be taken depends upon guidance from the Asian Carp Monitoring and Rapid Response Work Group (MRRWG); however, the baseline number of samples to be taken from each reach is 60. Prior to sampling, aerial imagery and a site visit should be used to determine where (generally) samples will be collected and to establish access points and gain any necessary permissions to access the river. Specific site selection will be determined in the field per the following guidelines. Table 2-2 contains boat launch information.

2.1.2 Pre-trip Planning Procedure

USACE will determine the reach to be sampled, the number of samples to be taken within the reach, and confirm with MRRWG. USACE will provide this information to the sampling agency.

(1) Reaches to be sampled on a monthly basis include the North Shore Channel, Chicago River to South Branch Chicago River, and Little Calumet River North Leg to Lake Calumet. Reaches to be sampled on a periodically include the CSSC upstream and downstream of the barrier, CSSC and Cal-Sag Channel confluence, and the Upper Des Plaines River (Table 2-1).

(2) The baseline number of samples to be collected from each reach is 60 with at least 1 cooler blank sample per sample cooler.

(3) LRC will seek input from MRRWG prior to each sampling trip to confirm the number of samples to be taken from a reach and the reach to be sampled. The number of samples and the reach to be sampled will be provided to the sampling agency by LRC.

Use interactive aerial imagery software (i.e., Google Earth) to scope out reaches to be sampled, placement of samples, and unique features that should be targeted during sampling.

(1) Aerial maps will be detailed enough to show unique features (e.g., barge slips, factory, etc.) that can be identified in the field and used as markers for location when sampling. The recommended minimum scale is 1':500'.

(2) Aerial maps must be marked with sample locations and should ensure spatial coverage and overall representativeness of the sample area.

(3) Target specific areas (backwaters, island side channels, pooled areas, below and around structures, confluence of tributaries) as well as integrating transect plots in the sampling plan. Transect plots should be spaced approximately 500 meters apart.

(4) Print map(s) with detailed sample plan.

- Locate access points for boat launch and acquire permission to use if necessary. Table 2-2 provides information on boat launch locations, permission contacts, fees, and reaches that can be accessed by a specified launch. The agency that is conducting the sampling event is responsible for coordinating boat launches or any access or real estate issues associated with the sampling.

- If sampling around locks, or if sampling will require lockage, notify the Lockmaster at least 1 day before sampling. Table 2-3 provides Lockmaster contact information. The agency that is conducting the sampling event is responsible for coordinating with the Lockmaster.

- Coordinate sample plan with sample crew, which will comprise four people at a minimum: one boat operator, one lead sampler, and two sampling assistants. All participants involved in the sampling must have their resume (CV) on file with LRC, prior to the sampling event. All participants involved in the sampling must have read this QAPP and must have a signed certification statement (Exhibit 7) on file with LRC, prior to the sampling event. All participants involved in the sampling must meet the minimum qualifications given for their role in Section 1.3 of this document.

- A field equipment checklist (Exhibit Form 1) and datasheets (Exhibit Form 2) should be printed prior to each sampling trip on Rite-in-the-Rain® paper or other waterproof paper. Datasheets are printed on front and back.

- Check river stage and weather forecast.

(1) No eDNA sampling should occur within 5 days after a significant rainfall event (more than 1.5 inches in a 24-hr period), on the rising limb of a hydrograph of the river as it exceeds flood stage, and/or within 2 days of a combined sewer overflow (CSO) event. Weather data and river stage for the area to be sampled can be checked at: http://waterdata.usgs.gov/nwis. The occurrence of a CSO event can be verified by contacting the eDNA Program Manager listed in Appendix A.

2.1.3 Field equipment

(1) Minimum 18-ft boat with specified motor, including trailer and vehicle to pull, provided by the sampling agency.

(2) Personal flotation devices for each crew member, using the type of devices listed in the safety plan. Minimum PFD requirements are Type I within the Safety Zone near the existing electric barrier (on the Chicago Sanitary and Ship Canal) or Type II for the remainder of the CAWS.

(3) 100-qt coolers (each cooler capable of holding 20 2L samples); provided cleaned and filled with bottles by USACE. Sampling agency picks up bottles at 536 S. Clark Street, 10th Floor, Chicago, Illinois.

(4) 2L sample bottles (label affixed and numbered in the lab), provided cleaned and labeled by USACE in the coolers.

(5) 3-gallon sprayer

(5) Habitat measurement equipment (Global Positioning System [GPS], Digital Depth Sounder); provided by the sampling agency.

(6) Field datasheets (Forms 1 and 2) provided by USACE with the coolers and bottles.

(7) Chain of custody form (Form 3) provided by USACE with the coolers and bottles.

(8) Sharpie® permanent marker in black provided by the sampling agency.

(9) Powderless nitrile gloves provided by the sampling agency.

(11) Ice provided by the sampling agency.

(12) Drinking water for crew provided by the sampling agency.

(13) Safety plan – USACE plan represents minimum requirements; agency-specific alternative plans are allowable as long as all hazards are addressed and minimum requirements are met.

2.1.4 Site Selection Procedure (in the field)

Using the sampling plan as guidance, refinement of exact sampling should depend on the following factors:

(1) Sampling should occur in a downstream to upstream direction to minimize the potential for surface water disturbance caused by the vessels wake within the sample reach. The only exception where sampling may occur in an upstream to downstream direction would be if the nearest boat launch is upstream of the reach to be sampled. Sample direction should be noted on the Field Collection Summary datasheet (Form 1).

(2) Samples will be collected in two ways – transect and targeted sampling.

(a) Transect: Location of transects will be determined by LRC prior to the start of a sampling event. The first transect will be set across the downstream end of the reach to be sampled with subsequent transects set 500 m apart heading upstream (see exception to protocol above (1)). Transects will run perpendicular to flow, and three 2L samples will be collected along each transect using the following scheme: one collected near the left descending bank (LDB), one in mid-channel (MC), and one near the right descending bank (RDB). Where samples are collected should be recorded in the "Habitat" column of the Sampling datasheet (Form 2). Samples should be collected on the upstream side of the boat or off the bow.

- When collecting samples near the bank, be observant of wake-disturbed surfaces. To compensate for the wake created by a passing boat, samples may need to be collected 2–3 ft off the bank to obtain displaced surface film.

(b) Targeted sampling is collecting samples in the most probable places of eDNA accumulation, such as (but not limited to):

- Backwater areas
- Island side channels
- Confluences of tributary waters
- Effluent areas
- Barge slips
- Eddies or pooled areas
- Near structures that create slack-water (e.g., sunken barges)
- Bays
- Below Lock and Dam structures
- Other areas where organic material has accumulated on the water surface

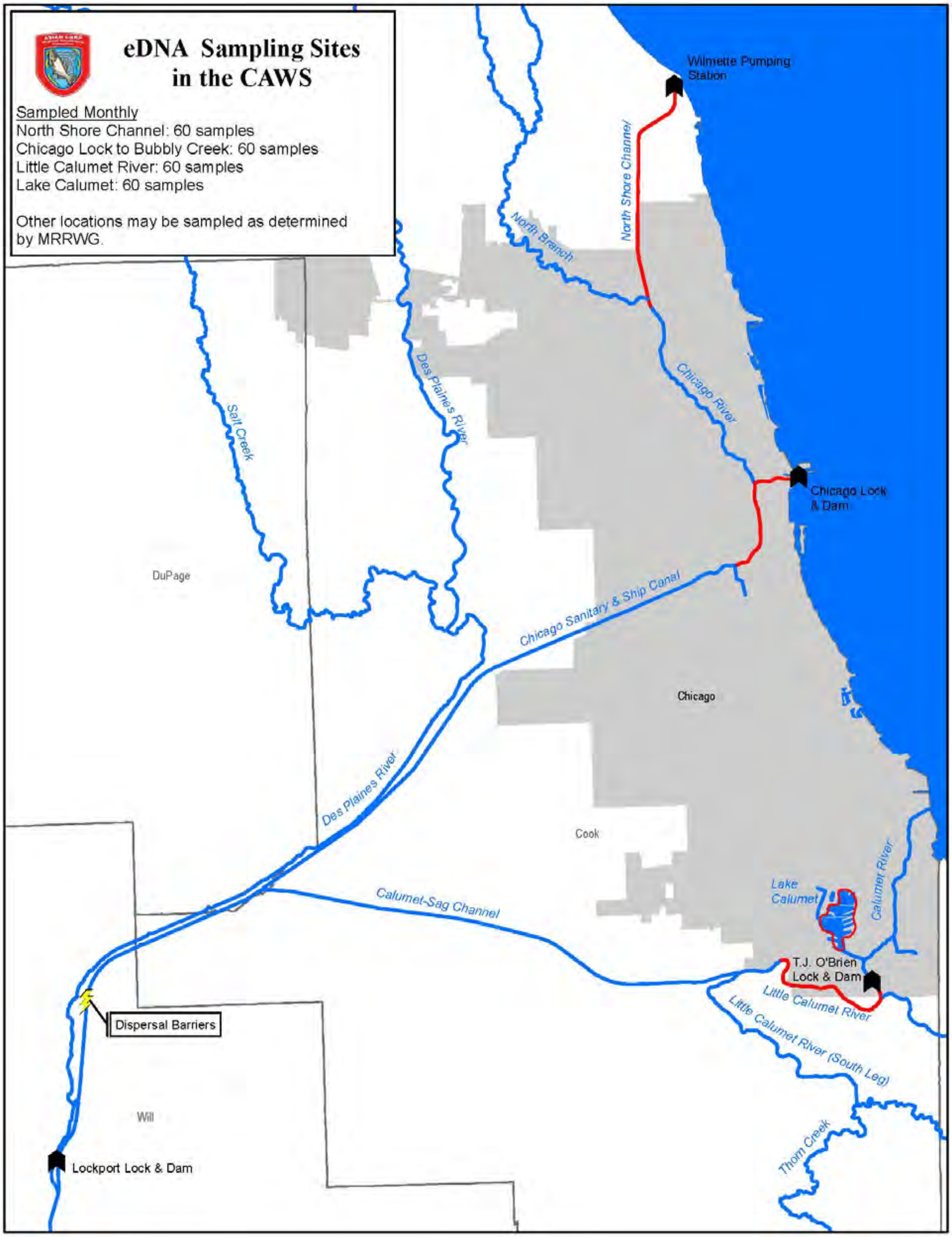

Figure 2-1. General locations of sample reaches within the Chicago Area Waterway System. Specific reach locations and descriptions are found in Table 2-1. The specific sampling locations will be chosen by the MWWRG and/or the sampling agencies working in partnership. Specific sampling

locations and the rationale for those locations will follow the general protocol given above. Each specific sampling location will be identified using a GPS device and the location and characteristics will be recorded on the logs given in the Exhibits .

Table 2-1. Descriptions of sample reaches, designation of sample groups, and basic number of samples to be collected during sampling trips.

Reach ID	Description	River Miles	# of Samples	# of Blanks
CR/SBCR	Chicago Lock to Bubbly Creek, including the Chicago River (Chicago Lock; RM 326.7) and South Branch Chicago River (RM 321.5)	5.2	60	6
NSC	North Shore Channel from the Wilmette Control Works to the North Branch Chicago River (RM 333.45)	2	60	6
LCAL/LCALR	Little Calumet River North Leg downstream of O'Brien Lock (RM 326.5) to Acme Bend (RM 321) and within Lake Calumet	4.5	120	6
LPA	Within the Lockport Pool above the electric barrier but below the confluence with the Cal-Sag Channel (RM 296.2 to RM 300.5)	4.3	60	3
LPB	Within the Lockport Pool but below the electric barrier (RM 291.0 to RM 296.2)	5.2	60	3
CSSC/CALSAG	Northern section of the CSSC from the HWY 43 Bridge to the Ashland Ave. Bridge (RM 300.5 to RM 305.0)	4.5	60	6
DPR-RVS	Des Plaines River from Riverside Station downstream to Ogden Avenue	2	40	2
DPR-CWS	Des Plaines River from Gilbert Avenue downstream to South County Line Road	2	40	2
DPR-LRS	Des Plaines River from 355 Tollway upstream to Lemont Road	2	40	2

Table 2-2. Boat launch information.

Launch	Location	Owner	Contact	Fee	Access For:
Burnham Harbor	Chicago, IL	Public	312-747-7009	$20	NSC
Western Avenue	Chicago, IL	Public	NA	$25	NSC, CR/SBCR
Village of Summit	Summit, IL	Public	708-588-4223	$15	LPA, CSSC/CALSAG
Village of Worth	Worth, IL	Public	708-448-3979	NA	LPA, CSSC/CALSAG, LCAL/LCALR
Village of Alsip	Alsip, IL	Public	708-385-6902	NA	LPA, CSSC/CALSAG, LCAL/LCALR
Beaubien Forest Preserve	Altgeld, IL	Public	708-403-6951	NA	LCAL/LCALR
O'Brien Lock and Dam	Chicago, IL	USACE	773-646-2183	NA	LCAL/LCALR
Cargill	Lockport, IL	MWRD	312-751-5133	NA	LPB

Table 2-3. Lockmaster contact information for Illinois River and CAWS locks.

Lock	River Mile	Location	Telephone Number
LaGrange	80	Versailles, IL	217-225-3317
Peoria	158	Creve Coeur, IL	309-699-6111
Starved Rock	231	Ottawa, IL	815-667-4114
Marseilles	245	Marseilles, IL	815-795-2593
Dresden Island	272	Morris, IL	815-942-0840
Brandon Road	286	Joliet, IL	815-744-1714
Lockport	291	Lockport, IL	815-838-0536
O'Brien	327	Chicago, IL	773-646-2183
Chicago	327	Chicago, IL	312-787-4795

2.2 Boat and Equipment Preparation

2.2.1 Purpose

In order to perform laboratory molecular analyses to detect eDNA, vessels and equipment must be sanitized in accordance with the following protocols to eliminate introduction of outside DNA sources in the sampling regime.

Cautions: Precautions should be made to avoid direct skin contact with hypochlorous acid products; hypochlorous acid solution may also stain clothing or other materials. Be aware of pollutants in the aquatic environment and related health hazards.

2.2.2 Equipment Procedure

(1) A 10% hypochlorous acid solution with deionized (DI) water will be prepared in a 3-gallon hand-held sprayer that is dedicated to the project. The hypochlorous acid solution must be prepared immediately prior to use, and each time disinfection activities will be occurring.

(2) At least 1 day prior to use, sample transport coolers will be sterilized with freshly made 10% hypochlorous acid solution by LRC. Using the hand-held sprayer, the inside surfaces of the cooler will be thoroughly covered with the hypochlorous acid solution. At least 10 min of contact between the solution and the inside surfaces of the cooler will be allowed before rinsing with DI water. Coolers will then be left to air dry.

(3) Sterilized disposable 2L bottles may be used for sample collection or 2L autoclavable reusable bottles sterilized in the following way may be used:

(a) Reusable 2L bottles will first be thoroughly rinsed with DI water to remove any material that may be on the outside or inside of the bottle and cap.

(b) After rinsing the bottles, lightly screw on cap (cap will not be able to come off, but will still be able to move).

(c) Place capped 2L bottles into autoclave and set cycle for 1 hour at 121°C (15 psig (1 bar)).

(d) Once the autoclave cycle is complete, carefully remove bottles and allow them to cool to room temperature before fully screwing on the caps.

(4) Once all 2L bottles have been sterilized, sample labels will be affixed to the outside of the bottles prior to going into the field. Bottle labels will be provided by LRC. Bottles will be labeled with an appropriate ID that does not indicate location (to allow blind processing). The numbering scheme will be as follows: YYYYMMDD-SSS, where YYYY is the year (such as 2010), MM is the month (such as 09 for September), DD is the day (such as 04 for the fourth), and SSS is the sample ID number (such as 001, 002, 003, etc). An example for a sampling event scheduled for 11 June 2009 would be: 20090611-001, 20090611-002, etc. The SSS numbers will be consecutive starting from 001 and increasing to the maximum number of samples taken that day (999). Labels will be printed on Rite-N-Rain® or some type of waterproof paper and affixed by LRC to the outside of the sample bottles using clear duct tape prior to the initiation of field activities.

(5) Once bottles have been labeled, they will be placed in the sterilized sample coolers in numerical order. Sample bottles will be stored in the sterilized coolers until use, and will be transported only in the coolers. Although the number of the sample is not relevant except for identification purposes, collecting in consecutive order will aid in determining where samples were taken in case of a recording error.

(6) Two samples out of approximately every 25 will serve as control samples or at least one control per cooler. Control samples will be chosen randomly and will be filled with DI water by LRC before coming into the field. The baseline number of control samples needed for each reach is listed in Table 2-1.

(7) Six samples out of every 120 samples collected will serve as duplicate samples. Duplicate samples will be determined prior to sampling and their locations will be identified on the aerial maps. At these designated areas, a duplicate sample will be taken in tandem with a regular sample.

2.2.3 Boat Preparation Procedure

(1) Vessels to be used for eDNA sample collection must be washed with a commercial power washer and detergent prior to the disinfection process. All detergent must be thoroughly rinsed and the boat must be allowed to air dry 1 day prior to disinfection. A 10% hypochlorous acid solution with DI water will be prepared in a 3-gal hand-held sprayer that is dedicated to the project. The hypochlorous acid solution must be prepared immediately prior to use, and each time disinfection activities will be occurring.

(2) The outer surfaces of the sampling vessel (i.e., hull, motor, etc.) as well as the transport trailer will be thoroughly rinsed with the prepared hypochlorous acid solution using a hand-held sprayer. Outer surfaces should be thoroughly covered with the hypochlorous acid solution and left in contact with no rinsing. Three gallons of solution may not be sufficient to rinse the entire boat and trailer, and additional batches of hypochlorous acid solution must be mixed when needed to ensure thorough rinsing of the hull, trailer,

motor, etc. A minimum of 10 minutes contact time must be used for the boat to allow sufficient time for the hypochlorous acid solution to oxidize organic matter that is present on the boat.

2.3 Sample Collection Procedure

2.3.1 Purpose

In order to perform laboratory molecular analyses to detect eDNA, samples must first be collected from the appropriate aquatic environment in accordance with the following protocols.

Cautions: Lifejackets must be worn at all times in transport vessels (boats). Additionally, disposable latex or nitrile gloves must be worn when collecting water samples and measuring water depth and temperature. Be aware of pollutants in the aquatic environment and related health hazards.

2.3.2 Water Collection Procedure

(1) Prior to launch, crew members will have reviewed this QAPP, will have signed the QAPP certification form, and will understand their assigned roles in the sample collection procedure. All sampler identification information and other field data will be recorded in pencil on the Field Collection Summary (Exhibit Form 1).

(2) The transport vessel will be launched from an appropriate area (such as those locations listed in Table 2-2) that allows access to the reaches to be sampled.

(3) Sampling will commence at the first transect located at the downstream end of the reach to be sampled and will proceed in an upstream direction. The only exception to this protocol is when the boat launch is located upstream of the sampling reach. Then sampling will commence at the first transect located at the upstream end of the reach to be sampled and will proceed in an upstream direction. The direction traveled for sampling should be recorded on the Field Collection Summary (Exhibit Form 1).

(4) When arriving at a sample location (within either a transect or targeted area), the lead sampler and sampling assistant 1 will put on sterile exam gloves (powderless latex or nitrile). **REMINDER – Gloves must be changed before each new transect is taken to prevent cross contamination. The same gloves may be worn when collecting duplicate or blank samples in tandem with a regular sample in a transect.**

(5) Going in consecutive numerical order based on the bottle labels, the lead sampler will remove a labeled 2L sample bottle from the sample cooler.

(6) Just prior to collecting the sample, the lead sampler will unscrew and remove the lid from the sample bottle.

(7) The lead sampler will then reach over the upstream side or the bow of the transport vessel with the 2L sample bottle and fill the bottle by skimming the surface of the field

water. The sample bottle must not be submerged or dipped beyond the upper 2 inches of the surface water for sample collection, since the intent of the sampling is to collect floatable materials that are in the water surface.

(8) Once the sample bottle is filled (approximately 1 in. of space should be left within the sample bottle), the lead sampler will screw the lid back on to the bottle until it is tight. The closed bottle will then be returned to the sample cooler from which it was removed.

(9) While the lead sampler is collecting the water sample, sampling assistant 1 will take habitat measurements: water temperature, depth, GPS coordinates in Decimal Degrees, military time of sample, location (e.g., LBD, center, RBD), and relate the information to sampling assistant 2, who will record the information on the datasheet next to the appropriate sample ID.

(10) If the lead sampler pulls a transport blank (2L of DI water filled prior to trip) from the cooler, the sampler will unscrew the lid and remove to expose the bottle contents to the atmosphere for 5 sec, reseal the bottle, fully submerge the bottle in the field water, and return the bottle to the sample cooler from which it was removed. The lead sampler should relay to sampling assistant 2 that the sample was a blank, so that it can be recorded on the data sheet next to the appropriate ID. BLANKS ARE TAKEN IN TANDEM WITH THE NEXT ACTUAL SAMPLE AND DO NOT REPLACE A SAMPLE IN THAT LOCATION. If a blank has been pulled, the boat will remain at the same location and an actual sample will be taken.

(11) Duplicate samples will be taken as part of quality control. Duplicate sample locations will be identified by RED STARS on the aerial location map. Duplicate samples will be collected the same as a regular sample; however, the lead sampler should relay to sampling assistant 2 that the sample was a duplicate, so that it can be recorded on the data sheet next to the appropriate ID. DUPLICATE SAMPLES ARE TO BE TAKEN IN TANDEM WITH THE NEXT REGULAR SAMPLE. If a duplicate sample is designated, this sample should be taken concurrently with the regular sample, side-by-side, to best replicate the regular sample collection. Sampling assistant 1 should assist the lead sampler by holding one of the sample bottles and filling that sample bottle in tandem with the lead sampler filling their own sample bottle. If a blank sample is pulled from the cooler at a designated duplicate location on the aerial map, take the duplicate sample at the NEXT DESIGNATED REGULAR SAMPLE LOCATION.

(12) Steps 1 through 10 will be repeated at each sampling location until sampling has been completed for the targeted reach.

(13) Once sampling is complete, ice will be added to the sample coolers as soon as possible. Enough ice should be added to each cooler to completely surround each sample bottle and maintain an inside temperature of 4.4°C. If at any time during transport the inside temperature of the cooler(s) rises above 4.4°C, additional ice should be added.

(14) Chain of custody (COC) forms (Exhibit 3) will be completed for every sample. All samples, including blanks, will be logged onto COC forms. The forms will be collected and signed whenever the coolers are transferred between parties.

SECTION 3

3. SAMPLE FILTERING

3.1 Purpose

In order to isolate eDNA from water samples collected in the field, particulate matter must be filtered from the sample using a vacuum filtration system. Passing each water sample through the appropriate sterile filter (1.5 micron, 5.5 cm diameter glass fiber filter) will collect particulate matter from the water sample, including sloughed cellular materials containing eDNA, on the filter paper. DNA will later be extracted from the filtered particulates and utilized in subsequent analyses.

Cautions: Wear gloves when filtering samples (a glove change is required for each sample). Be careful to avoid unintentional punctures of gloves when using forceps. Punctured gloves must be changed immediately. Be careful not to touch commonly used items in the laboratory when wearing sample gloves (i.e., writing utensils, stationary lab equipment). If in doubt, change your gloves!

3.2 Filtering Procedure

Water samples collected in the field need to be filtered within 12–16 hours after the last field sample is collected.

Equipment needed:

- Manifolds
- Glass fiber filters
- Forceps (microforceps) – at least two pair, labeled with different colors or other identifier
- Carboy (3.5 gal or larger), for wastewater generated during filtering
- Rubber tubing (1.25 in. inner diameter), double hole stopper that fits carboy opening, and glass connectors for connecting manifold to carboy and manifold to vacuum line
- Sterile conical tubes (50mL plastic) with caps and labels
- Sterile conical tubes (15mL plastic) with caps and labels
- Paper towels
- Black permanent markers
- Sterile powderless latex or nitrile gloves
- Vacuum system capable of −75 kPa vacuum
- Hypochlorous acid
- Sterile 1L graduated cylinder for measuring reagent grade water
- Reagent-grade water
- Dedicated lab equipment cleaning sink
- Waste water disposal location such as nonspecified-use sink

- Sterile bench paper
- Dedicated water bottles: one for DI water; one for hypochlorous acid solution
- Washbin for manifolds, such as a dedicated 10 qt plastic tub

3.3 Laboratory Preparation

(1) Wash hands thoroughly prior to starting. Prepare a dedicated plastic wash bottle with 10% hypochlorous acid (14-412-53) solution for wiping down lab tables and manifold surfaces prior to processing samples. Sterilize all equipment needed prior to starting. Collect all supplies needed.

(2) Rinse down each workstation with hypochlorous acid solution prior to beginning the filtration process. Cover each workstation surface with sterile bench paper. Bench paper must be switched out between samples.

(3) Put on new sterile powder-free latex or nitrile gloves. Prior to filtering a sample, each work station should have one black waterproof permanent marker (e.g., Sharpie®) for labeling sample tubes, one sterile 50mL and one sterile 15mL plastic conical tube (50mL tubes Fisher Scientific Item #14-959-49A; 15mL tubes Fisher Scientific Item #14-959-49D), sterile filter paper (1.5 micron, 5.5 cm diameter glass fiber filter paper, Type 934-AH; 55mm circles, #1827-055), one set of sterile forceps (19-027506) for placing filter paper on filter apparatus, one set of forceps for handling used filter paper, dedicated wash bottle (03-409-34) with DI water, and dedicated wash bottle with 10% bleach solution.

(4) At each workstation, connect a 3-place sterile stainless steel filter funnel manifold (Millipore 3-place stainless steel Hyrrosol Manifold, Item #XX2504735) to a large (3.5-gal Kimax recommended, Fisher Scientific Item #12-141-310) carboy bottle using rubber vacuum tubing (14-176-24), two-hole stopper (14-140P), and plastic tubing connector (509557177). Connect carboy bottle to vacuum line with second piece of rubber tubing and plastic connector. Glass connectors may be used instead if available.

3.4 Sample Preparation

(1) Put on new sterile powder-free latex or nitrile gloves prior to handling each sample.

(2) Remove first sample from transport cooler and rinse bottle thoroughly under ultrapure water to remove residual biological materials. Place prepared bottle at workstation.

(3) Label one sterile 15mL conical tube and one sterile 50mL conical tube with sample number (YYYYMMDD-SSS); indicate that the filter to be stored in the 15mL tube is the equipment control by labeling this tube with a “C”. Ensure gloves that come into contact with labeling marker are not used again for handling other samples.

3.5 Filtering the Equipment Control

(1) Put on clean powder-free latex or nitrile exam gloves prior to processing each new sample

(2) Place bottom portion of sterile magnetic filter funnel (Pall-Cat No. 4238; VWR catalog no. 28150-496) equipped with rubber stopper on manifold and open vacuum line

(3) Take designated forceps for handling clean filter paper (1.5 micron, 5.5 cm diameter glass fiber filter paper (Type 934-AH); #1827-055), remove one filter and place on bottom portion of sterile magnetic filter funnel. Once the filter paper is positioned on the magnetic filter funnel, attach the upper portion of the magnetic filter funnel (i.e., the funnel) to the bottom portion.

(4) A sterile and clean magnetic filter funnel must be used for each sample. The cleaning process is described under the Equipment and Work Area Cleaning section.

(5) Using a sterile 1L graduated cylinder, measure out 1L of DI water

(6) Once the magnetic filter funnel top is secured to the bottom portion, pour the 1L of DI water from the graduated cylinder into the magnetic filter funnel top as a control. In order to capture any potential contaminant DNA in the funnel, be sure to pour the DI water quickly so as to immerse all the internal surfaces of the magnetic filter funnel top with DI water. Once the DI water has been poured into the filter funnel top, turn the vacuum on to draw the water down quickly and filter the material as quickly as possible.

(7) Once the 1L of DI water has been filtered through the funnel, remove the filter funnel top. Take the sterile forceps designated for used filter paper and grasp the edge of the filter paper. Roll or fold the filter paper until it is of a size to fit into the 15mL conical tube labeled as a control for the appropriate sample ID.

(8) Place the control filter paper into the 15mL tube, screw on top, and place tube with control sample filter paper into a −20°C frost-free freezer. The freezer should be secured (i.e., locked) if samples are left for any period of time unattended.

3.6 Filtering the Sample

(1) Take designated forceps for handling clean filter paper (1.5 micron, 5.5 cm diameter glass fiber filter paper (Type 934-AH); #1827-055), remove one filter and place on bottom portion of sterile magnetic filter funnel. Once the filter paper is positioned on the magnetic filter funnel, attach the upper portion of the magnetic filter funnel (i.e., the funnel) to the bottom portion.

(2) Take the sample bottle and gently shake to distribute the contents within the sample evenly.

(3) Once the magnetic filter funnel top is secured to the bottom portion, turn on the vacuum and then pour approximately one-third of the sample into the magnetic filter funnel top.

(4) DI water from a wash bottle may be used to rinse any particulates attached to the sides of the magnetic filter funnel onto the filter paper. Make sure that DI in wash bottle is fresh and was replaced prior to the filtering process for the entire collection.

(5) Once the one-third portion of the sample has been filtered through the funnel, remove the filter funnel top. Take the forceps designated for used filter paper and grasp the edge of the filter paper. Roll or fold the filter paper until it is of a size to fit into the 50mL conical tube labeled for the appropriate sample ID.

(6) Repeat steps 1–4 at least two more times or until the entire sample has been filtered. All filter paper used to process the sample is placed in a single 50mL conical tube labeled for the appropriate sample ID. Place the sample filter paper into the 50mL conical tube, screw on the top, and place the tube with sample filter paper into a −20°C frost-free freezer. The freezer should be secured (i.e., locked) if samples are left for any period of time unattended.

(7) The number of filters used to process the sample is up to the discretion of personnel processing the sample. If the water sample exhibits an excessively slow filtration rate, multiple filters should be used. Also up to the discretion of the personnel processing the sample is the amount of sample water to run through a single filter. A general rule is to run one-third of a 2L sample through a single filter; however, if the sample water is extremely turbid, for example, less water should be put through a filter. At the other end of the spectrum, if the sample water is extremely clear, more than one-third of a 2L sample may be run through a single filter.

(8) On the field datasheet (Exhibit Form 2) next to the appropriate sample ID, mark the military time of filter completion and the initials of the person that processed the sample.

(9) Change gloves and sterilize the workstation between samples. Repeat steps 1–8 until all samples have been processed.

(10) When filtering, if the water collection carboy becomes full, disconnect the carboy from the vacuum and manifold and dispense water in a sink separate from the one used to clean equipment. Once emptied, reconnect the carboy to the vacuum and manifold and proceed with the filtering process. **CAUTION: Be sure to open manifold valve and turn off the vacuum air supply when disconnecting and connecting the carboy so as to prevent explosion of the glass.**

(11) If sample is accidentally spoiled during the filtering process (e.g., hypochlorous acid was used to rinse filter funnel instead of DI water, forceps from previous sample used, etc.), immediately throw away ruined samples. If portions of the sample are still viable, place in 50mL conical tube. On the outside of the sample tube, label with the amount of the viable sample (e.g., 2/3 sample). On datasheet, label with the same information (e.g., 2/3 sample) next to appropriate sample ID. Note the reason for the ruined or diminished sample (i.e., spilled bottle, acid solution used for rinsing instead of DI water, suspected cross contamination due to dirty gloves, etc.).

3.7 Preparing and Filtering a Positive Equipment Control

(1) In advance of filtering, small aliquots (0.5-1.5ml) of a dilute slurry of DNA material from Asian carp (e.g., scales, slime coat) should be maintained in a freezer. Positive control slurries should be prepared using different equipment than any used for filtering and should be prepared in a different room from that used for filtering.

(2) Following the completion of all other filtering (Sections 3.5–3.6), a DNA slurry aliquot should be dispensed into 2L of sterile water. Opening and dispensing the slurry aliquots should be done, if possible, in a room separate from the filtering lab. If that is not possible, it should occur in an area of the room not used for filtering, preferably within a sink or other area where genetic material is likely to be washed away and/or unlikely to come into contact with equipment or laboratory personnel.

(3) The 2L positive control solution should then be vigorously swirled for 1 min. Shaking should be avoided in order to minimize the chance of spreading DNA contaminants in the work area.

(4) The positive control solution should then be treated and filtered in the same way as other samples were treated and filtered (Sections 3.5–3.6).

(5) The positive control filters will be sent to ERDC for processing along with all other samples from an eDNA sampling event.

3.8 Equipment and Work Area Cleaning After Filtering Each Sample

(1) Fill a 500mL glass beaker with 10% hypochlorous acid solution. Forceps designated for used filter paper must be switched out for each sample. Used forceps will be placed in beaker with 10% hypochlorous acid solution for a minimum of 10 min for sterilization. Once sterilized, remove forceps from hypochlorous acid solution and rinse thoroughly with DI water before use.

(2) Fill at least a 10-qt plastic tub (e.g., Rubbermaid® plastic storage bin) with 10% hypochlorous acid solution. Once a sample has been processed, the filtering apparatus must be dismantled (i.e., the magnetic filter funnel should be separated into the upper and lower parts), rinsed with DI water to remove any particles and/or film, and placed in the plastic tub with the 10% hypochlorous acid solution for a minimum of 10 min for sterilization. Once sterilized, remove the two parts of the magnetic filter funnel and thoroughly rinse with DI water before use. Rinsing should continue until all residues and scent from the hypochlourous acid can no longer be detected.

(3) In between each sample, dispose of bench paper. Wipe down surface with 10% hypochlorous acid solution using wash bottle and paper towels. Cover work station with new bench paper. CHANGE GLOVES!

SECTION 4

4. SAMPLE SHIPMENT

4.1 Purpose

Samples must be shipped to the ERDC lab in Vicksburg, MS for processing of eDNA within 24 hours of sample preparation (after filtering is complete) or up to one week after filtering is complete if samples are properly stored before shipping (-20°C). The Filtering Leader is responsible for ensuring that samples are properly packed and shipped according to the procedure below.

Cautions: Wear gloves and use caution when working with dry ice.

4.2 Shipping Procedure

(1) Corrugated boxes (minimum outer dimensions 12" X 12" X 12") with Styrofoam cooler inserts will be prepared for shipment. The number of boxes to prepare depends upon the number of samples collected (e.g., a 120-sample collection will require more boxes for shipping than a 50-sample collection); however, at least two boxes will be prepared for every collection: one for the controls and one for the samples.

(2) The bottom of the coolers will be lined with dry ice pellets (approximately 1–2 inch thickness). Oven-mitt type gloves must be worn by personnel that are handling dry ice to protect hands.

(3) Remove the 15mL conical tubes with control filter paper from secure (i.e., locked) −20°C freezer and place in clean 1-gal resealable bag (e.g., Ziploc®). Multiple bags may be used if the entire sample does not fit in one bag. Seal the opening of all bags used with tamper-evident tape (e.g., Evidence Tape – NC9709516). Be sure to remove as much air as possible from all resealable bags used before sealing. Place bags of conical tubes on top of dry ice pellets in cooler. Layer approximately 1 in. of dry ice pellets on top of bag before placing another bag in the cooler. Repeat until only 2 in. of space is left at the top of cooler and fill the space with dry ice pellets. Before closing the Styrofoam cooler, record the inside temperature on the datasheet (Exhibit Form 1). Place the Styrofoam lid on top of the sample contents and seal with tamper-evident tape (e.g., Evidence Tape).

(4) Sign and place the COC form (Exhibit Form 3) in a clean 1-gal resealable bag on top of the cooler before closing the corrugated shipping box and sealing with packing tape. A copy of the COC form should be made and retained by the sender.

(5) Repeat steps 1–4 for 50mL conical tubes and additional boxes. Each box will have a separate signed COC form included to document the samples included therein.

(6) Fill out a Federal Express (FedEx) air bill shipping label with appropriate information. On the label be sure to designate FedEx Overnight Express (delivery is usually the following

day between 8 and 10 AM) as well as to identify the weight of the dry ice in the package. When ready, call FedEx (1-800-463-3339) for pickup. Be sure to tell the operator that the package contains dry ice and ask for an approximate pickup time. Be sure to record tracking numbers for all boxes being shipped.

(7) Items will be shipped to the following address:

Richard Lance
c/o Xin Guan
CEERD-EP-P
3909 Halls Ferry Road
Vicksburg, MS 39180-6199

(8) Once items have been picked up for shipment, personnel at ERDC will be contacted and notified of approximate delivery date and time. The following personnel will be contacted via telephone and email regarding shipment:

Richard Lance
Phone: 601-634-3971
Email: Richard.F.Lance@usace.army.mil

Xin Guan
Phone: 601-634-3022
Email: Xin.Guan@usace.amy.mil

(9) Upon receipt of the samples at ERDC, inside temperature of all the coolers must be taken and recorded. Samples that have remained at room temperature (approximately 20°C) for more than 24 hours will be discarded, and the sample names and reason(s) for discarding will be noted in the laboratory log.

(10) Personnel receiving the shipment must immediately sign the COC form.

SECTION 5

5. DNA ASSAYS

5.1 General Quality Assurance and Chain-of-Custody Considerations

(1) Any change to described DNA handling, storage, or processing procedures must not result in reduction of eDNA sensitivity relative to current values and must be cleared with the Project Leader. The current protocol produces the following results at different concentrations of total genomic DNA [1]in sterile water:

Species	Total Genomic DNA Dilution (ng DNA/µl water)	% PCR Success (positive band)
Bighead Carp	0.0001	100
Silver Carp	0.001	100
	0.0001	25-100

(2) Each stage of eDNA genetic processing procedures (eDNA sample extraction, PCR setup, and post-PCR processes) should be performed in a separate room in order to minimize the risk of sample cross-contamination.

(3) Every effort should be made to ensure that equipment, work areas, and solutions are free from DNA contamination. As a minimum, all surfaces should be wiped clean with 10% hypochlorous acid solution before and after use.

(4) All centrifuge tubes and glassware must be autoclaved at 121 ˚C for 20 min before being used.

(5) Good housekeeping policy should be practiced at all times. Reagents that have passed expiration dates should not be used, nor should any reagents that have been kept at incorrect storage temperatures for a significant length of time. All reagents, reaction tubes, etc., must be clearly labeled. Records of batch numbers of all reagents used in individual assays should be made whenever reagents are signed out from the designated freezer. The temperatures of cold storage units must be monitored twice a day, once in the morning and once at end of day, using the form given in Exhibit 5.

(6) Positive and/or negative reactions should be used to test all new batches of critical components prior to or concurrent with their application to eDNA samples.

(7) Standard sterile techniques should be used in the DNA laboratory to prevent the unintended transfer of DNA between surfaces, and to prevent cross-contamination

[1] Total genomic DNA refers to DNA extracted directly from Asian carp tissues.

between samples. Contamination can adversely affect the outcome of a case; therefore, it is essential that the laboratory have procedures in place to limit, recognize, and address contamination.

(8) Gloves (e.g., sterile nitrile or latex) must be worn throughout sample processing. At a minimum, gloves should be changed at the completion of each step of the process. If gloves become contaminated or if contamination is suspected, discard them and replace them with new ones. For example, gloves should not be worn when using or handling keyboards, notebooks, pens, telephones, etc. and must be replaced immediately before recommencing bench work.

(9) Centrifuge tubes before opening the reagents. Uncap and close tubes carefully to prevent aerosol contamination.

(10) Ensure that centrifuges are always balanced when centrifuging samples.

(11) Ensure that all equipment, including paper, pens, and lab coats, are dedicated for use only in that particular laboratory (e.g., laboratory coat for each stage of procedure rooms. Workbooks that have been in contaminated areas shall not be taken into clean PCR areas. A Project Lab Book should be kept in a room separate from the DNA Extraction Room and DNA PCR Room. Each room (Extraction, PCR, Post-PCR) should have note-taking materials (e.g., loose-leaf paper, networked tablet PCs) that can be transported or viewed for consolidation in the Project Lab Book. Other solutions for preventing contamination of sensitive areas via lab notes may be used following approval by the eDNA Processing Leader. Any changes should be incorporated into a revised QAPP. Laboratory notes/notebooks should:

- Be written or printed on tamper-proof paper (e.g., does not exactly photocopy).
- Have lab book identification, with consecutive numbering, dates, and signatures (of the note-taker) on each page.
- Be made using permanent ink. Special pens may be required for certain paper types.
- Have any changes to notebooks be dated and initialed by the person who made the change. Any incorrect information should have a single line drawn through it and not be completely obscured.
- Contain all data images (e.g., gel photographs, denaturing curves, DNA sequence electropherograms). Images should be permanently affixed to the notebook and signed across both the edge of the insert and the page.
- Be kept in a locked drawer or cabinet with restricted access when not in use.

(12) A log of all batches of critical components should be kept. This log should include material safety data sheets (MSDS) and product information sheets. Dates of receipt, opening, testing, and disposal for each component should be recorded in the log.

5.2 Quality Control for Sample Custodian Procedure and Storage

(1) A COC Log Book should be kept for all samples. Tamper-proof paper should be used. The log book should be kept in a locked drawer or cabinet when not in use.

(2) Separate freezers should be designated for storage of (a) filter samples, (b) DNA extracts, (c) PCR and sequencing product, and (d) PCR, cloning, and sequencing kit components. A dedicated refrigerator should also be maintained for any PCR, gel electrophoresis, cloning, and sequencing kit components that require 4 ˚C storage.

(3) Maps or other designations of the location of samples within freezers should be maintained.

(4) All items in freezers should have indelible ink identifications.

(5) All freezers should have non-universal locks or marine brackets attached that can be used with keyed locks.

(6) All samples removed from freezers should be signed for on freezer log in addition to sample log (see Exhibits 2 and 4). All reaction components should be signed for.

5.3 Physical Separation of Pre-PCR and Post-PCR Assay Stages

5.3.1 The eDNA Extraction Room

- Extraction of DNA must be performed where PCR products and stocks of cloned material are not handled.
- A PCR hood with a built-in ultraviolet (UV) light and HEPA filter may be used to further isolate DNA extraction kit solutions and elutes from ambient DNA.
- A complete separate set of necessary laboratory equipment, consumables, and laboratory coats should be dedicated for use in DNA extraction.

5.3.2 Pre-PCR Room

- To prevent carry-over of amplified DNA sequences, PCR reactions should be set up in a separate room from that used for post-PCR manipulations.
- A completely separate set of necessary laboratory equipment, consumables, and laboratory coats should be dedicated for the specific use of pre-PCR manipulations.
- Reagents and supplies should be taken directly from clean storage into the PCR area and should never be taken or shared with areas in which post-PCR analyses are being performed. Equipment such as pipettes should never be taken to the containments area after use with amplified material.
- A sign-in log system should be implemented for use of the thermal cyclers (PCR machines), including the run name, plate orientation, and the number of thermal cycler heads.

5.3.3 Product Analysis Room

This is the post-PCR Room where post-PCR manipulations are performed, including agarose gel electrophoresis of products.

- This room is a contaminated area; therefore, **<u>no</u>** reagents, equipment, laboratory coats, etc. from this room should be used in any of the other PCR areas.
- A biological or PCR-type hood may be used for setting up cloning or sequencing reactions.

5.4 Receipt of Filters

5.4.1 Source

Water samples have been taken in the field according to Section 2 and filtered according to Section 3. Filters have been shipped to ERDC and personnel assigned to the ERDC eDNA Team have either received or picked-up shipping boxes from the ERDC Receiving Office.

(1) Upon receipt of samples from the eDNA sample filtering team, the shipped box(es) should be opened and the temperature inside each box recorded. The general condition of the box(es) should also be recorded.

(2) To measure temperature upon opening the box, either (a) place a glass thermometer inside the styrofoam container, replace lid, and leave the thermometer in place for at least 2 min before removing it and immediately recording temperature or (b) immediately aim an infrared laser thermometer at the samples and press the MEASURE button to record the temperature inside the cooler.

(3) Place samples in filter sample storage freezer (–20°C) and log samples on freezer sheet (see Exhibit 4).

(4) Sign and date the COC forms that accompanied the samples. Place them in a designated file; a copy should also be provided to LRC. If the forms were inside sealed bags, slit the bag to remove the COC forms, and then place unbreakable tape across entire slit. Sign and date tape. Note any condition issues (broken tape or seals, damaged containers or bottles, etc.) with the samples on the COC forms. Note any samples that must be discarded due to condition issues and the reason for discard.

(5) Enter sample data into new sample log book, including noting any samples that are being discarded and that should not be analyzed, and create new ERDC COC form for samples. Note any observations about samples such as condition issues.

(6) Alert Sampling and Filtering Leader that samples have been received. Use e-mail addresses with return/receipt requested or directly contact via telephone; team contacts are listed in Appendix A. This reporting must be done within 1 hour of receipt.

5.5 DNA Extraction from Filters

5.5.1 Source

Filters from eDNA sampling have been received by ERDC eDNA Team and logged. Samples should be in designated −20°C freezer.

5.5.2 DNA Extraction Quality Assurance and Chain of Custody

At this stage, a critical component of quality control should be to correctly label all sample extraction processing tubes so that there is no question about the origin of samples.

(1) Bench areas in DNA extraction laboratory and PCR-type hood (if used) should be wiped before and after use with 10% hypochlorous acid. PCR hood should be sterilized using a built-in UV radiation bath capability following use. Validated, commercially available sterilization reagents, such as LookOut® DNA Erase®, may be preferred.

(2) After an item or surface is cleaned with hypochlorous acid, it must be rinsed with purified water or alcohol to prevent the build-up of sodium hypochlorite crystals. Instruments or equipment cleaned with hypochlorous acid must be rinsed to avoid corrosion.

(3) It is common practice for moisture barrier paper towels to be placed on the bench top while processing samples to act as a barrier. The paper barriers must be changed and the bench top cleaned between sample batches.

(4) Centrifuges, thermal cycler, tube racks, pipettes, and any other equipment used for the extraction process should be cleaned before and after each use.

(5) Instruments such as forceps and scissors should be cleaned just prior to use. Sterile disposable equipment should be opened just prior to sample processing and discarded after one use.

(6) When using pipettors, use filtered tips and never allow the liquid in a pipette tip to rise up to the cotton barrier.

- Do not rest the pipette on a dirty surface.
- Avoid cross-contamination by changing pipette tips after each use.
- Watch that the tip -- and only the tip -- is allowed to go into a bottle of reagent, never the pipette itself.

(7) Record all solution batch numbers used for reactions in lab notes.

(8) Vortex tubes before opening.

(9) No deviations to the DNA extraction protocol are allowed without written approval by the Project Leader. Any errors in processing should be noted in the laboratory log. Samples affected by errors in the extraction protocol should be clearly identified.

Cautions: As with all components of eDNA processing, quality control and sterilization procedures must be carefully followed in order to avoid contamination of downstream procedures.

5.5.3 Procedure

(1) Remove samples from freezer; note on freezer and sample logs (see Exhibits 2 and 4), as well as COC form.

(2) Keep samples on ice.

(3) Positive and negative extraction controls should be added to each eDNA extraction procedure batch.

- Before proceeding with extraction, a positive control filter is prepared by pipetting 25 µl of a mixed slurry of homogenized silver and bighead carp tissue directly onto a sterile filter paper (1.5 micron, 5.5 cm diameter glass fiber filter paper, Type 934-AH; #1827-055). Alternative species (e.g. – sturgeon) may be used as the positive control to reduce risk of sample contamination from carp tissue. Alternative species must have PCR primers that (1) do not cross-react with carp DNA and (2) can be run on the same themocycler settings as carp samples
- Additionally, an extraction negative control sample should be prepared by UV-sterilizing a previously unused filter paper (1.5 micron, 5.5 cm diameter glass fiber filter paper, Type 934-AH; #1827-055) and placing it in a new sterile 15mL tube. A batch of extraction blanks can be prepared in advance and kept frozen at −20°C
- For every 23 samples processed, conduct DNA extraction (below) on one frozen, sterile extraction negative control filter and one prepared positive control filter.

(4) For all samples and cooler, equipment, and extraction controls, follow the DNA extraction protocol detailed below:

This DNA extraction utilizes the PowerWater DNA Isolation Kit (MoBio Laboratories, MoBio Inc.) and the protocol is adapted from the manufacturer's protocol (http://www.mobio.com/images/custom/file/14900.pdf).

(a) Place Solution PW1 in a 55°C water bath for 5–10 min to dissolve any precipitates that have formed at room temperature. Remove Solution PW1 from the water bath immediately prior to use.

(b) Remove the appropriate filter sample from −20°C freezer and transfer the filter(s) to a labeled 5mL PowerWater Bead Tube. If DNA will be extracted from multiple filter samples, continue to remove each filter sample from −20°C freezer immediately prior to filter transfer. Each PowerWater Bead Tube will hold up to three filters per sample. If any filtered water samples required four pieces of filter paper, split the filters into two PowerWater Bead Tubes.

Note: Change gloves between the transfer of each filter sample to avoid cross-contamination of samples.

(c) Add 1mL of Solution PW1 to the PowerWater Bead Tube and secure the cap tightly. Mount the tube on a vortex adaptor (MoBio Inc.) and vortex on high for 5–10 min, or until the contents of the bead tube appear liquefied. Times can vary depending on the number of filter papers being extracted. No more than four pieces can be extracted per bead tube. If more than four are used for one sample, use multiple bead tubes and combine the supernatants from these tubes in step 9 (below).

(d) Centrifuge the tubes at 4,000 x *g* for 1 min at room temperature. Ensure centrifuge is balanced before centrifuging. Transfer supernatant using a 1mL pipette to a labeled 2mL collection tube.

(e) Centrifuge tubes at 13,000 x *g* for 1 min and carefully decant supernatant into a labeled 2mL collection tube.

(f) Add 200µL of Solution PW2, vortex briefly, and incubate at 4°C for 5 min.

(g) Centrifuge the tubes at 13,000 x *g* for 1 min and carefully decant supernatant into a labeled 2mL Collection Tube.

(h) Add 650µL of Solution PW3 and vortex briefly. Load 650µL of supernatant onto a spin filter and centrifuge at 13,000 x *g* for 1 min. Discard the flow through and repeat until all the supernatant has been loaded onto the spin filter.

(i) Place the spin filter basket into a labeled 2mL collection tube and add 650µL of Solution PW4.

(j) Centrifuge the tubes at 13,000 x *g* for 1 min and discard flow through.

(k) Add 650µL of Solution PW5 and centrifuge at 13,000 x *g* for 1 min. Discard flow through and centrifuge again at 13,000 x *g* for 2 min.

(l) Place the spin filter basket into a 2mL collection tube labeled with the sample identification number, the date the water sample was collected in the field, and the volume of water collected.

(m) Add 100µL of sterile water (autoclaved, de-ionized) to the center of the white filter membrane and centrifuge at 13,000 x *g* for 1 min.

(n) Discard the spin filter basket and store the eluted DNA samples at −20°C.

(5) DNA extraction elutes should be placed into a designated freezer or, if immediately used for PCR, kept on ice. Make note of sample addition to freezer log if necessary (see Exhibit 4). Note completion of extraction on sample log.

5.6 PCR Amplification of eDNA Samples

5.6.1 Purpose

In order to determine if the DNA of a specific species is present in the filtered water samples taken in the field, the total DNA extracted from the filtered samples must be amplified using species-specific primers.

5.6.2 Source

Filters from eDNA sampling have been received by ERDC eDNA Team and DNA has been extracted. DNA elutes from samples should either be located in designated −20°C freezer or carried on ice from DNA extraction room to PCR room.

5.6.3 PCR Quality Assurance and Chain of Custody

This stage of DNA processing is particularly susceptible to contamination and, subsequently, inaccurate results. Carefully follow quality control and COC steps listed below:

(1) PCR-type hood bench should be wiped before and after use with 10% hypochlorous acid. PCR hood should be sterilized using a built-in UV radiation bath capability following use. Validated commercially available sterilization reagent such as LookOut® DNA Erase® may be preferred.

(2) After an item or surface is cleaned with hypochlorous acid, it must be rinsed with purified water or alcohol to prevent the build-up of sodium hypochlorite crystals. Instruments or equipment cleaned with hypochlorous acid should be rinsed to avoid corrosion.

(3) Centrifuges, thermal cycler, tube racks, pipettes, and any other equipment used for PCR Amplification should be cleaned before and after each use.

(4) Use UV radiation to cross-link 96-well PCR plates and the exteriors of manual pipettors, as well as autoclaved, filtered, or commercially sterile water prior to use for setting up PCR reactions.

(5) Aerosol-resistant pipette tips should be used. Place the sterile tip on the pipette immediately prior to use. If the pipette is set down with the tip on, discard the tip. A new pipette tip must be used for the addition of each reagent to a sample tube.

(6) Autoclave sample tubes.

(7) Only one microcentrifuge tube should be open at a time. Close each tube immediately after labeling and after the addition of sample or reagents to prevent cross-contamination.

(8) Use a tube opener, clean Kimwipe®, or other suitable barrier, rather than gloved fingers, to open microcentrifuge tubes.

(9) Record all solution batch numbers used for reactions in lab notes.

(10) PCR reagents should be aliquoted (a portion of the original stock) to avoid excessive freeze-thawing and to protect stock reagents if contamination occurs.

(11) Centrifuge tubes before opening the reagents. Uncap and close tubes carefully to prevent aerosol contamination.

(12) Any revisions to the DNA amplification protocol must be approved by the Project Lead and documented in writing.

Cautions: Wear gloves throughout the DNA amplification and gel electrophoresis procedures. Ethidium bromide, used in DNA gel electrophoresis to visualize DNA, is a known mutagen that affects biological processes.

5.6.4 Procedure

(1) If DNA samples (extraction elutes) are removed from freezer, note on freezer log (see Exhibit 4). Also note on sample log and ERDC CoC form (Exhibits 2 and 3).

(2) Use preprinted 96-well plate map to determine which samples will be pipetted into which wells. Clearly mark plate identification on top right corner of plate. Write plate identification information (number and corresponding samples) in lab notes. Mark left side (technician's left) of the plate with L, right side (technician's right) with R, top (T; 90° clockwise from L side) with T, and bottom (B; 270° clockwise from L side) with B using indelible marker.

(3) Plate identification can be as simple as date, order of processing, and target species. For example, SC011210A might refer to the first (A, B, C...) silver carp PCR assay conducted on 1 December 2010.

(4) Make sure sample map for each plate is attached to lab notes.

(5) Keep samples on ice or in cold block.[2]

(6) Follow DNA amplification protocol detailed below.

Primers specific to either *Hypophthalmichthys molitrix* (silver carp) or *H. nobilis* (bighead carp) are used to screen eDNA samples and amplify unique DNA sequences in each species potentially present in the eDNA samples by PCR. The PCR programs used to amplify the extracted DNA are specific to the primer set used. The PCR protocol has been optimized to utilize specialized *Taq* polymerase and buffer from Platinum® *Taq* DNA polymerase (Invitrogen Corporation, Carlsbad, CA) in the eDNA screening. Eight reactions are set up for

[2] Several different companies sell a variety of cold blocks for 96-well plates.

each sample, in addition to negative (DNA blank) and positive (DNA extracted from tissue[3]) controls for each master mix. The PCR reactions are prepared as follows:

(1) Wipe lab bench area with 10% hypochlorous acid, 75% Ethanol, or commercial DNA sterilization wipes. Also wipe down work area with PCR hood. Use built-in UV lamps to radiate bath hood area for 10 min prior to PCR set-up.

(2) UV radiate cross-link water aliquots, manual pipettors, and 96-well plates. This can be done concurrently with Step 1. Electronic pipettors should be wiped down with one of the solutions or wipes listed in Step 1.

(3) Obtain all PCR master mix reagents (using only those that have not expired and that have been tested and found viable). Keep reagents on ice or in cold block:
- 10X PCR buffer
- 10mM equally mixed dNTP solution (2.5 mM per nucleotide)
- 25mM Mg2+ solution
- Species-appropriate forward primer
- Species-appropriate reverse primer
- Taq DNA polymerase
- Sterile DI water (commercially sterile or Millipore filtered, autoclaved, and UV cross-linked)

(4) Also set aside enough positive control DNA for each species for three PCR reactions each.

(5) Record in lab notebook the lot number of all reagents used.

(6) Prepare PCR master mixes. The master mix volume can be adjusted according to the number of samples to be processed. In order to make sure that master mix does not run out prior to supplying all the desired reactions (this may occur as a result of minor errors or variations in pipetting volumes), it is generally helpful to make more than enough master mix than is needed for the desired number of reactions. For example, make enough master mix for 100 reactions when actually preparing for 96 reactions.

(a) Each Initial PCR 1X reaction should contain:
- 2.5 µL 10X PCR buffer
- 0.5 µL dNTP (= 10 mM mixed dNTP)
- 0.75 µL Mg2+ solution (25mM)
- 0.5 µL forward primer (= 10mM working dilution)
- 0.5 µL reverse primer (= 10mM working dilution)
- 0.25 µL Platinum® *Taq* polymerase (= 1.25 U)
- 19.0 µL sterile water.

[3] Extract from tissue using commercial DNA extraction kit (e.g., Qiagen DNeasy Blood & Tissue Kit), and manufacturer protocol. Run test PCR before relying on any DNA extract for eDNA assay positive controls.

(7) Place a 96-well PCR plate into a prepared cold block, positioned from left to right. Add 24 µL of the master mix into each well on the 96-well PCR plate (except the two empty wells in the 12th column).

(8) Pipette 1 µL of each sample to be screened into each well of a column, changing the pipette tip between each sample. Each column of eight wells should be filled with the same sample (i.e., eight replicates per sample to be tested). The first 11 columns of the PCR plate can test 11 different samples for one target species. Into the 12th column, pipette 1 µL of sterile water into the bottom three wells (F, G, H) and pipette 1µL of each of the target species positive control DNA into each of the top three wells (A, B, C). Leave the intervening two wells (D, E) empty.

(9) Place the positive control DNA back in to the appropriate −20°C freezer and change gloves immediately in order to reduce risk of contamination.

(10) Place PCR film over the PCR plate and press firmly to ensure the edges of all wells are sealed. Remove plate from ice block and gently tap a few times on the lab bench to ensure thorough mixing of each reaction.

(11) Place the 96-well PCR plate in the thermal cycler, close and secure lid, and select the appropriate PCR thermal program (thermal cycle programs for silver carp and bighead carp utilize different annealing temperatures). The thermal programs both consist of:

- Initial denaturation at 94°C for 10 min

Followed by 45 cycles of:

- 94°C for 1 min,
- 50°C for silver carp program or 52°C for bighead carp for 1 min
- 72°C for 1.5 min.

Followed by:

- final extension at 72°C for 7 min
- 4°C hold temperature until plate removed from thermal cycler.

(12) Record the plate ID, thermal cycler unit or head, plate orientation, and run times for the PCR plate in the PCR log (Exhibit 6).

(13) Place PCR plates and product in designated –20°C freezer for long-term storage, in designated 4°C refrigerator for mid-term storage (1–6 hours) or in ice or in cold block for short-term (less than 60 min) storage.

5.7 Gel Electrophoresis of eDNA PCR Assays

5.7.1 Purpose

Once amplified, the DNA samples should then be subjected to gel electrophoresis in order to visualize the amplified DNA. This method is useful in determining the presence of DNA from any given species in different aquatic environments.

5.7.2 Source

PCR product following amplification can be taken either from cold storage (see #11 above) or directly from the thermal cycler.

5.7.3 Gel Electrophoresis Assurance and Chain of Custody

This stage of DNA processing is particularly susceptible to pipetting error. It is also highly susceptible to mislabeling and, consequently, confounding of sample results.

(1) Draw or otherwise produce a map of which sample will be electrophoresed on which gel and in which lane of the select gel.

(2) Carefully pipette samples so as to avoid:

- Injecting samples to incorrect wells.
- Piercing the bottom of sample wells and losing PCR product.
- Spill over from adjacent wells.

(3) Record all solution batch numbers or name/date identification for stock solutions. Record precast gel batch identification if appropriate.

(4) Centrifuge plates, strip tubes, etc. before removing film or caps in order to prevent aerosol cross-contamination.

(5) Any revisions to the DNA amplification protocol must be approved by the Project Lead (and documented) and incorporated into a revised QAPP.

5.7.4 Option A Procedure

(1) Prepare 1% agarose gels with SB (sodium hydroxide and boric acid) buffer and ethidium bromide stain (5µg/mL of gel) and allow the gel to polymerize for a minimum of 25–30 min prior to loading samples. Gels can be prepared at any time prior to PCR or immediately after PCR.

(2) To prepare PCR samples for gel electrophoresis, transfer 10µl from each well of the 96-well plate to new wells in an identically labeled 96-well plate. Add 2µl of 6X loading dye to each well with PCR product.

(3) Place the 1% agarose gel in the electrophoresis chamber that contains SB Buffer and remove the gel combs. In the first well of each row on the 1% agarose gel, load 100bp DNA adder/loading dye mix. Next, load 10µL of each sample mixture (i.e., each PCR reaction and loading dye), positive controls, and negative controls into the remaining wells. An aliquot (~ 500 ng) of an appropriate DNA ladder (distinct bands between 0-500 bp) is loaded at one end of each row and a positive control PCR product at the other end. Run electrophoresis at ~100V for ~45 min depending on migration times through the gel. Times and voltages required to run each gel are approximate.

(4) After documentation (5.8), dispose of gel in appropriate ethidium bromide waste containers.

5.7.5 Option B Procedure

Pre-cast 2% E-Gel® 48 gels (Invitrogen) are used with the E-Base™ system (Invitrogen).

(1) Preparing Samples (based on manufacturer recommendations)

- Load 20−100 ng DNA.
- Prepare DNA samples in a total sample volume of 15 with deionized water or 6X loading buffer.

(2) Selecting Program on E-Base™

- Connect the Daughter E-Base™ to a Mother E-Base™ or another Daughter E-Base™ if running multiple gels.
- Select program EG by pressing and releasing the pwr/prg (power/program) button on the base.

(3) Loading E-Gel® 48 Gels

- Load each gel within 30 min of removing gel from the pouch and run within 15 min of loading.
- Remove gel from the pouch. Remove comb from the gel.
- Slide gel into the two electrode connections on the Mother E-Base™ or Daughter E-Base™. If gel is properly inserted, a fan in the base begins to run, a red light illuminates, and digital display shows 20 min.
- Load 15µL prepared DNA sample into each well of an E-Gel® 48 gel. Keep all sample volumes uniform. Load with a multichannel pipettor.
- Load appropriate DNA ladder (distinct bands between 0-500 bp) into one marker well and positive control PCR product into the other marker well. Ensure the marker salt concentration is similar to that of adjacent samples (2% gel uses 100 bp DNA Ladder).
- Load 15µL of sample buffer containing the same salt concentration as the sample into any empty wells.

(4) Run Conditions

- To begin electrophoresis, press and release the pwr/prg button on the Mother E-Base™ and Daughter E-Base™. The red light changes to green.
- At the end of the run (signaled with a flashing red light and rapid beeping), press and release the pwr/prg button on the base to stop the beeping and flashing red light.
- Remove gel cassette from the base and analyze results.

5.8 eDNA Gel Documentation and Storage

5.8.1 Purpose

Once eDNA gels have been visualized, the results must be documented, interpreted (i.e., scored), and recorded. In some cases, very light bands may be visible, making scoring difficult. Documentation and storage are critical for later quality control review.

5.8.2 Source

Following electrophoresis, agarose gels should be immediately documented.

5.8.3 Gel Documentation and Storage Assurance and Chain of Custody

Because of the difficult nature of scoring some results, careful records must be kept of all gels and results. These results must be maintained so as to minimize the risk of tampering or data loss.

(1) Gel image quality should be assessed at the time images are obtained. Images should exhibit all bands on gels as clearly as possible. All gel digital image files should be saved and should be archived at the end of each working day. All gel image data are referenced to the samples batch receiving date to make sure the consistency of the sample custody.

(2) Gel score data should be entered and stored in the appropriate database in an Excel file and in the spreadsheet manually in a designated binder. The binder should be stored in a locked drawer.

(3) All reports should reviewed by the eDNA Processing Leader before being reported.

(4) A paper copy of the report should be held in the files for 5 years.

(5) Electronic copies of all reports should be held for 5 years or longer, as space permits.

(6) Any substantive revisions to the DNA amplification protocol must be approved by the Project eDNA Processing Leader and approved by the Project Leader. Any such changes must be incorporated into a revised QAPP.

5.8.4 Procedure

(1) After electrophoresis is complete, remove casting tray with gel from the electrophoresis chamber and place the gel onto the gel scanner (BioRad Molecular imager FX), select DNA ethidium bromide stain gel, set up scanning area, and then select 100 micrometer to start scanning the gel.

(2) Alternatively, place the gel on a UV transilluminator equipped with a digital camera, such as the Alpha red Imager (Cell Biosciences, Inc.), and capture a digital photograph of the gel.

(3) After the gel scanning is done, properly label file name and save the file on the hard drive immediately.

(4) Print out a picture of the gel image and insert into lab book. A copy should be kept with the Project Lab Book.

5.9 Gel Interpretation

5.9.1 Purpose

Once a gel is visualized, the quality of the results and presence of potential positive bands must be assessed in order to determine which samples need to be further assayed.

5.9.2 Source

Immediately following the cessation of electrophoresis, the agarose gel containing the eDNA PCR products should have been visualized on either the UV-based imager or the laser-based imager. In both cases, the gel image should be captured (saved to hard disk) immediately.

5.9.3 Gel Interpretation Quality Assurance and Chain of Custody

(1) The three positive controls should have bright bands at the appropriate migration distance (number of base pairs), indicating a positive reaction.

(2) No bands at the targeted sizes (~200bp silver, ~300bp bighead) should be observed in the negative controls.

(3) If any of the initial PCR reactions are positive (i.e., a visible band at the appropriate migration distance), the initial sample is designated a “presumptive positive”.

(4) Record the number of presumptive positive reactions for each sample both in the gel electrophoresis notebook and the personal laboratory notebook of the researcher.

(5) Presumptive positive results will initiate a series of results confirmation mechanisms (see below). These mechanisms include screening the transport and equipment controls, and DNA sequencing.

5.10 Confirmation of Positive Results

5.10.1 Purpose

These confirmation mechanisms are initiated if a sample returns as a positive for the PCR test (any number of the eight reactions, e.g., one of eight up to eight of the eight PCR reactions).

5.10.2 Source

Positive results for Asian carp eDNA require that those positive samples be further assayed. The original DNA elutes from samples should be located in designated −20°C freezer.

5.10.3 Gel Interpretation Assurance and Chain of Custody

Positive assays (PCR reactions) are validated through DNA sequencing and testing of additional control samples.

Any revisions to the DNA QA/QC amplification protocol must be approved by the eDNA Processing Leader and approved by the assigned LRD senior executive. Any such changes should be incorporated into a revised QAPP.

5.10.4 Procedure

(1) Conduct PCR assays of the paired equipment control for each presumptive positive. DNA extraction, amplification, documentation, and interpretation following protocols detailed above (Sections 5.6–5.9).

(2) Ensure that the transport blanks (see 2.2.2 (6) and 2.3.2 (10)) have been tested for that sample group (i.e., from the same cooler in which the presumptive positive sample was transported).

(3) If the equipment control and transport blanks test negative, the sample is designated a "confirmed positive."

(4) All positive samples and for samples testing positive from ecologically sensitive areas (to be determined by Sampling Lead), bidirectional sequencing confirmation is performed. This is done by using a commercially available gel extraction kit (e.g., Qiagen Qiaquick Gel Extraction kit) per the manufacturer's recommendations on the positive PCR reactions, or E-Gel® CloneWell Agarose Gels (Invitrogen) per manufacturer's recommendation.

(5) The following sequencing reaction can be done either by cloning (TA TOPO cloning kit used for sequencing per manufacturer's recommendation) then sequencing, or by a direct Sanger sequencing method (ABI BigDye® Terminator v3.1 Cycle Sequencing Kit) modified by ERDC. BigDye Terminator Reaction Master Mix for 1X reaction:

- 1µL BigDye terminator mix,
- 4µL 5X reaction buffer,
- 0.8µL either SC/BH forward primer, and
- 10.2µL of water.

Add 16µL of master mix to the 4µL of purified DNA. Total reaction size is 20µL.
The positive control reaction of sequencing was done per manufacturer's recommendation.

For each pGEM control PCR master mix:

- 4uL BigDye terminator mix,
- 4µL 5X reaction buffer,
- 2µL m13 primer,
- µL water, and

- 2µL pGEM.

Add 20µL to each control well.

Place the PCR plate on the thermal cycler and begin temperature cycling protocol. Program the thermal cycler as follows: 25 cycles of [96°C for 10 sec, 50°C for 10 sec, 60°C for 4 min], then ramp to 4°C.

(6) To clean the BigDye reaction:

- Spin plate after removing from thermal cycler (just to make sure that everything is at the bottom of the well).
- Add 5µL of 125mM EDTA to each well.
- Add 60µL of 100% EtOH.
- Seal the plate and mix by inverting four times.
- Incubate at room temperature (RT) for 15 min.
- Spin plate at 3000xg for 30 min (at 4°C) or at 2000 x g for 45 min. Proceed to next step **immediately**.
- Invert the plate and spin at 185 x g for 1 min (time from when rotor starts).
- Add 60µL of 60% EtOH.
- Spin at 1650 x g for 15 min at 4°C.
- Invert plate and spin at 185 x g for 1 min.
- Resuspend samples in 20µL Hi-Dye.

Samples in Hi-Dye can be stored at 4°C overnight, but may not be left any longer than 12 hours.

(7) To sequence:

- Denature samples for 5 min at 95°C
- Place immediately on ice
- Load into sequencer plate and onto sequencer.

Resulting sequencing reactions that are successful are screened in GenBank (http://www.ncbi.nlm.nih.gov/blast) using the BLAST (Basic Local Alignment Search Tool) algorithm. If the resulting sequence is a positive match to the targeted species of Asian carp, the sample is designated a "**confirmed positive – sequenced**".

References: User Manual, "BigDye® Terminator v3.1 Cycle Sequencing Kit"
http://www.ibt.lt/sc/files/BDTv3.1_Protocol_04337035.pdf
"TOPO TA Cloning® Kit for Sequencing"
http://tools.invitrogen.com/content/sfs/manuals/topotaseq_man.pdf
"QIAquick Gel Purification Kit"
http://molecool.wustl.edu/krolllab/Kroll_Lab_Protocols/Molecular%20Biology%20protocols/Cloning%20protocols%20folder/Gel%20extraction-Qiagen.pdf

5.11 Communication of eDNA Assay Results from ERDC to USACE

5.11.1 Purpose

To convey to LRD designated personnel the progress and results of eDNA assays.

5.11.2 Source

ERDC keeps a record of each samples progress through the EDNA assay procedure. These records are summarized for each batch for reporting to USACE.

5.11.3 Quality Control

The ERDC eDNA Team designates one of its personnel to be the Progress & Results Reporter (PRR). The PRR should provide updates and reports to the LRD designated Point of Contact for eDNA results. At this time, Matt Carr is the ERDC eDNA Team PRR designee. Kelly Baerwaldt is the LRD POC designee. Any permanent or temporary changes to either position should be communicated immediately to the eDNA Processing Leader and the assigned LRD senior executive. Any permanent changes should be incorporated into a revised QAPP.

Any revisions to the reporting procedures must be approved by the eDNA Processing Leader and approved by the assigned LRD senior executive. Any such changes should be incorporated into a revised QAPP.

5.11.4 Procedure

The ERDC PRR should, on every Friday during the period over which the ERDC eDNA Team is processing samples, provide updates on all sample batches to the LRD POC no later than 1200 CST/CDT. Reports should be organized by batch and should consist of spreadsheets showing the stage of processing for each sample. Additionally, following approval by the eDNA Processing Leader, the ERDC PRR should convey final results for each batch (= all samples confirmed as positive or negative for AC eDNA) within 18 hours of completion of processing for the last sample within a batch.

The LRD POC may request updates from the PRR at any point. The ERDC PRR is expected to respond as soon as possible during normal working hours.

Table 5-1. List of critical components for eDNA Processing, and component information for ERDC. No vendor is listed for generally available supplies.

**Equipment**
Three sets of manual pipettes ranging from 0.1µl to 1000µl<br>Electronic 8 or 12 channel multi-dispense pipettes ranging from 0.5µl to 125µl<br>ERDC: Finpipettes models # (0.1-25 µl), . . .
Programmable thermal cyclers equipped with four 96-well plate heads<br>• Thermal cycler should be capable of self-test upon instrument startup.<br>• All thermal cyclers and heads should be equipped with the heated lid function.
Centrifuge equipped with programmable speeds and time.<br>• Interchangeable Rotors capable of holding 96-well plates and tubes of various sizes (5mL, 1.5mL, and 2mL).
Electrophoresis Chambers including gel casts and gel combs
Sterile Hoods equipped with UV light and Hepa filters
Autoclave
Locking Refrigerator monitored at 4˚C equipped with a temperature sensitive alarm
Locking Freezer monitored at −20˚C equipped with a temperature sensitive alarm
Locking Freezer monitored at −80˚C equipped with a temperature sensitive alarm
Sequencer
Gel Image Scanner
UV Stratalinker
**Reagents**
LookOut DNA Erase (Sigma Aldrich Cat# L8917 refill cat # L9042)
Power Water DNA Isolation Kit (Mo Bio Laboratories Cat # 14900-100-NF)
QIAquick Gel Extraction Kit (Qiagen Cat# 28706)
Taq DNA Polymerase
Big Dye Terminator Sequencing Kit (Applied Biosystem Cat # 4337455)
Nuclease Free Water (Ambion Cat # AM9932) (Autoclaved before use.)
pGEM-3Z(+) Vector 20 g (Promega Cat # P2271)
Ethanol (Sigma Aldrich Cat # E7023)
dNTP
MgCl2
10X buffer
Primers (forward and reverse)
20X SB buffer
Cloning Reagents
Sequencing Reagents
Agarose
Hypochlorous acid Solution (10%)
Ethidium Bromide Gel Stain
100 bp DNA ladder
Gel Loading Dye
**Disposable Supplies**
Sterile Pipette Tips: various sizes and types to fit manual and electronic pipettes.
Sterile Nuclease Free conical tubes: 1.5 mL, 2.0 mL, 5.0 mL

Sterile 96-well PCR Plates – UV cross-linked prior to use.
Gloves: Nitrile or Latex, various sizes
Forceps: cleaned by soaking a minimum of 10 min in a 10% hypochlorous acid solution or by LookOut DNA Erase following manufacturer protocol or by autoclaving.
Sterile Foil Sealers for 96 well plates.
Sterile Optical Sealing Tape for 96 well plates.
Kimwipes®
Paper Towels
Countertop moisture barrier papers
Black Permanent Markers

SECTION 6

6. INTERNAL QUALITY CONTROL CHECKS

Details on quality control are found within each of the various protocol sections. In summary, however, quality control relative to sample contamination is covered by the transport (or cooler), equipment, DNA extraction, PCR, and sequencing blanks. Quality control for efficacy of methodology, solutions, etc. is covered by positive DNA controls for each sample handling step (sampling, filtering, extraction, PCR, and sequencing) of the eDNA protocol. Furthermore, each new solution or kit to be used in eDNA processing will be tested with positive and negative controls before use.

The ERDC eDNA processing facilities and protocols will be reviewed by an EPA audit shortly after completion of the QAPP and full deployment of Asian carp eDNA dedicated equipment.

6.1 Laboratory Quality Control Evaluation Criteria

Quality control is measured in two ways:

- If transport, filtering, extraction, PCR, and DNA sequencing negative controls show product (e.g., bands in PCR or DNA sequence), the associated data are negated and, when possible, samples are reprocessed. Contamination of DNA extract will require that samples be removed from consideration.
- Positive controls are currently employed for extraction, PCR, and sequencing. If the positive controls fail to behave as expected, any sample showing an apparent lack of results will be rerun at the same time or following rerunning of the positive controls. This will be done until all positive controls produce the expected results.
- We incorporate two types of positive controls during sequencing. One positive control PCR products from positive control reactions and several one sequencing reactions with a standard DNA sequencing template (pGem) provided by the manufacturer are sequenced with every set of 16 eDNA samples that are sequenced. In the case that any of these fail, any samples that fail to produce sequence data that were run at the same time will be rerun at the same time as positive controls are rerun.
- In cases where fewer than 16 eDNA samples are sequenced, both types of positive sequence controls are still run.

SECTION 7

7. SPECIFIC ROUTINE PROCEDURES TO ASSESS DATA PRECISION, ACCURACY, AND COMPLETENESS

7.1 Field Measurements

During each filtering event, two positive control samples, consisting of dilute solutions of carp scales and slime coat, will be pipetted into 2L aliquots of clean water and filtered according to filtering protocols. Filters will be sent to ERDC for processing. ERDC will report results of PCR reactions from filtering positive controls.

7.2 Laboratory Data

Every 3 months, a dilution series of Asian carp DNA will be processed from extraction through sequencing to ensure that current practice, instruments, and personnel are maintaining the same level of sensitivity and accuracy. A brief report will be provided by the DNA Processing Lead to the eDNA Program Manager.

SECTION 8

8. CORRECTIVE ACTIONS

Corrective actions may be required for two classes of problems: analytical/equipment problems and noncompliance problems. Analytical and equipment-related problems may develop during sampling and sample handling, sample preparation, laboratory instrumental analysis, and data review. Noncompliance issues arise when eDNA sampling, filtering, or processing execution deviates from procedures described in the QAPP.

In the case of analytical/equipment problems or deviations from set procedures (as outlined in QAPP), the responsible lead will determine if the problem or deviation will impact the accuracy of the resulting data. If it is determined that the problem or deviation does impact data accuracy, two courses of action may be followed:

(1) If possible, the procedure is repeated until it is performed without any problem or deviation, or

(2) The sample or samples are removed and not processed any further.

In either case, a corrective action report must be completed. Careful notes of any corrective actions and what incident led to them, as well as the resolution or preventative measure(s) identified will be carefully noted in the corrective action report, which must be provided electronically to all Leaders (Project Leader, eDNA Program Manager, etc) as an after action report. The paper copy of the corrective action report will be maintained in the project file as a long-term record.

In the case that the responsible lead determines that data accuracy is not affected by the analytical/equipment problem or deviation from procedure, the sample or samples may continue to be processed. The responsible lead will make careful note of the incident in project records and include the rationale for continuing processing.

SECTION 9

9. PREVENTATIVE MAINTENANCE PROCEDURES

9.1 Field Equipment/Instruments

Hand-held sonar: Batteries will be changed at least once a month (if not required sooner) to ensure accurate readings of the instrument. In addition, reading accuracy should be checked once a month. Depth readings may be checked by filling a container of a known depth with water and submerging the instrumentation. Temperature readings of the sonar may be checked against a thermometer.

GPS equipment: Batteries will be changed at least once a month (if not required sooner) to ensure accurate readings of the instrument. In addition, coordinate accuracy will be checked against known benchmarks.

Plastic 2L sample bottles: After autoclaving, bottles will be inspected for dents and/or warping of the material. Any bottle failing inspection will be disposed of and replaced.

Forceps: Forceps will be inspected monthly, and those exhibiting large amounts of rust will be disposed of and replaced.

Carboys: Carboys will be inspected monthly for cracks in the glass that could pose a safety hazard to filtering personnel. Any carboy failing inspection will be disposed of and replaced.

Plastic tubing: Plastic tubing used to connect the carboy to the manifold will be inspected monthly for cracks in the plastic. Any plastic tubing failing inspection will be disposed of and replaced.

All other laboratory equipment will be inspected monthly and undergo proper maintenance to maintain their ideal working condition. Any equipment not performing accurately or to established standards will be disposed of and replaced.

9.2 Laboratory Instruments

Pipettes: Annually all pipettes will be inspected, calibrated, and certified. Any pipette failing inspection and certification will be disposed and replaced.

Any thermal-cycler head that fails the manufacturers self-test upon instrument startup will be removed and replaced with the manufacturer’s certified replacement part.

Equipment maintenance contracts, with annual maintenance check-ups, will be used for any appropriate equipment (i.e., DNA sequencer).

SECTION 10

10. PERFORMANCE AND SYSTEM AUDITS

10.1 Field Audits

Internal audits of field crew performance and quality controls may be made semi-annually by the EDNA Program Manager to make sure that all procedures in the sample collection portions of the QAPP are being followed. A brief report will be made to the eDNA Program Manager of audit findings, including a signed checklist of audited procedures.

10.2 Laboratory Audits

Internal audits of USACE laboratory performance and quality controls will be made semi-annually by the Filtering Leader to make sure that all procedures in the sample filtering portions of the QAPP are being followed. A brief report will be made to the eDNA Program Manager of audit findings, including a signed checklist of audited procedures.

Internal audits of ERDC laboratory performance and quality controls will be made semi-annually by the DNA Processing Lead to make sure that all procedures in the DNA processing portions of the QAPP are being followed. A brief report will be made to the eDNA Program Manager of audit findings, including a signed checklist of audited procedures. Every 2 years an external review of ERDC eDNA processing will be undertaken. The review panel or consultant(s) will be selected by the Project Lead. The DNA Processing Lead may assist the eDNA Program Manager in identifying one or more potential reviewers.

EXHIBITS

Exhibit 1
Form 1

Field Collection Summary

Sample Date ____________ **Sample Collector** ____________ **Data Recorder** ____________

Location ____________

Samples (range) ____________ Blanks (CI) ____________ (CII) ____________

(CIII) ____________ (CIV) ____________ (CV) ____________ (CVI) ____________

Boat ramp (lat, long) ____________

USACE Personnel ____________

Other Personnel ____________

Boat Driver ____________

Prep List (Initial and Date)

Bottles autoclaved ____________

Coolers bleached ____________

Bottles loaded ____________

Blanks filled ____________

Boat bleached ____________

(See opposite side for Gear Checklists)

Time Frame (EST)

Start ____________ End ____________

Bottles Iced ____________

Return to USACE ____________

Other Notes: ____________

Map: Indicate starting positon on map with (*) then show general path taken during sampling using arrows

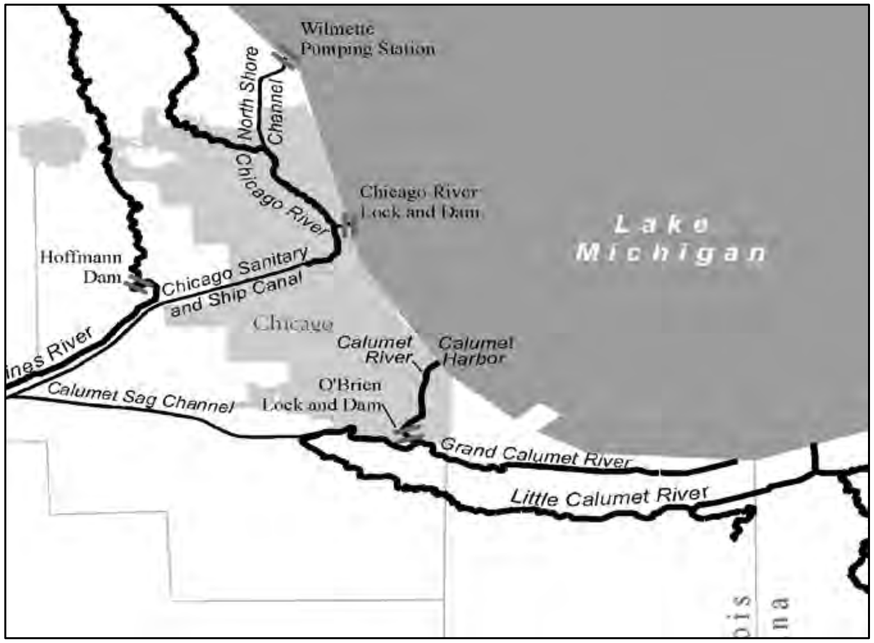

Exhibit 1
Form 1

Field Collection Summary

Gear Checklist

Coolers

- [ ] Labled Bottles

Clipboard

- [ ] Pencils
- [ ] Data Sheets
- [ ] GPS
- [ ] Extra AA Batteries
- [ ] Maps

Drybag

- [ ] Gloves
- [ ] Depth/Temp Torch
- [ ] Wet Wipes
- [ ] Sunblock

Others

- [ ] Bleach
- [ ] Mop
- [ ] Bucket
- [ ] Camera
- [ ] IPass
- [ ] Life Jackets

Exhibit 2
Form 2

DATE__________________ NAME________________________________ START TIME______________ SHEET _____of_______

ID	Volume	Latitude	Longitude	Temp ($^0F/^0C$)	Depth (Ft/M)	Double (D) Blank (B)	Habitat (LBD/MC/RBD)	Collect Time	Filter Time

Notes/Comments:

Exhibit 2
Form 2

DATE__________________ NAME____ START TIME_______________ SHEET _____of_______

ID	Volume	Latitude	Longitude	Temp ($^0F/^0C$)	Depth (Ft/M)	Double (D) Blank (B)	Habitat (LBD/MC/RBD)	Collect Time	Filter Time

Notes/Comments:

Exhibit 3
Form 3

US Army Corps of Engineers	CHAIN OF CUSTODY RECORD	File No.<br>Inv.

Date and Time of Collection:	River Reach:	Collected By:

Notes:

Collection No.	Description of Collection (include river reach, river mileage (if known), and any serial numbers):

Collection No.	From: (Print Name, Agency)	Release Signature:	Release Date:	Delivered Via:<br>☐ U.S. Mail<br>☐ In Person<br>☐ Other:
	To: (Print Name, Agency)	Receipt Signature:	Receipt Date:	
Collection No.	From: (Print Name, Agency)	Release Signature:	Release Date:	Delivered Via:<br>☐ U.S. Mail<br>☐ In Person<br>☐ Other:
	To: (Print Name, Agency)	Receipt Signature:	Receipt Date:	
Collection No.	From: (Print Name, Agency)	Release Signature:	Release Date:	Delivered Via:<br>☐ U.S. Mail<br>☐ In Person<br>☐ Other:
	To: (Print Name, Agency)	Receipt Signature:	Receipt Date:	
Collection No.	From: (Print Name, Agency)	Release Signature:	Release Date:	Delivered Via:<br>☐ U.S. Mail<br>☐ In Person<br>☐ Other:
	To: (Print Name, Agency)	Receipt Signature:	Receipt Date:	
Collection No.	From: (Print Name, Agency)	Release Signature:	Release Date:	Delivered Via:<br>☐ U.S. Mail<br>☐ In Person<br>☐ Other:
	To: (Print Name, Agency)	Receipt Signature:	Receipt Date:	
Collection No.	From: (Print Name, Agency)	Release Signature:	Release Date:	Delivered Via:<br>☐ U.S. Mail

Exhibit 3
Form 3

	To: (Print Name, Agency)	Receipt Signature:	Receipt Date:	☐ In Person<br>☐ Other:
Collection No.	From: (Print Name, Agency)	Release Signature:	Release Date:	Delivered Via:<br>☐ U.S. Mail<br>☐ In Person<br>☐ Other:
	To: (Print Name, Agency)	Receipt Signature:	Receipt Date:	
Collection No.	From: (Print Name, Agency)	Release Signature:	Release Date:	Delivered Via:<br>☐ U.S. Mail<br>☐ In Person<br>☐ Other:
	To: (Print Name, Agency)	Receipt Signature:	Receipt Date:	
Collection No.	From: (Print Name, Agency)	Release Signature:	Release Date:	Delivered Via:<br>☐ U.S. Mail<br>☐ In Person<br>☐ Other:
	To: (Print Name, Agency)	Receipt Signature:	Receipt Date:	
Collection No.	From: (Print Name, Agency)	Release Signature:	Release Date:	Delivered Via:<br>☐ U.S. Mail<br>☐ In Person<br>☐ Other:
	To: (Print Name, Agency)	Receipt Signature:	Receipt Date:	
Collection No.	From: (Print Name, Agency)	Release Signature:	Release Date:	Delivered Via:<br>☐ U.S. Mail<br>☐ In Person<br>☐ Other:
	To: (Print Name, Agency)	Receipt Signature:	Receipt Date:	
Collection No.	From: (Print Name, Agency)	Release Signature:	Release Date:	Delivered Via:<br>☐ U.S. Mail<br>☐ In Person<br>☐ Other:
Collection No.	From: (Print Name, Agency)	Release Signature:	Release Date:	Delivered Via:<br>☐ U.S. Mail<br>☐ In Person<br>☐ Other:
	To: (Print Name, Agency)	Receipt Signature:	Receipt Date:	
Collection No.	From: (Print Name, Agency)	Release Signature:	Release Date:	Delivered Via:<br>☐ U.S. Mail<br>☐ In Person<br>☐ Other:

Exhibit 4

USACE ERDC EL EP-P SAMPLE RECEIPT CHECKLIST

Project:	Receipt Date:
Other:	Rec'd By:

		Comments:
Were Samples Shipped?	Yes, FEDEX/UPS/Other____________________<br>No, Courier Pickup/Hand Delivered	
Cooler Temperature Upon Arrival ______________________?C/NA		
Chain of Custody Present?<br>Complete?	Yes/No<br>Yes/No	
Custody Seals Present on Cooler?<br>Samples?	Yes/No<br>Yes/No	
Were Sample Containers Intact?	Yes/No	
Samples and COC Match?		
If Any Problems Was Project Manager Notified? Yes/No<br>By Whom?____________________________		
Appropriate Sample Containers?	Yes/No	
Date/Time of Collection on COC	Yes/No	
Location and ID of Sample Storage:	Freezer ______________________________<br>Refrigerator ________________________	
Temperature Log updated for Storage:	Yes/No	

Exhibit 5

Refrigerator Temperature Log

Month/Year:______________ Days 1–15

Refrigerator ID:______________________________ **Refrigerator Location:______________________________**

	Day of Month	1		2		3		4		5		6		7		8		9		10		11		12		13		14		15	
	Staff Initials																														
	Room Temp.																														
	Exact Time																														
	?C Temp	am	pm	am	pm	am	pm	am	pm	am	pm	am	pm	am	pm	am	pm	am	pm	am	pm	am	pm	am	pm	am	pm	am	pm	am	pm
Too Warm	?11?																														
	10?																														
	9?																														
	8?																														
	7?																														
	6?																														
	5?																														
IDEAL	4?																														
	3?																														
	2?																														
Too Cold	1?																														
	0?																														
	?1?																														

Freezer Temperature Log

Month/Year:______________ **Days 1–15**

Freezer ID:____ **Freezer Location:**____

	Day of Month	1		2		3		4		5		6		7		8		9		10		11		12		13		14		15	
	Staff Initials																														
	Room Temp.																														
	Exact Time																														
	?C Temp	am	pm	am	pm	am	pm	am	pm	am	pm	am	pm	am	pm	am	pm	am	pm	am	pm	am	pm	am	pm	am	pm	am	pm	am	pm
Too Warm	?-12?																														
	-13?																														
	-14?																														
	-15?																														
	-16?																														
	-17?																														
	-18?																														
	-19?																														
IDEAL	-20?																														
	-21?																														
	-22?																														
	-23?																														
	-24?																														
	-25?																														

Ultra-Low Freezer Temperature Log

Month/Year:______________ **Days 1–15**

Freezer ID:____________________________________ **Freezer Location:**____________________________________

	Day of Month	1		2		3		4		5		6		7		8		9		10		11		12		13		14		15	
	Staff Initials																														
	Room Temp.																														
	Exact Time																														
	?C Temp	am	pm	am	pm	am	pm	am	pm	am	pm	am	pm	am	pm	am	pm	am	pm	am	pm	am	pm	am	pm	am	pm	am	pm	am	pm
Too Warm	?-70?																														
	-71?																														
	-72?																														
	-73?																														
	-74?																														
	-75?																														
	-76?																														
	-77?																														
	-78?																														
	-79?																														
IDEAL	-80?																														
	-81?																														
	-82?																														
	-83?																														
	-84?																														
	-85?																														

Exhibit 6

PCR Log System

Plate ID	PCR Machine ID	Tetrad Heads ID	Plate Orientation	PCR Program	Run Start Time	Run Finish Time

Exhibit 7

Quality Assurance Project Plan Certification Statement

I, the undersigned, certify that I have read and that I understand the Quality Assurance Project Plan (QAPP) for the eDNA Monitoring of Invasive Asian Carp in the Chicago Area Waterway System. I further certify that I will follow the procedures listed in this QAPP.

Signed:

Name	Agency	Date

APPENDIX A

STAFF ASSIGNMENTS

As of 22 May 2012, the following staff are assigned to the eDNA Monitoring Project.

Project Leader: Rich Hancock, LRD, 513-684-3005

eDNA Program Manager: Kelly Baerwaldt, LRD, 309-794-5285

Sampling Quality Assurance Specialist: Shawna Herleth-King, LRC, 312-846-5407

Filtering Leader: Shawna Herleth-King, LRC, 312-846-5407

Filtering Quality Assurance Specialist: Nicholas Barkowski, LRC, 312-846-5578

eDNA Processing Leader: Richard Lance, ERDC, 601-634-3971

DNA Processing Quality Assurance Specialist: Xin Guan, Badger Technical Services (ERDC onsite), 601-634-3022

Data Documentation & Reporting Specialist: Matthew Carr, Badger Technical Services (ERDC onsite), 601-634-4840

Supporting Agency Contacts

USFWS
Sam Finney, Carterville Fish and Wildlife Conservation Office, 618-997-6869
Pam Thiel, LaCrosse Fisheries Resource Office, 608-783-8431
Tracy Hill, Columbia Fish and Wildlife Conservation Office, 573-234-2132

ILDNR
Vic Santucci, Illinois Dept of Natural Resources, 847-608-3100 x 2011

USEPA
Aaron Jastrow, USEPA, 312- 353-7386

APPENDIX B

MONITORING AND RAPID RESPONSE WORK GROUP (MRRWG)
MONITORING AND RAPID RESPONSE PLAN (MRRP)

STRATEGY FOR eDNA MONITORING IN THE CAWS
AND UPPER DES PLAINES RIVER

Strategy for eDNA Monitoring in the CAWS

Participating Agencies: USACE (lead), USFWS and IDNR (field support), USEPA (field and lab support)

Location: Monitoring will take place in the CAWS upstream of Lockport Lock and Power Station

Introduction and Need: Monitoring is essential to determine the effectiveness of efforts to prevent self-sustaining populations of Asian carp from establishing in the Great Lakes. In the past, traditional fishery techniques have been used to detect the presence of Asian carp in the Upper Illinois Waterway; however, these methods have been somewhat ineffective at targeting these species at low densities. With funding from USACE, the University of Notre Dame applied a method to detect environmental DNA (eDNA) left behind in the aquatic system by the targeted species (Jerde et al., 2011). Use of this method has been to provide detection of Asian Carp DNA where fish, if they exist at all, exist at very low densities (e.g., CAWS). The results of eDNA sampling in conjunction with traditional fishery techniques will guide rapid response actions designed to remove Asian carp from the waterway. Results of eDNA sampling will also be used to inform decisions regarding the success of removal efforts and when individual actions should be terminated.

At present, the capacity to process eDNA is 120 samples per week. The sampling strategy for the 2012 field season takes into account the current level of sample processing, but the number of samples required was determined based on sampling regime and results from prior years (i.e., 2009-2011), individual site characteristics, and the need to gather information from several strategically important reaches of the waterway.

Objectives: eDNA sampling will be used to:

1) Determine whether Asian carp DNA is present in strategic locations in the CAWS to help guide rapid response actions; and
2) Detect Asian carp DNA in areas targeted for rapid response actions, as a measure of the effectiveness of conventional gear or rotenone removal efforts

Status: Sampling for Asian carp DNA began during June 2009 in the upper Illinois River and continued through August 2010 at other locations, including the Des Plaines River, CAWS, and near shore areas of Lake Michigan. In the summer of 2010, Federal agencies assumed the lead for eDNA monitoring. The USACE became responsible for coordinating sampling, processing samples, and posting results; while the U.S. Fish and Wildlife Service and Illinois Department of Natural Resources became responsible for sample collection. During the 2011 field season, a total of 1693 water samples were collected from the CAWS upstream of the Dispersal Barrier, 57 from below the Dispersal Barrier, and 114 from the upper Des Plaines River (i.e., between Hofmann Dam and the Lemont Road Bridge). In addition, 684 samples were collected over the course of three days in October from the CAWS as part of the eDNA snapshot sampling event. Detailed results from the 2011 field season are available in the 2011 interim summary report document (MRRWG 2012).

To date, no relationship between the number of positive detections and Asian carp population abundance has been established, therefore eDNA results should be interpreted with caution. Additional research on the calibration of the eDNA method has been occurring since 2010; however, the full results of this multi-agency study will not be known until 2013. Until completion of this additional research to calibrate eDNA results and assess potential alternative sources of DNA in the waterway, the MRRWG views positive eDNA results as an indicator of the possible presence of live Asian carp. When viewed over the long term (e.g., multiple positive hits on consecutive sample dates at the same location), these data will be used to guide decisions on the location and timing of targeted rapid response removal actions.

Methods: Standard operating procedures have been outlined in the eDNA Quality Assurance Project Plan (USACE 2011) and were reviewed and agreed upon by all partnering agencies (e.g. USACE, USFWS, and IDNR). In general, IDNR and USFWS will collect 60 water samples on a bi-monthly basis from a specified reach on Monday or Tuesday. Samples will be transferred to USACE biologists at the USEPA laboratory in Chicago where they will be filtered and preserved in a -20ºC freezer. Preserved samples will be shipped overnight to the ERDC laboratory for analysis. Results will be posted on a USACE web site after analysis of each sampling event is complete (approximately 14 days). A general description of the eDNA sample collection method is given below. Detailed field, laboratory, and reporting protocols are available in the eDNA Quality Assurance Project Plan (USACE 2011).

Locations-Samples will be collected every two weeks from late May through October (weather permitting) such that Lake Calumet and each partial barrier to Lake Michigan are sampled once every 30 days (N= 114 samples and 6 cooler blanks bi-monthly; Figure 4). Sample locations were selected based on habitat thought to be preferred by Asian carp (Lake Calumet) and entry points to Lake Michigan (North Shore Channel downstream from Wilmette Pumping Station, Chicago River downstream from Chicago Lock, and Little Calumet River downstream from T. J. O‟Brien Lock and Dam. Sampling is complementary to fixed site sampling conducted with conventional gears in the locations listed below.

- North Shore Channel (60 samples) and South Branch Chicago River to the Chicago Lock (60 samples)
- Little Calumet River downstream of O‟‟Brien Lock (60 samples) and Lake Calumet (60 samples)

Paired sampling stations will be sampled in the same day once every other week so that all four stations will be sampled every 30 days. An additional 480 samples will be available for the 2012 field season. These samples will be used for sampling associated with rapid response actions or to monitor the Upper Des Plaines River, CSSC and Calumet-Sag Channel confluence, the CSSC upstream and downstream of the Dispersal Barrier, or other locations determined to be strategically important (e.g., re-sampling a site with previous positive detections for Asian carp DNA). The USACE will provide aerial site maps with specific sampling locations for each sample 1-2 weeks prior to each sampling event. In addition, sampling maps will be provided with the coolers upon pick-up from the USEPA laboratory, as well as a box of nitrile gloves, datasheets, COC form, handheld depth sonar, and sprayer with 10% bleach solution.

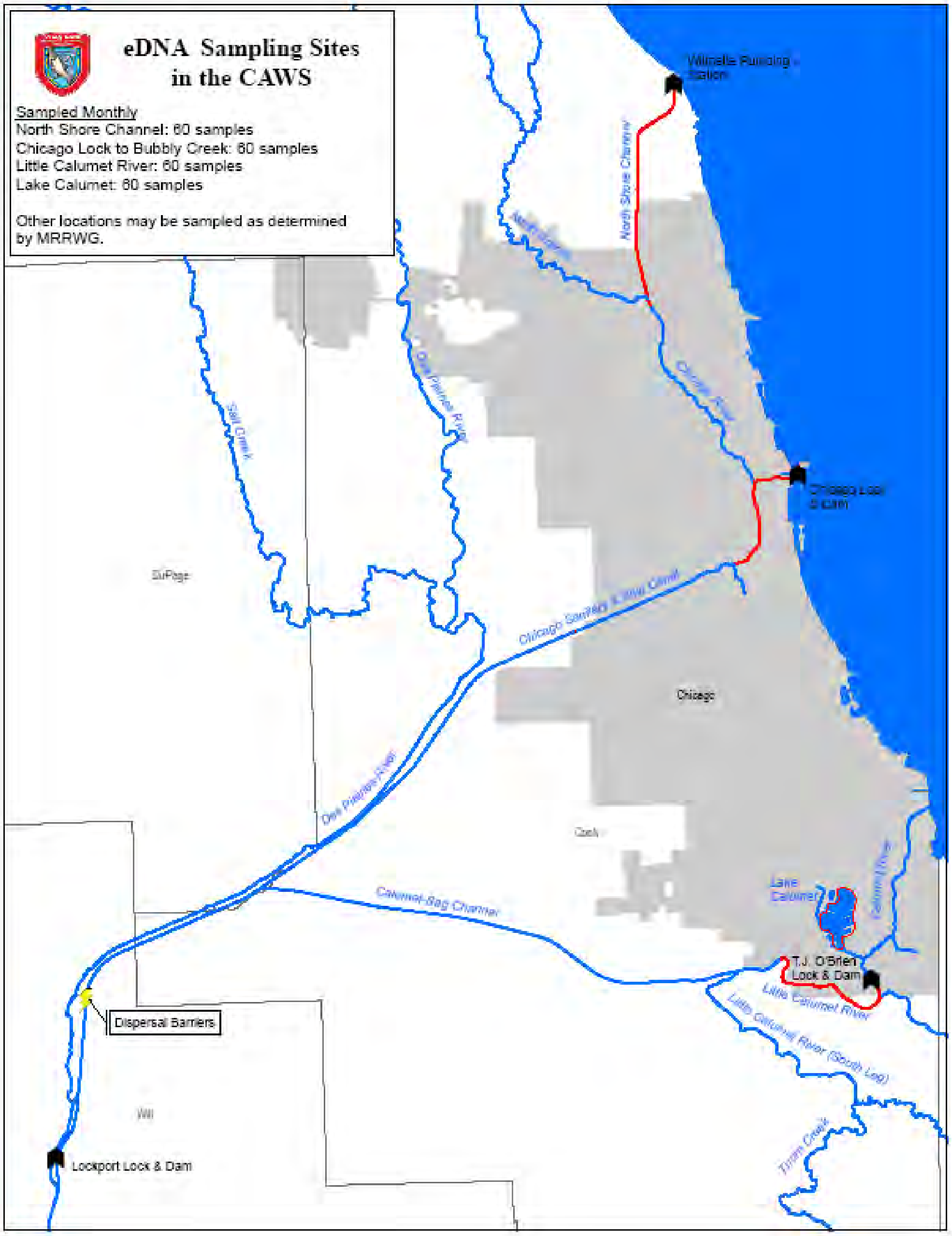

Figure 4. Locations for eDNA sampling in the CAWS.

The proposed strategy allows for eDNA sampling to take place in support of conventional gear or rotenone rapid response actions or other evaluations that might occur at locations other than those identified above. Highest sampling priority has been set for barriers to Lake Michigan (e.g. Wilmette Control Works, Chicago Lock and O'Brien Lock) and Lake Calumet. Sampling priority for the additional 480 samples that are not part of regular fixed site sampling will be directed by the MRRWG, and may include: 1) Rapid Response Action sites 2) CSSC upstream and downstream of the Dispersal Barrier; 3) CSSC and Calumet-Sag Channel confluence; and 4) upper Des Plaines River. A minimum of 60 samples is recommended for each eDNA sampling

event to reduce the probability of obtaining false negative results. Changes to the sampling frequency and/or procedures may be made by the MRRWG, as needed to minimize the risk of Asian carp entering the CAWS upstream of the barrier.

eDNA Sample Collection Protocol.

1) Sampling will be cancelled or postponed due to contamination concerns if a combined sewer overflow (CSO) occurs two days prior to sampling and/or if observed precipitation exceeds 1.5 inches in 24 hours five days prior to sampling. Sample crews will be notified as soon as possible of a cancellation.
2) The sampling boat and transport trailer must be disinfected prior to launching by spraying the outer surfaces (i.e. hull, motor, etc.) with a hand-held sprayer containing a prepared 10% bleach/water solution.
3) Prior to launch, crew members will be given their specific duties for the sampling trip. One crew member will be designated as the boat operator and will be in charge of driving the vessel to sample locations. A second crew member will be designated as the lead sampler and will be in charge of collecting all water samples and measuring water depth and temperature. A third crew member will record GPS location (decimal degrees) and habitat measurements for each water sample on a datasheet.
4) Sampling will begin at the first transect located at the DOWNSTREAM end of the reach to be sampled and will proceed in an UPSTREAM direction.
5) When arriving at a sample site, the lead sampler will put on sterile exam gloves (powderless latex or nitrile).
6) Going in consecutive order, the lead sampler will remove a labeled 2L sample bottle from the sample cooler.
7) Just prior to collecting the sample, the lead sampler will unscrew and remove the lid from the sample bottle.
8) The lead sampler will then reach over the upstream side or the bow of the boat with the 2L sample bottle and fill the bottle by skimming the water surface. The sample bottle should not be submerged or dipped beyond the upper 2 inches of the surface water for sample collection.
9) Once the sample bottle is completely filled (approximately 1 inch of space should be left within the sample bottle) the lead sampler will screw the lid back on to the bottle until it is tight. The closed bottle should then be returned to the sample cooler from which it was removed.
10) The lead sampler will take a surface water temperature and depth measurement at the sample site. The data recorder will record the bottle ID number, GPS location (decimal degrees), time of sample, water temperature, and water depth on the data sheet.
11) If the lead sampler pulls a transport blank (2L of DI water filled prior to trip) from the cooler, the sampler will unscrew and remove the lid to expose the bottles contents to the atmosphere for 5 seconds, reseal the bottle, fully submerge the bottle in the field water, and return the bottle to the cooler from which it was removed. The lead sampler should

relay to the data recorder that the sample was a blank, so that it can be recorded on the data sheet next to the appropriate ID number. BLANKS ARE TAKEN IN TANDEM WITH THE NEXT ACTUAL SAMPLE AND DO NOT REPLACE A SAMPLE IN THAT LOCATION. If a blank was collected, the boat will remain at the same location and an actual sample will be taken.

12) Duplicate samples are collected as part of quality control. Duplicate sample locations are designated as red stars on the aerial location map. Duplicate samples will be collected the same a regular sample; however, the lead sampler should relay to the data recorder that the sample is a duplicate, so that it can be recorded on the data sheet next to the appropriate sample ID. DUPLICATE SAMPLES ARE TO BE TAKEN IN TANDEM WITH THE NEXT REGULAR SAMPLE. If a duplicate sample is designated, this sample should be taken concurrently with the regular sample, side-by-side, to best replicate the regular sample collection. If a blank sample is pulled from the cooler at a designated duplicate location on the aerial map, take the duplicate sample at the NEXT DESIGNATED REGULAR SAMPLE LOCATION.
13) Steps 5 through 12 should be repeated until sampling has been completed for the targeted reach.
14) Once sampling is complete, ice will be added to the sample coolers as soon as possible. Enough ice should be added to each cooler to completely surround each sample bottle and maintain an inside temperature of 40ºF. If at any time during transport the inside temperature of the cooler(s) rises above 40ºF, additional ice should be added.
15) Chain-of-custody (COC) forms will be completed for every sample. All samples, including blanks, will be logged onto COC forms. The forms will be collected and signed whenever the coolers are transferred between parties.

Boat Launches for eDNA Sampling.
<u>North Shore Channel</u> – Western Avenue Launch – No contact needed.
<u>Chicago Lock to Bubbly Creek</u> – Western Avenue Launch – No contact needed.
<u>Little Calumet River</u> – O‟Brien Lock Launch – Contact the O‟Brien Lockmaster for permission. Will need to launch at O‟Brien Lock and lock through to sample downstream of lock and dam.
<u>Lake Calumet</u> – O‟Brien Lock Launch – Contact the O‟Brien Lockmaster for permission.

Sampling Schedule: A tentative sampling schedule for 2012 is shown in the table below. Date and agency assignments will remain fixed, whereas the station sampling will be assigned for each week by USACE following monitoring plan protocols described above.

Week of	Agency	Week of	Agency
May 21	IDNR	Aug 20	IDNR
Jun 11	USFWS	Sep 10	USFWS
Jun 25	IDNR	Sep 17	IDNR
Jul 9	USFWS	Oct 1	USFWS
Jul 23	IDNR	Oct 22	IDNR
Aug 6	USFWS		

Deliverable: Results of each sampling event will be reported on the USACE website http://www.lrc.usace.army.mil/AsianCarp/eDNA.htm within 2 weeks of sample collection. Data will be summarized for an annual interim report and project plans updated for annual revisions of the MRRP.

Field Points of Contact

Name	Agency	Office #	Mobile #	Email
Mike McClelland	IDNR	-	630-360-4180	Michael.McClelland@Illinois.gov
Peg Donnelly	USEPA	312-886-6109	312-860-1407	Donnelly.Peggy@epamail.epa.gov
Nick Bloomfield	USFWS-La Crosse	608-783-8441	309-371-4271	Nicholas_Bloomfield@fws.gov
Brett Witte	USFWS-Columbia	573-234-2182 x103	573-864-1579	Brett_Witte@fws.gov
Heather Calkins	USFWS-Columbia	573-234-2182 x164	314-808-1352	Heather_Calkins@fws.gov
Brad Rogers	USFWS-Carterville	618-997-6869	217-621-1818	Philip_Rogers@fws.gov

Laboratory Points of Contact

Name	Agency	Office #	Mobile #	Email
Aaron Jastrow	USEPA	312-353-7386	312-213-6537	Jastrow.Aaron@epamail.epa.gov
Rob Snyder	USEPA	312-353-9083	-	Snyder.Robert@epamail.epa.gov
Shawna Herleth-King	USACE	312-846-5407	312-806-8207	Shawna.S.Herleth-king@usace.army.mil
Matt Shanks	USACE	312-846-5581	312-806-3760	Matthew.R.Shanks@usace.army.mil
Nick Barkowski	USACE	312-846-5578	312-401-3927	Nicholas.A.Barkowski@usace.army.mil

APPENDIX C

SAMPLING PLANS FOR THE CURRENT MRRWG eDNA SAMPLING STRATEGY

North Shore Channel

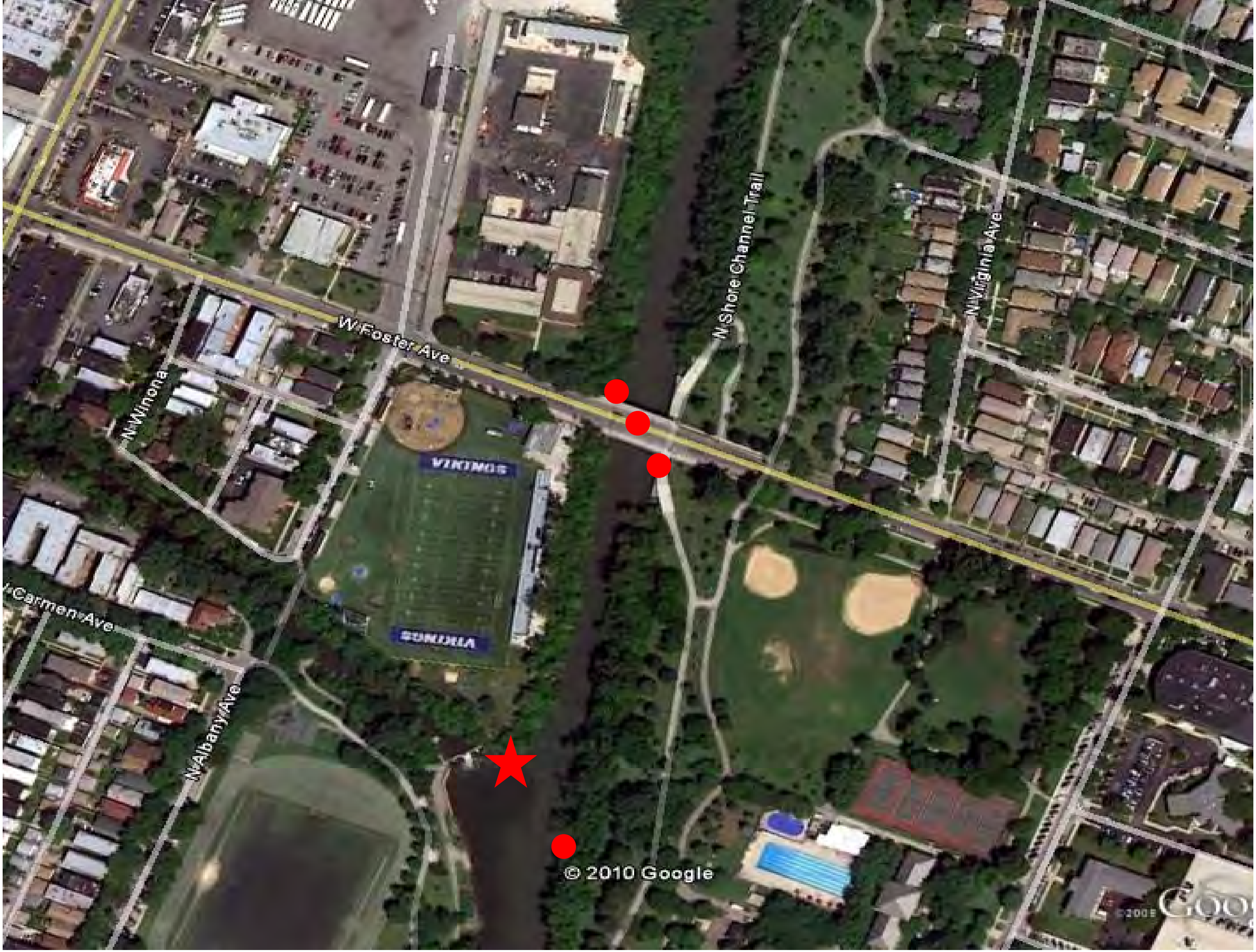
W Foster Ave
N Shore Channel Trail
N Virginia Ave
N Winona
VIKINGS
VIKINGS
W Carmen Ave
N Albany Ave
© 2010 Google

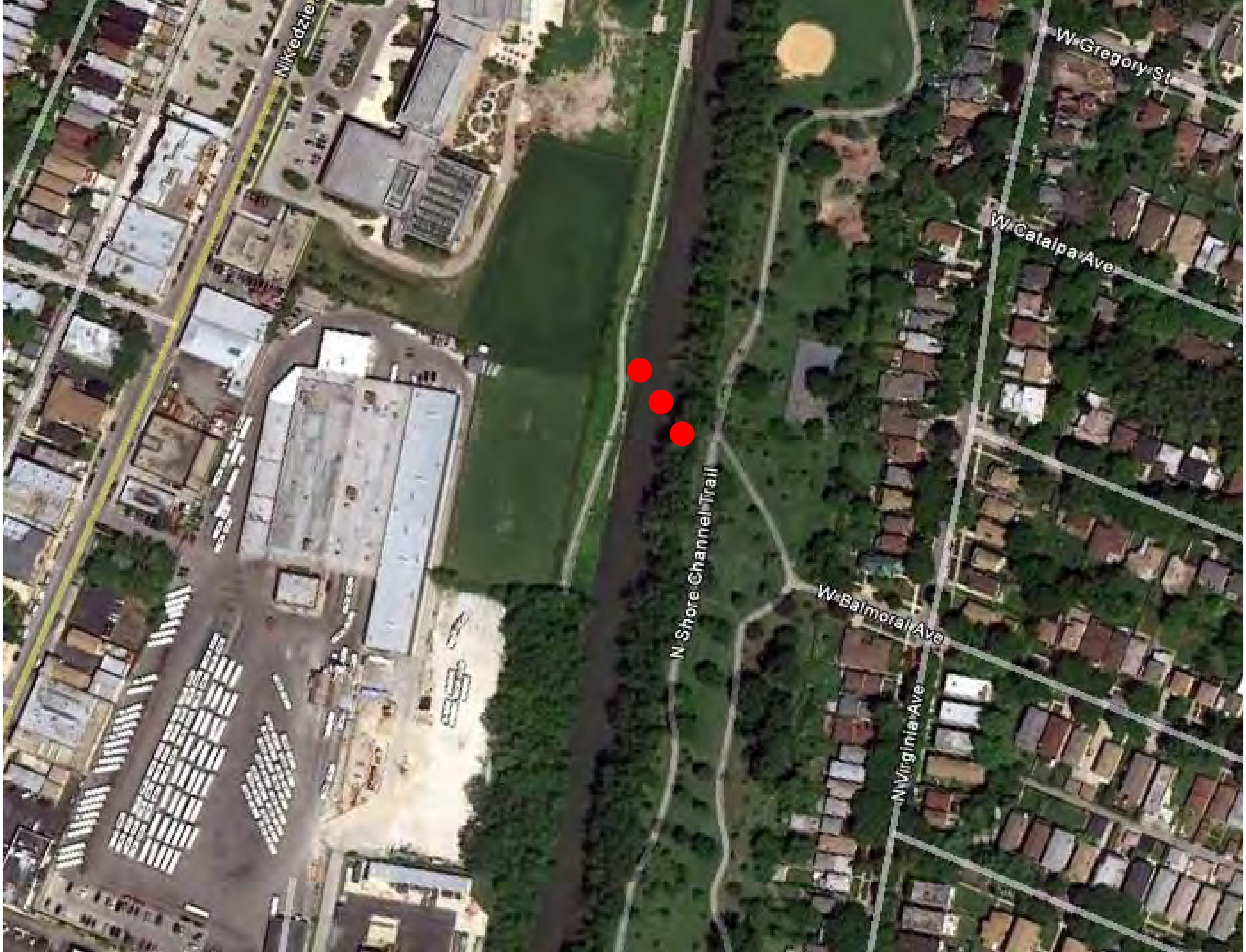
N Kedzie
W Gregory St
W Catalpa Ave
N Shore Channel Trail
W Balmoral Ave
N Virginia Ave

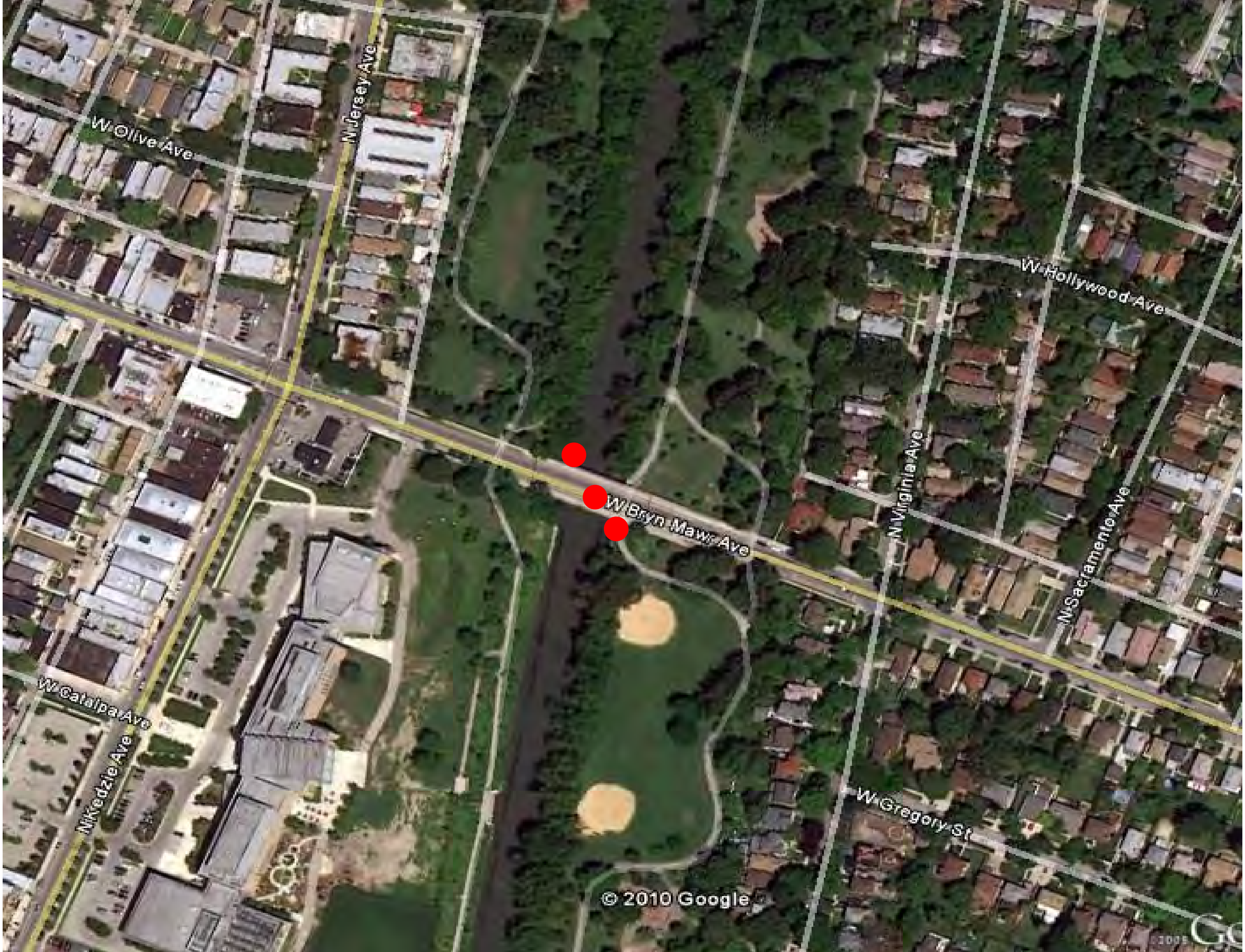
W Olive Ave
N Jersey Ave
W Hollywood Ave
W Bryn Mawr Ave
N Virginia Ave
N Sacramento Ave
W Catalpa Ave
N Kedzie Ave
W Gregory St
© 2010 Google

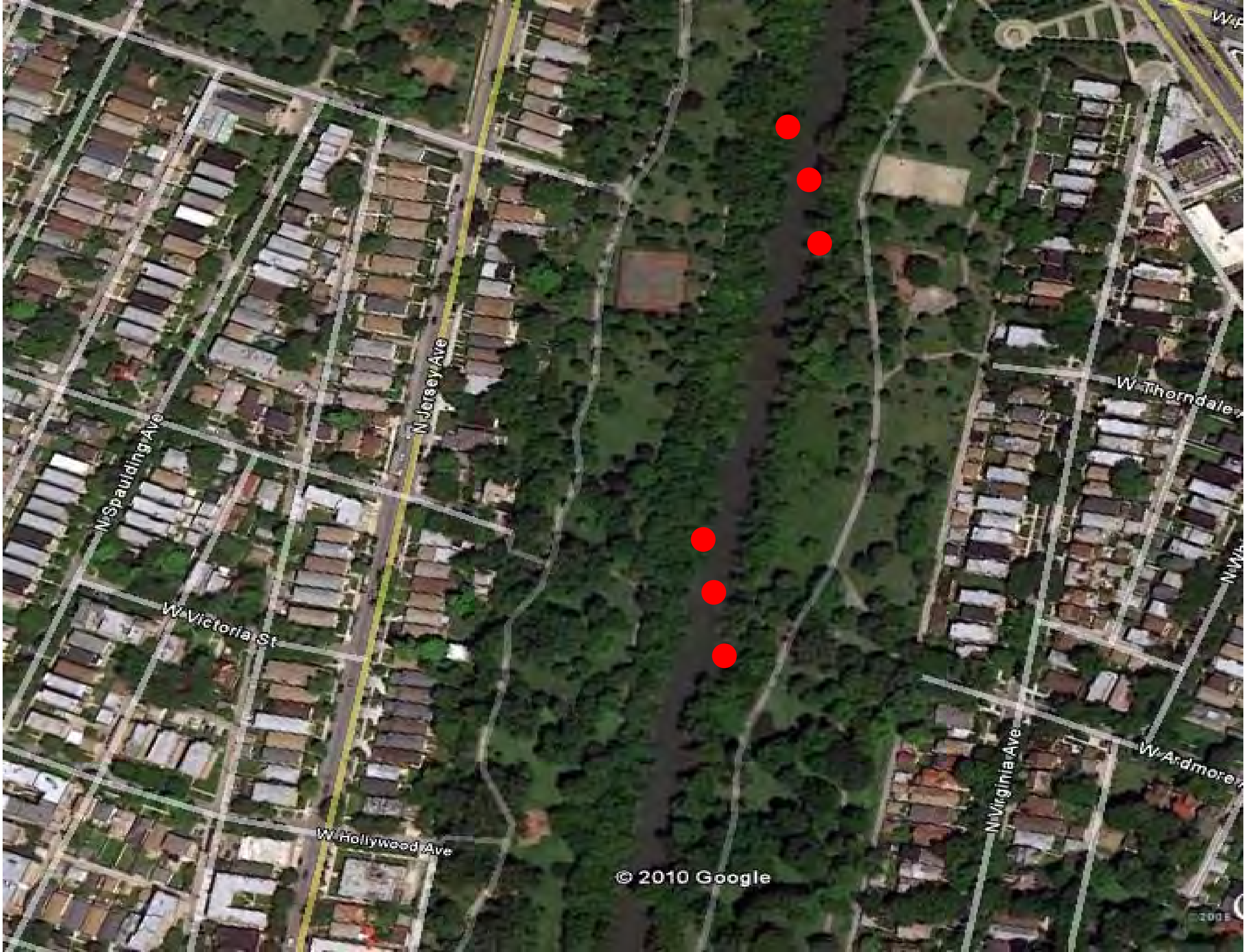
N Jersey Ave
N Spaulding Ave
W Victoria St
W Hollywood Ave
W Thorndale Av
W Ardmore
N Virginia Ave
© 2010 Google

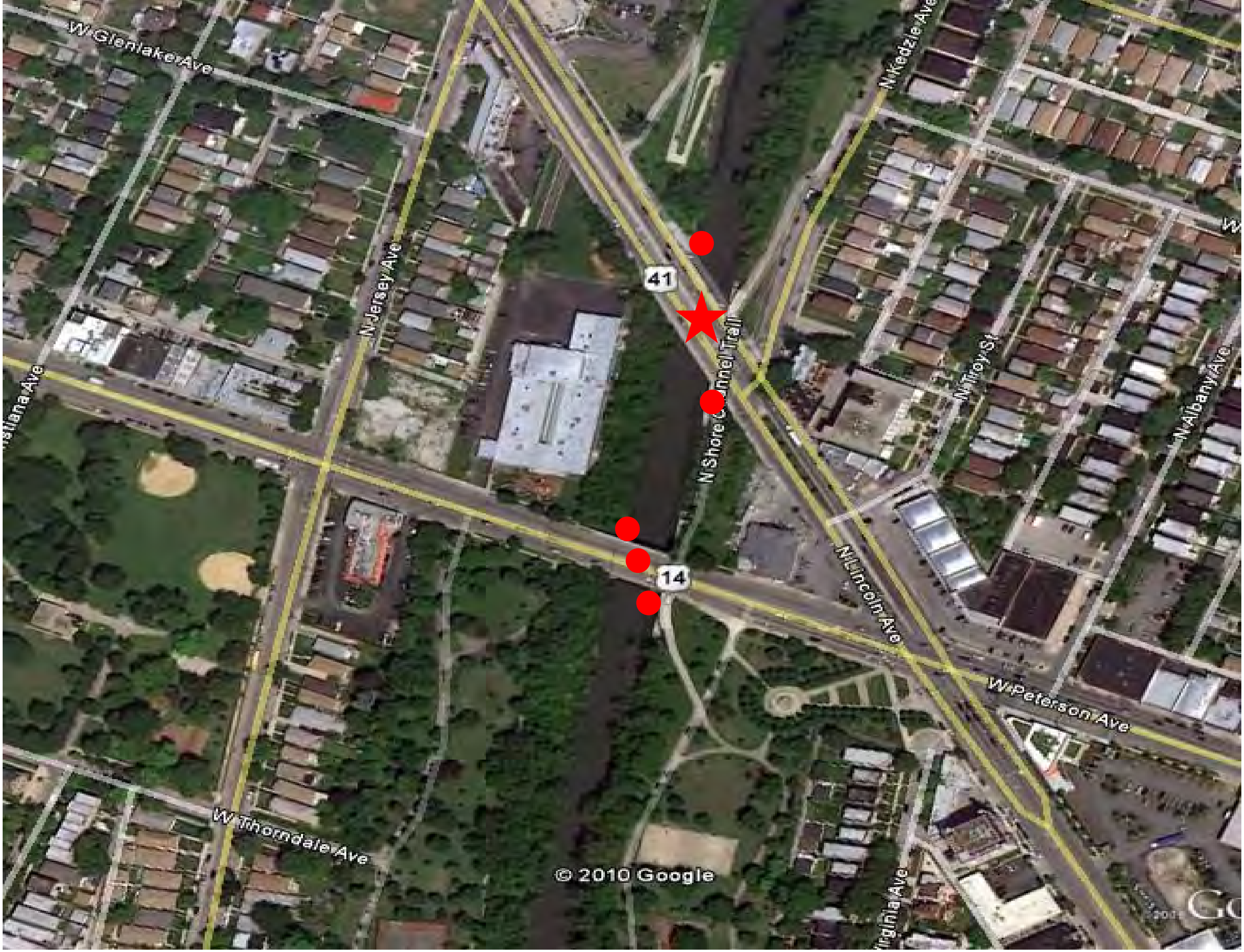
W Glenlake Ave
N Kedzie Ave
41
N Jersey Ave
N Shore Channel Trail
N Troy St
N Albany Ave
Christiana Ave
14
N Lincoln Ave
W Peterson Ave
W Thorndale Ave
© 2010 Google
Virginia Ave

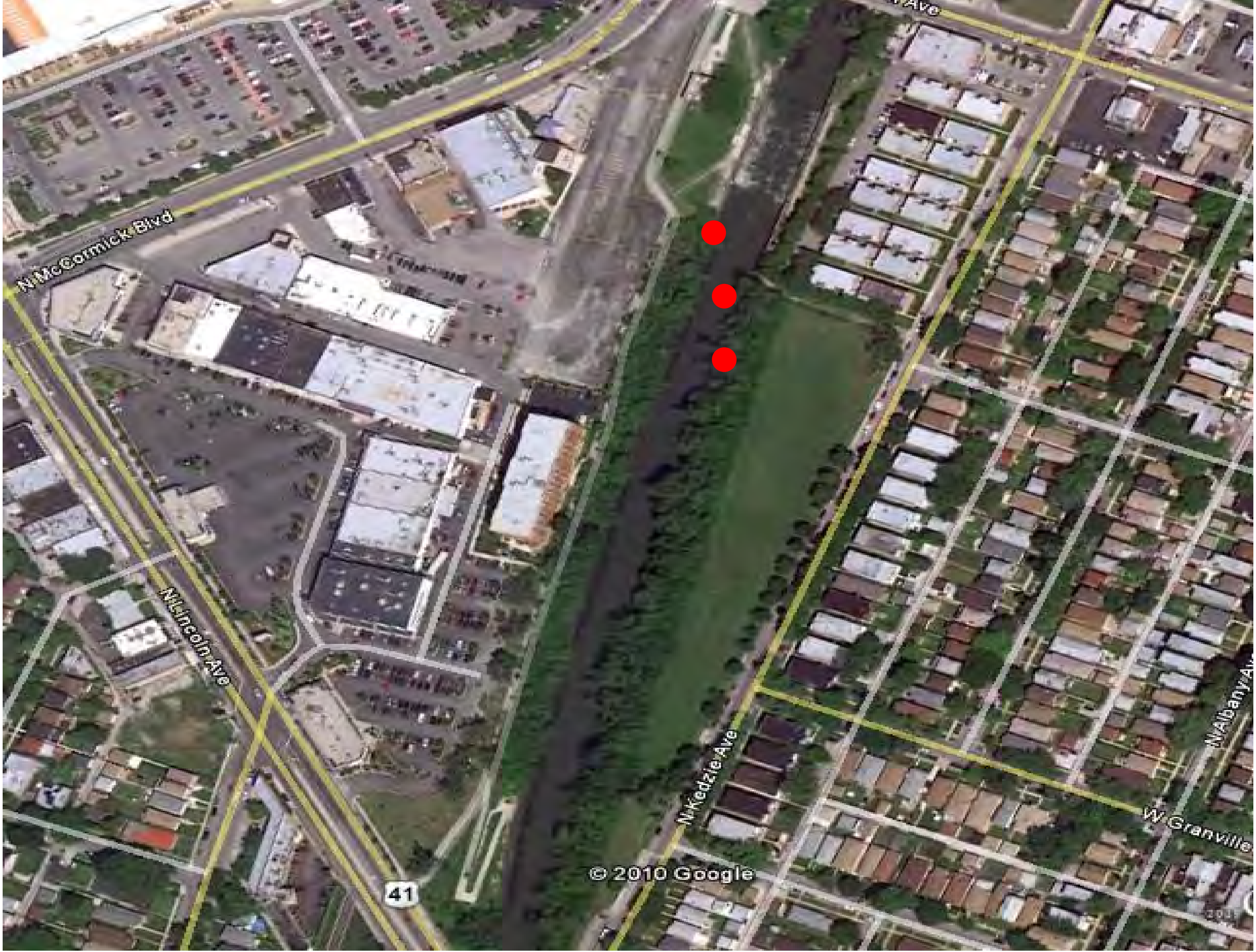
N McCormick Blvd
N Lincoln Ave
N Kedzie Ave
W Granville
N Albany Ave
41
© 2010 Google

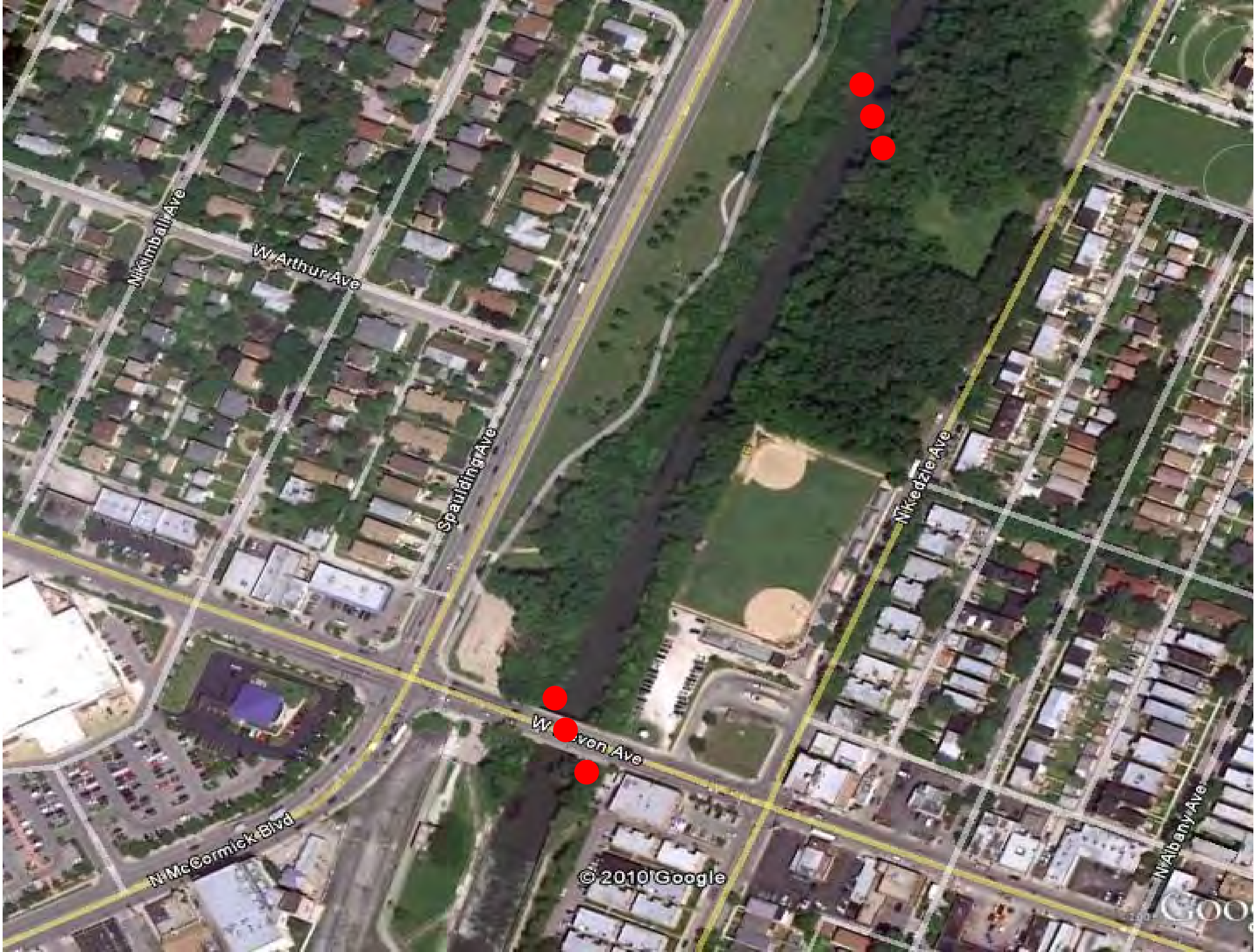

N Kimball Ave
W Arthur Ave
Spaulding Ave
N Kedzie Ave
N Albany Ave
N McCormick Blvd
© 2010 Google

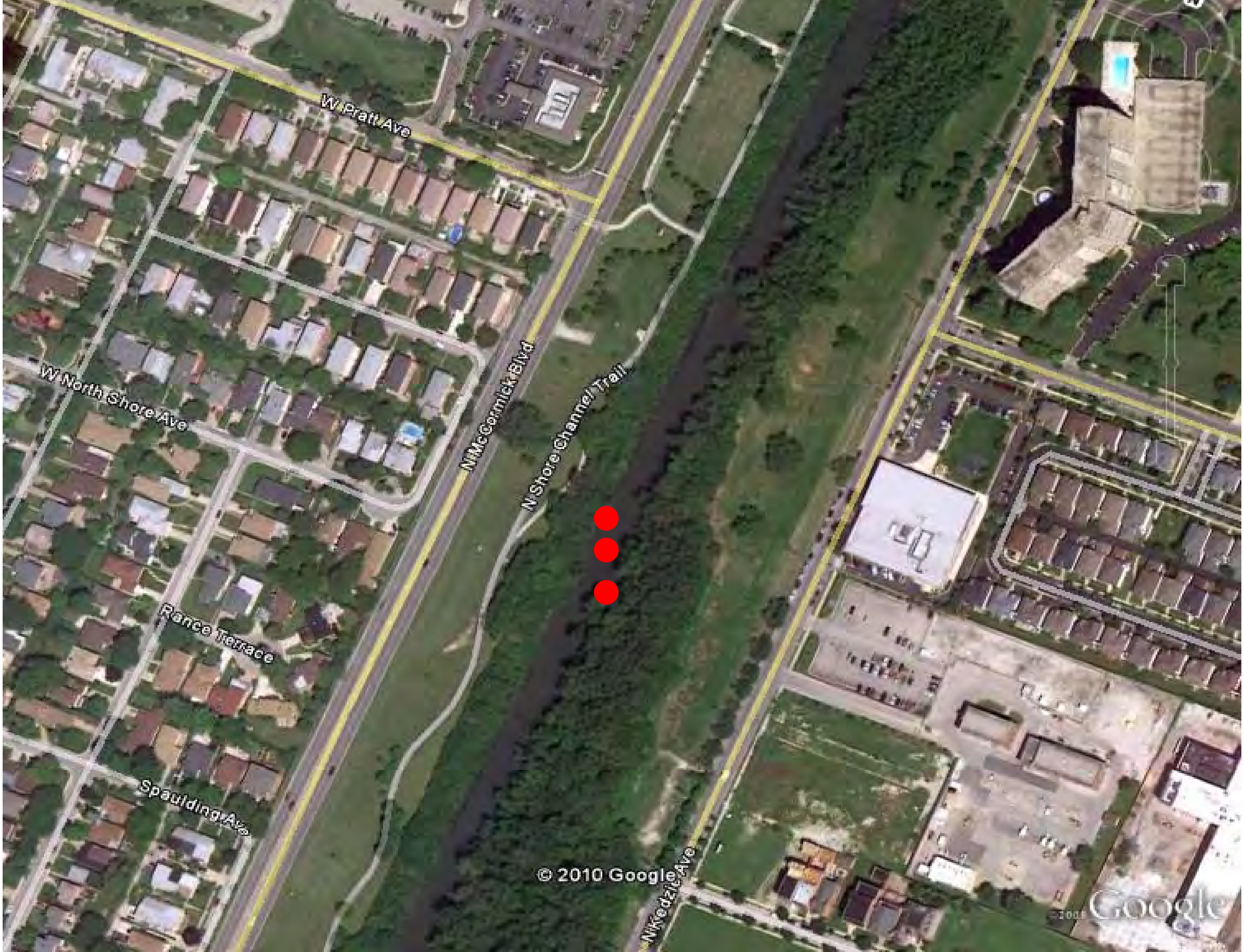
W Pratt Ave
W North Shore Ave
N McCormick Blvd
N Shore Channel Trail
Rance Terrace
Spaulding Ave
N Kedzie Ave
© 2010 Google
Google

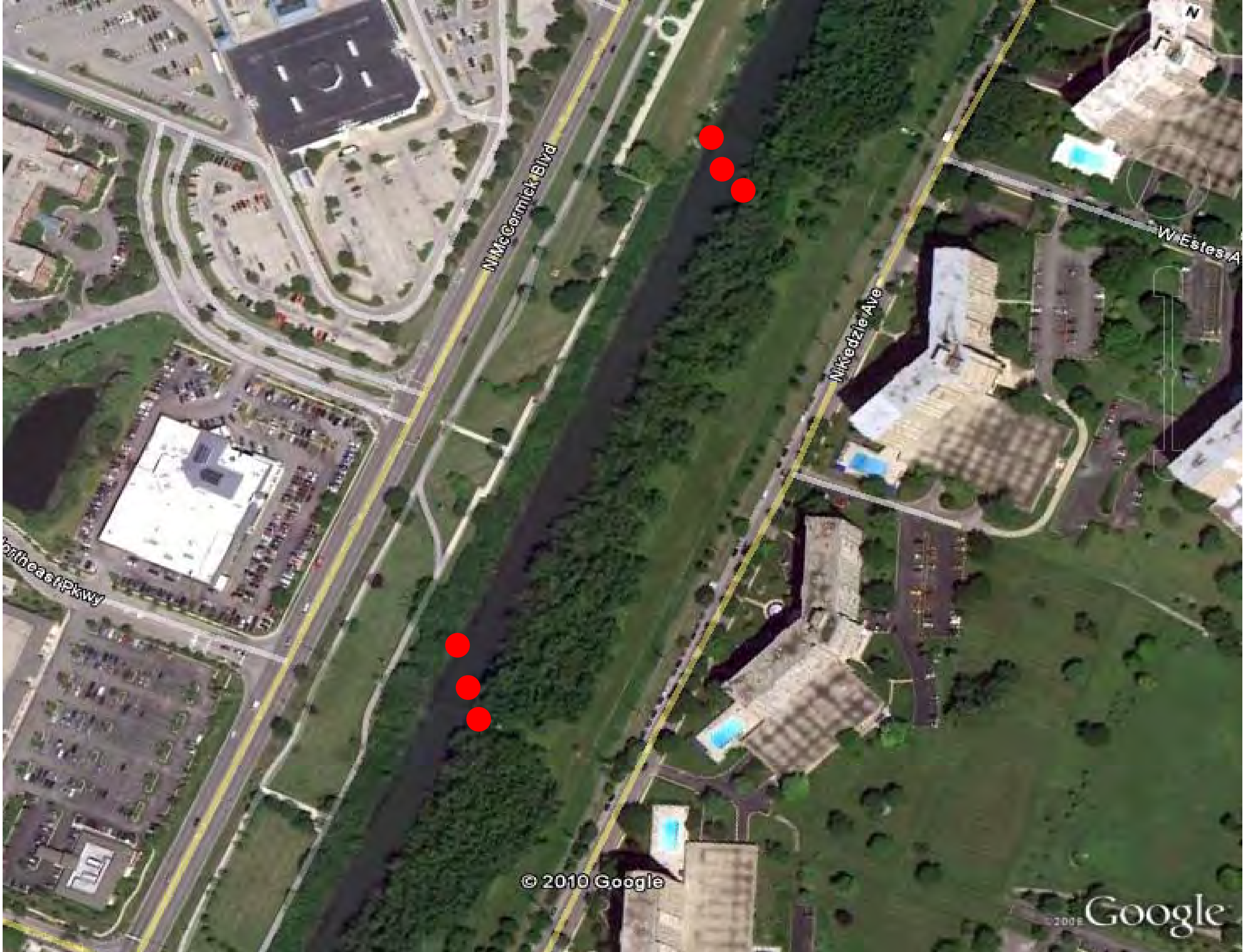
N McCormick Blvd
N Kedzie Ave
W Estes A
Northeast Pkwy
© 2010 Google
Google

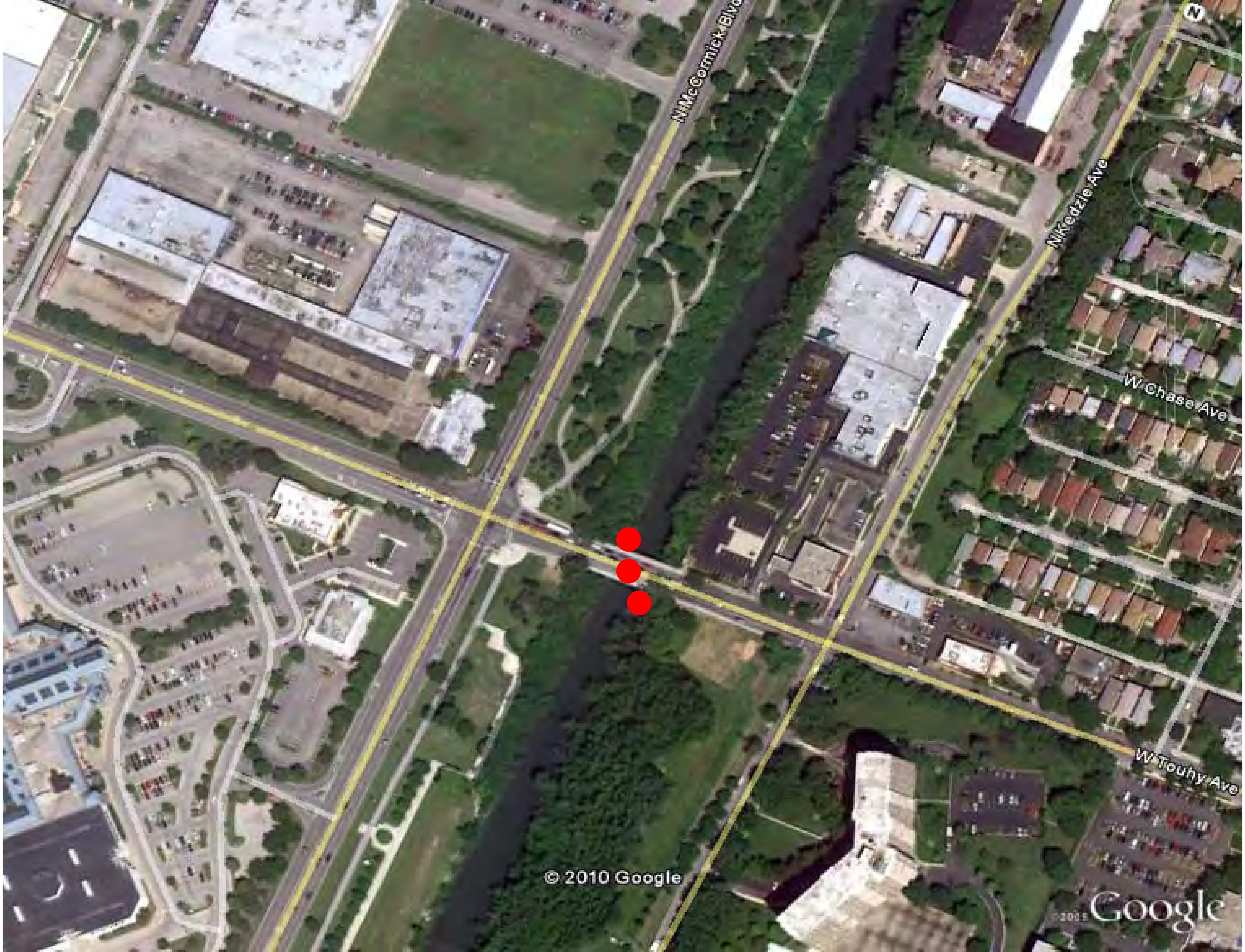
N McCormick Blvd
N Kedzie Ave
W Chase Ave
W Touhy Ave
© 2010 Google
Google

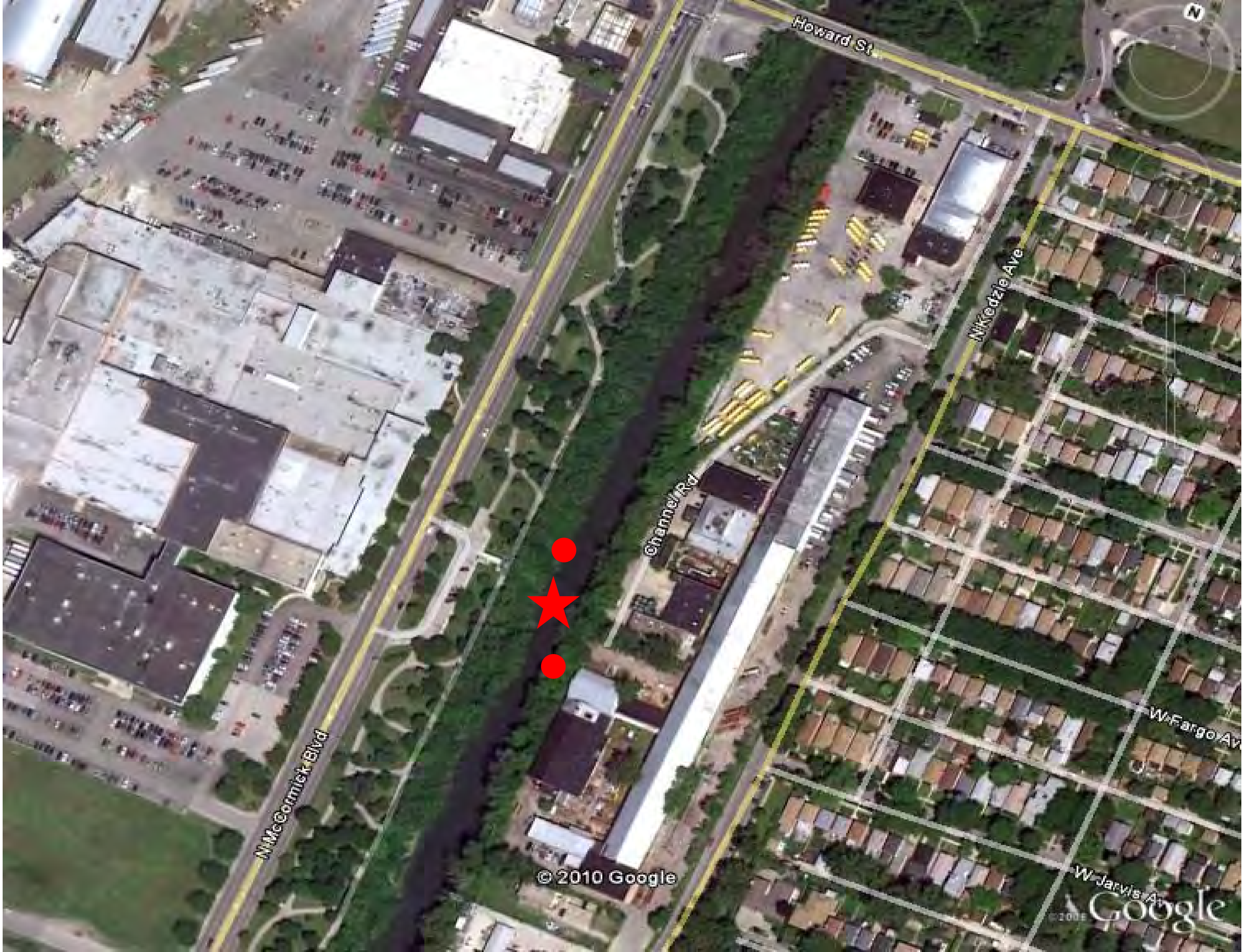

Howard St
N Kedzie Ave
Channel Rd
W Fargo Ave
W Jarvis Ave
N McCormick Blvd
© 2010 Google
Google

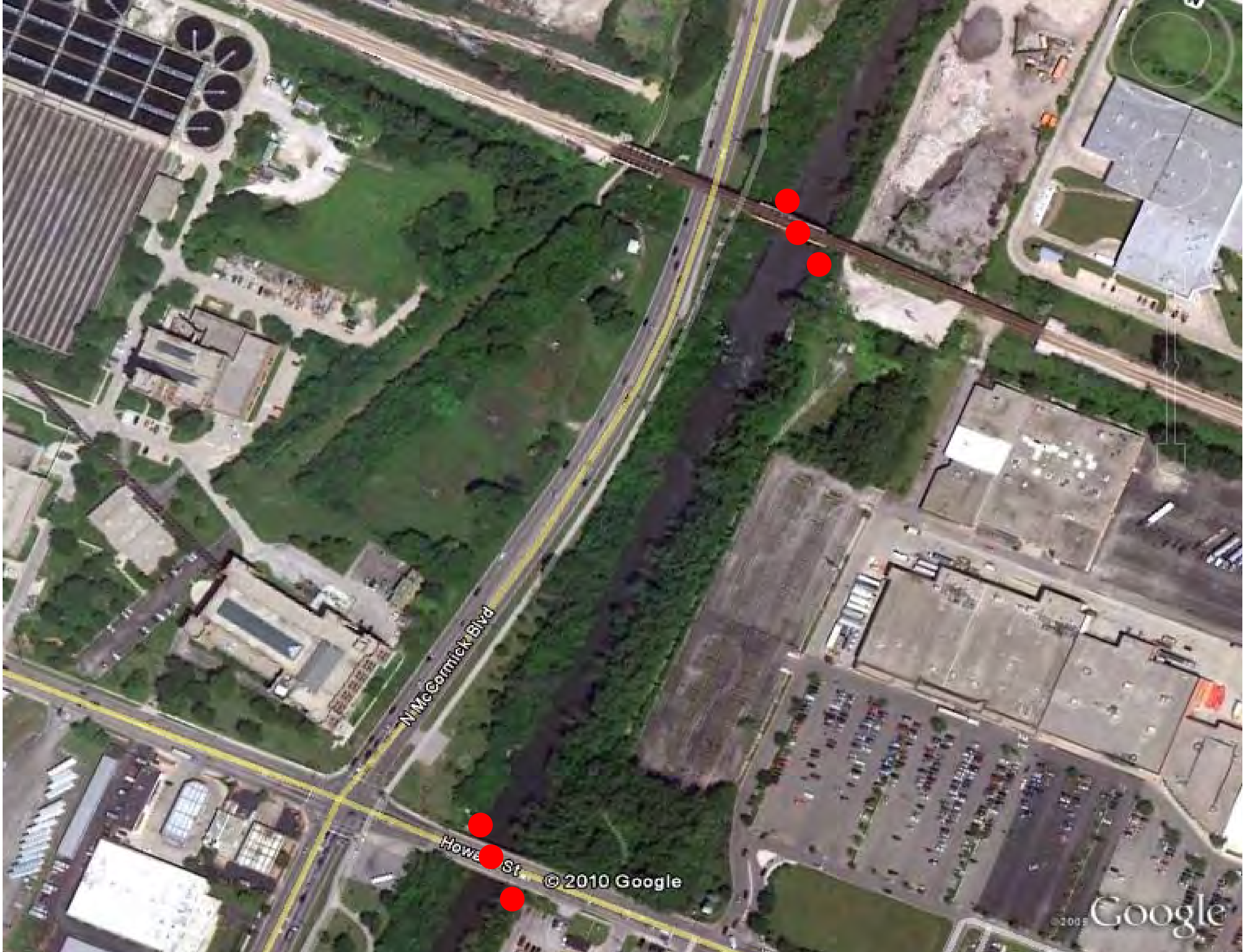
N McCormick Blvd
Howe St
© 2010 Google
Google

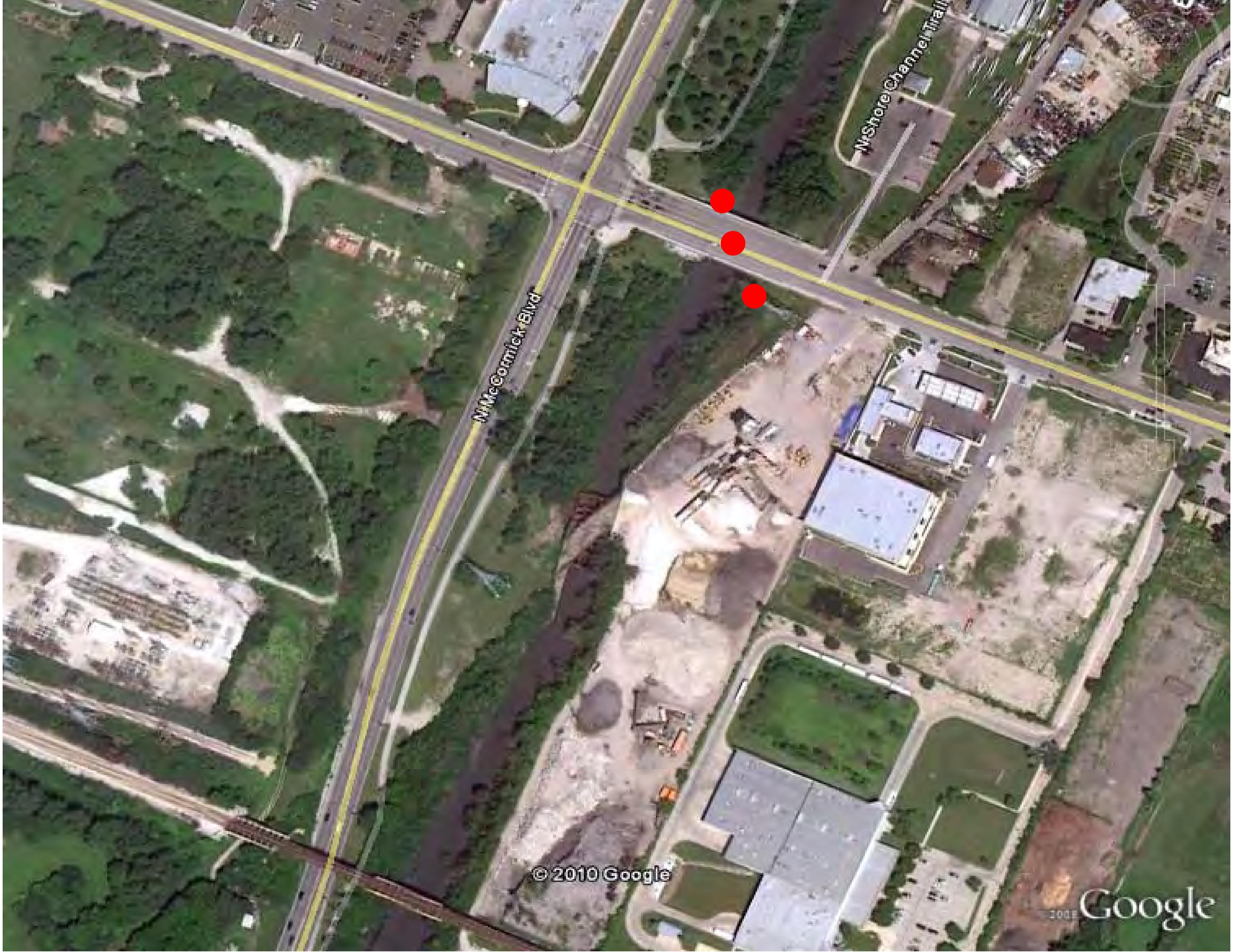
N Shore Channel Trail
N McCormick Blvd
© 2010 Google
Google

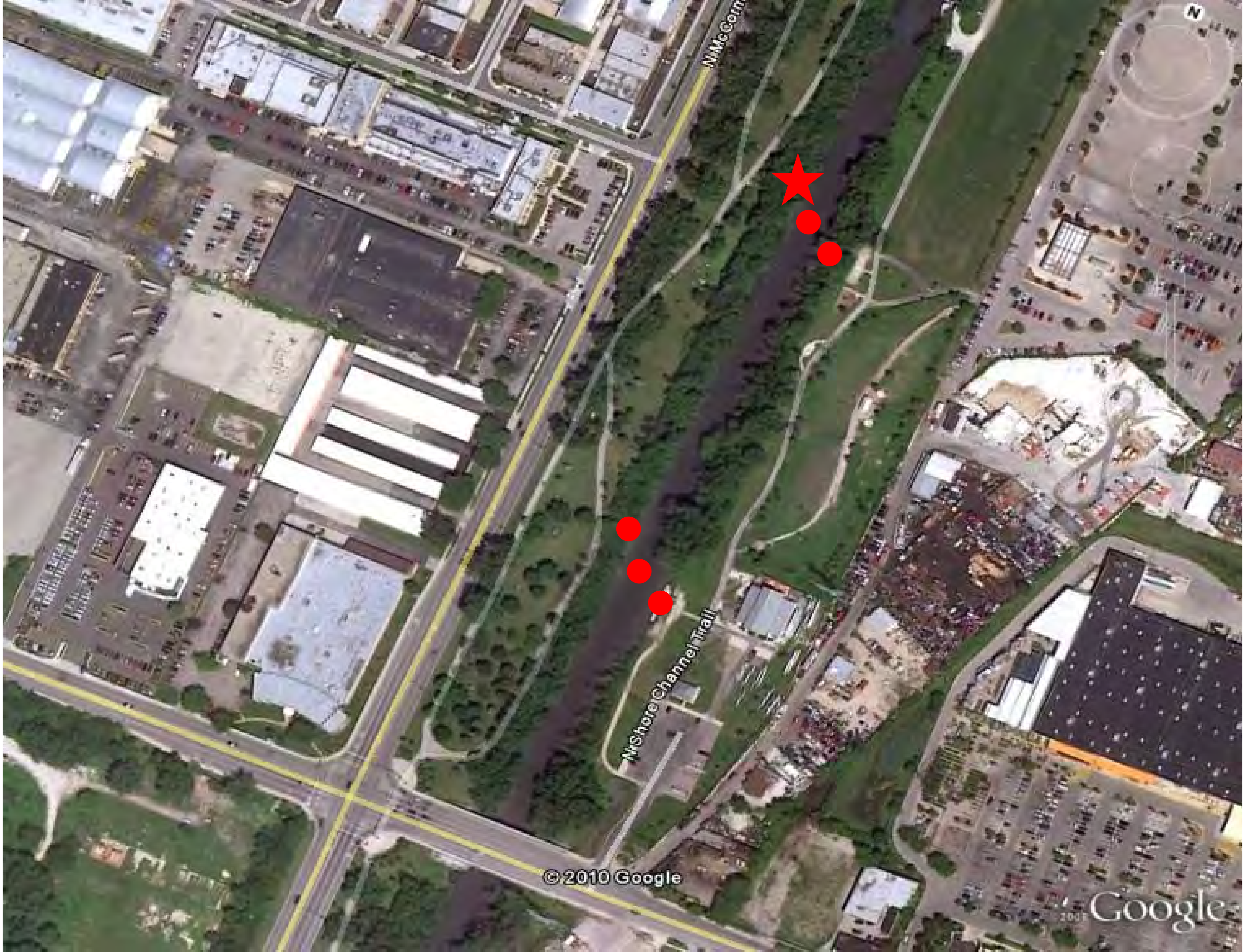
N Shore Channel Trail
© 2010 Google
Google

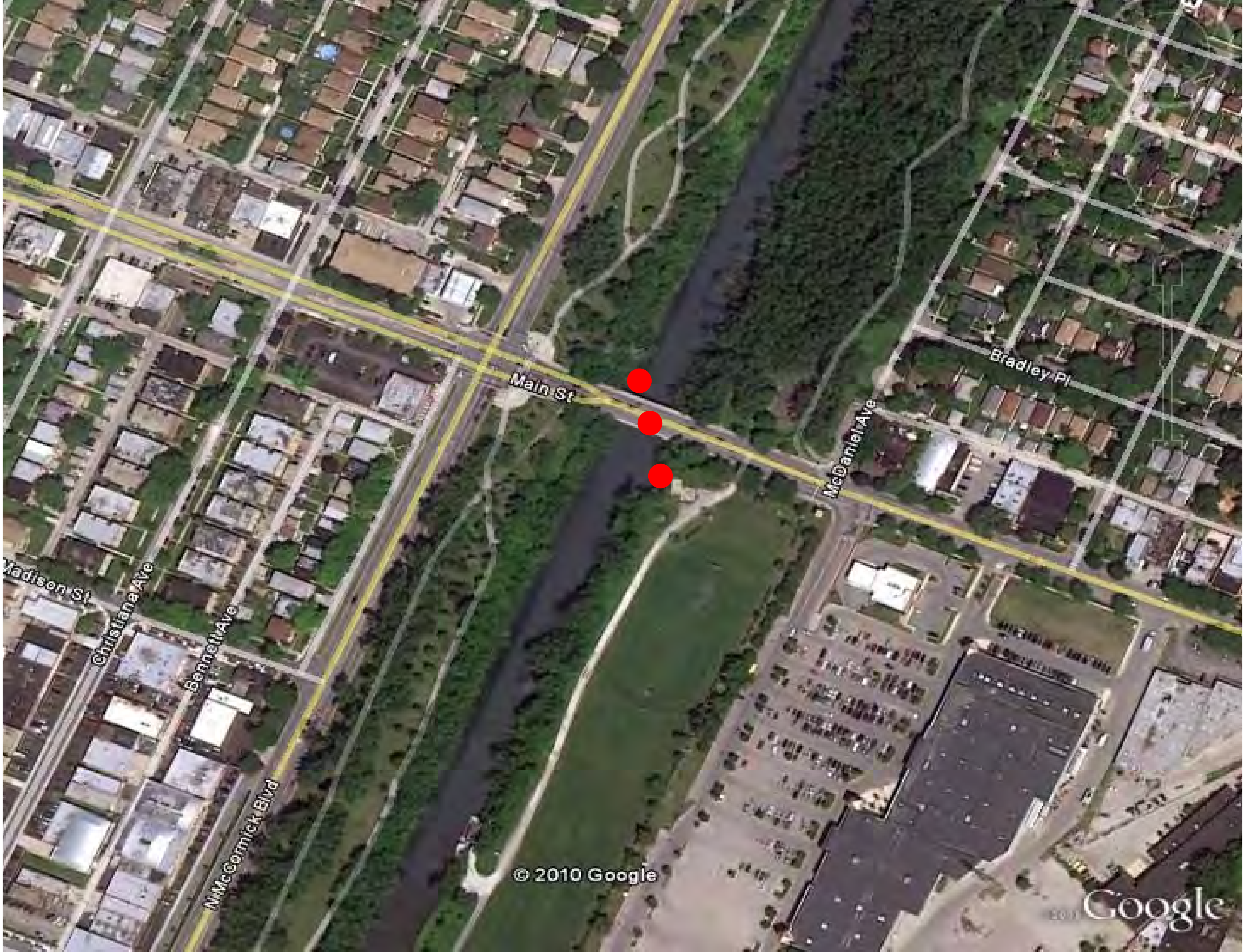
Main St
Bradley Pl
McDaniel Ave
Madison St
Christiana Ave
Bennett Ave
N McCormick Blvd
© 2010 Google
Google

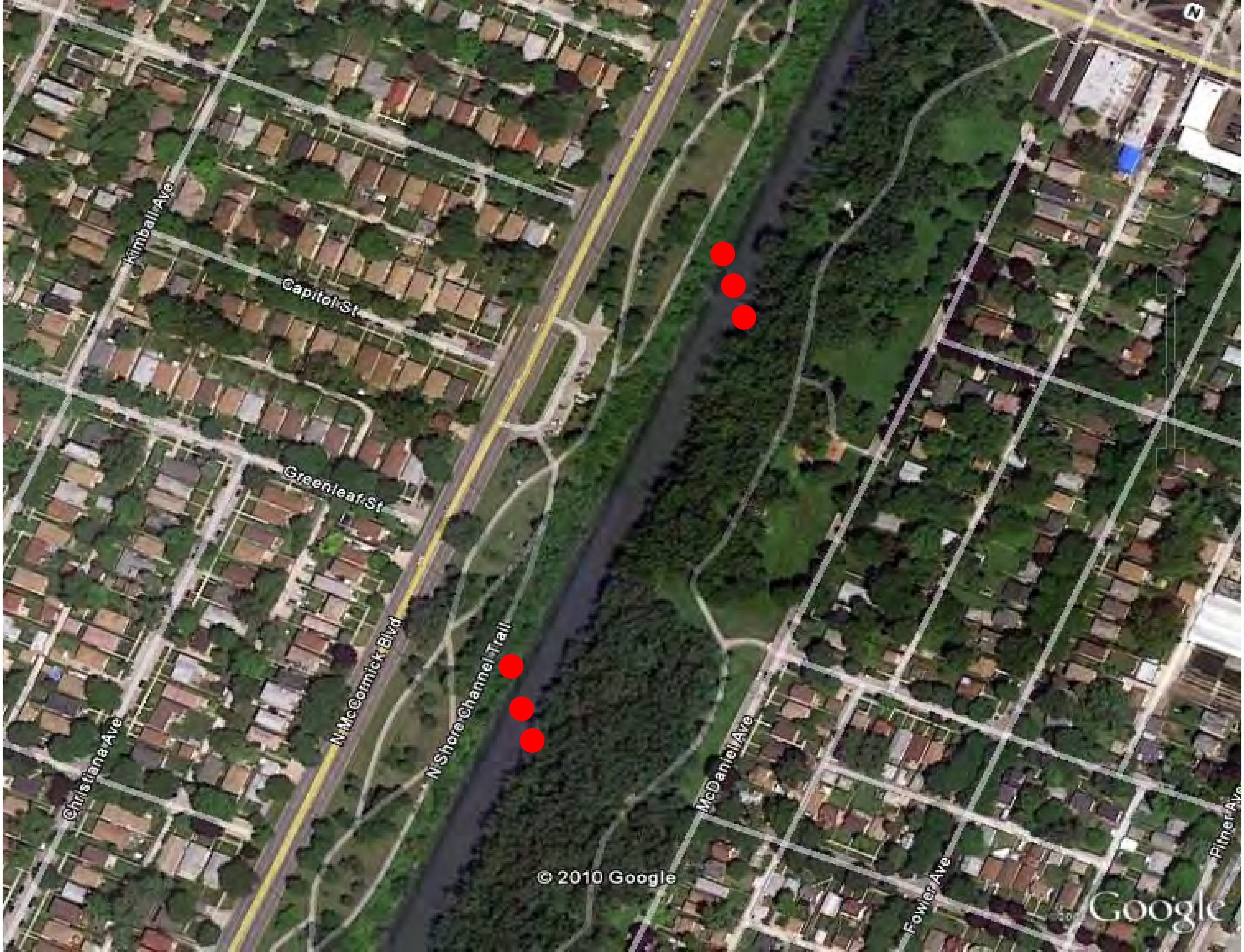

Kimball Ave
Capitol St
Greenleaf St
N McCormick Blvd
N Shore Channel Trail
Christiana Ave
McDaniel Ave
Fowler Ave
Pitner Ave
© 2010 Google
Google

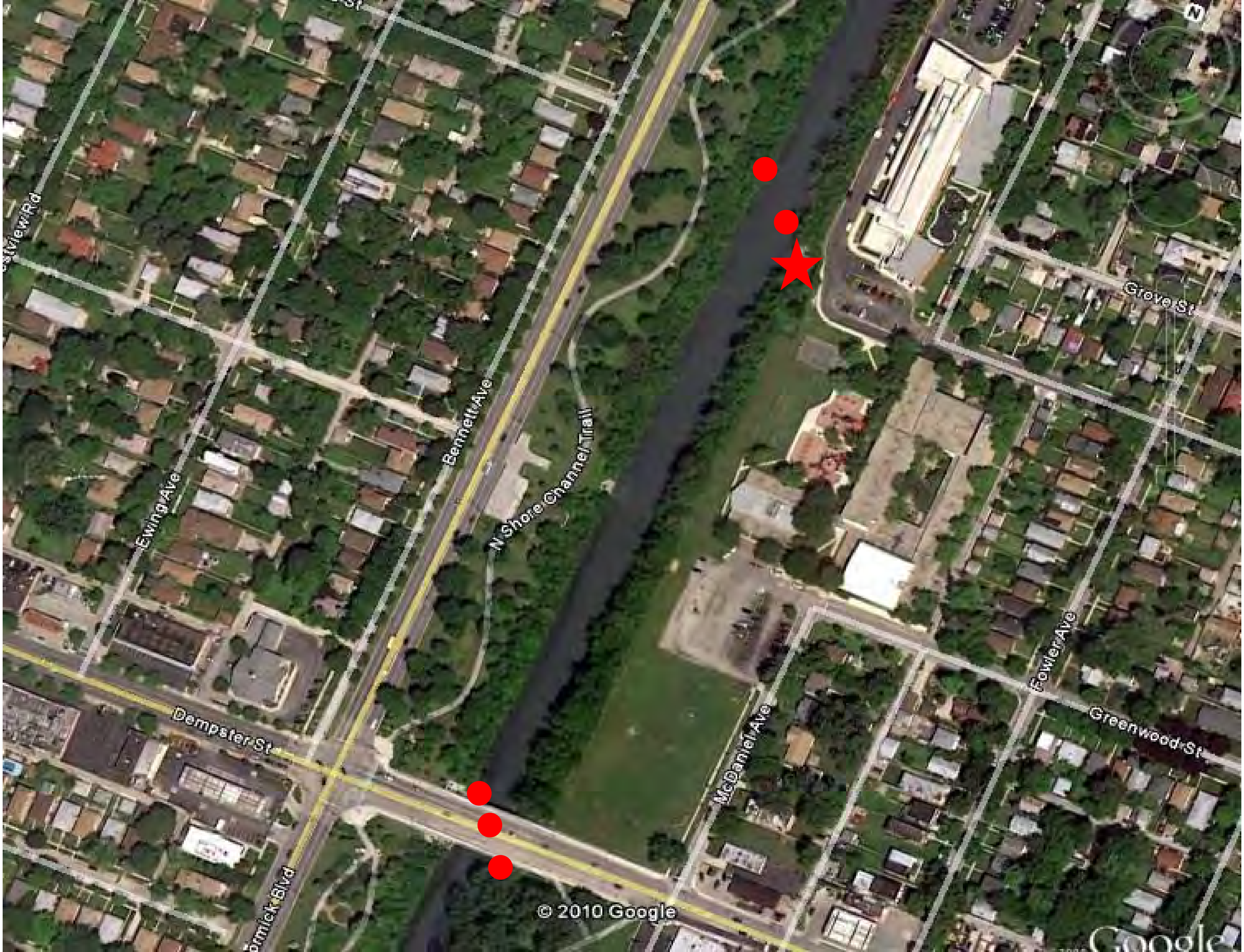

Bennett Ave
N Shore Channel Trail
Ewing Ave
Grove St
Dempster St
McDaniel Ave
Fowler Ave
Greenwood St
© 2010 Google

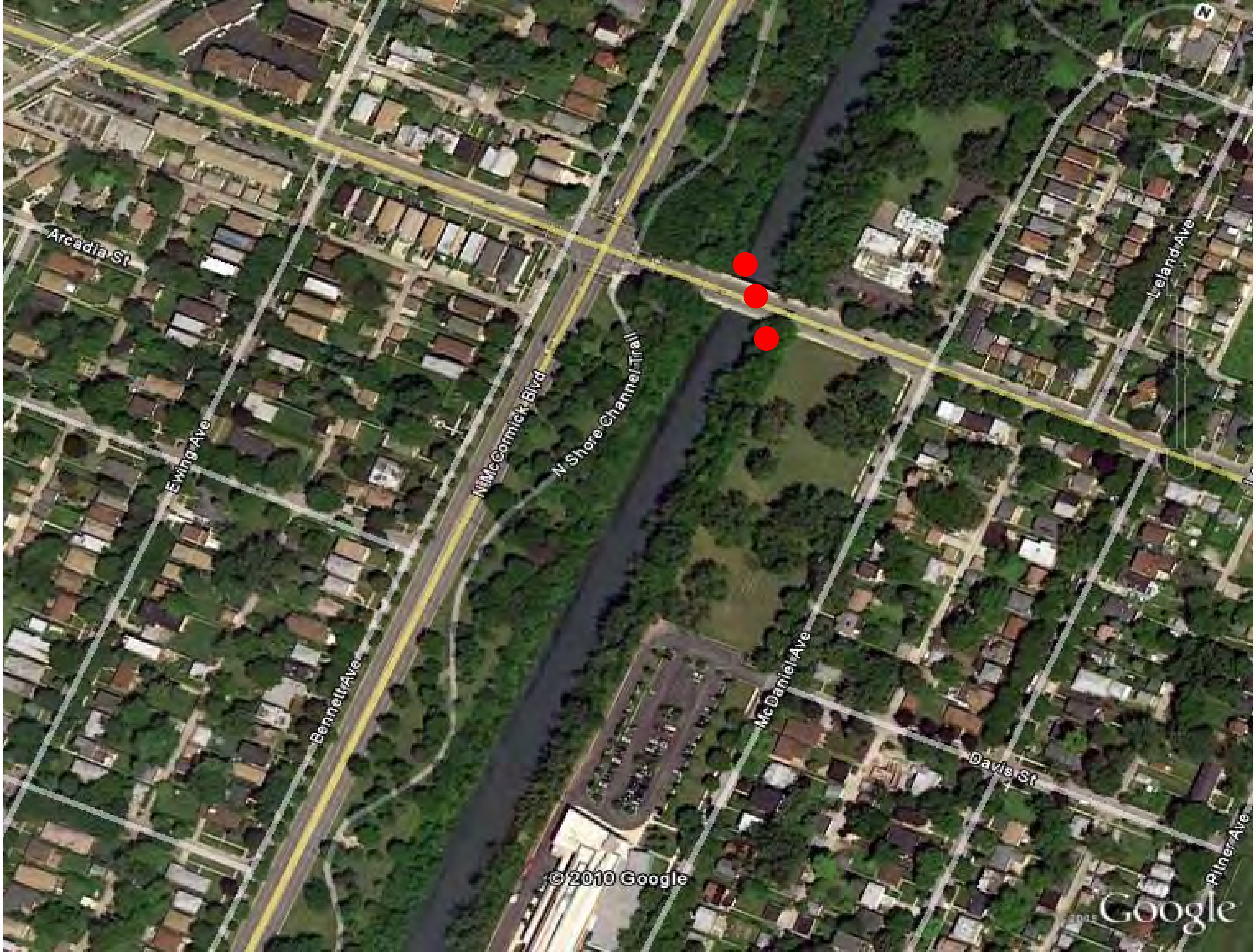
Arcadia St
Ewing Ave
N McCormick Blvd
N Shore Channel Trail
Bennett Ave
McDaniel Ave
Leland Ave
Davis St
Pitner Ave
© 2010 Google
Google

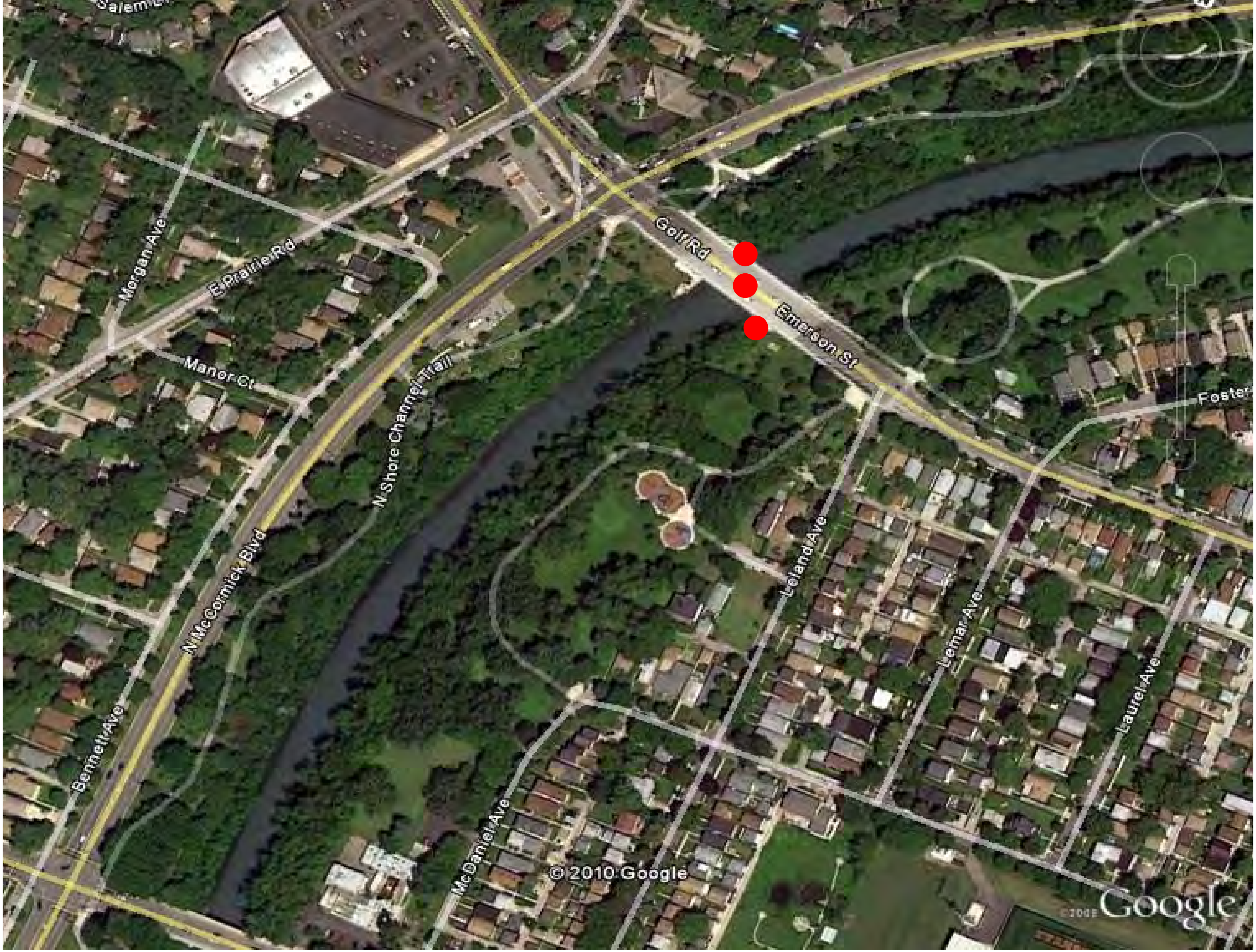
Golf Rd
Emerson St
E Prairie Rd
Morgan Ave
Manor Ct
N Shore Channel Trail
N McCormick Blvd
Bennett Ave
Leland Ave
Lemar Ave
Laurel Ave
McDaniel Ave
Foster
© 2010 Google
Google

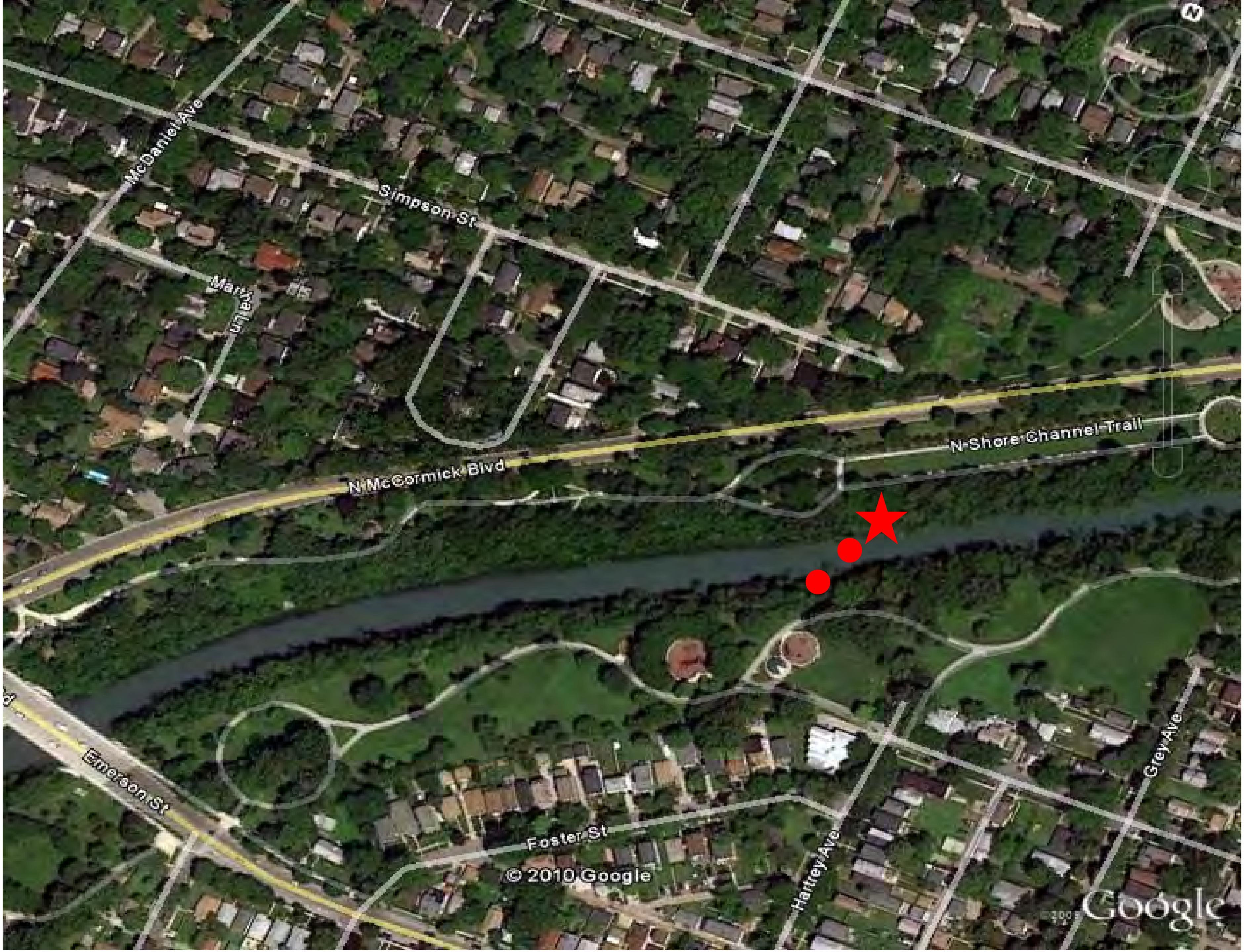

McDaniel Ave
Simpson St
Marcy Ln
N McCormick Blvd
N Shore Channel Trail
Emerson St
Foster St
Hartrey Ave
Grey Ave
© 2010 Google
Google

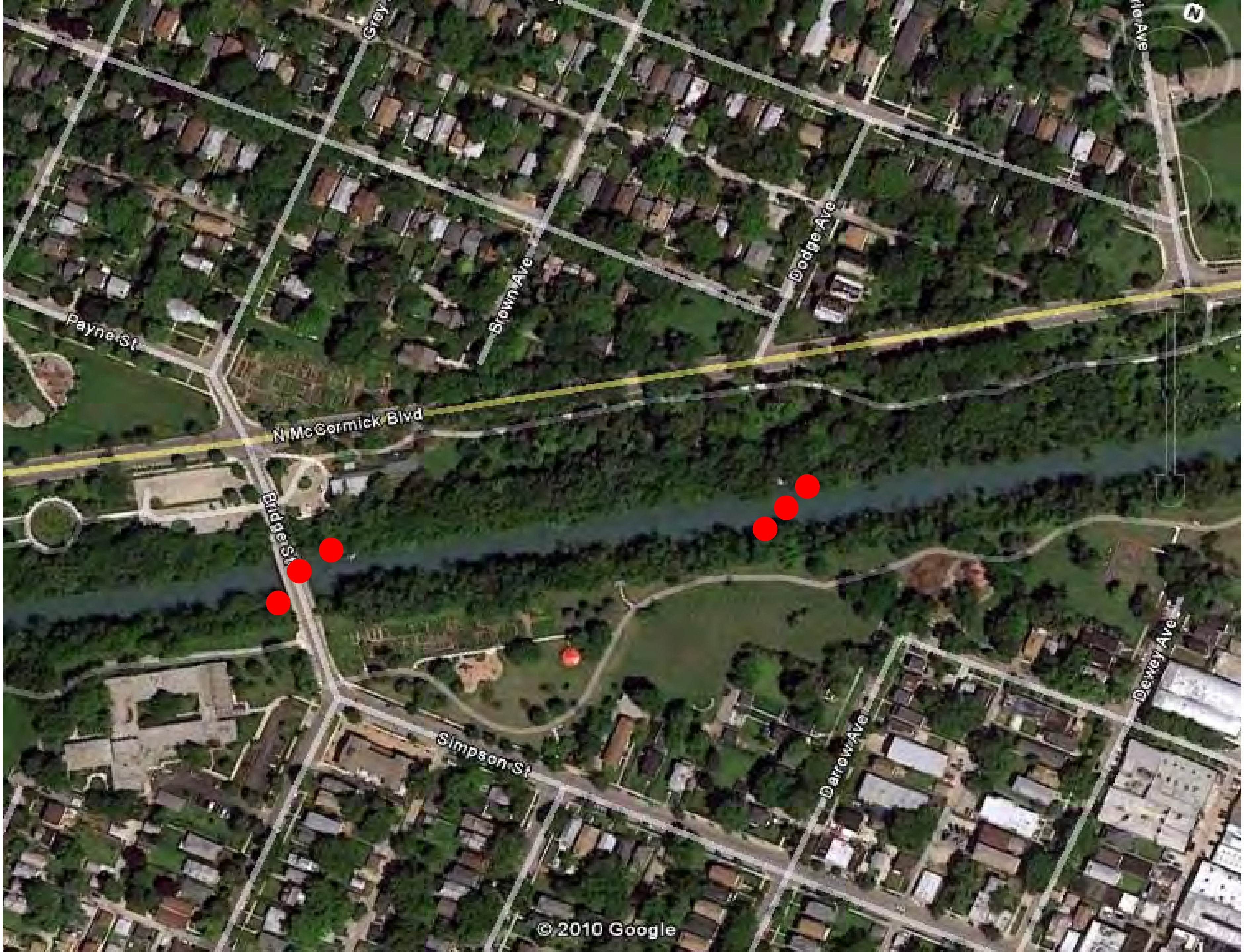

Payne St
N McCormick Blvd
Brown Ave
Dodge Ave
Bridge St
Simpson St
Darrow Ave
Dewey Ave
© 2010 Google

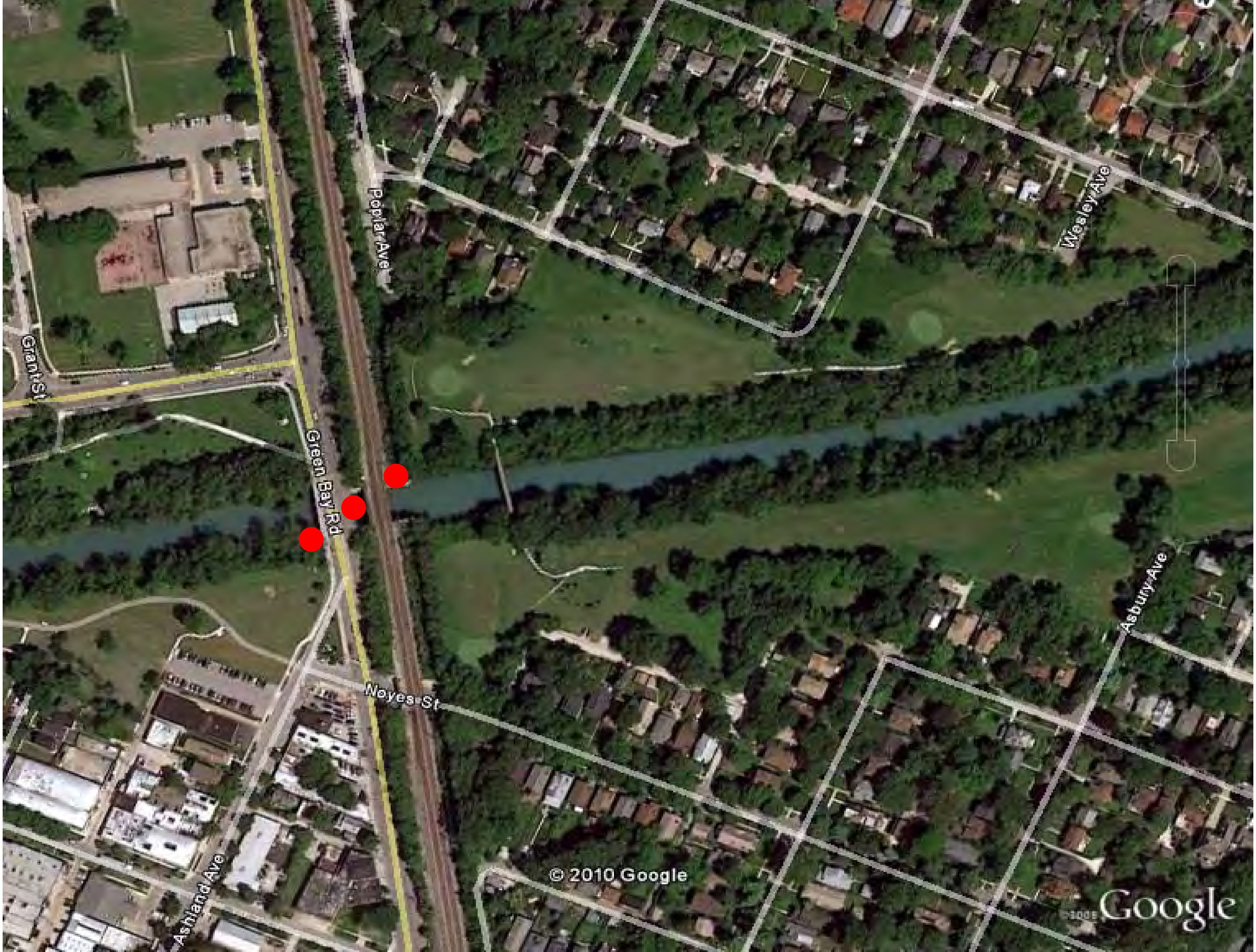
Poplar Ave
Wesley Ave
Grant St
Green Bay Rd
Asbury Ave
Noyes St
Ashland Ave
© 2010 Google
Google

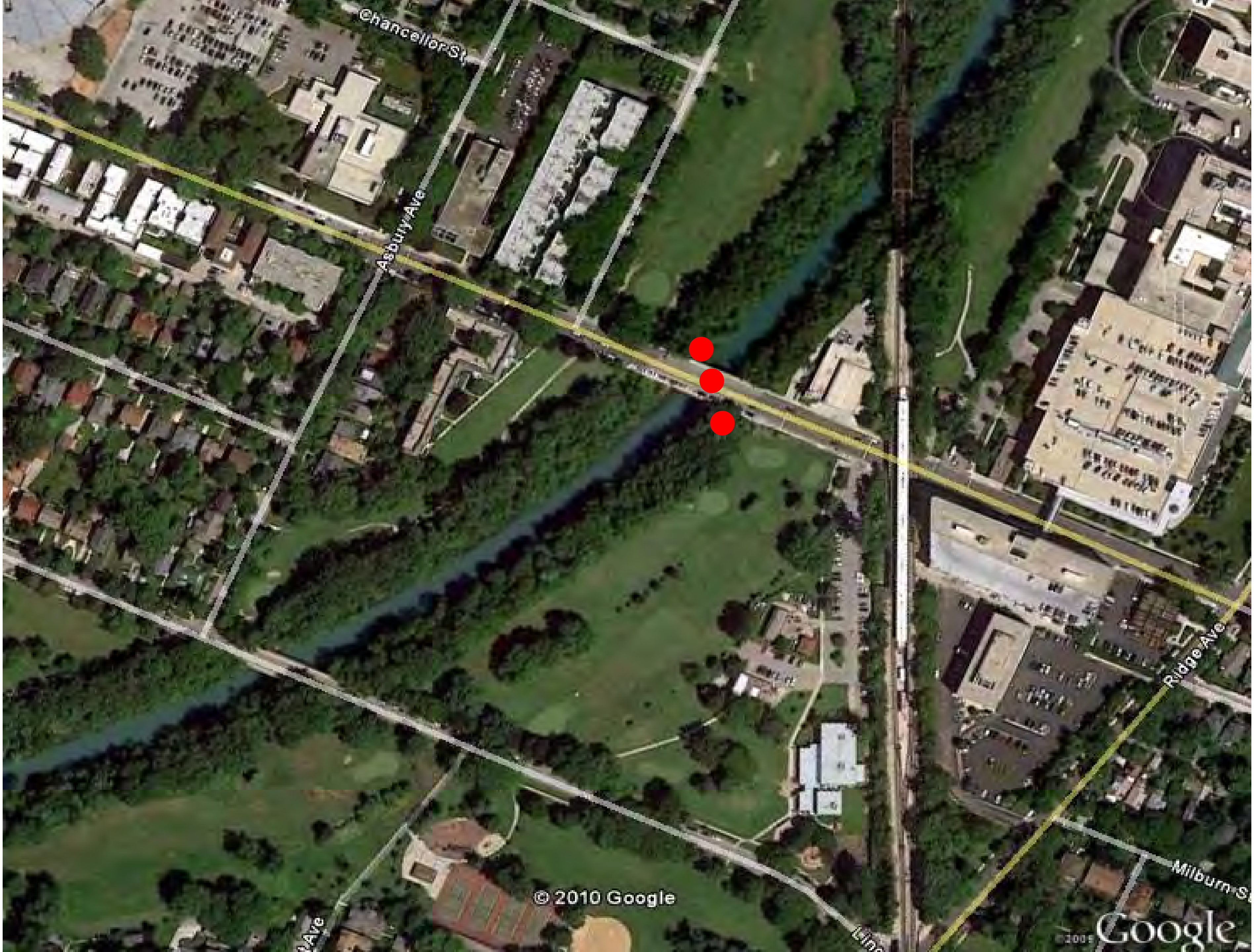
Chancellor St
Asbury Ave
Ridge Ave
Milburn St
© 2010 Google
Google

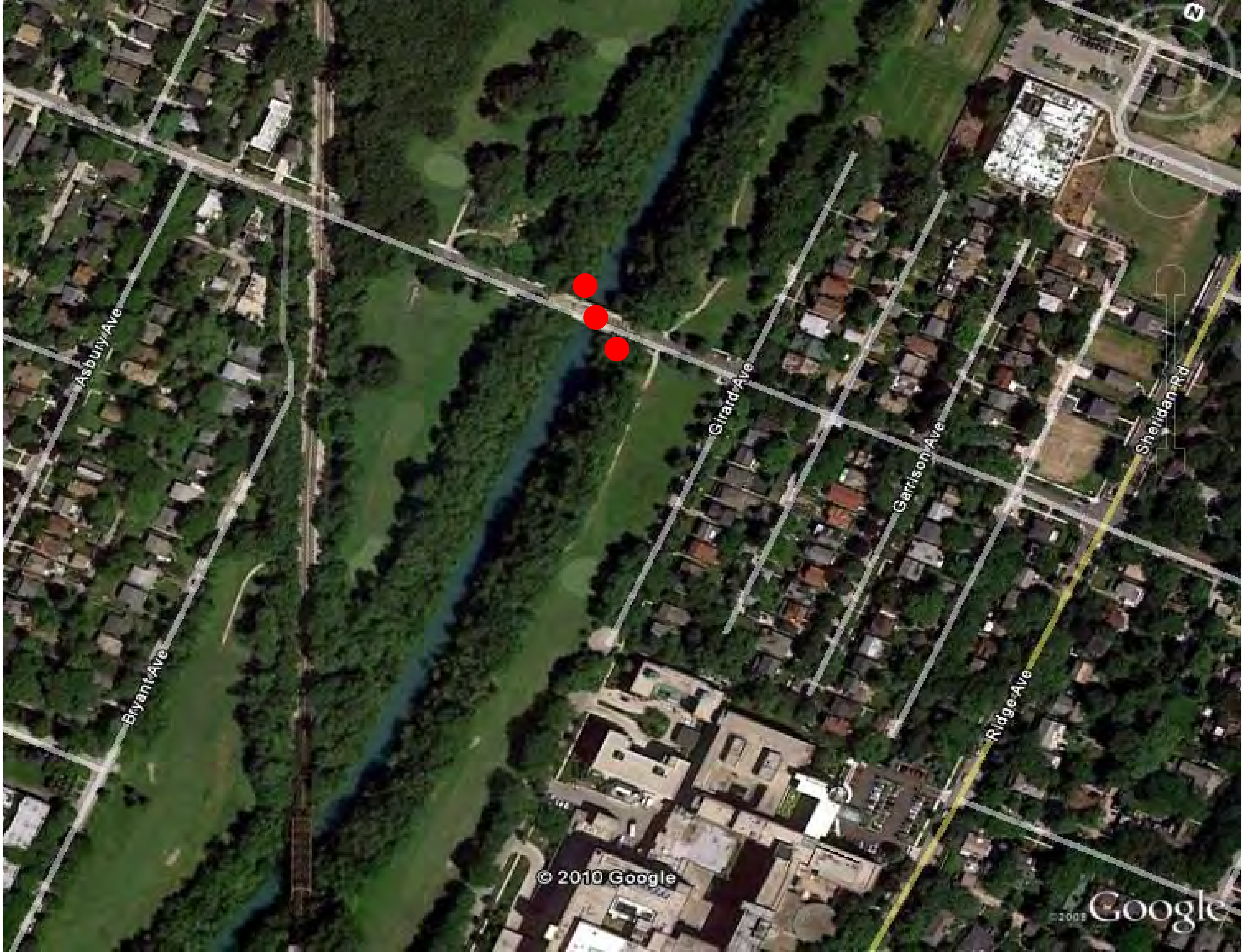
Asbury Ave
Bryant Ave
Girard Ave
Garrison Ave
Sheridan Rd
Ridge Ave
© 2010 Google
Google

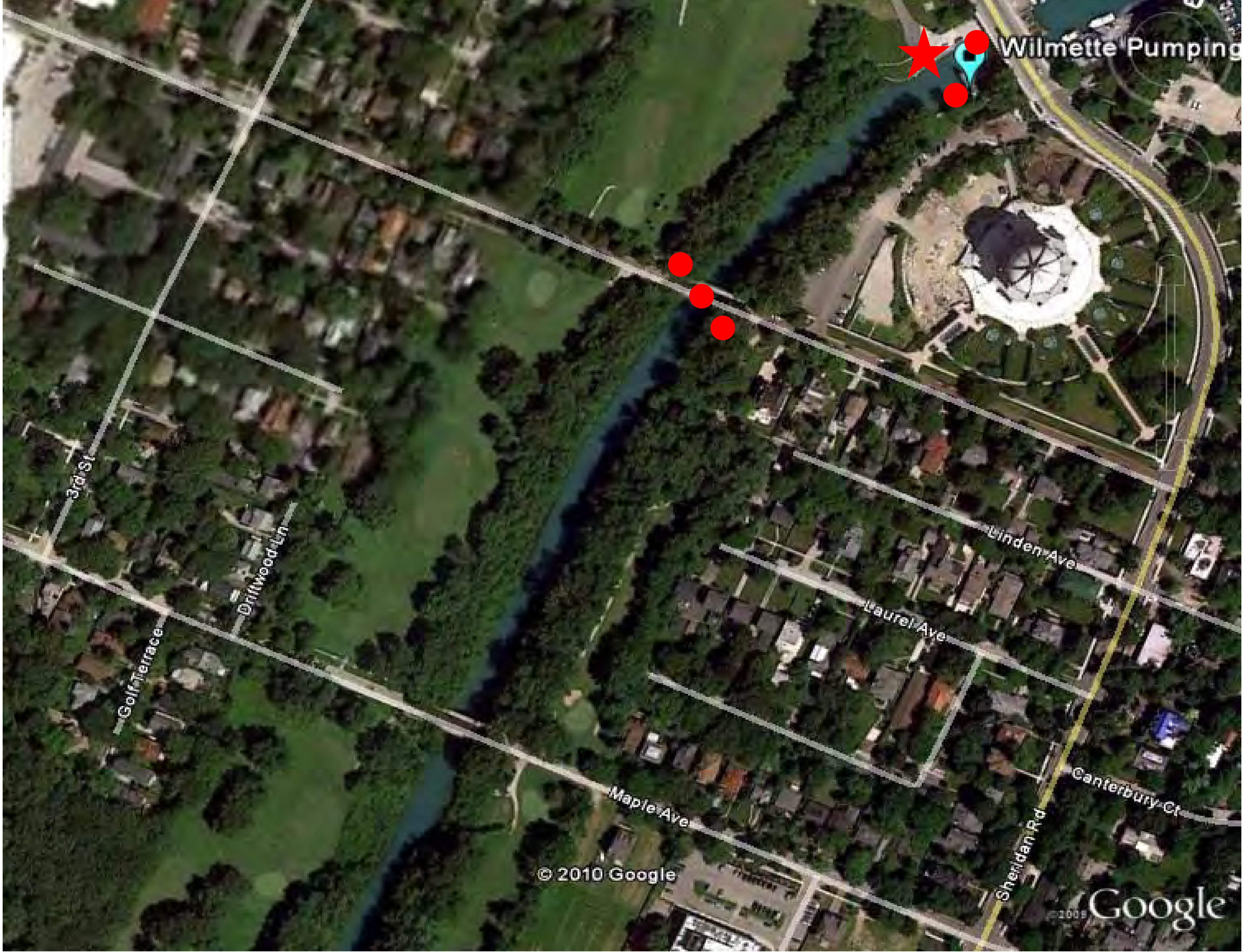
Wilmette Pumping
3rd St
Driftwood Ln
Golf Terrace
Linden Ave
Laurel Ave
Canterbury Ct
Maple Ave
Sheridan Rd
© 2010 Google
Google

Chicago Lock to Bubbly Creek

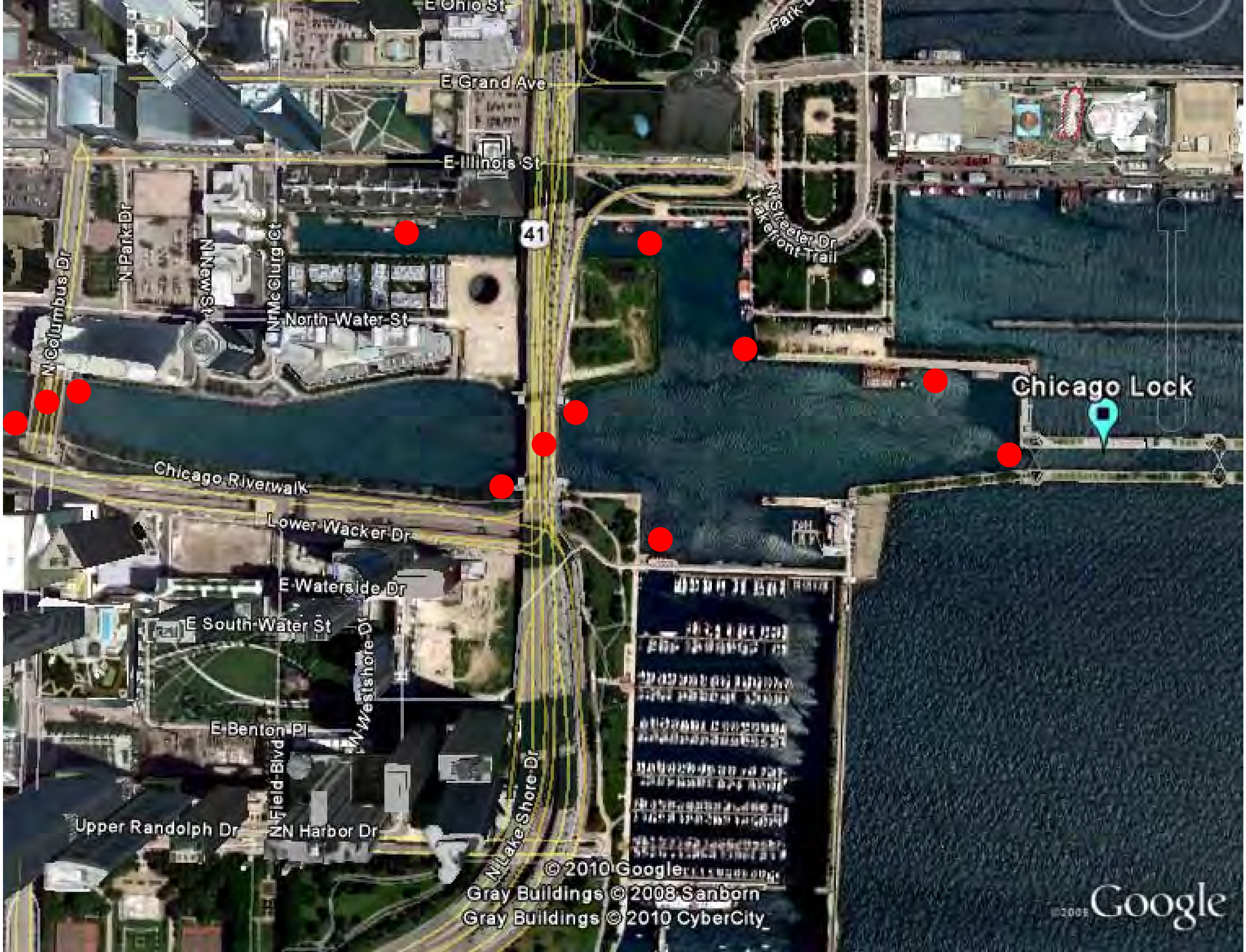
E Ohio St
E Grand Ave
E Illinois St
41
N Columbus Dr
N Park Dr
N New St
N McClurg Ct
North Water St
N Streeter Dr
Lakefront Trail
Chicago Lock
Chicago Riverwalk
Lower Wacker Dr
E Waterside Dr
E South Water St
N Westshore Dr
E Benton Pl
N Field Blvd
Upper Randolph Dr
N Harbor Dr
N Lake Shore Dr
© 2010 Google
Gray Buildings © 2008 Sanborn
Gray Buildings © 2010 CyberCity
Google

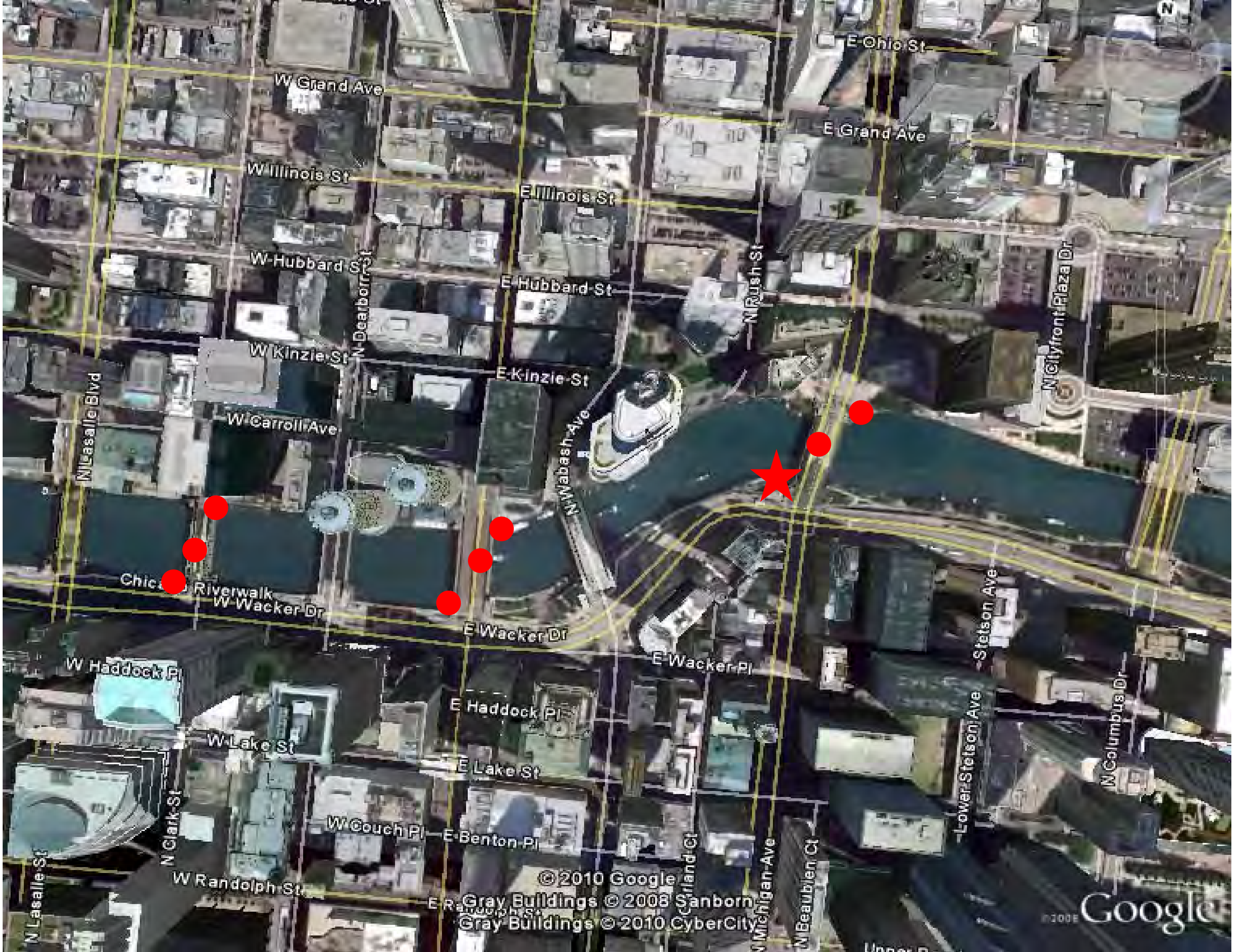

E Ohio St
W Grand Ave
E Grand Ave
W Illinois St
E Illinois St
W Hubbard St
E Hubbard St
N Dearborn St
N Rush St
N Cityfront Plaza Dr
W Kinzie St
E Kinzie St
N Lasalle Blvd
W Carroll Ave
N Wabash Ave
Riverwalk
W Wacker Dr
E Wacker Dr
E Wacker Pl
Stetson Ave
W Haddock Pl
E Haddock Pl
W Lake St
E Lake St
Lower Stetson Ave
N Columbus Dr
N Clark St
W Couch Pl
E Benton Pl
N Michigan Ave
N Beaubien Ct
W Randolph St
N Lasalle St
© 2010 Google
Gray Buildings © 2008 Sanborn
Gray Buildings © 2010 CyberCity
Google

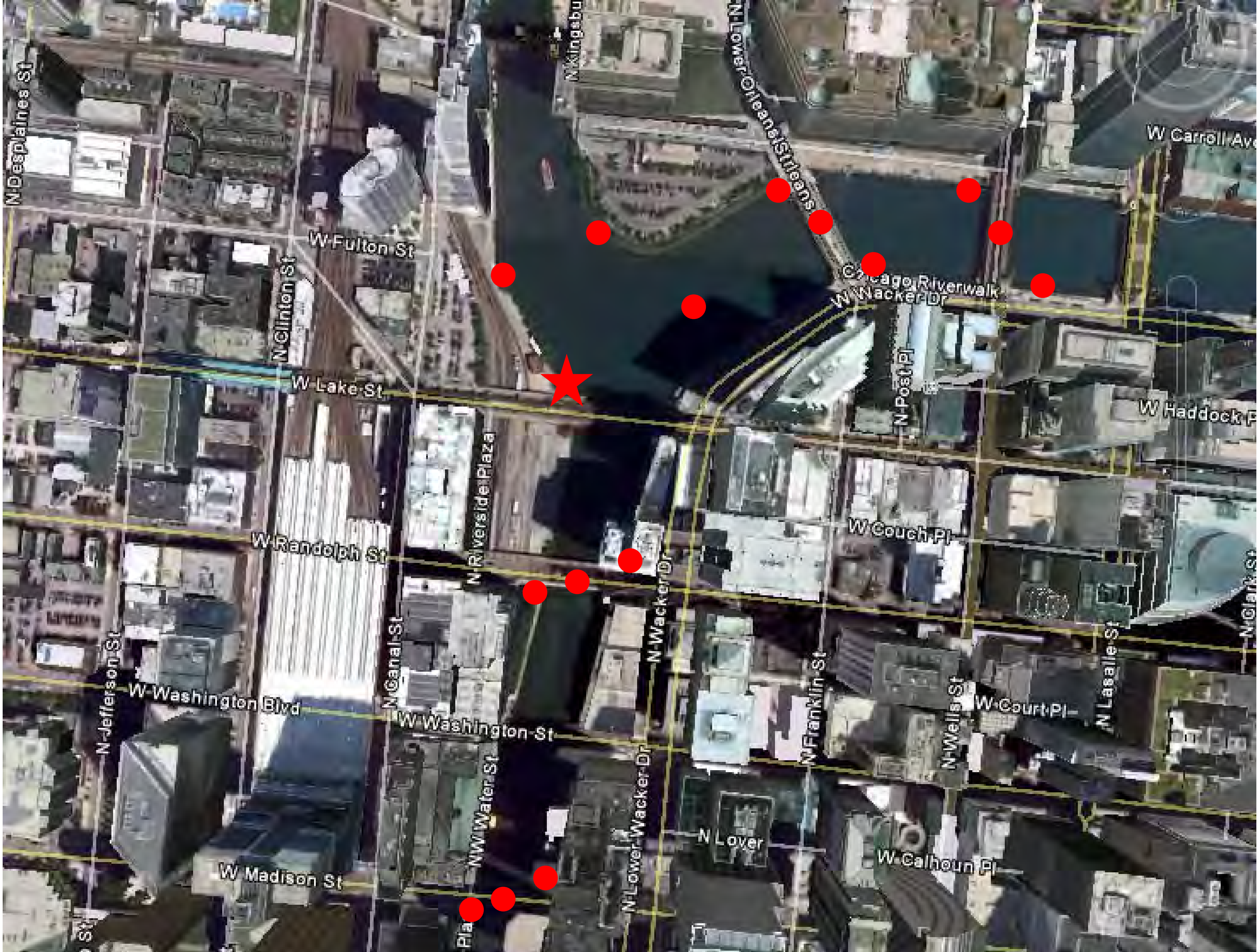
N Desplaines St
W Fulton St
N Clinton St
N Kingsbury
N Lower Orleans St
W Carroll Ave
Chicago Riverwalk
W Wacker Dr
W Lake St
N Post Pl
W Haddock Pl
N Riverside Plaza
W Randolph St
W Couch Pl
N Wacker Dr
N Clark St
N Canal St
N Jefferson St
W Washington Blvd
W Washington St
N Franklin St
N Wells St
W Court Pl
N Lasalle St
NW Water St
N Lower Wacker Dr
N Lower
W Calhoun Pl
W Madison St

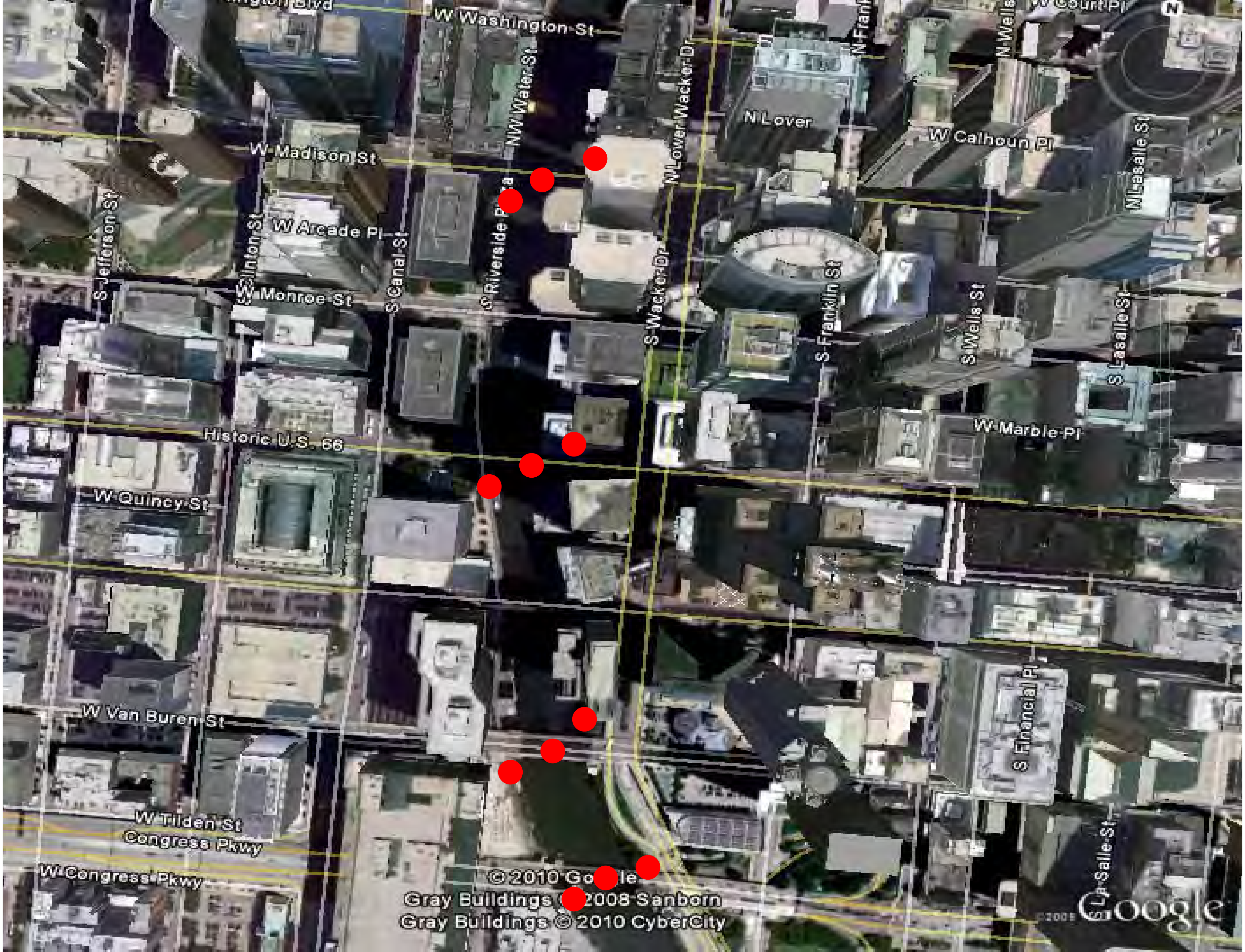
W Washington St
NW Water St
N Lower Wacker Dr
N Lover
W Calhoun Pl
W Madison St
W Arcade Pl
S Jefferson St
S Clinton St
S Canal St
W Monroe St
S Wacker Dr
S Franklin St
S Wells St
N Lasalle St
S Lasalle St
W Marble Pl
Historic U.S. 66
W Quincy St
W Van Buren St
S Financial Pl
W Tilden St
Congress Pkwy
W Congress Pkwy
Gray Buildings © 2010 CyberCity
Google

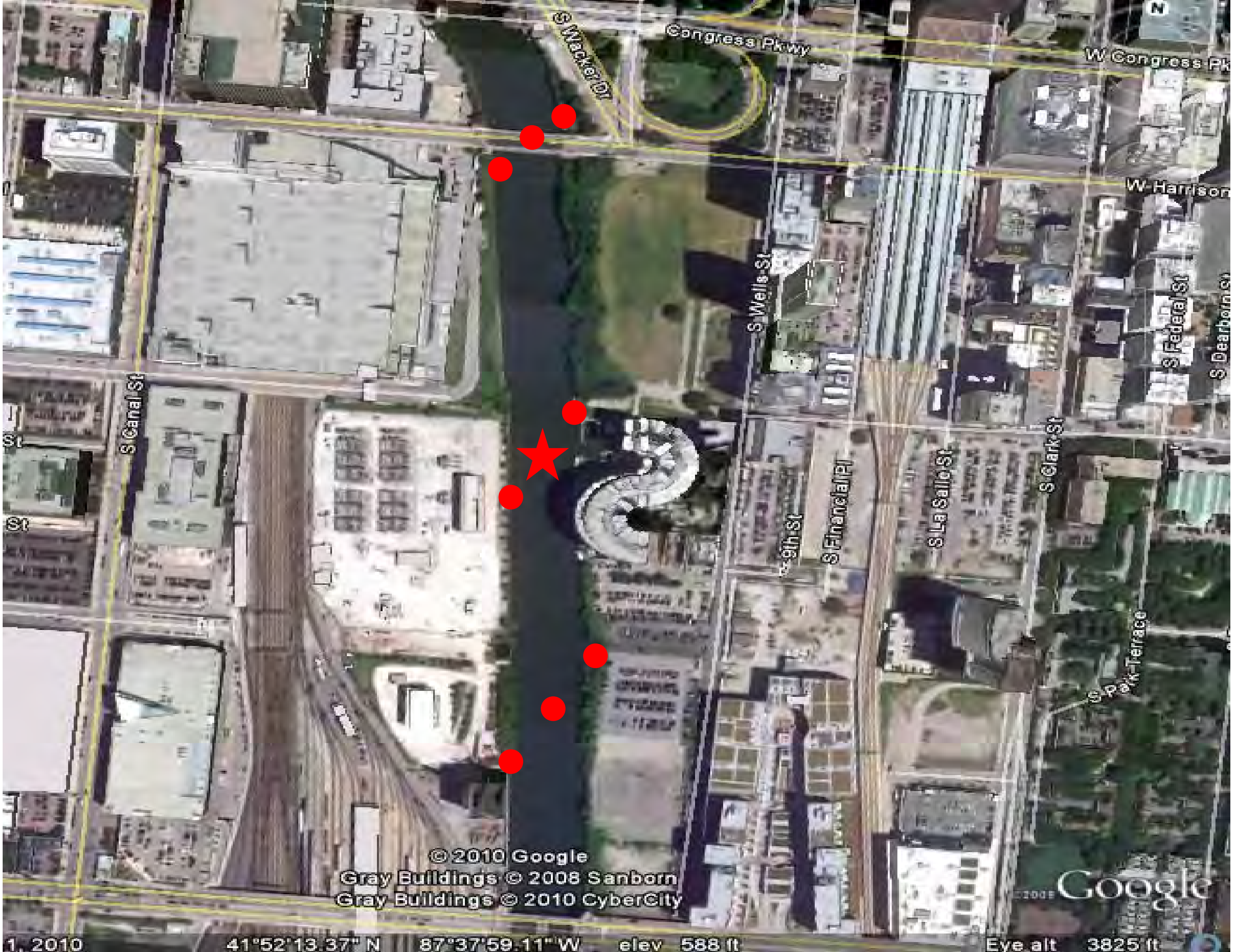
S Wacker Dr
Congress Pkwy
W Congress Pk
W Harrison
S Wells St
S Federal St
S Dearborn St
S Canal St
S Clark St
S La Salle St
S Financial Pl
9th St
S Park Terrace
© 2010 Google
Gray Buildings © 2008 Sanborn
Gray Buildings © 2010 CyberCity
Google
1, 2010
41°52'13.37" N
87°37'59.11" W
elev 588 ft
Eye alt 3825 ft

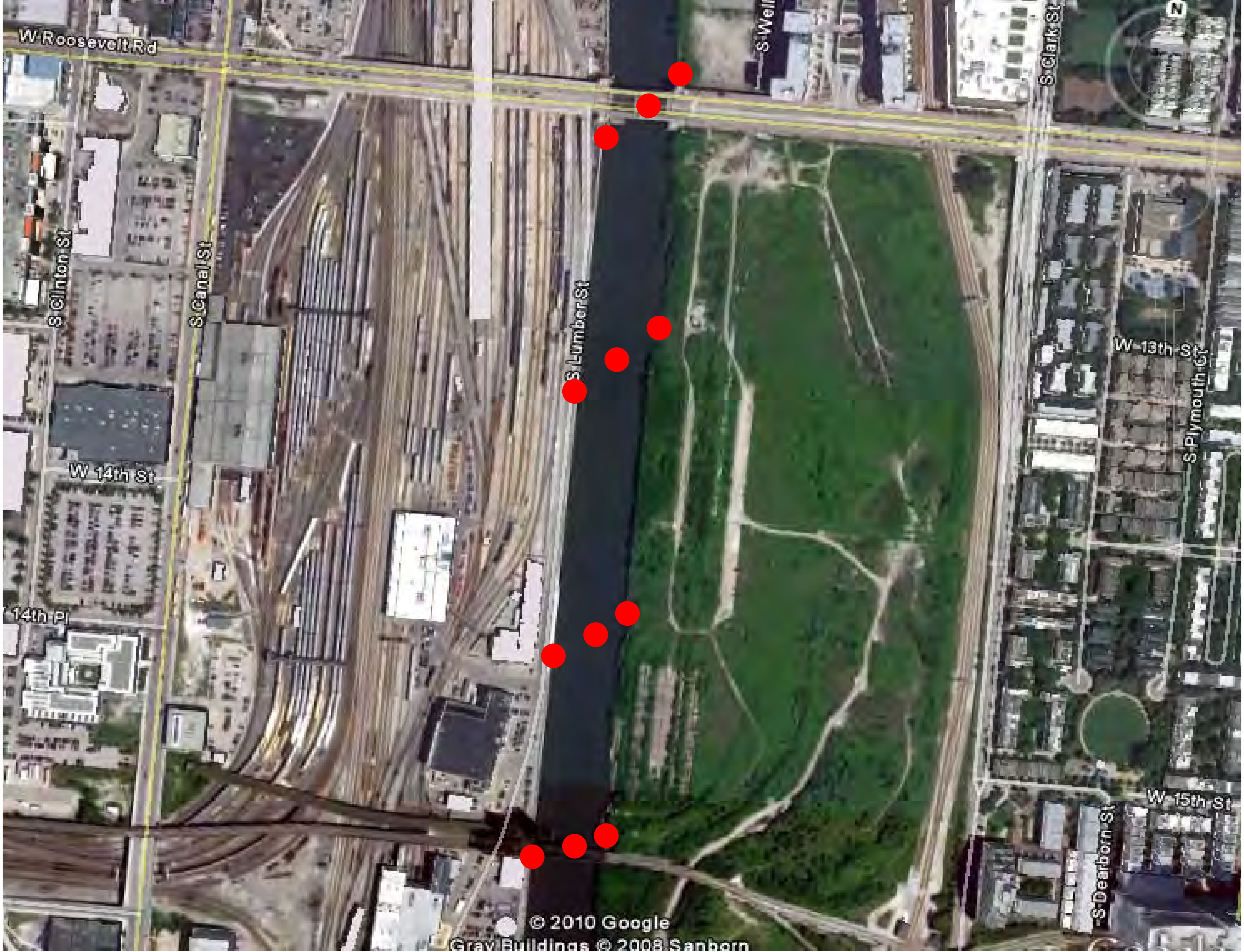

W Roosevelt Rd
S Wells
S Clark St
S Clinton St
S Canal St
S Lumber St
W 13th St
S Plymouth Ct
W 14th St
W 14th Pl
W 15th St
S Dearborn St
© 2010 Google
Gray Buildings © 2008 Sanborn

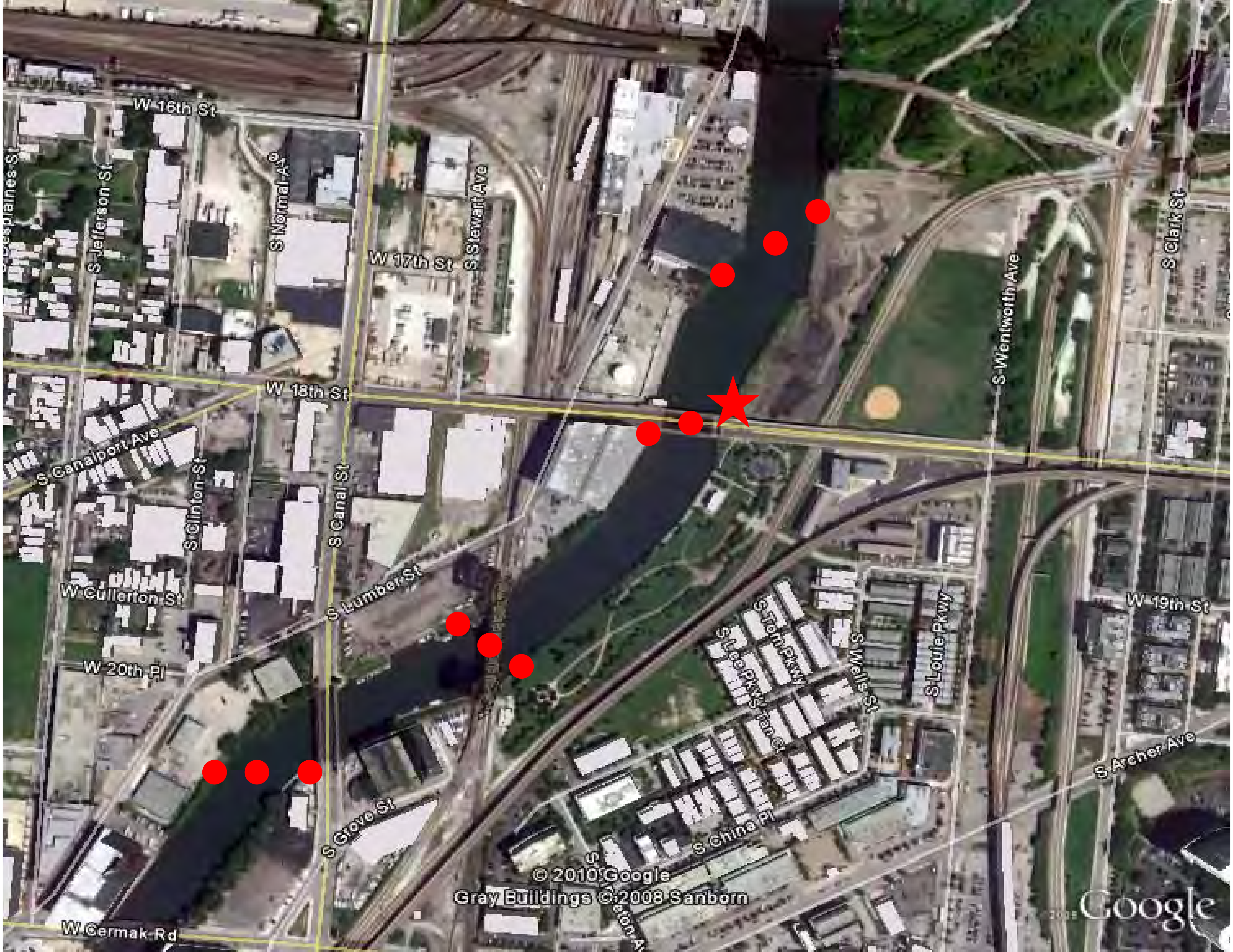
W 16th St
S Desplaines St
S Jefferson St
S Normal Ave
S Stewart Ave
W 17th St
W 18th St
S Canalport Ave
S Clinton St
S Canal St
W Cullerton St
S Lumber St
W 20th Pl
S Grove St
W Cermak Rd
S Wentworth Ave
S Clark St
W 19th St
S Tan Ct
S Lee Pkwy
S Wells St
S Loomis Pkwy
S Archer Ave
S China Pl
© 2010 Google
Gray Buildings © 2008 Sanborn
Google

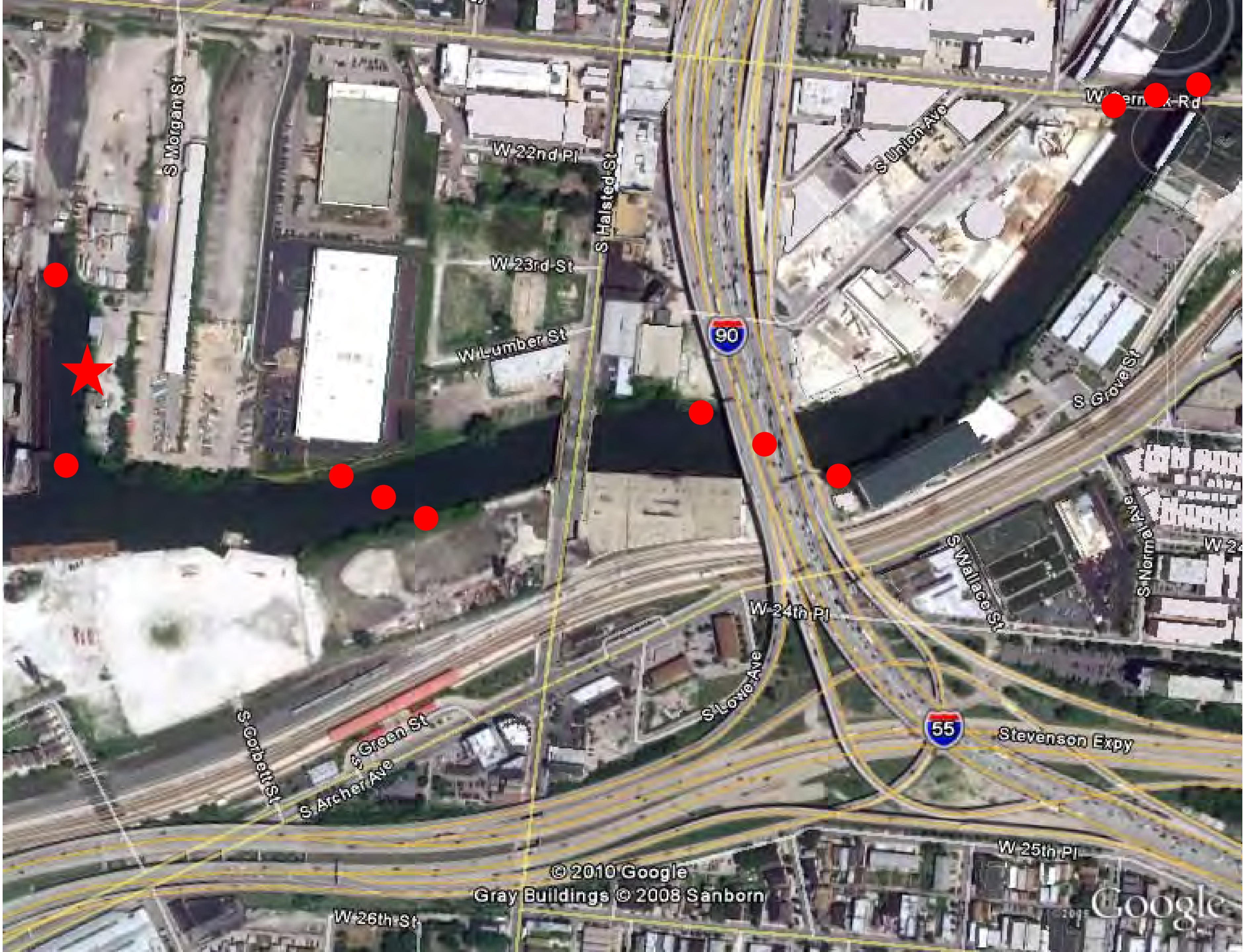

S Morgan St
W 22nd Pl
S Halsted St
W 23rd St
W Lumber St
S Union Ave
S Grove St
90
S Wallace St
S Normal Ave
W 24th Pl
S Loyle Ave
55
Stevenson Expy
S Corbett St
S Green St
S Archer Ave
W 25th Pl
W 26th St
© 2010 Google
Gray Buildings © 2008 Sanborn
Google

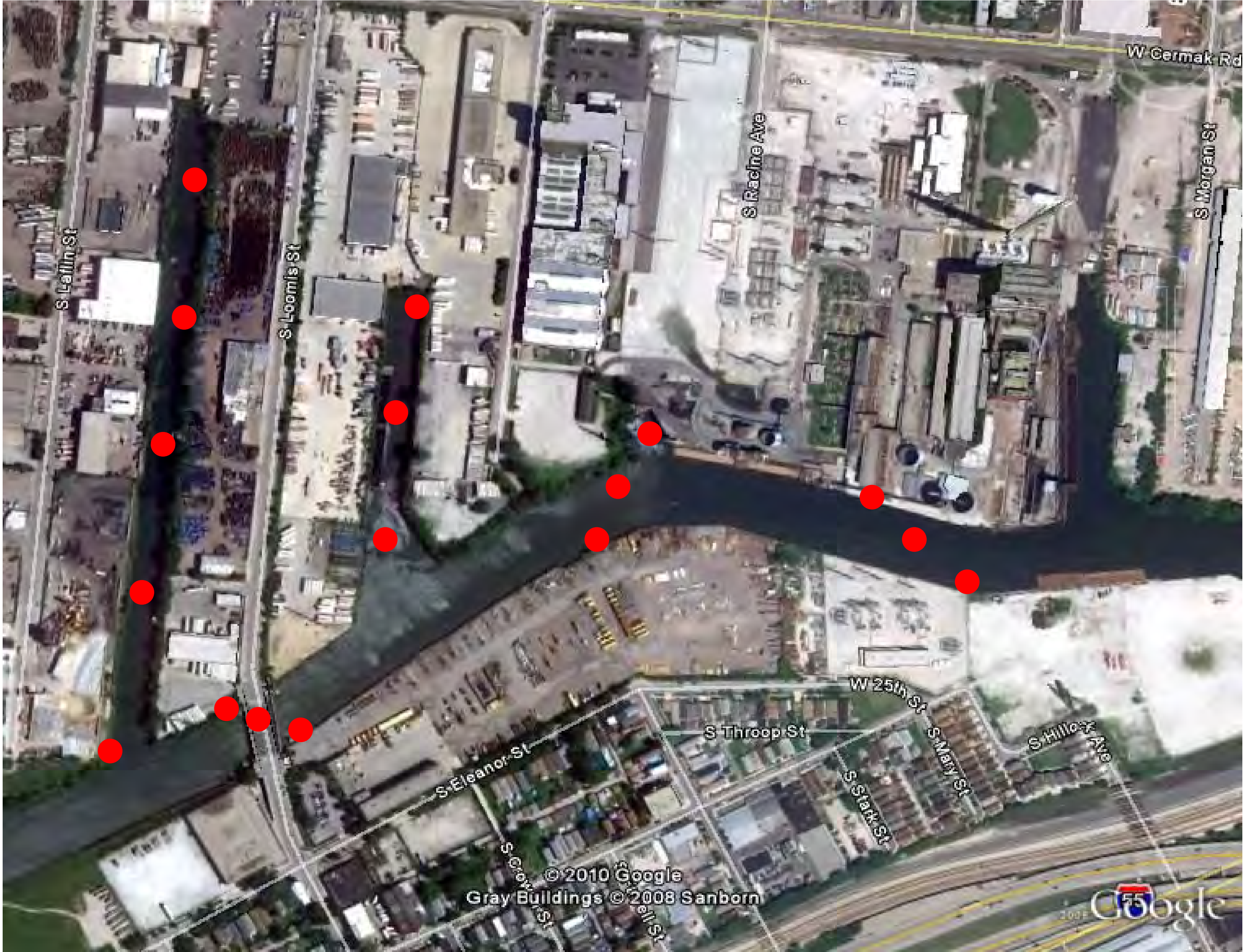
W Cermak Rd
S Laflin St
S Loomis St
S Racine Ave
S Morgan St
W 25th St
S Throop St
S Eleanor St
S Mary St
S Hillock Ave
S Stark St
© 2010 Google
Gray Buildings © 2008 Sanborn
Google

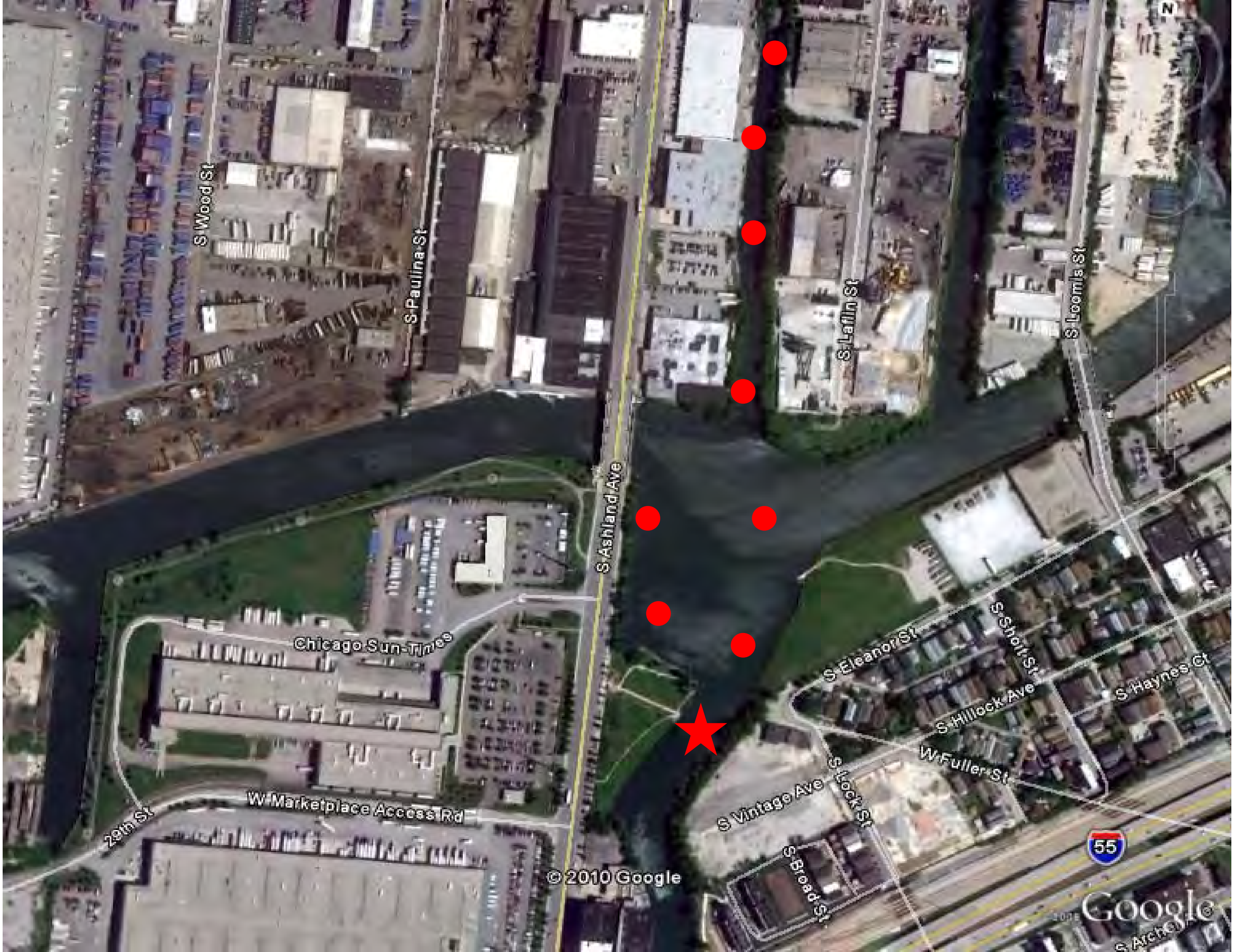

S Wood St
S Paulina St
S Laflin St
S Loomis St
S Ashland Ave
Chicago Sun-Times
S Eleanor St
S Short St
S Haynes Ct
S Hillock Ave
W Fuller St
S Vintage Ave
S Lock St
W Marketplace Access Rd
29th St
S Broad St
55
© 2010 Google
Google

T.J. O'Brien to Acme Bend

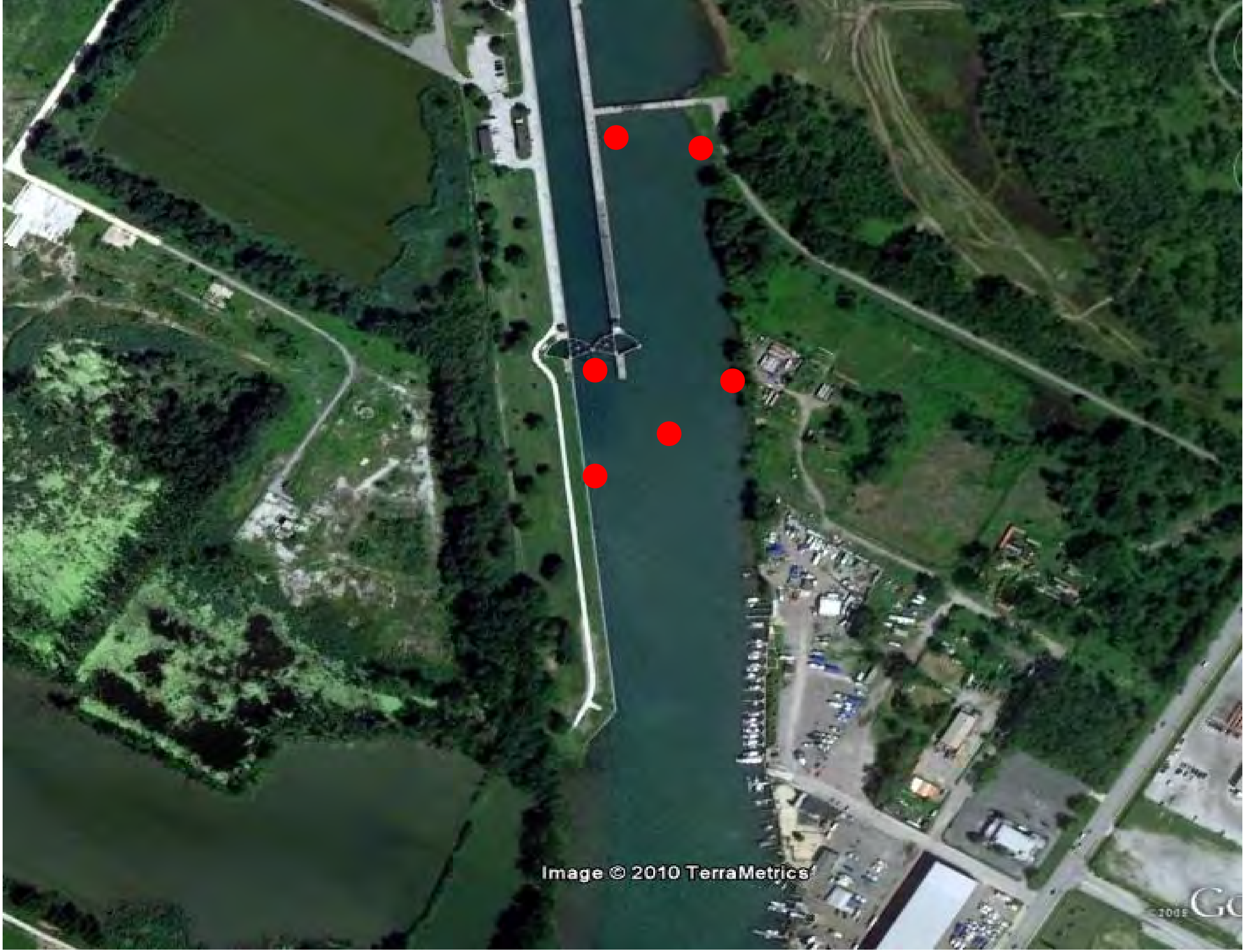
Image © 2010 TerraMetrics

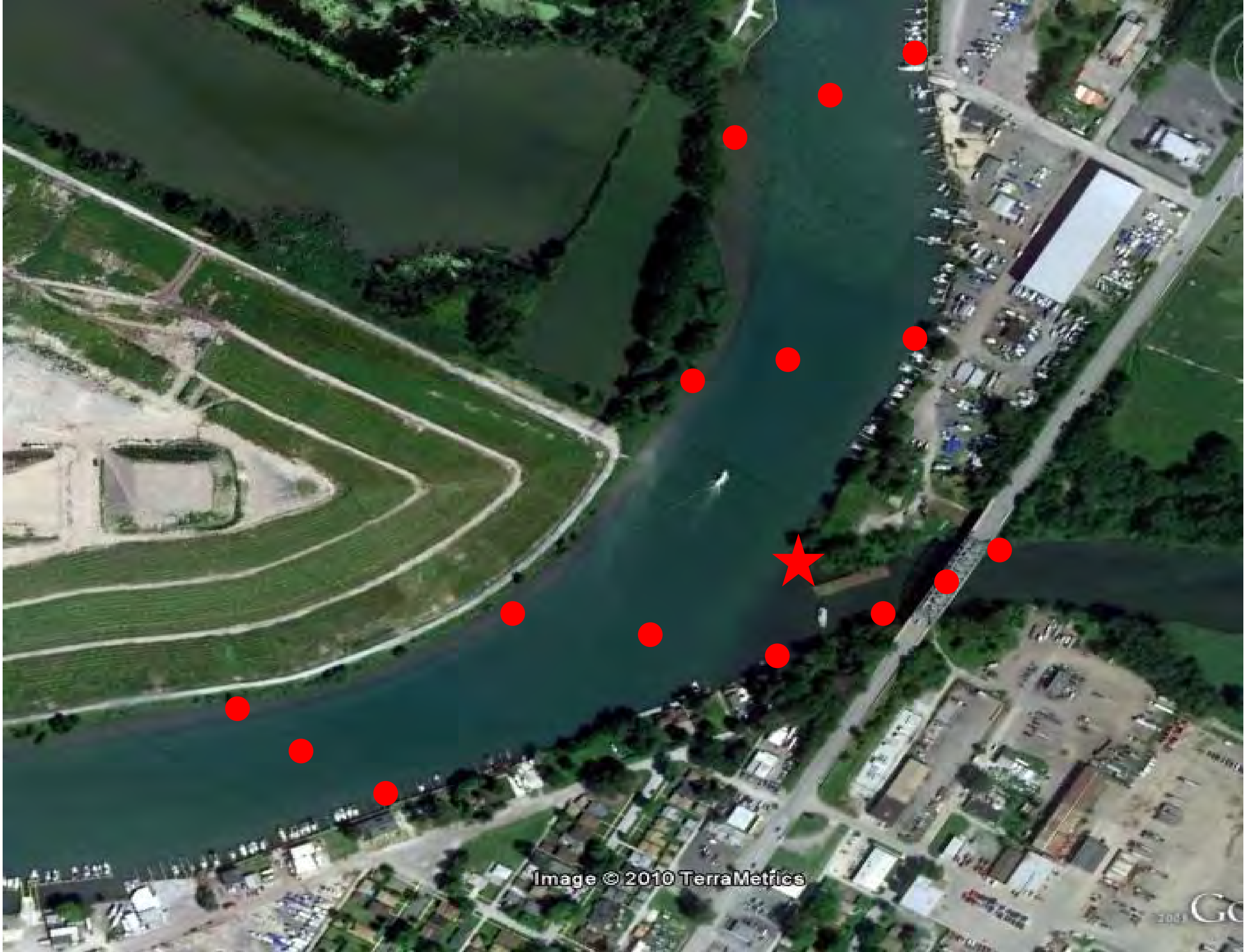
Image © 2010 TerraMetrics

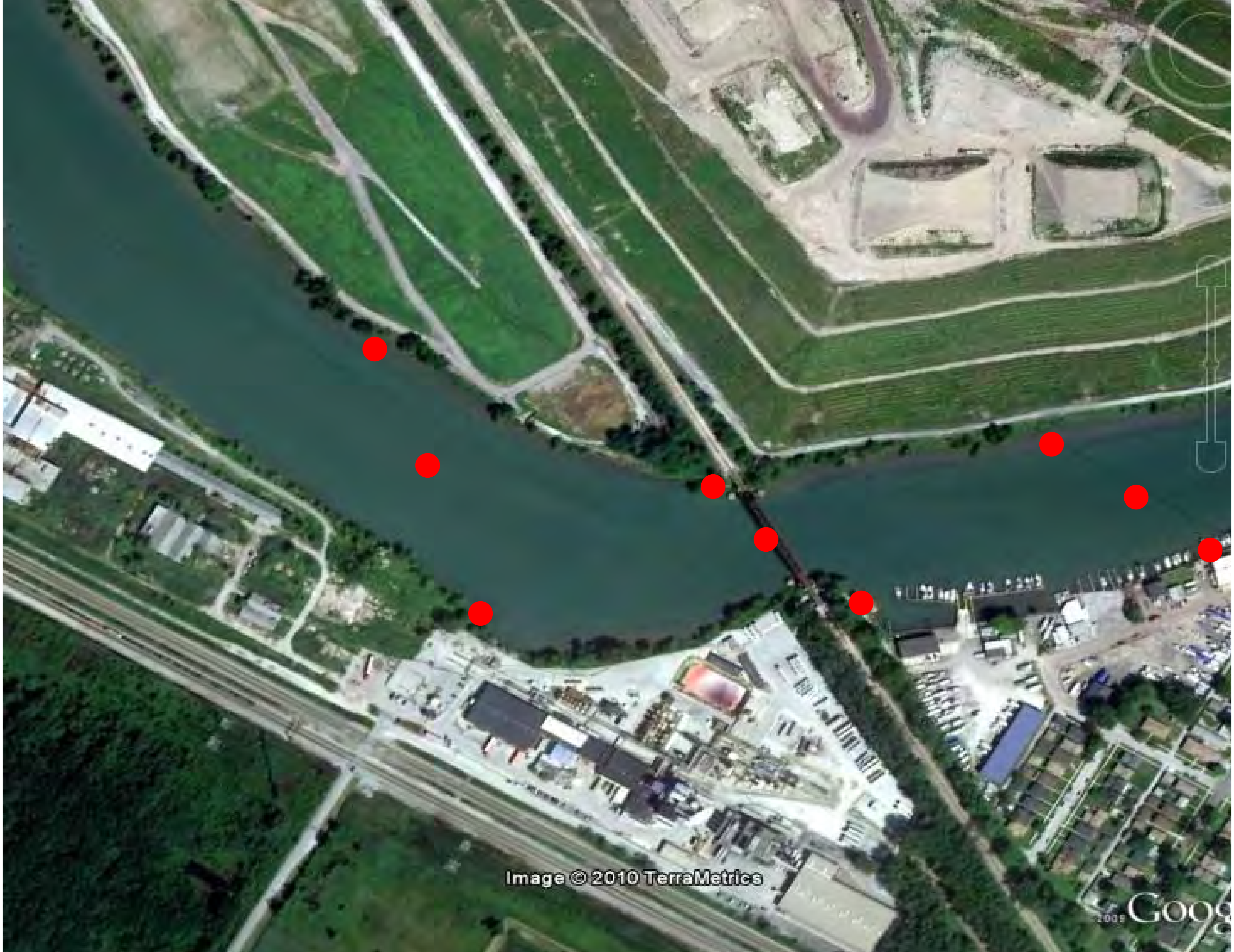
Image © 2010 TerraMetrics

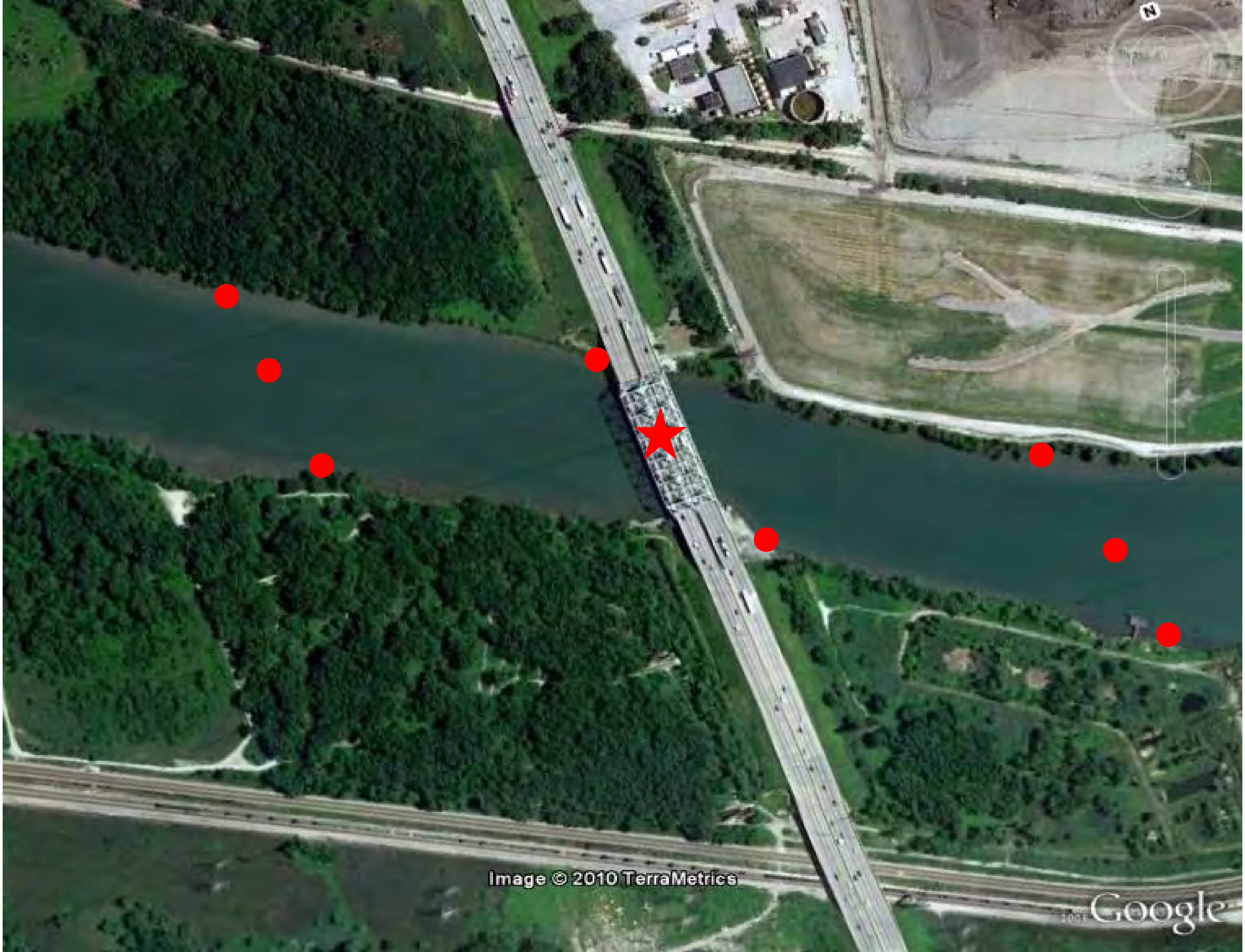
Image © 2010 TerraMetrics
Google

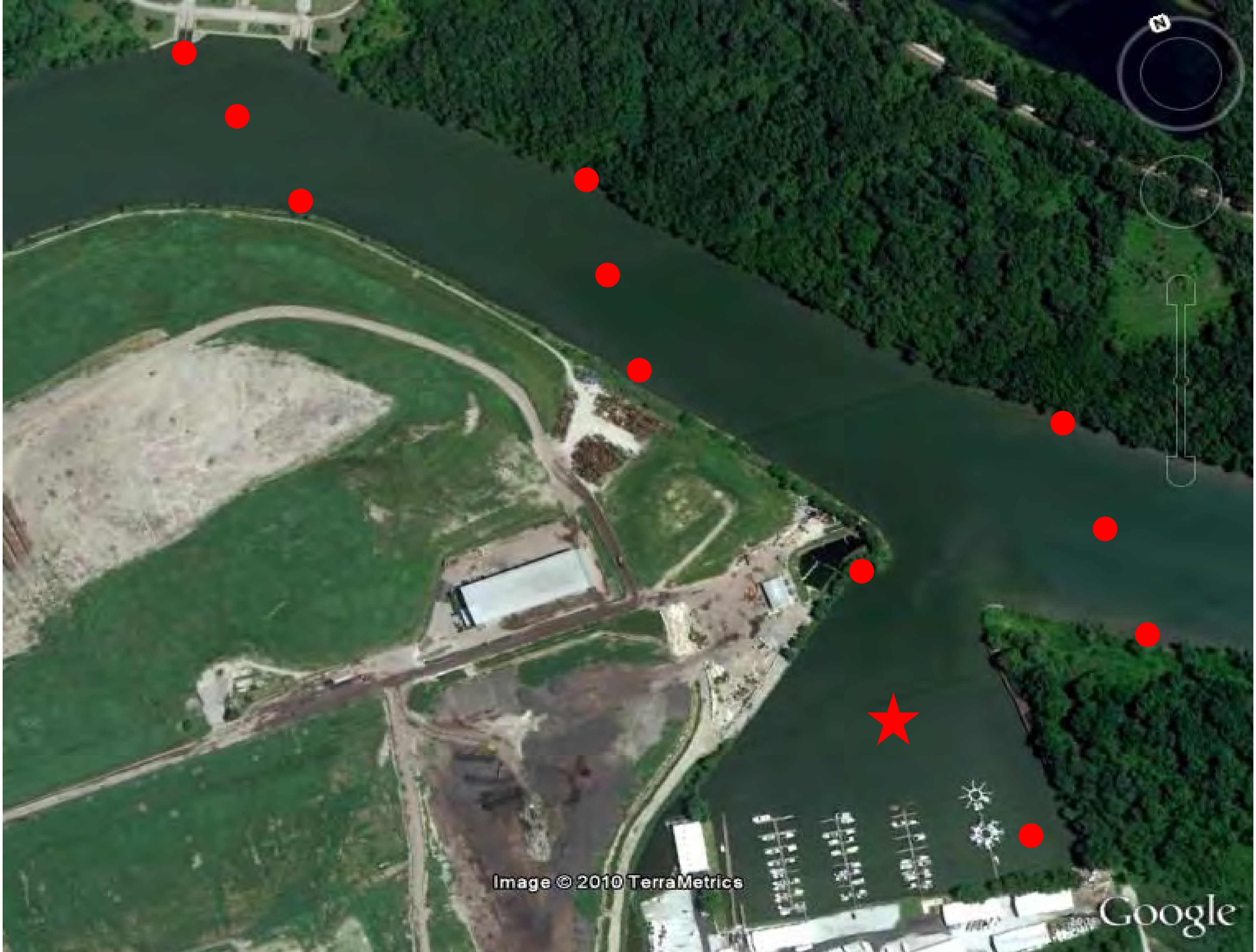

Image © 2010 TerraMetrics
Google

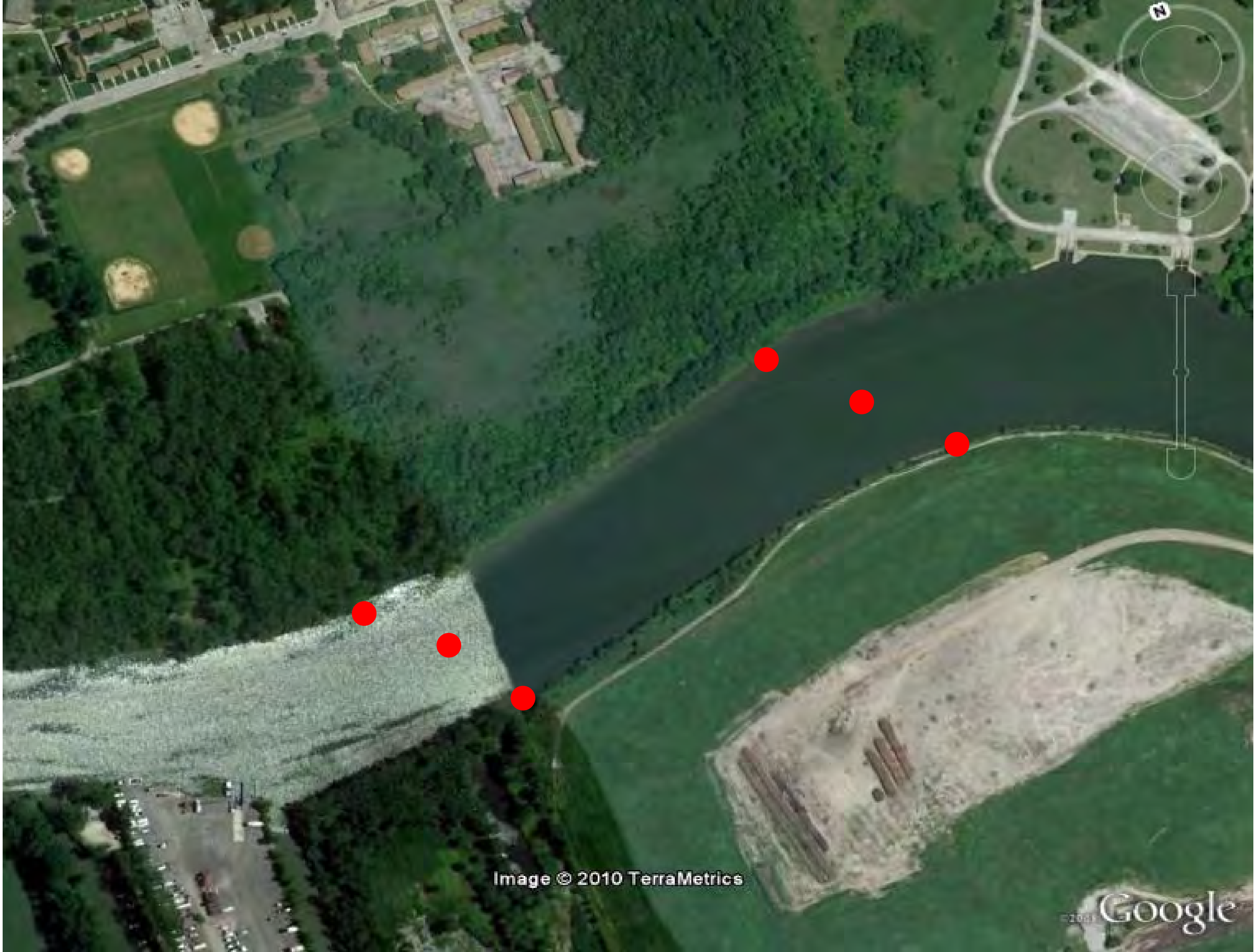
N
Image © 2010 TerraMetrics
Google

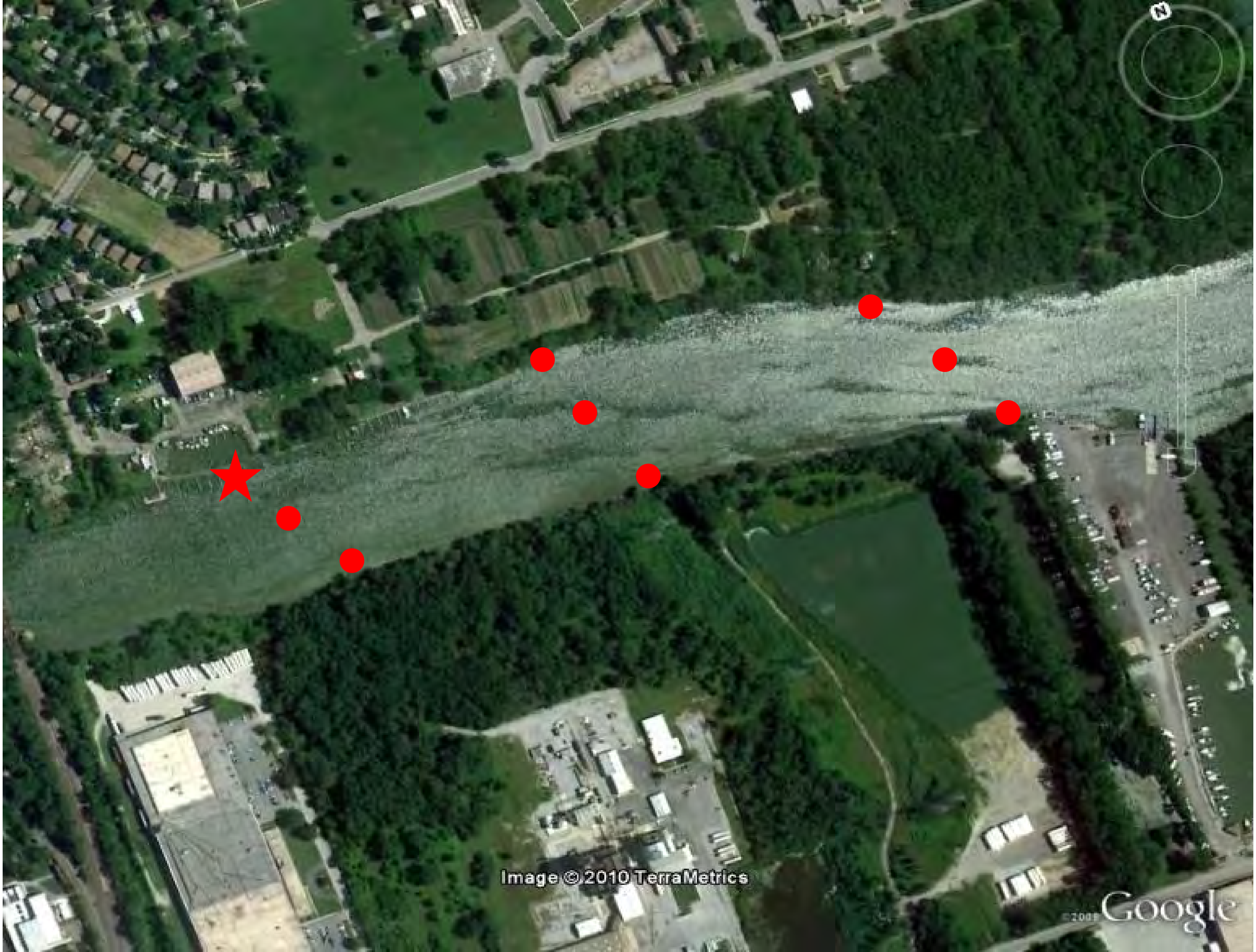
Image © 2010 TerraMetrics
Google

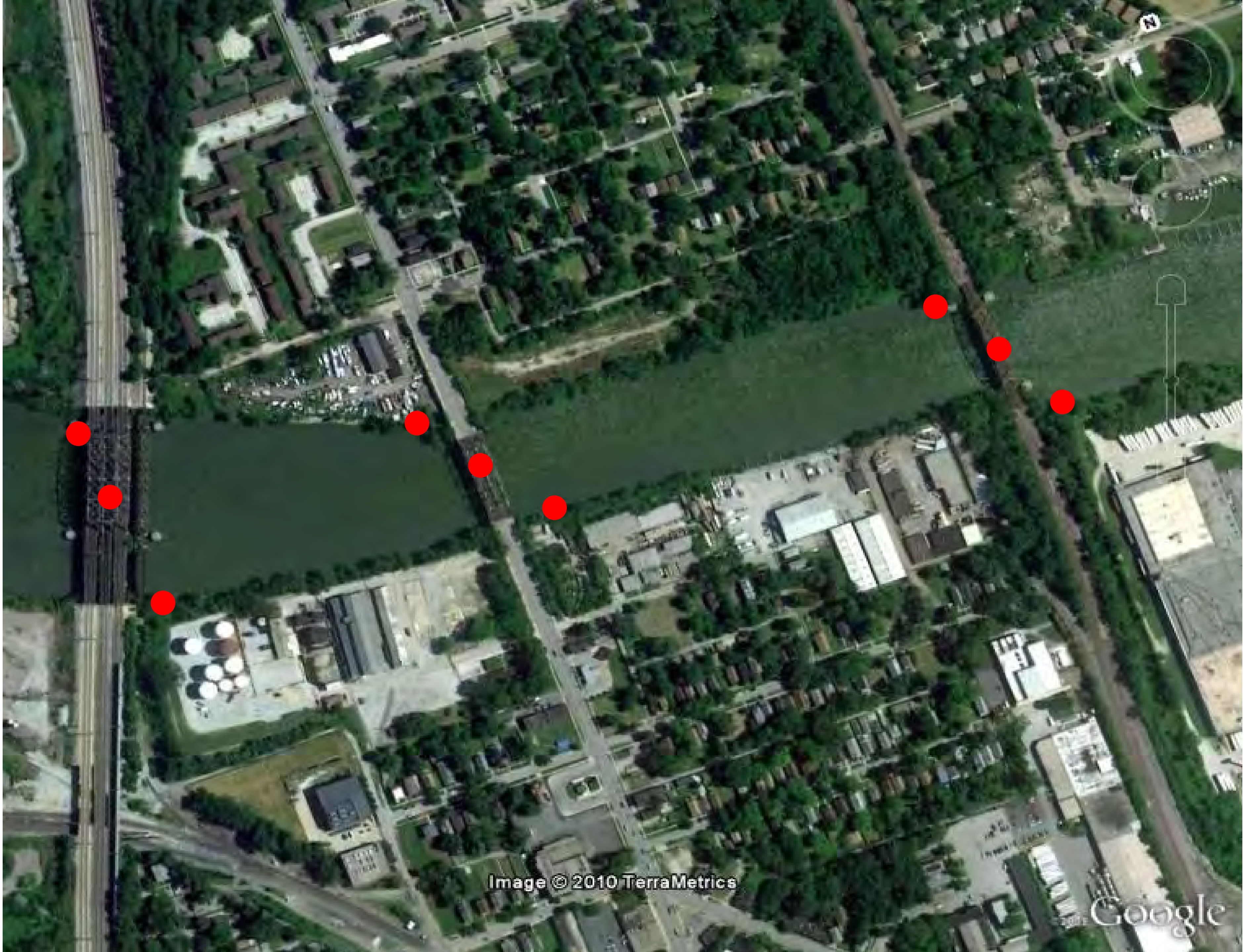
Image © 2010 TerraMetrics
Google

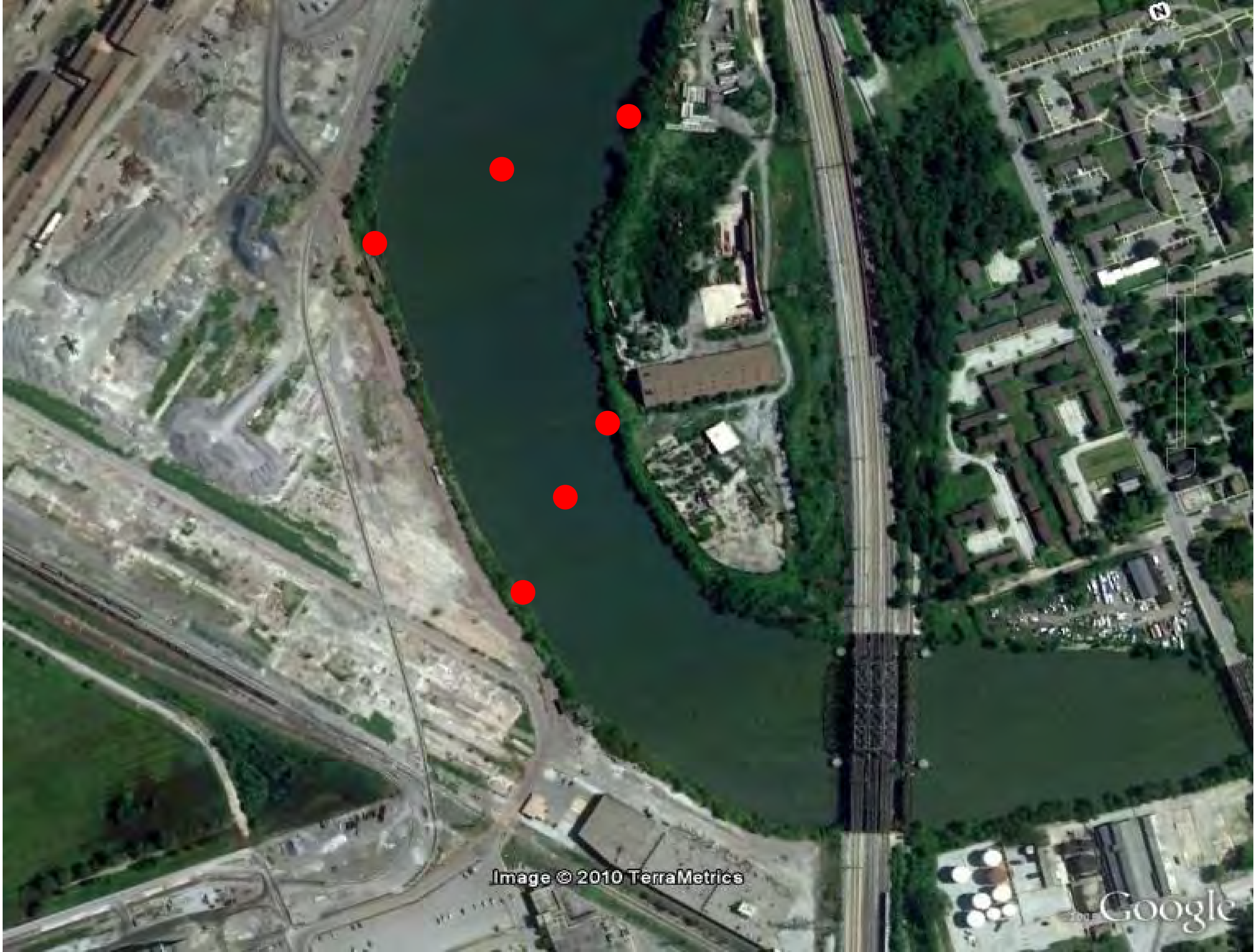
Image © 2010 TerraMetrics
Google

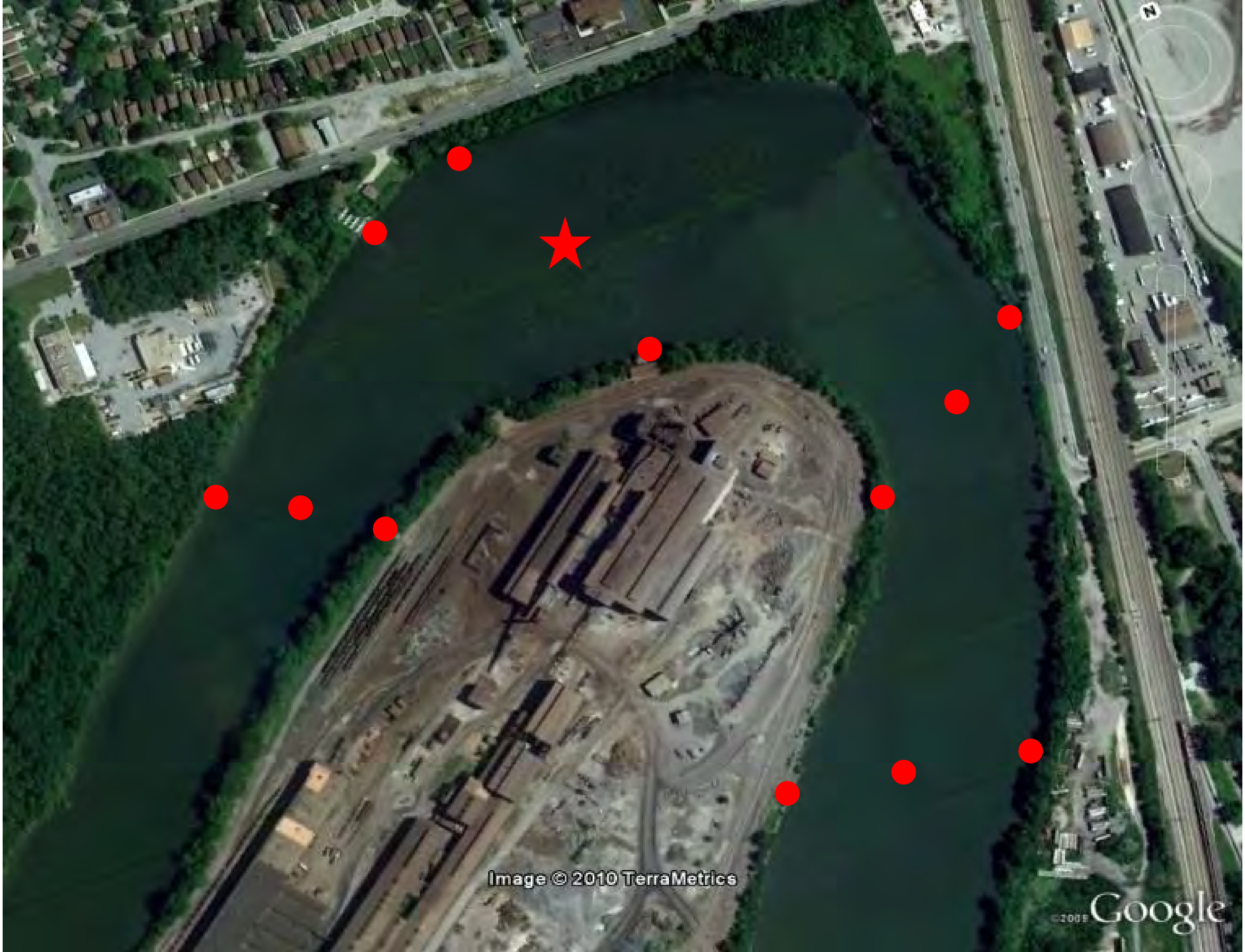

N
Image © 2010 TerraMetrics
©2009 Google

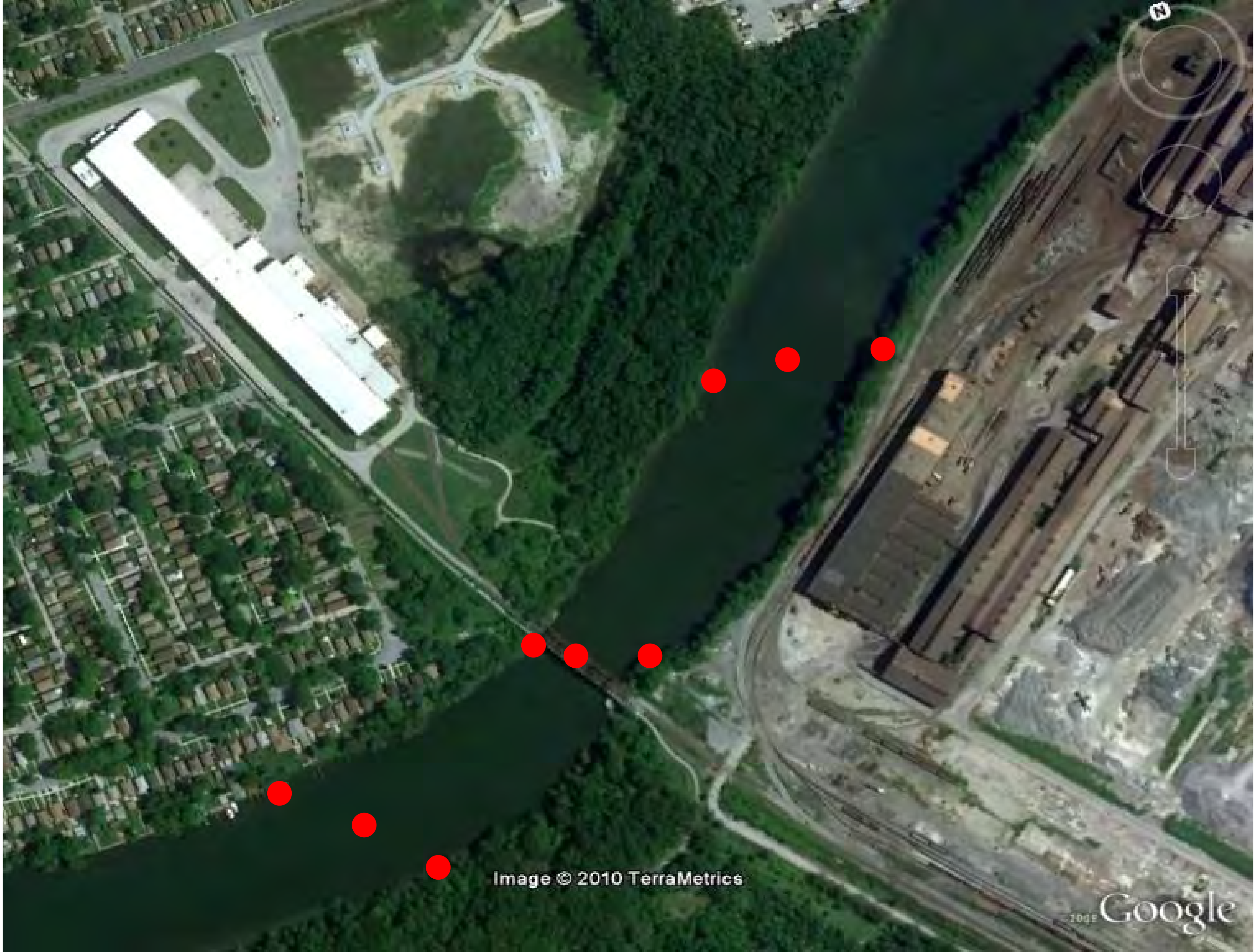
Image © 2010 TerraMetrics
Google

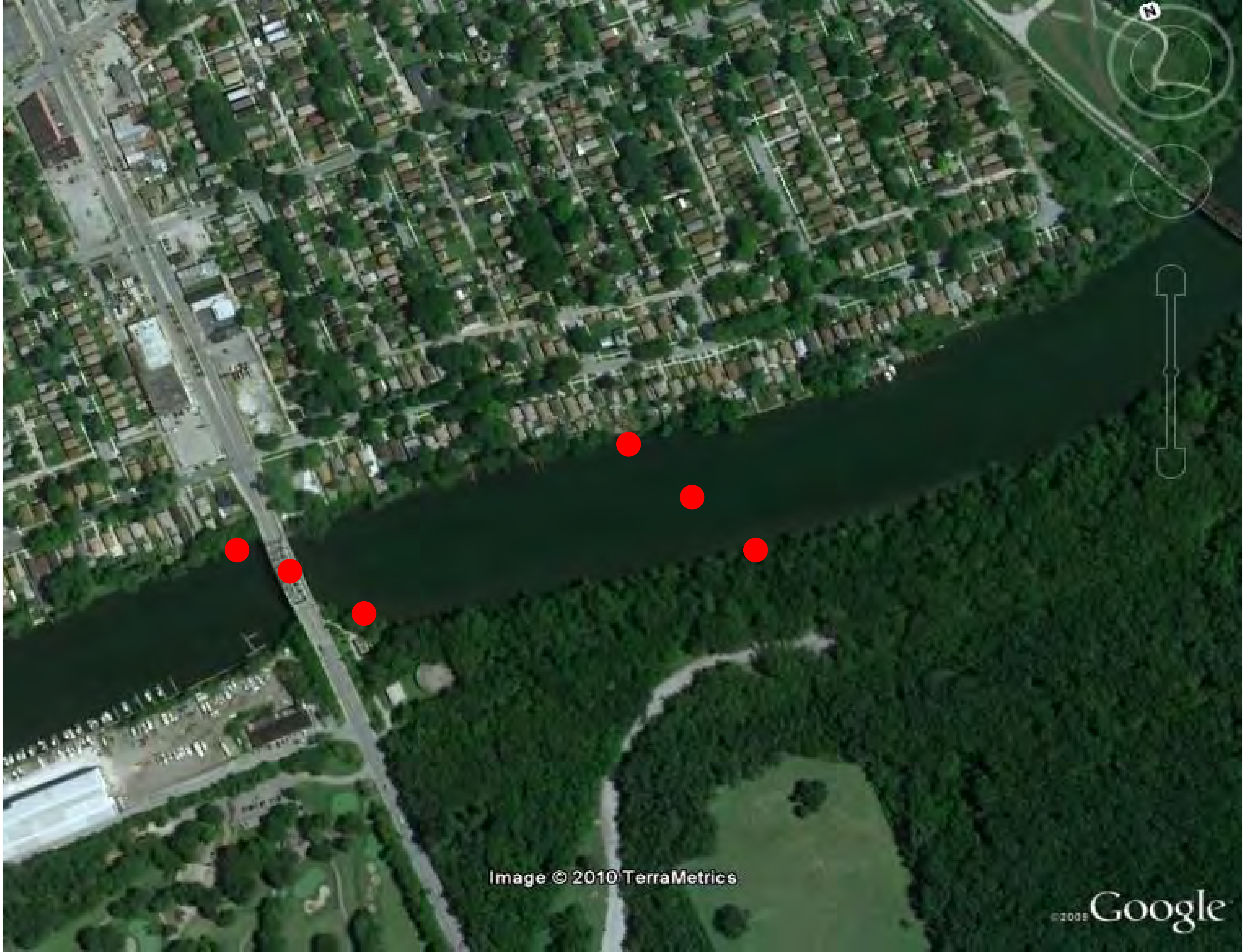
Image © 2010 TerraMetrics
©2009 Google

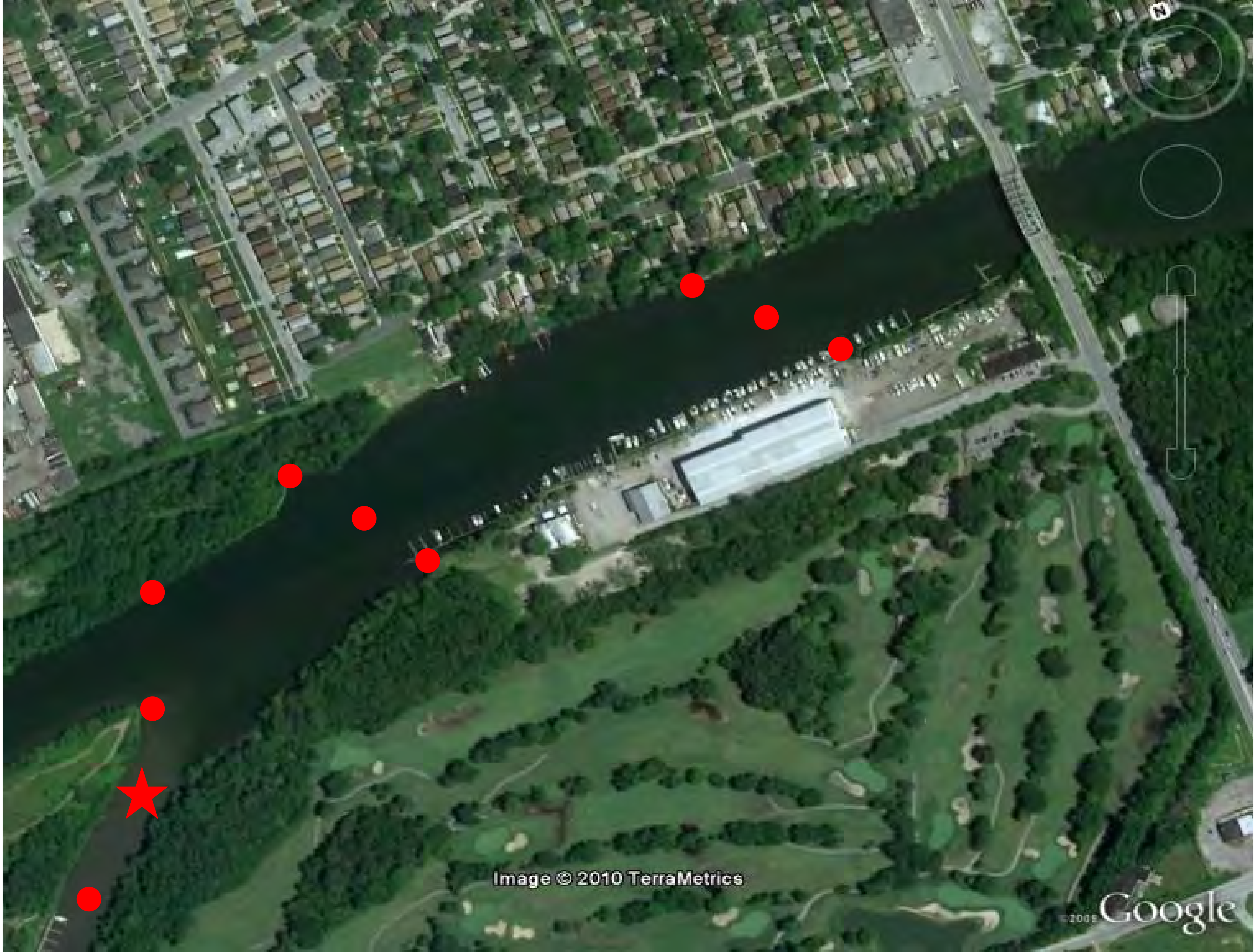
Image © 2010 TerraMetrics
Google

Lake Calumet

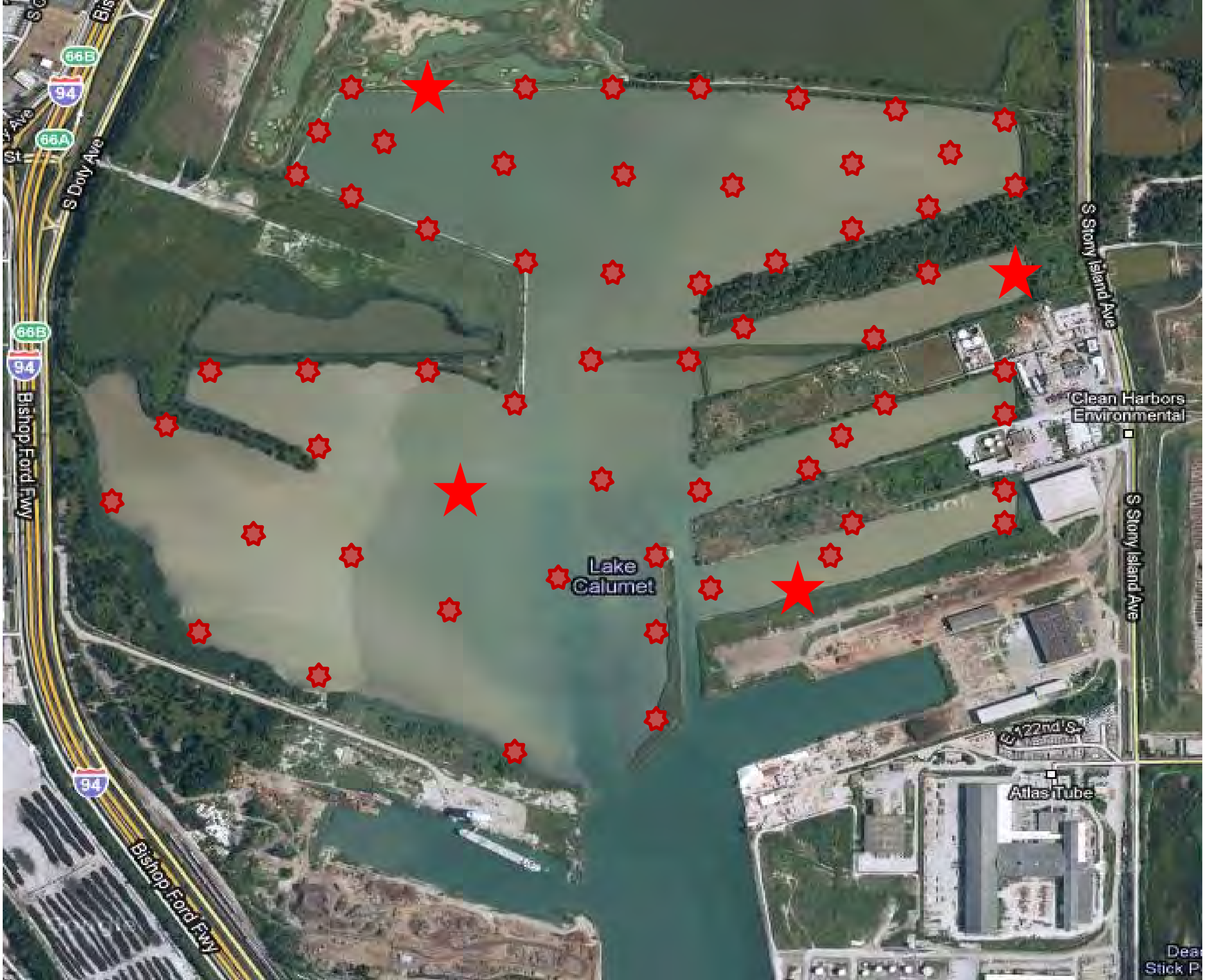

Lake Calumet
Clean Harbors Environmental
Atlas Tube
S Stony Island Ave
Bishop Ford Fwy
S Doty Ave
E 122nd St
94
66B
66A

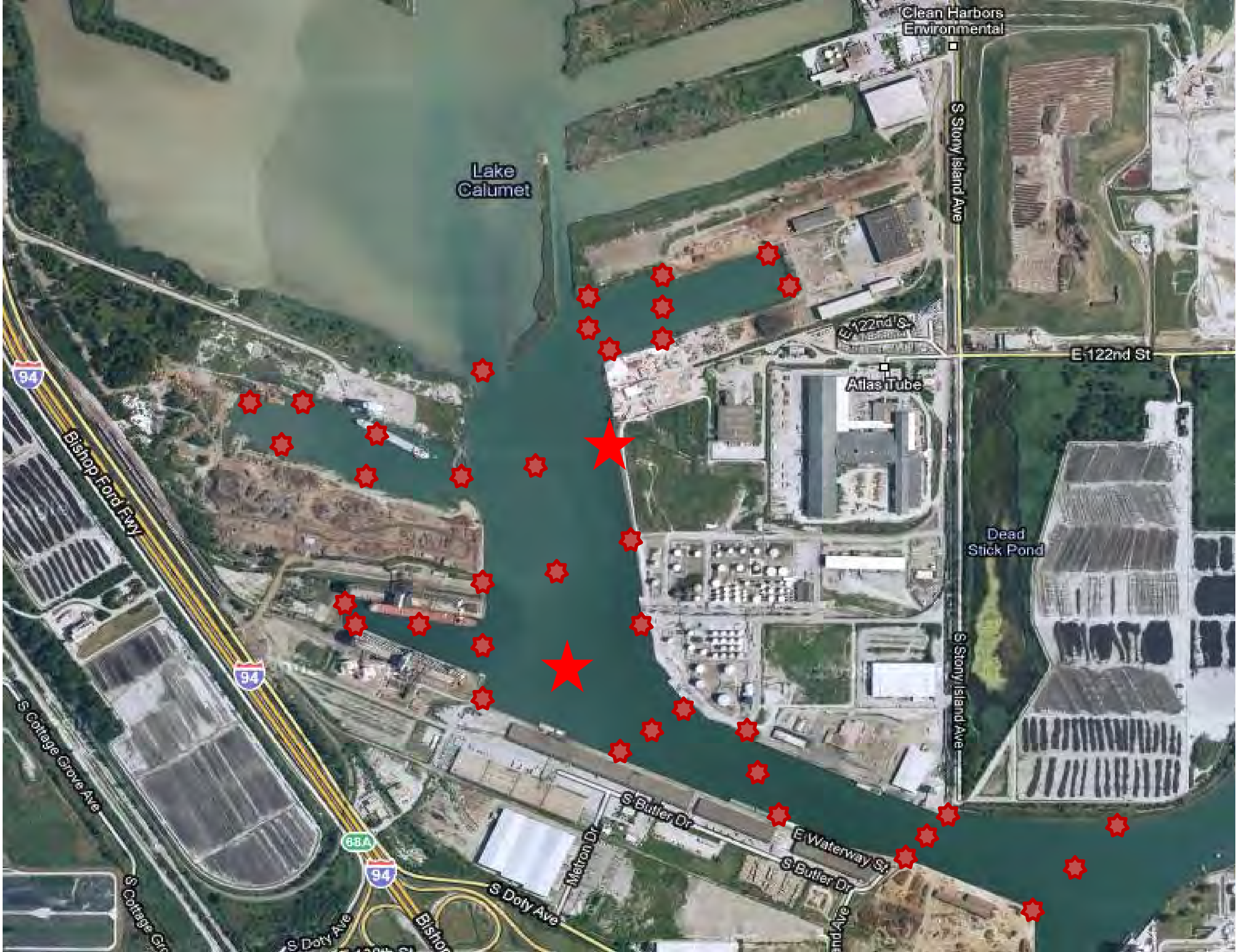

Clean Harbors Environmental
Lake Calumet
S Stony Island Ave
E 122nd St
E 122nd St
Atlas Tube
Dead Stick Pond
94
Bishop Ford Fwy
94
S Cottage Grove Ave
68A
94
S Doty Ave
Metron Dr
S Butler Dr
E Waterway St
S Butler Dr
S Stony Island Ave
S Doty Ave

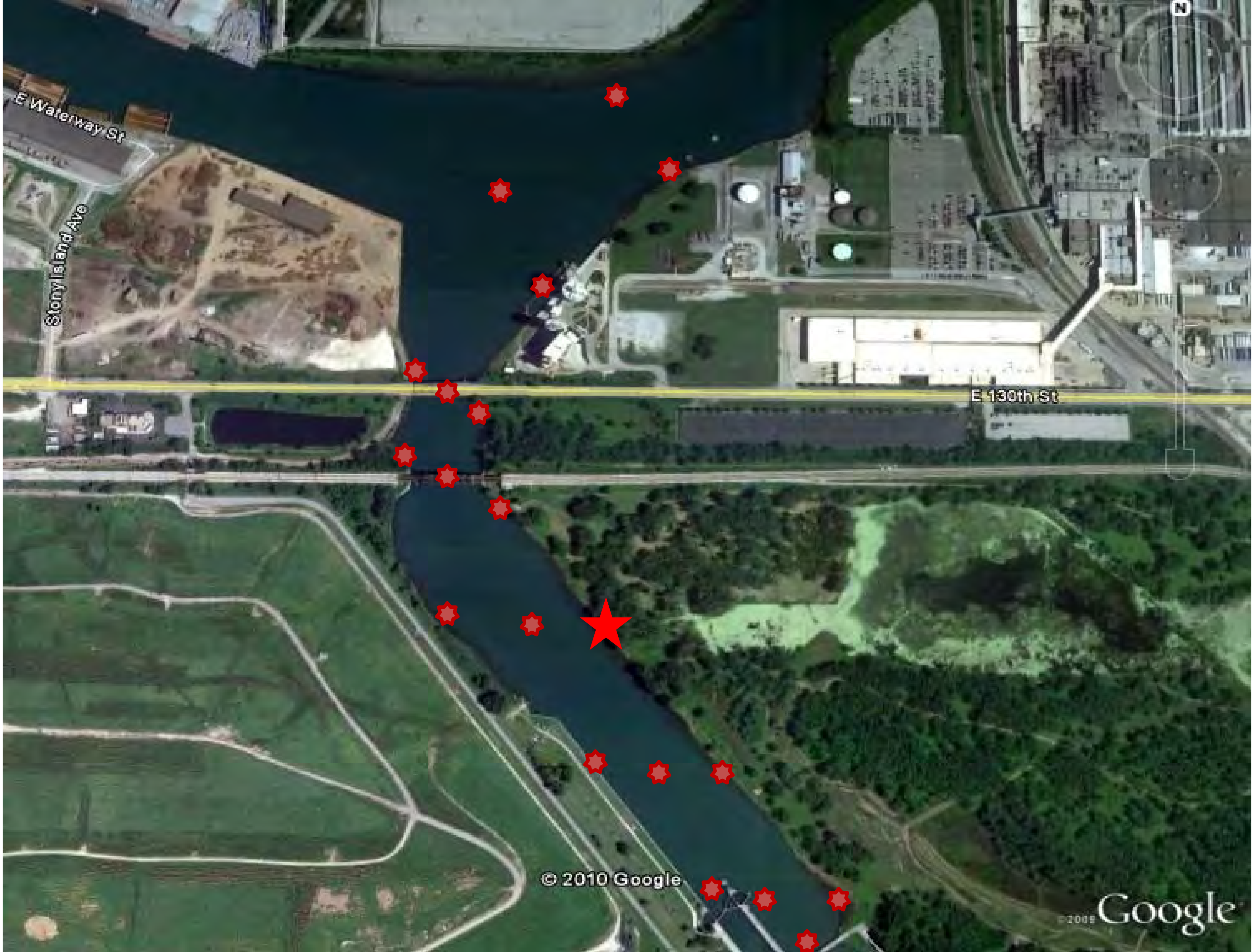
E Waterway St
Stony Island Ave
E 130th St
© 2010 Google
Google

Lockport Pool
(Above and Below Barrier)

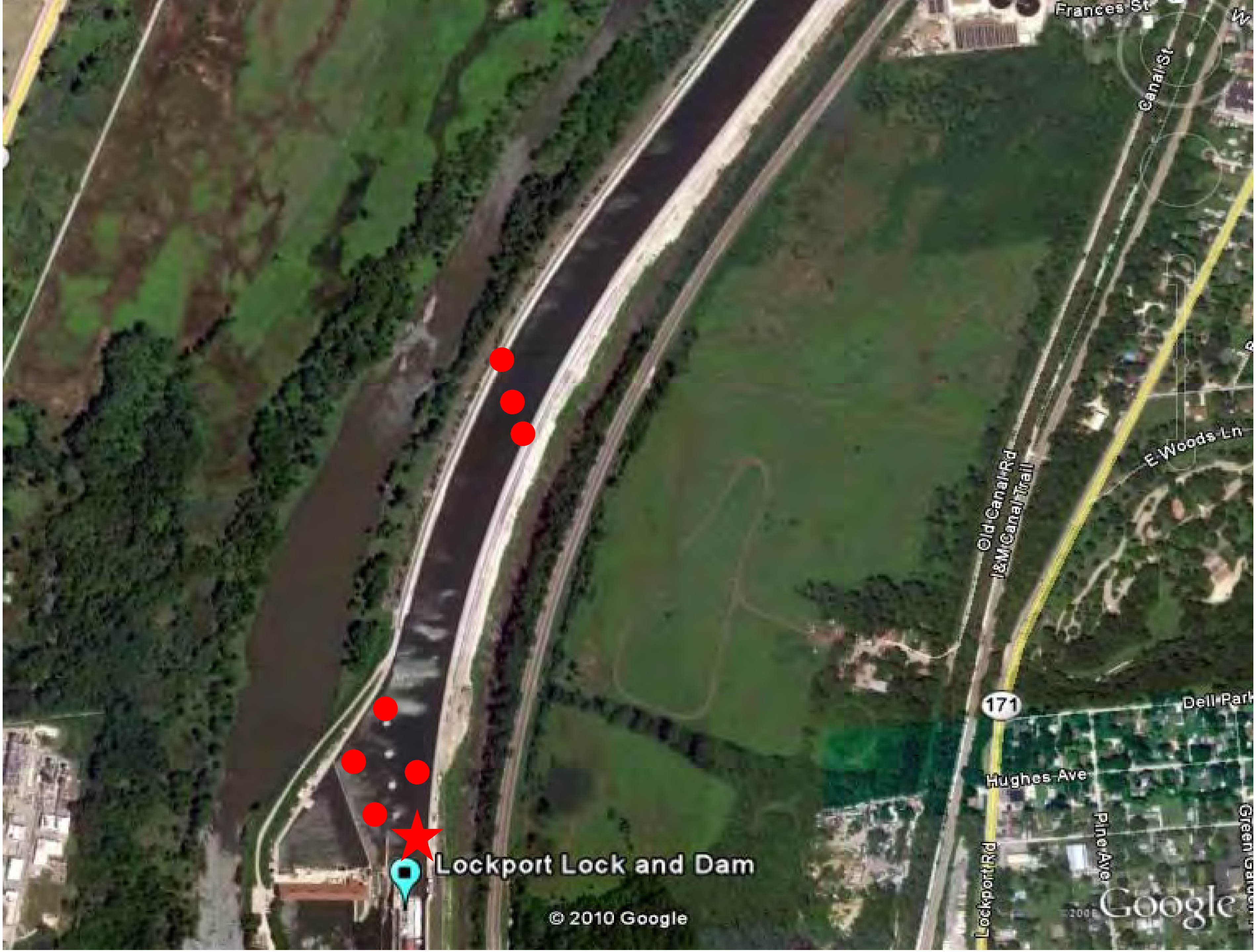

Frances St
Canal St
E Woods Ln
Old Canal Rd
I&M Canal Trail
171
Dell Park
Hughes Ave
Pine Ave
Lockport Rd
Lockport Lock and Dam
© 2010 Google
Google

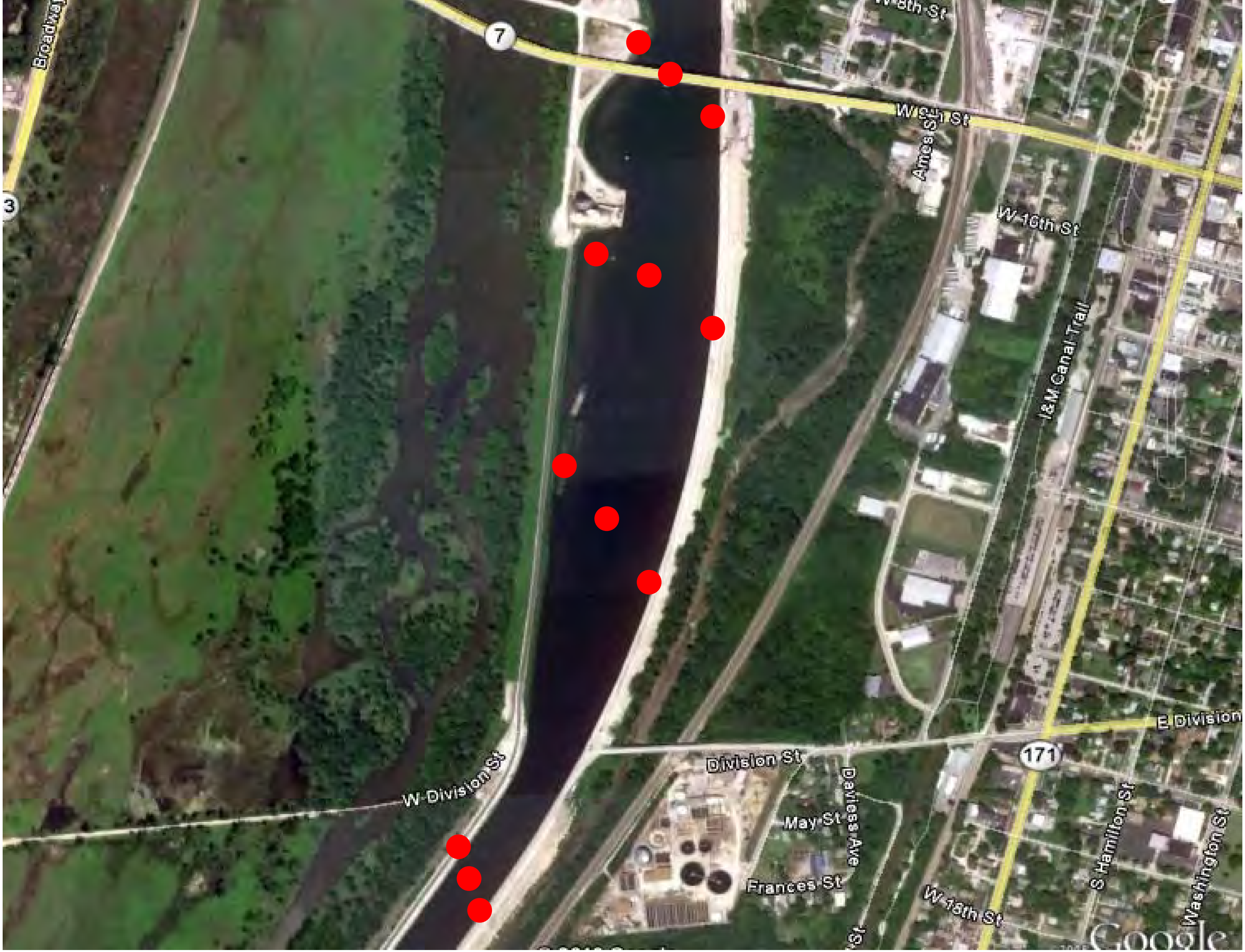
Broadway
7
Ames St
W 10th St
I&M Canal Trail
E Division
171
Division St
W Division St
Daviess Ave
May St
Frances St
W 15th St
S Hamilton St
Washington St
Google

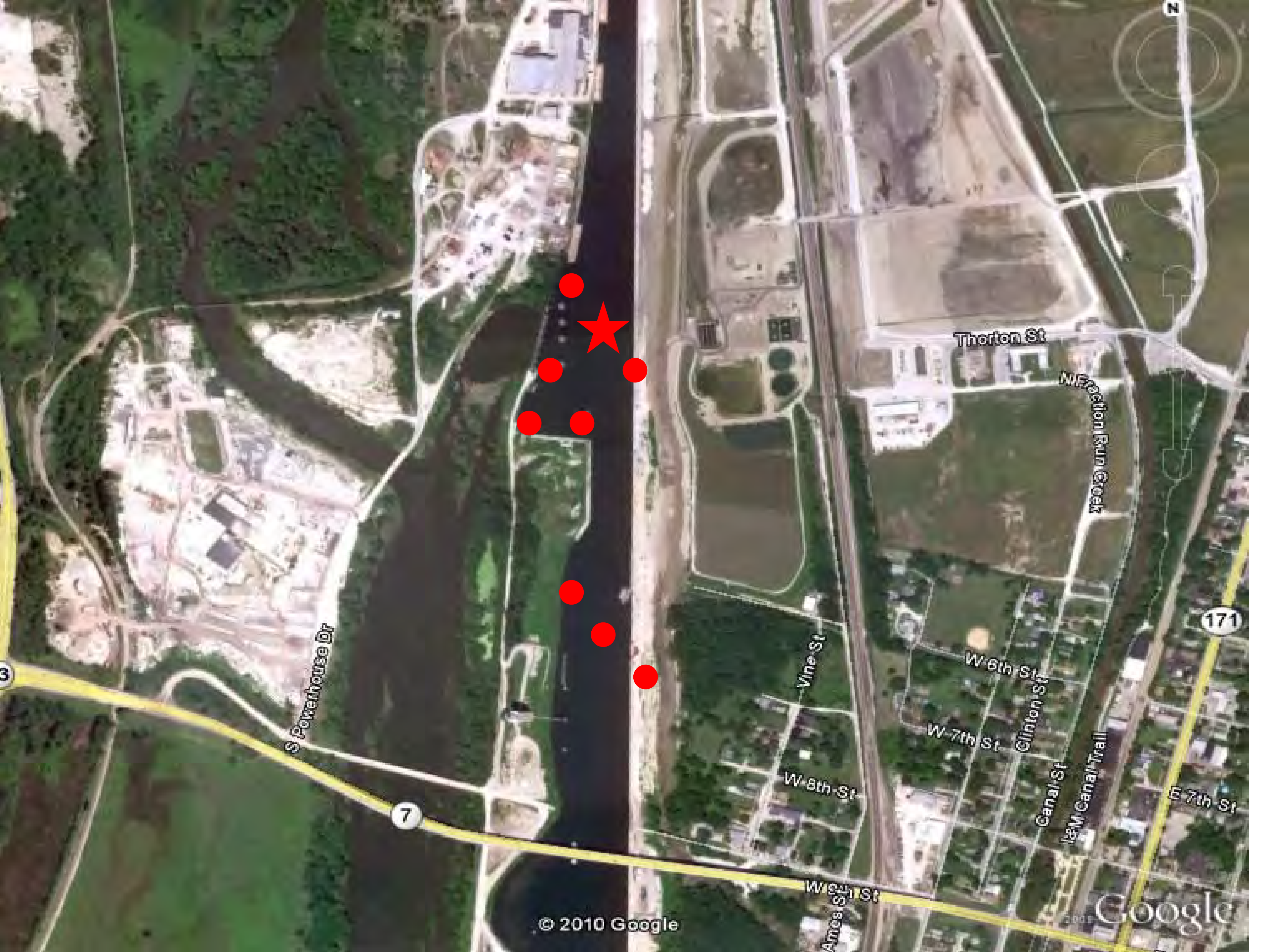

N
Thorton St
N Fraction Run Creek
171
S Powerhouse Dr
Vine St
W 6th St
Clinton St
W 7th St
W 8th St
Canal St
I&M Canal Trail
E 7th St
7
W 5th St
Ames St
© 2010 Google
Google

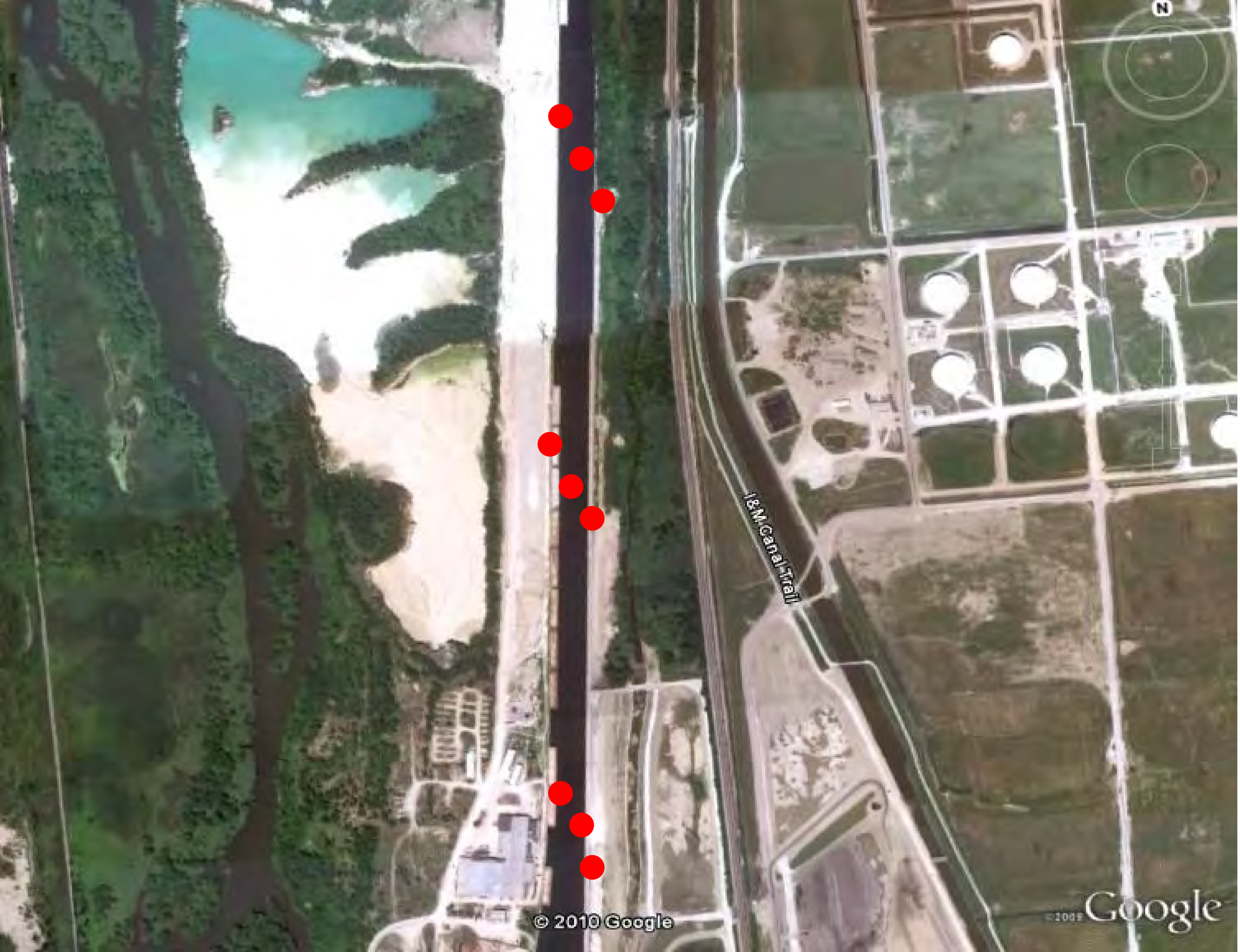
N
I&M Canal Trail
© 2010 Google
Google

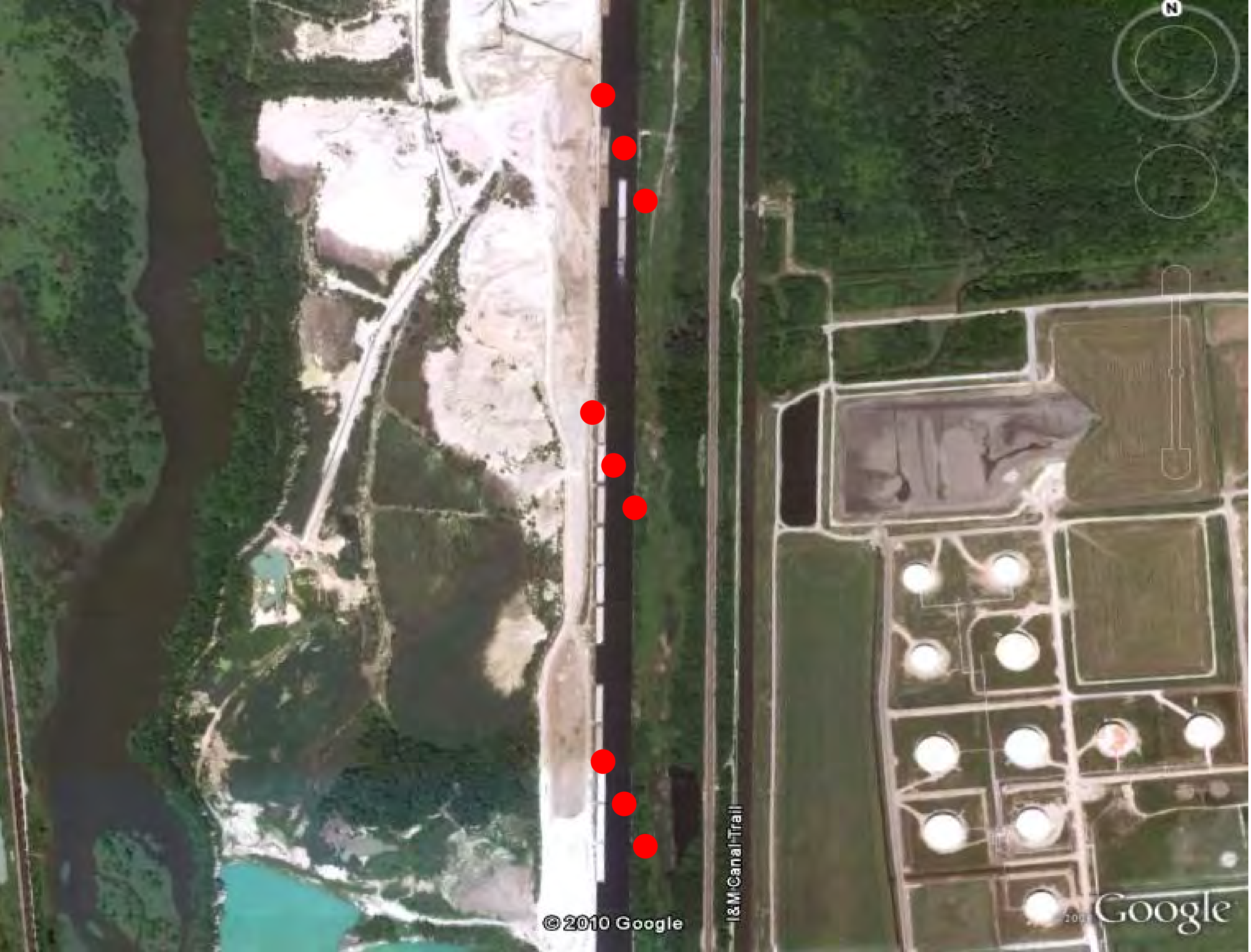
N
© 2010 Google
I&M Canal Trail
Google

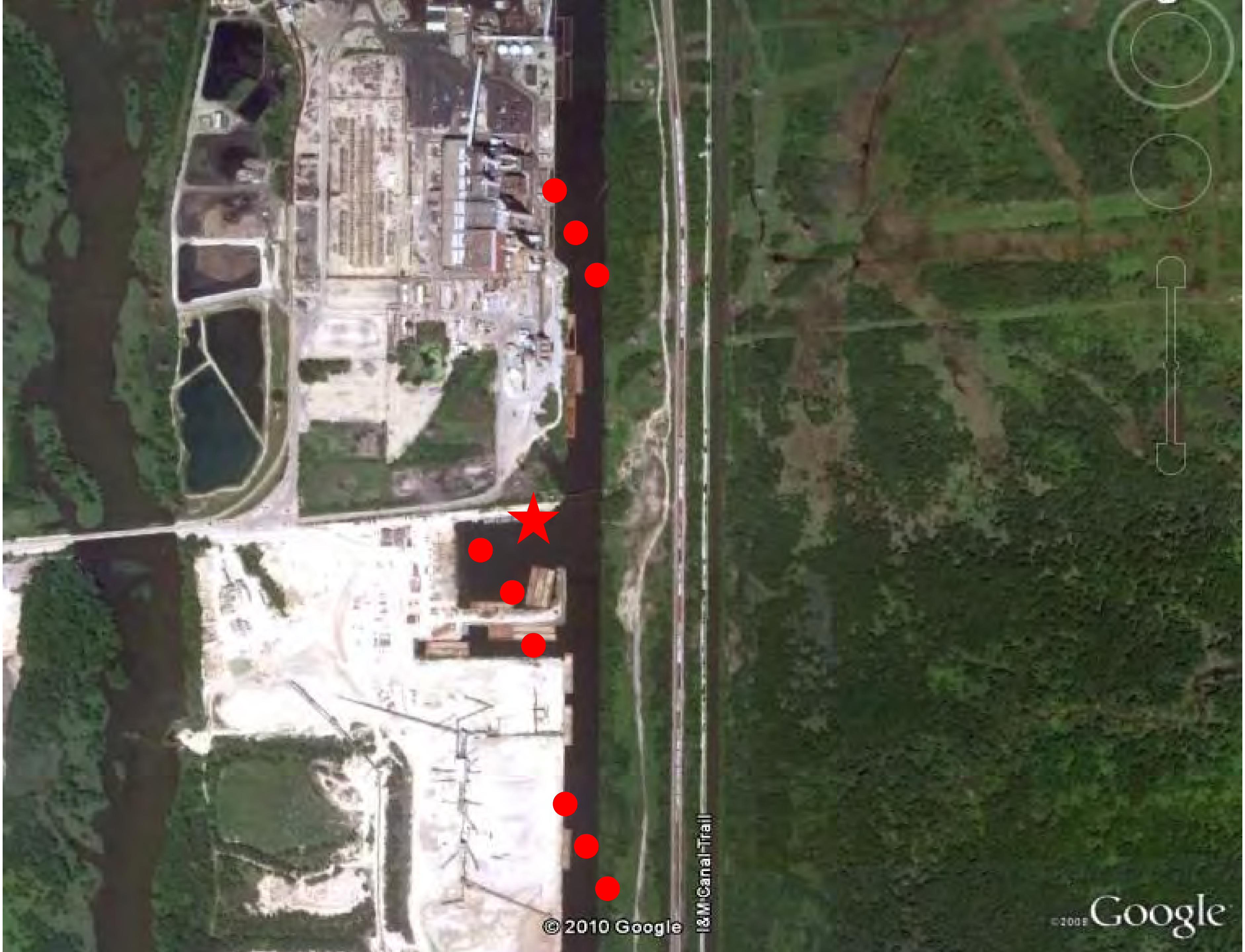
© 2010 Google
I&M Canal Trail
Google

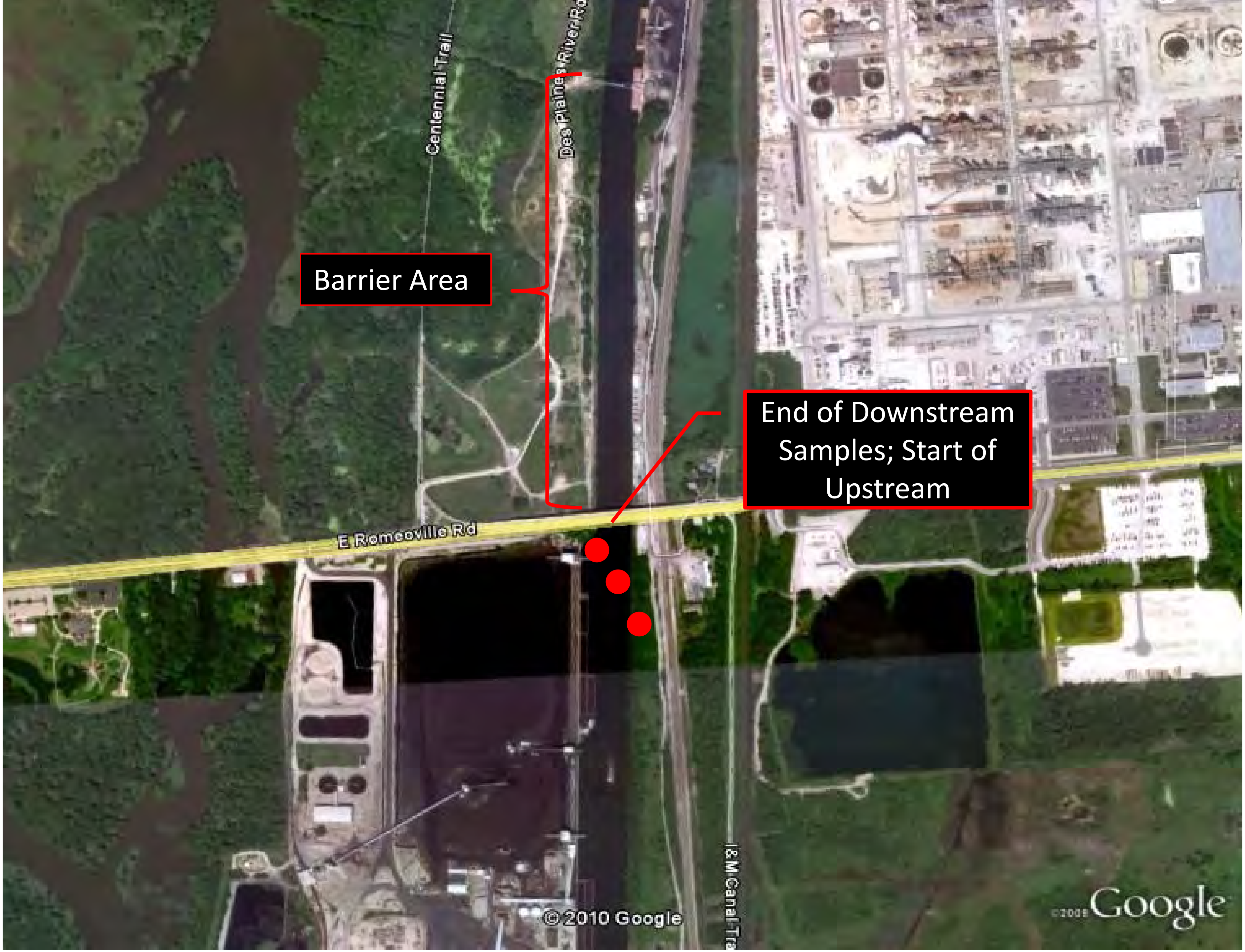

Barrier Area
End of Downstream Samples; Start of Upstream
Centennial Trail
Des Plaines River Rd
E Romeoville Rd
I&M Canal Tra
© 2010 Google
Google

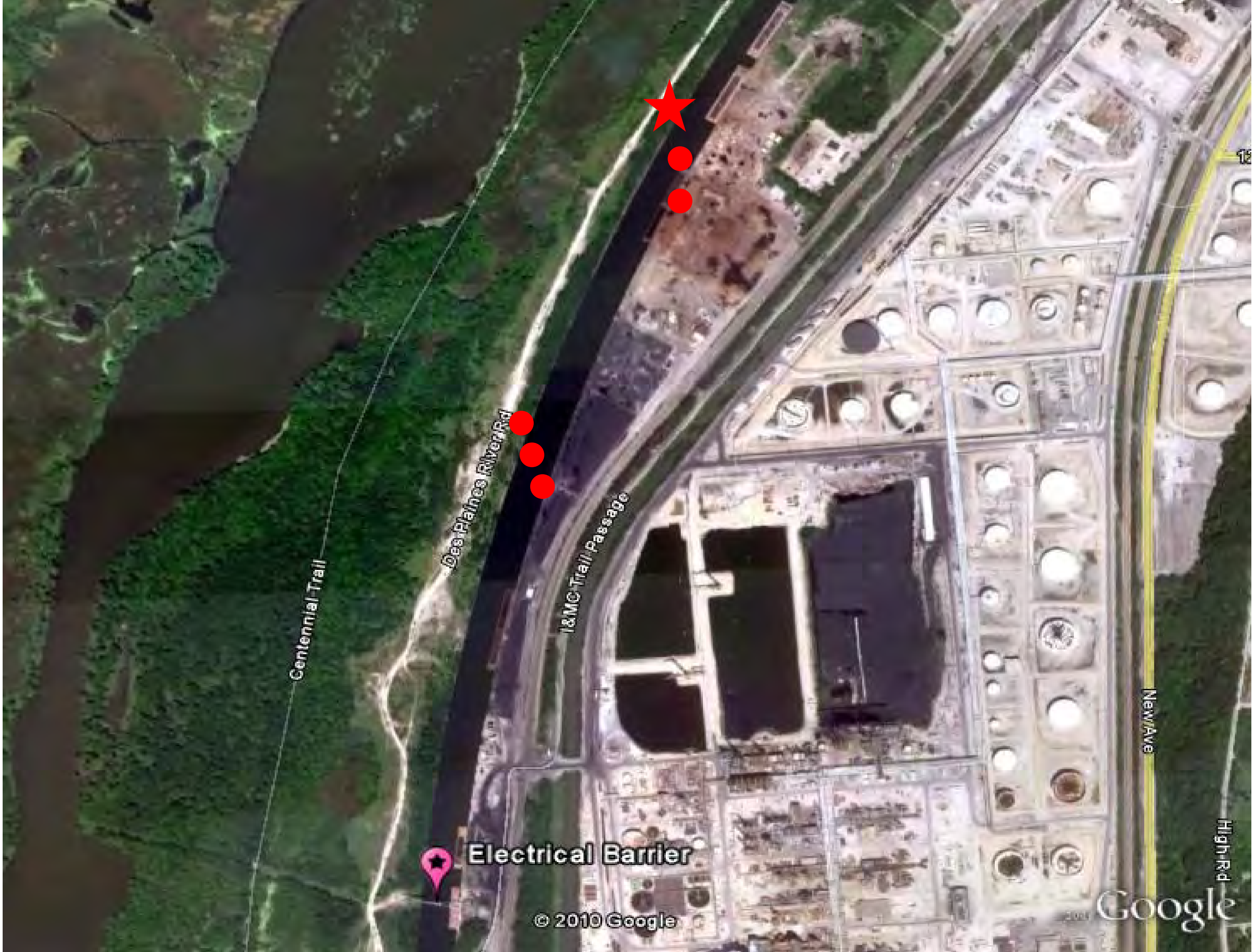
Centennial Trail
Des Plaines River Rd
I&MC Trail Passage
New Ave
High Rd
Electrical Barrier
© 2010 Google
Google

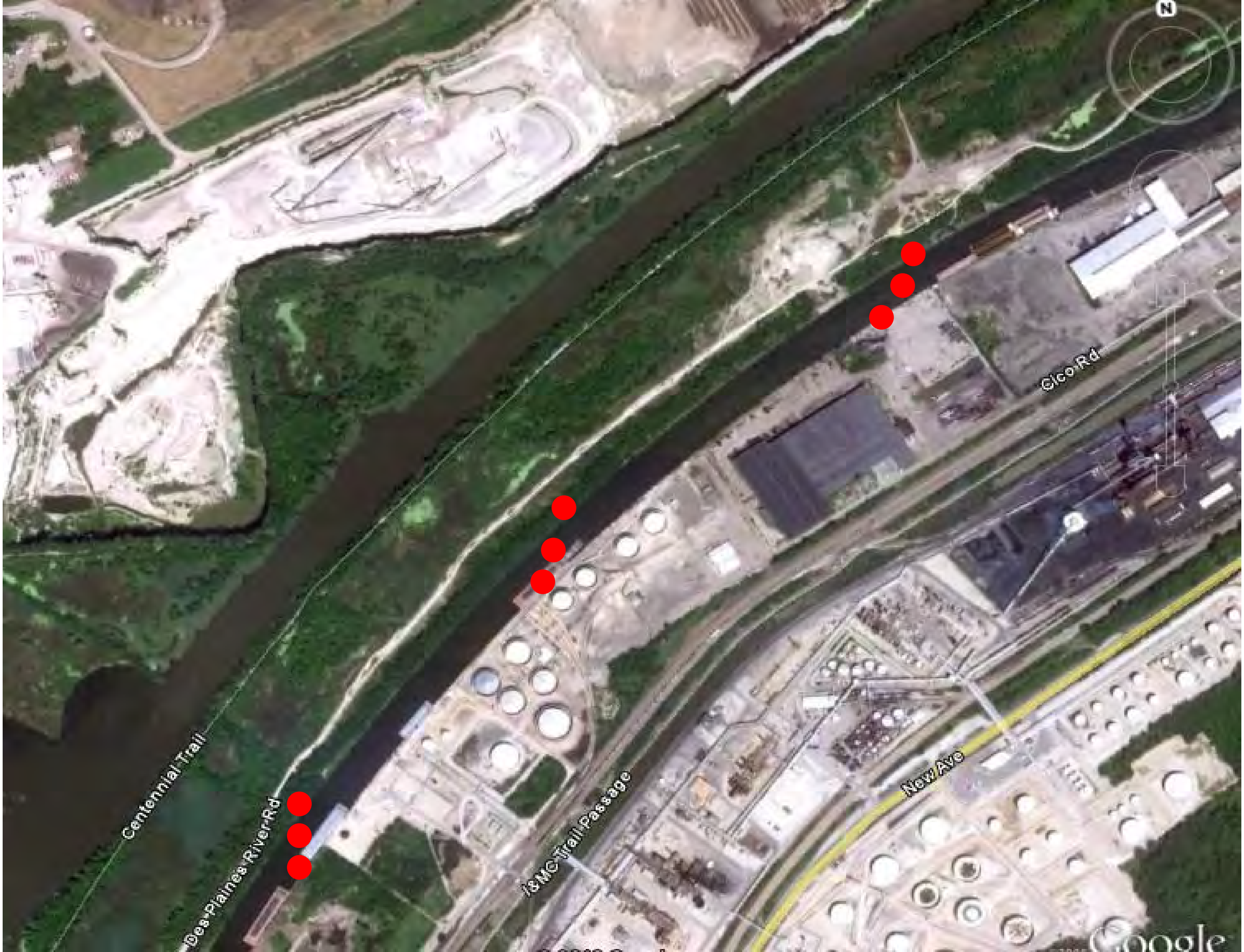
Cico Rd
Centennial Trail
Des Plaines River Rd
I&MC Trail Passage
New Ave
Google

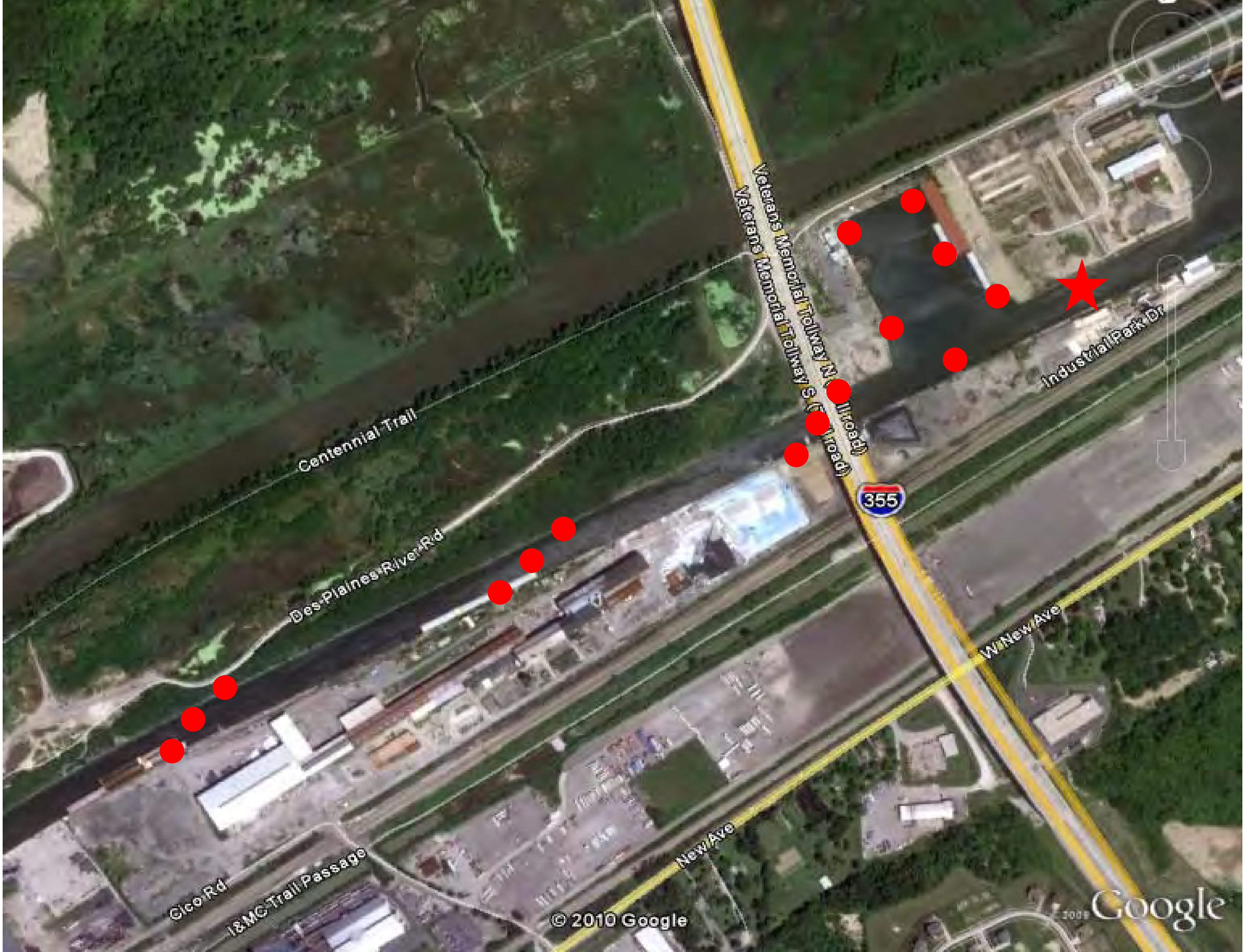

Veterans Memorial Tollway N (I-road)
Veterans Memorial Tollway S (I-road)
355
Centennial Trail
Des Plaines River Rd
Industrial Park Dr
W New Ave
New Ave
Cico Rd
I&MC Trail Passage
© 2010 Google
Google

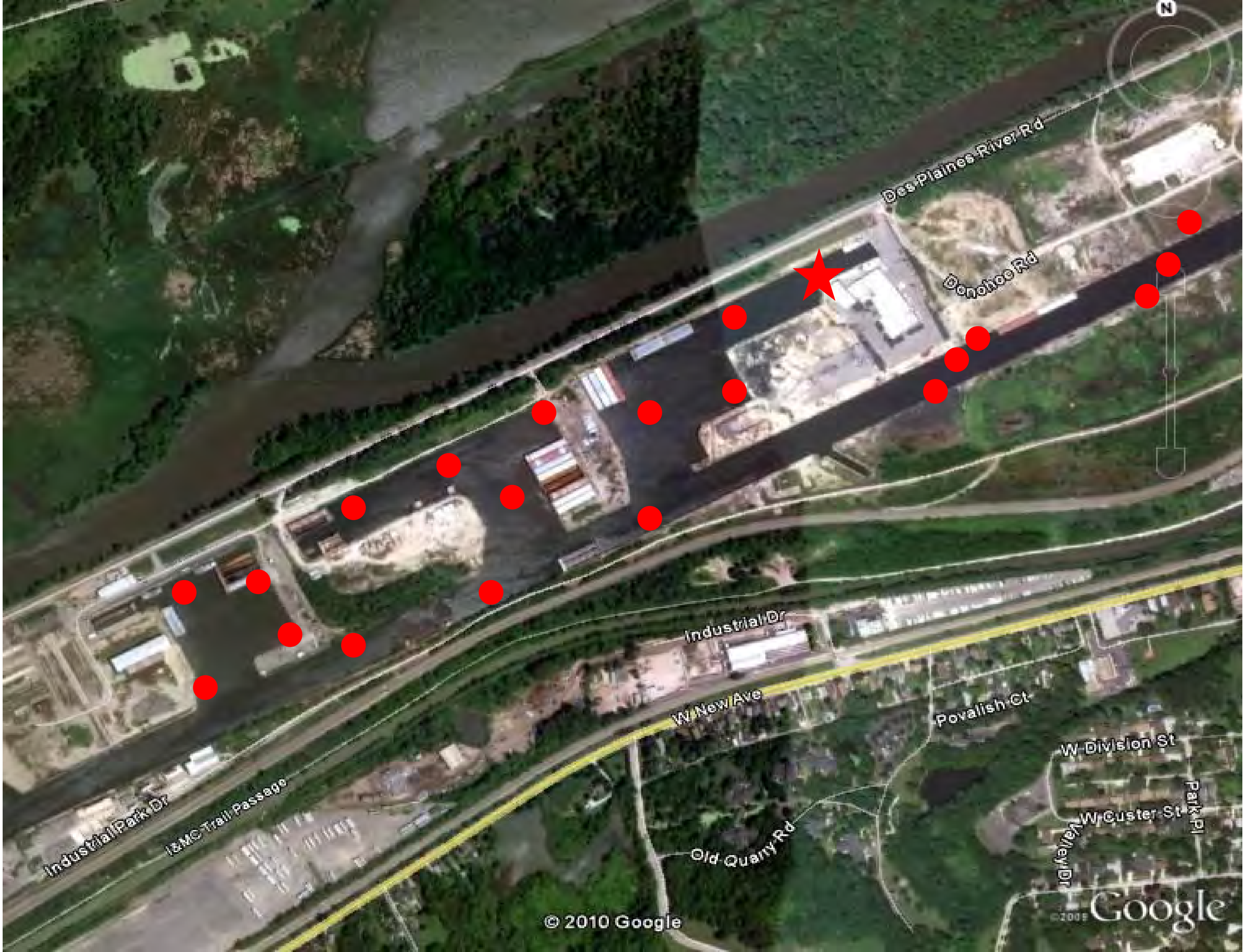

N
Des Plaines River Rd
Donohoe Rd
Industrial Dr
W New Ave
Povalish Ct
W Division St
W Custer St
Park Pl
Valley Dr
Old Quarry Rd
I&MC Trail Passage
Industrial Park Dr
© 2010 Google
Google

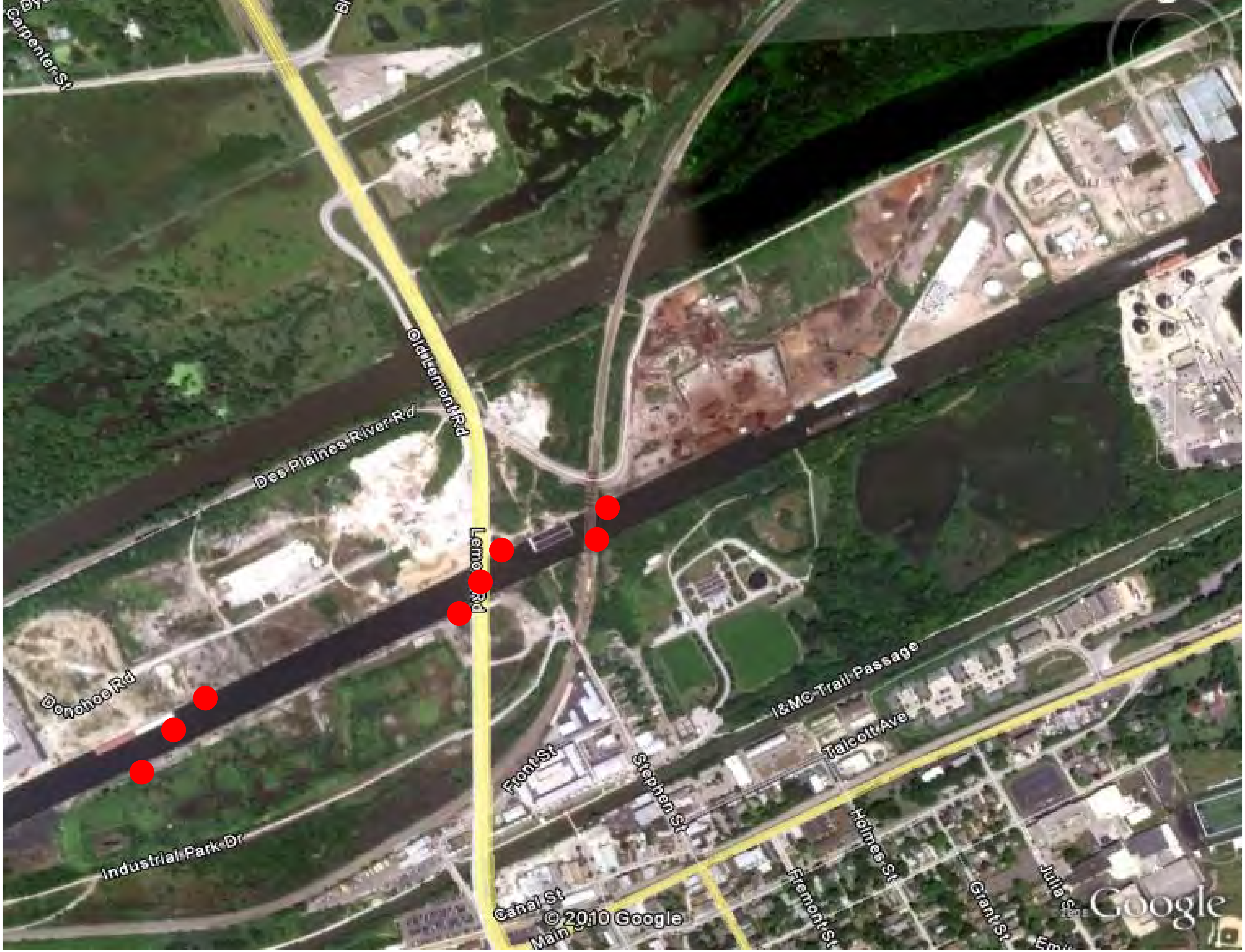
Carpenter St
Old Lemont Rd
Des Plaines River Rd
Lemont Rd
Denohoe Rd
I&M C Trail Passage
Talcott Ave
Front St
Stephen St
Holmes St
Fremont St
Grant St
Julia St
Industrial Park Dr
Canal St
Main St
© 2010 Google
Google

Des Plaines River

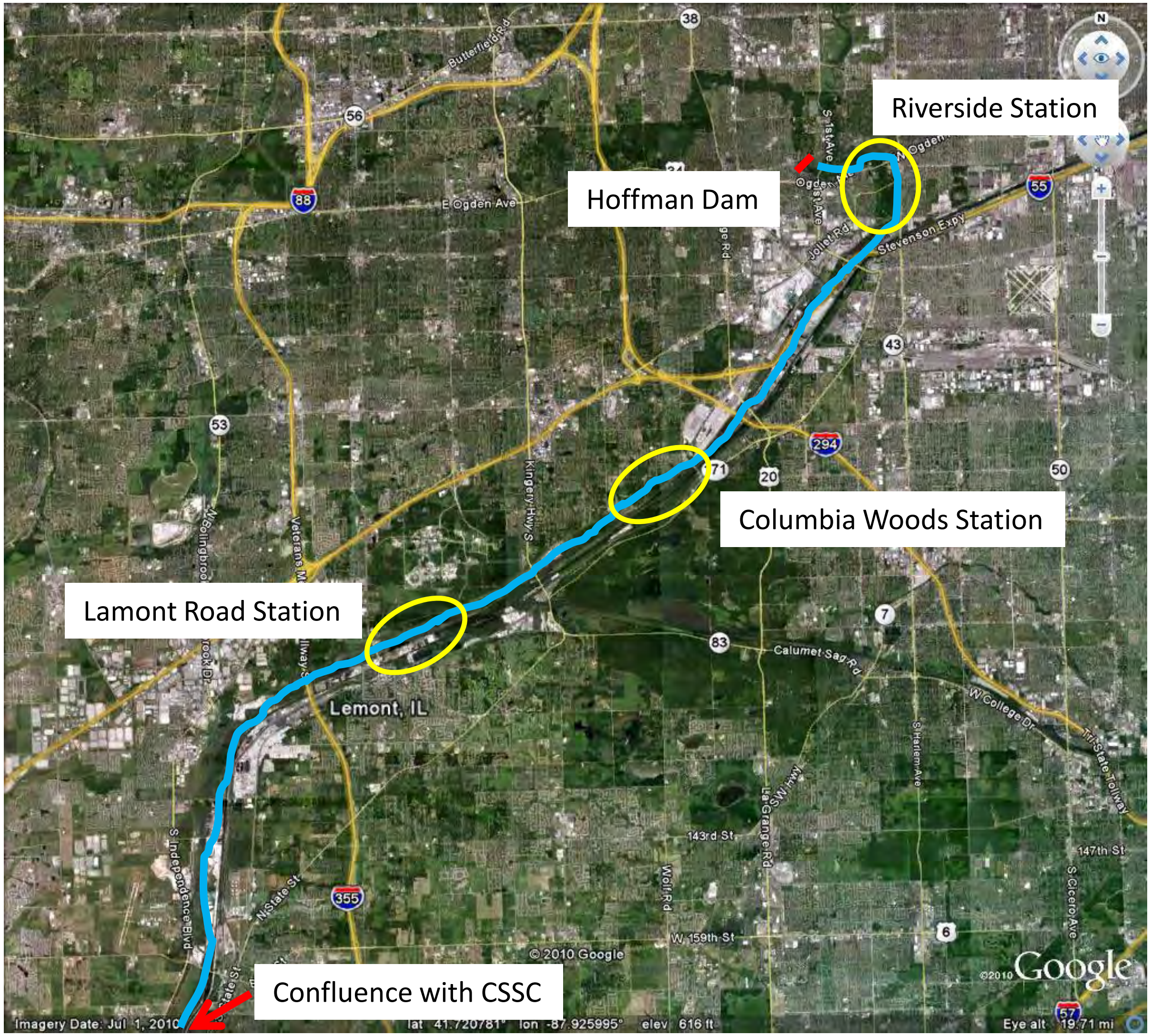

Riverside Station

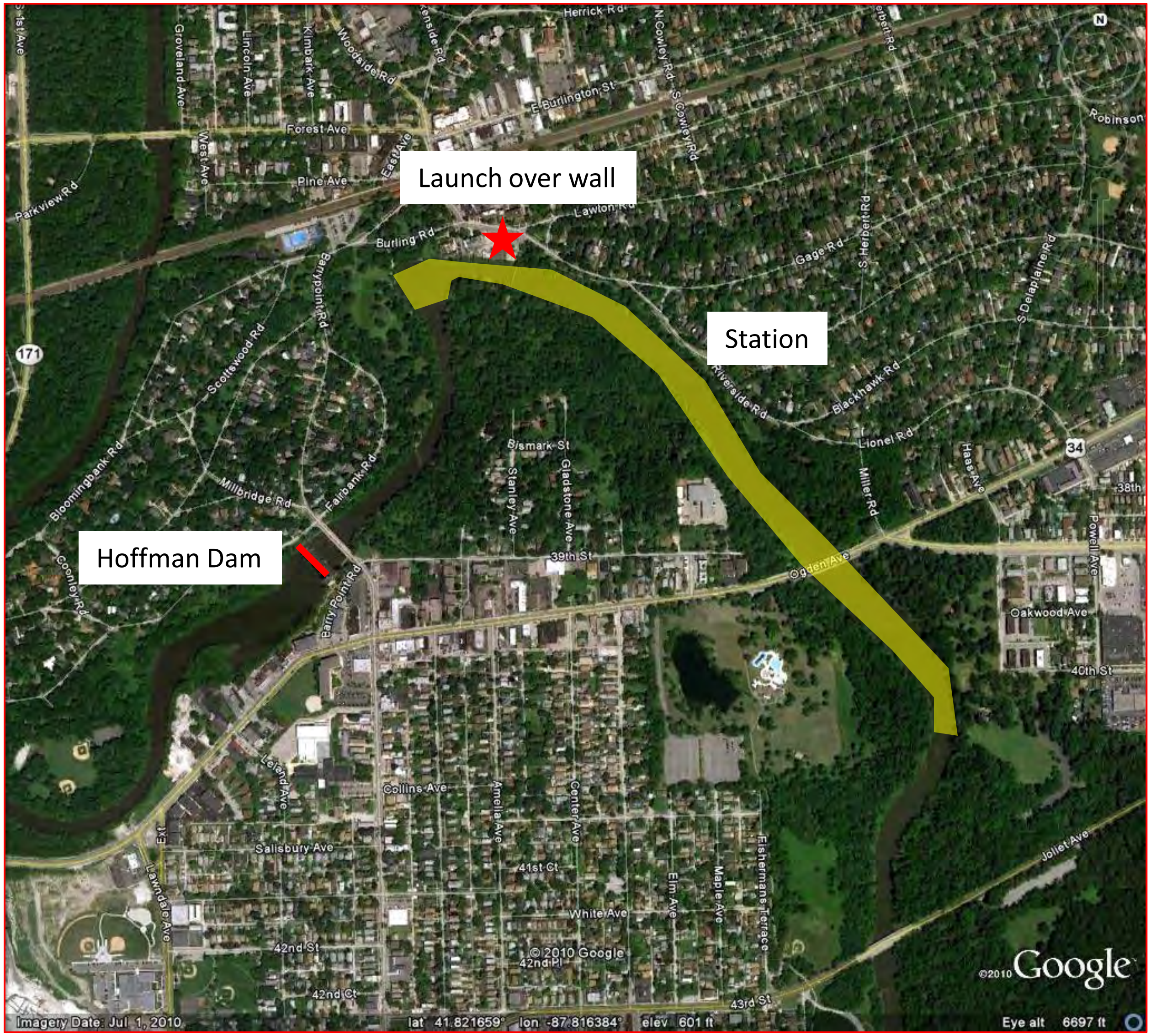

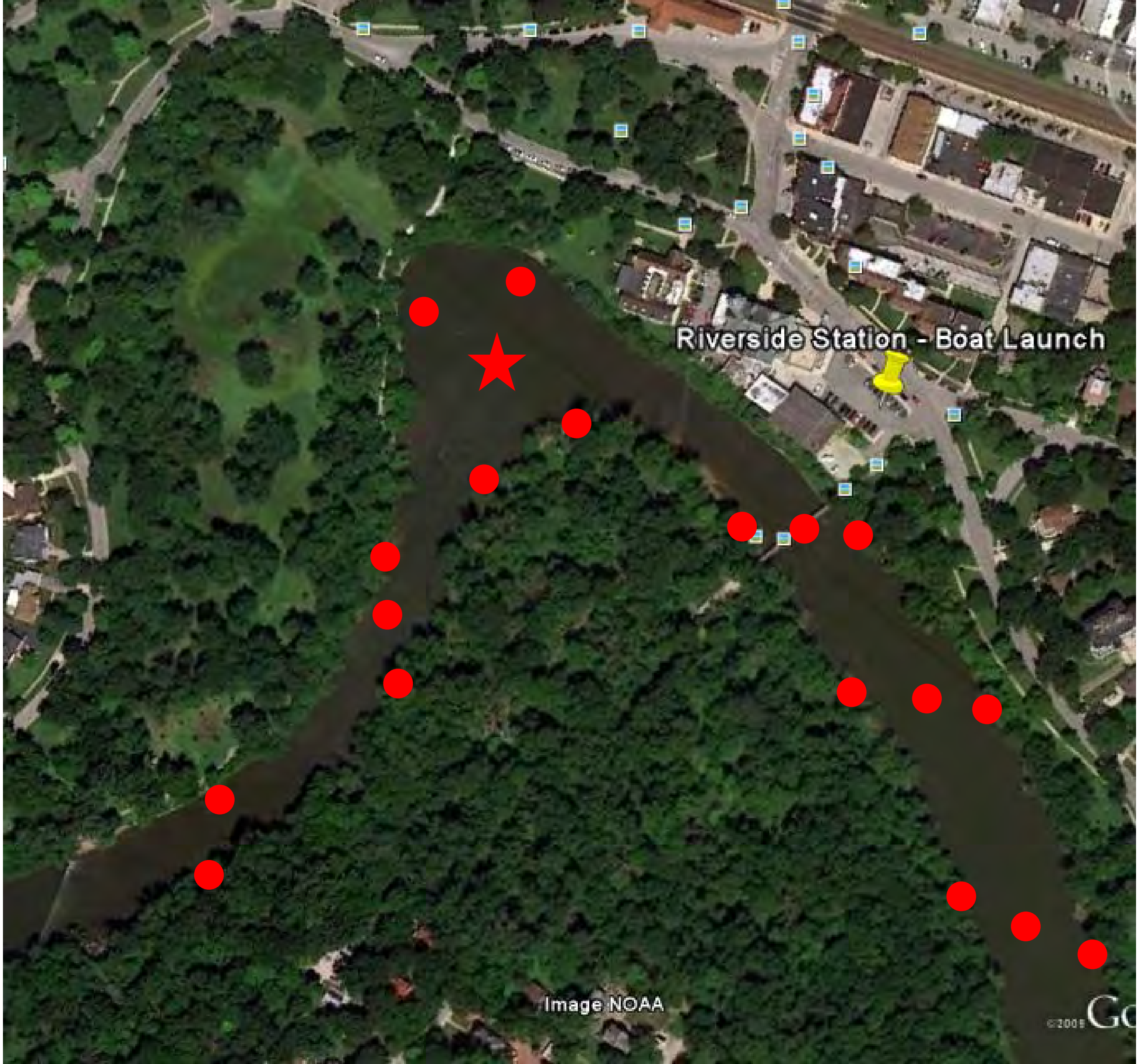
Riverside Station - Boat Launch
Image NOAA
©2008

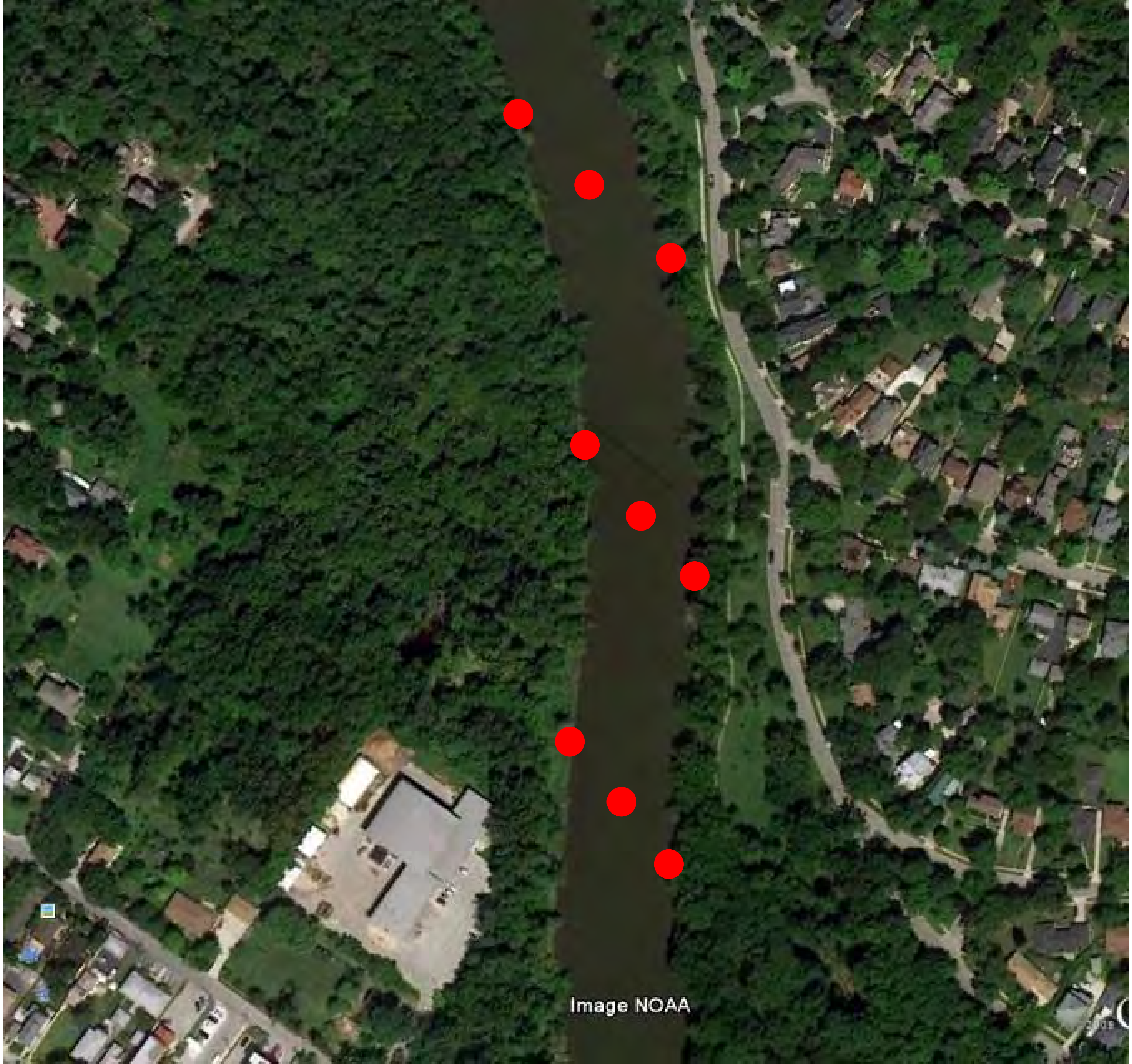
Image NOAA

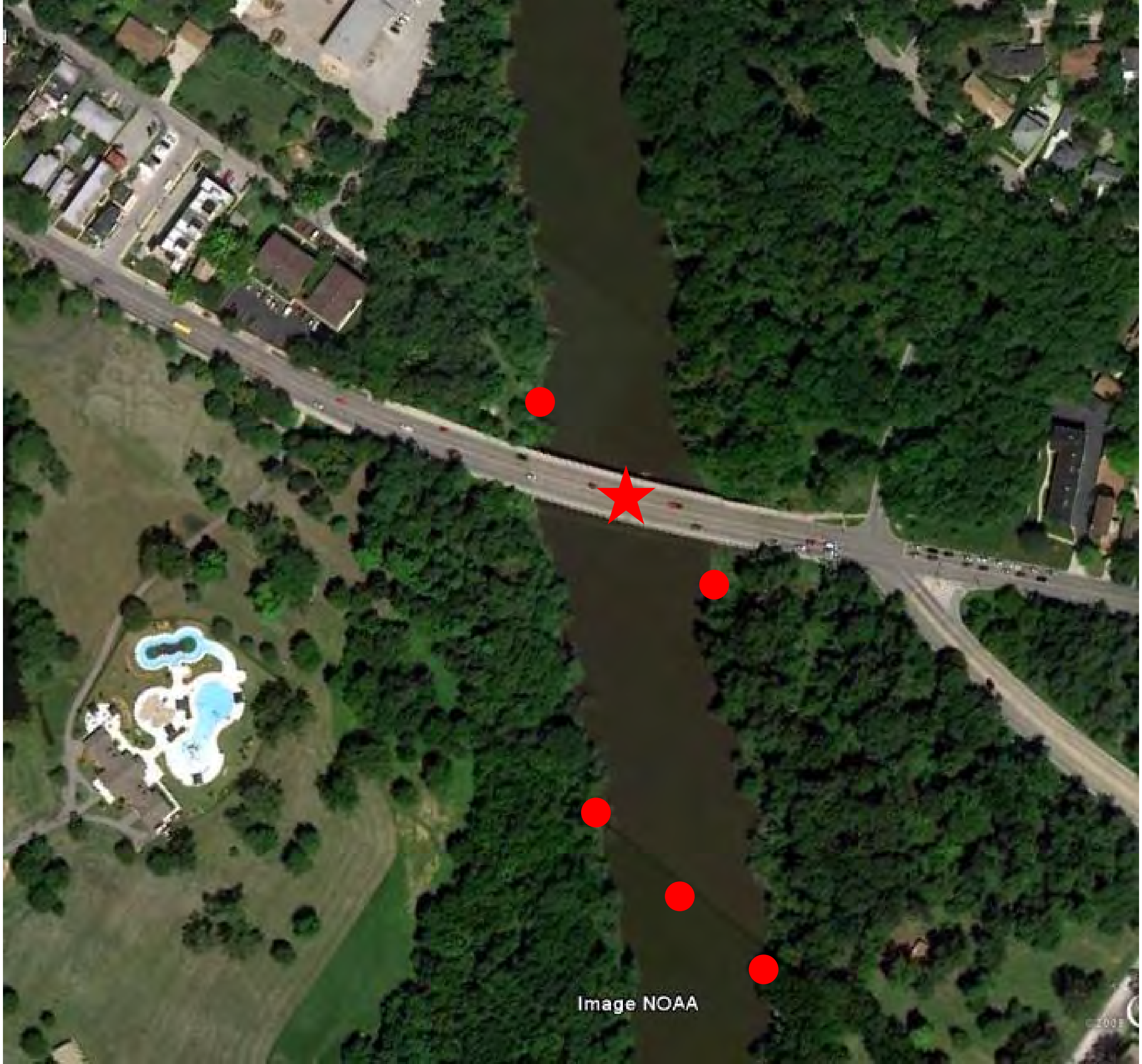
Image NOAA

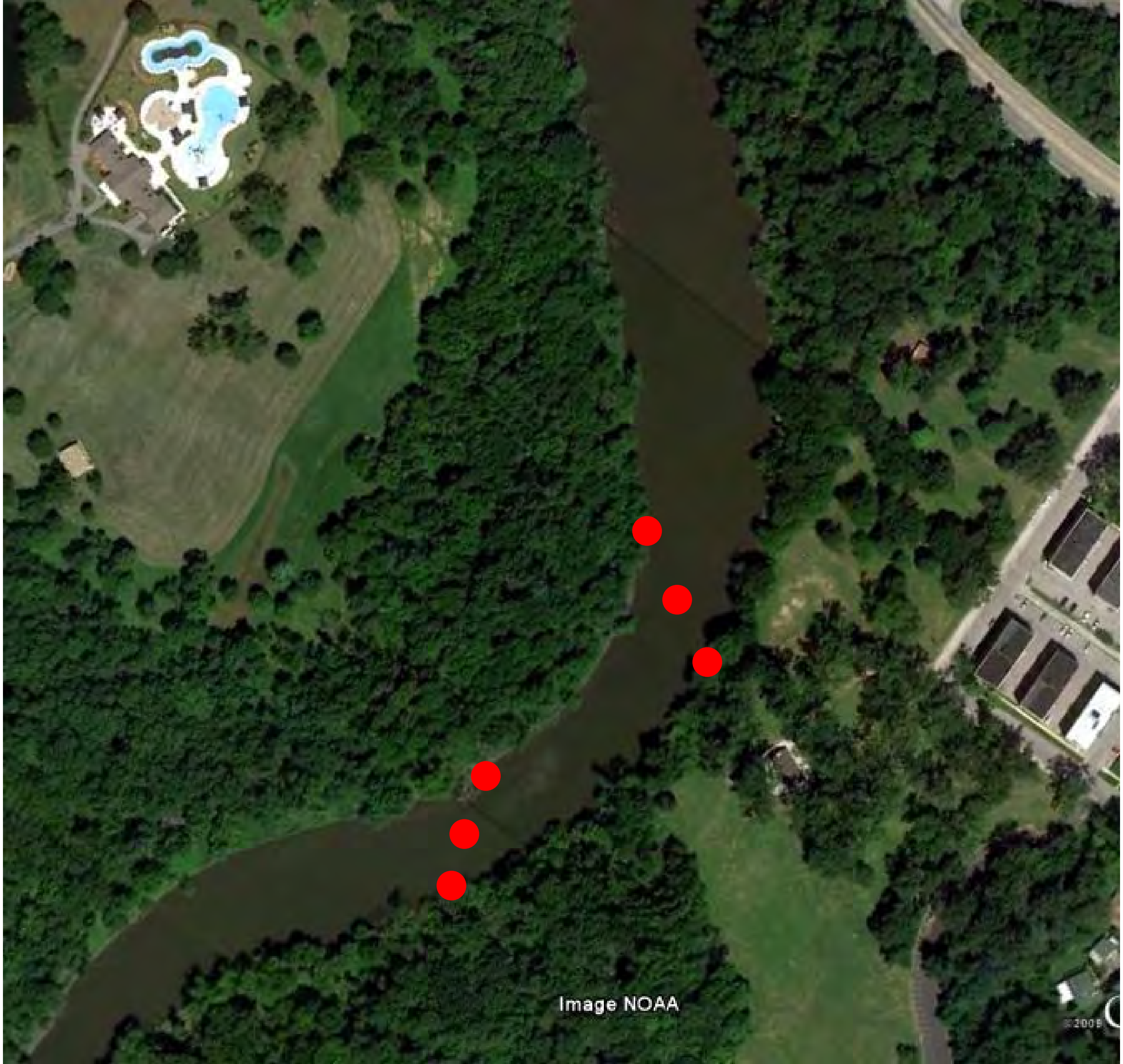
Image NOAA

Columbia Woods Station

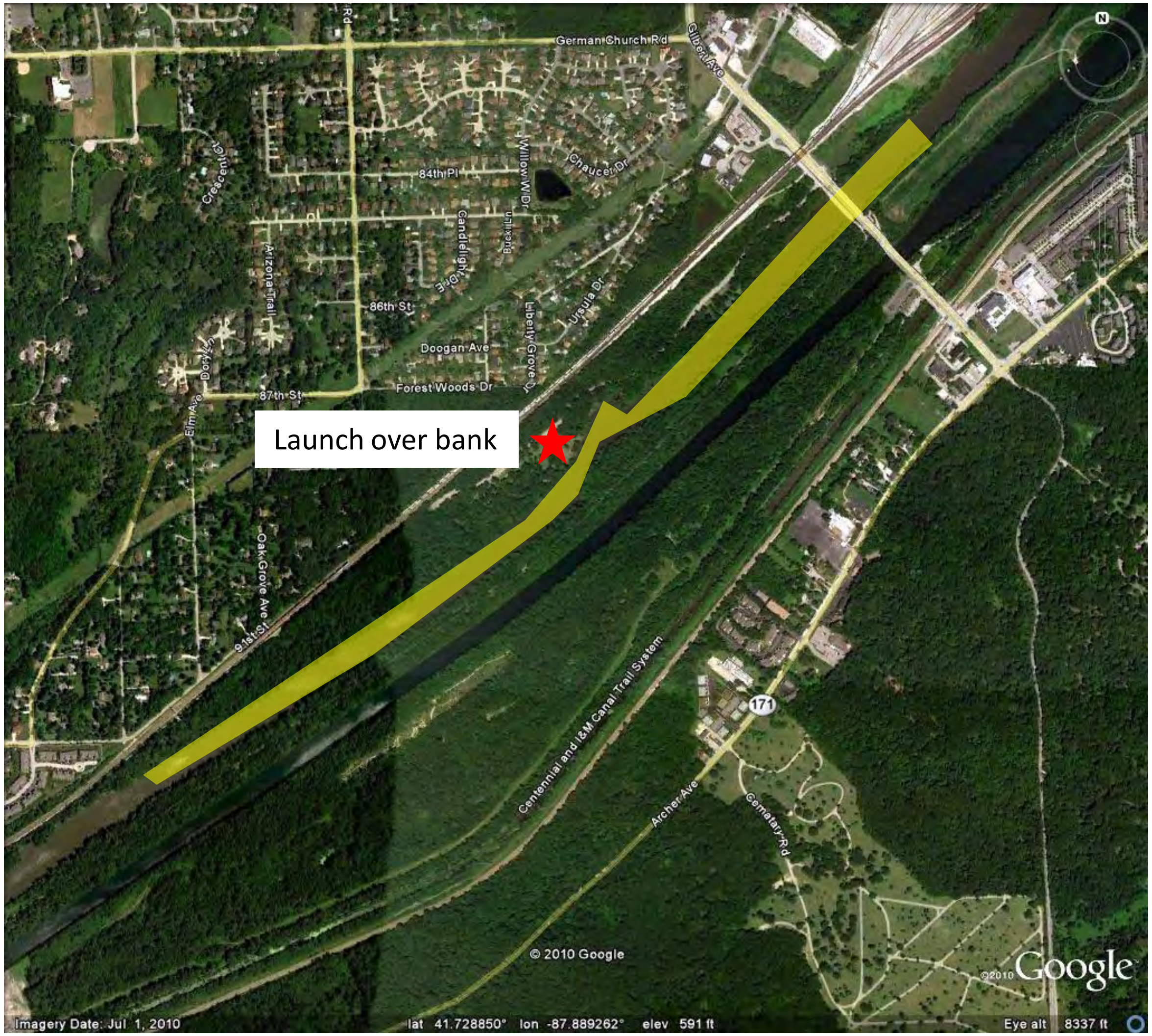

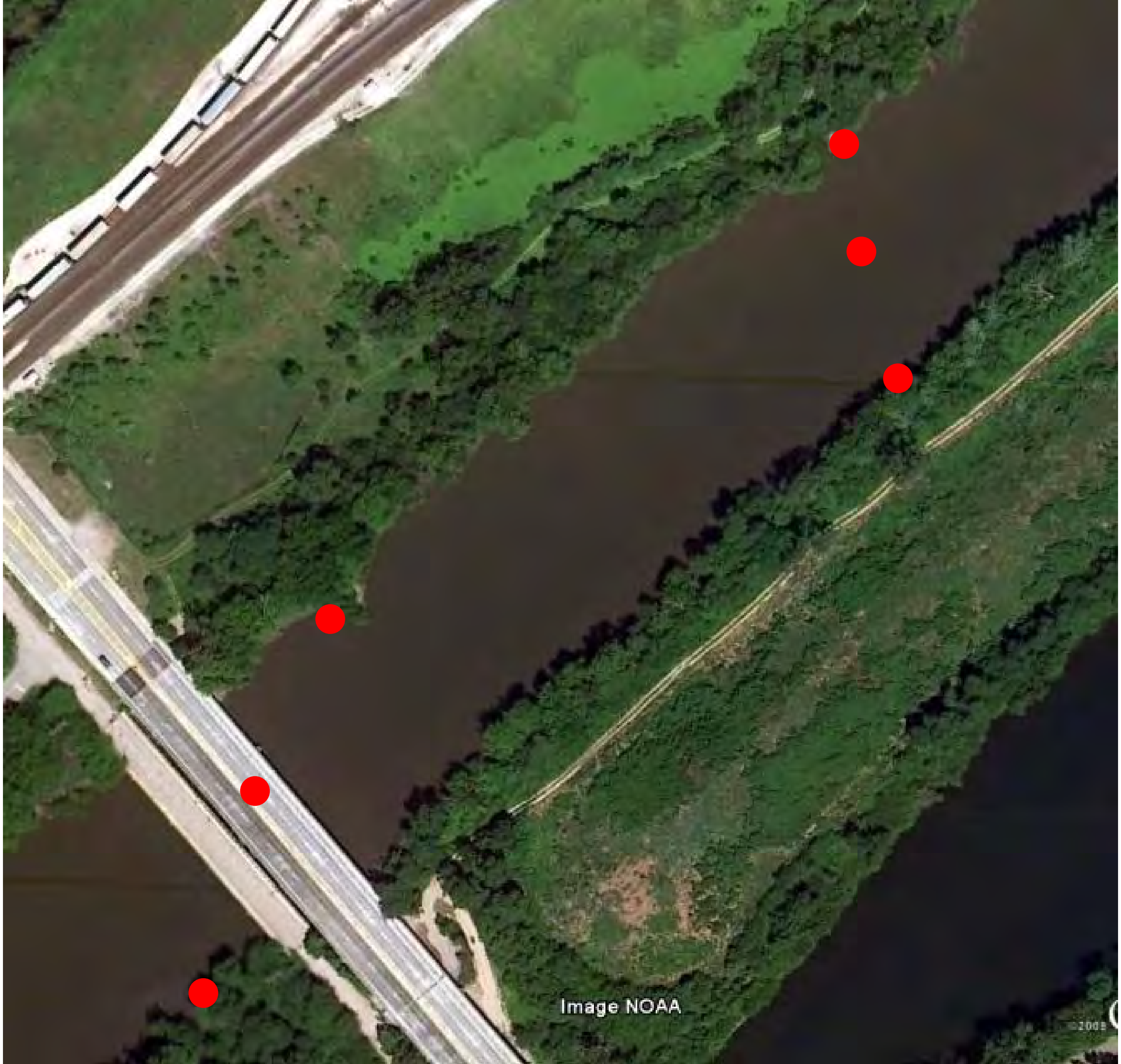
Image NOAA

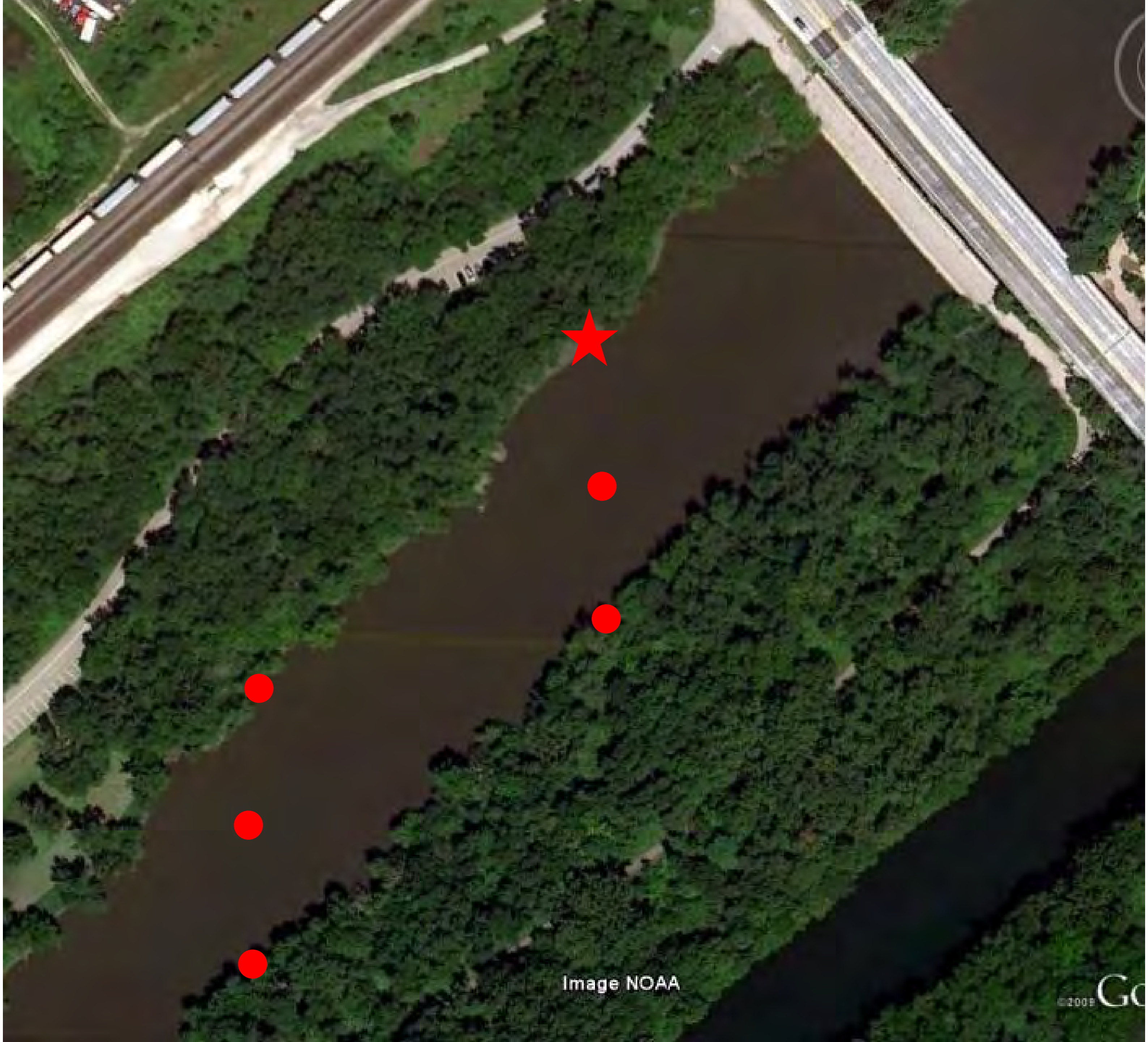
Image NOAA
©2009

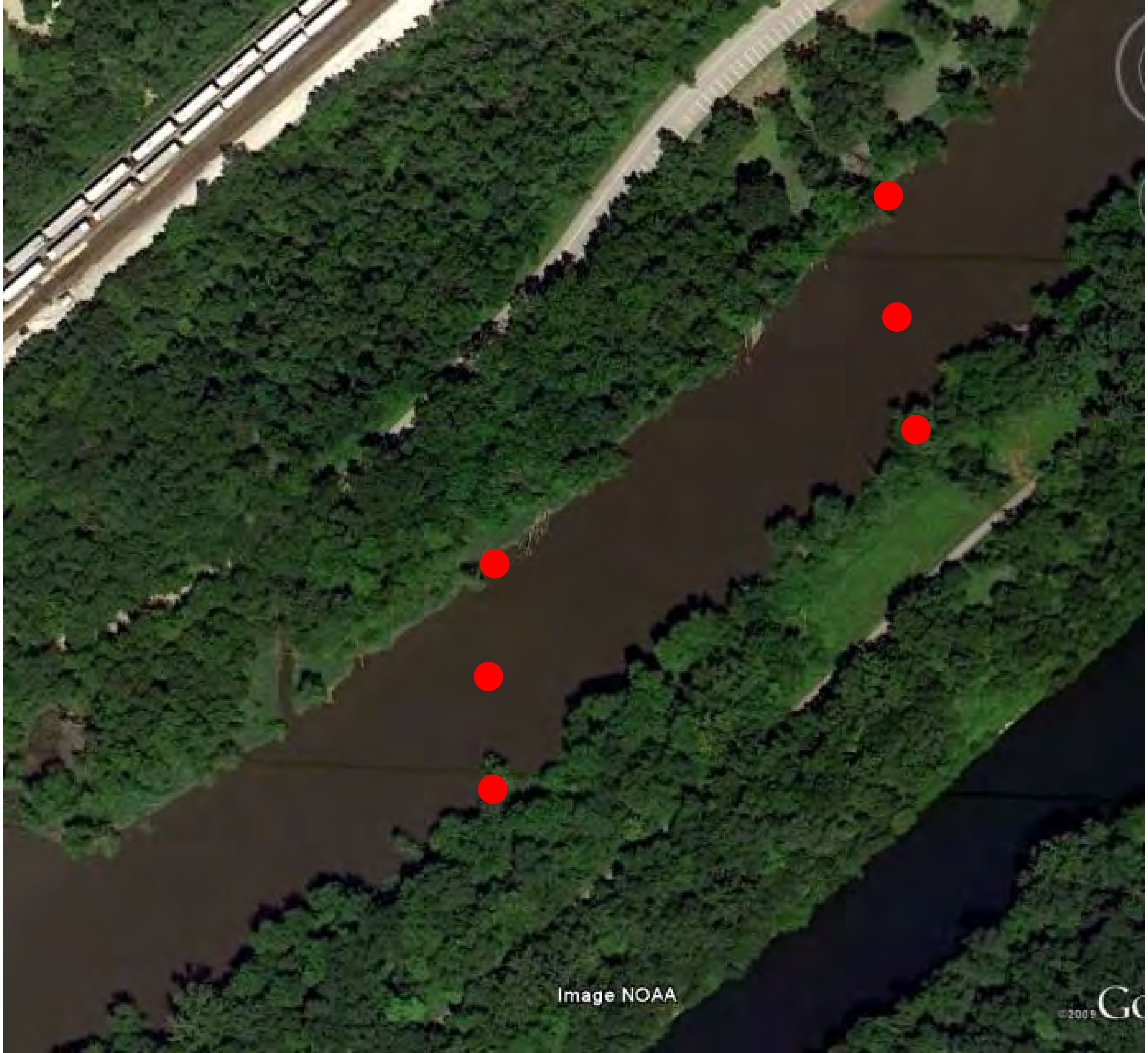
Image NOAA

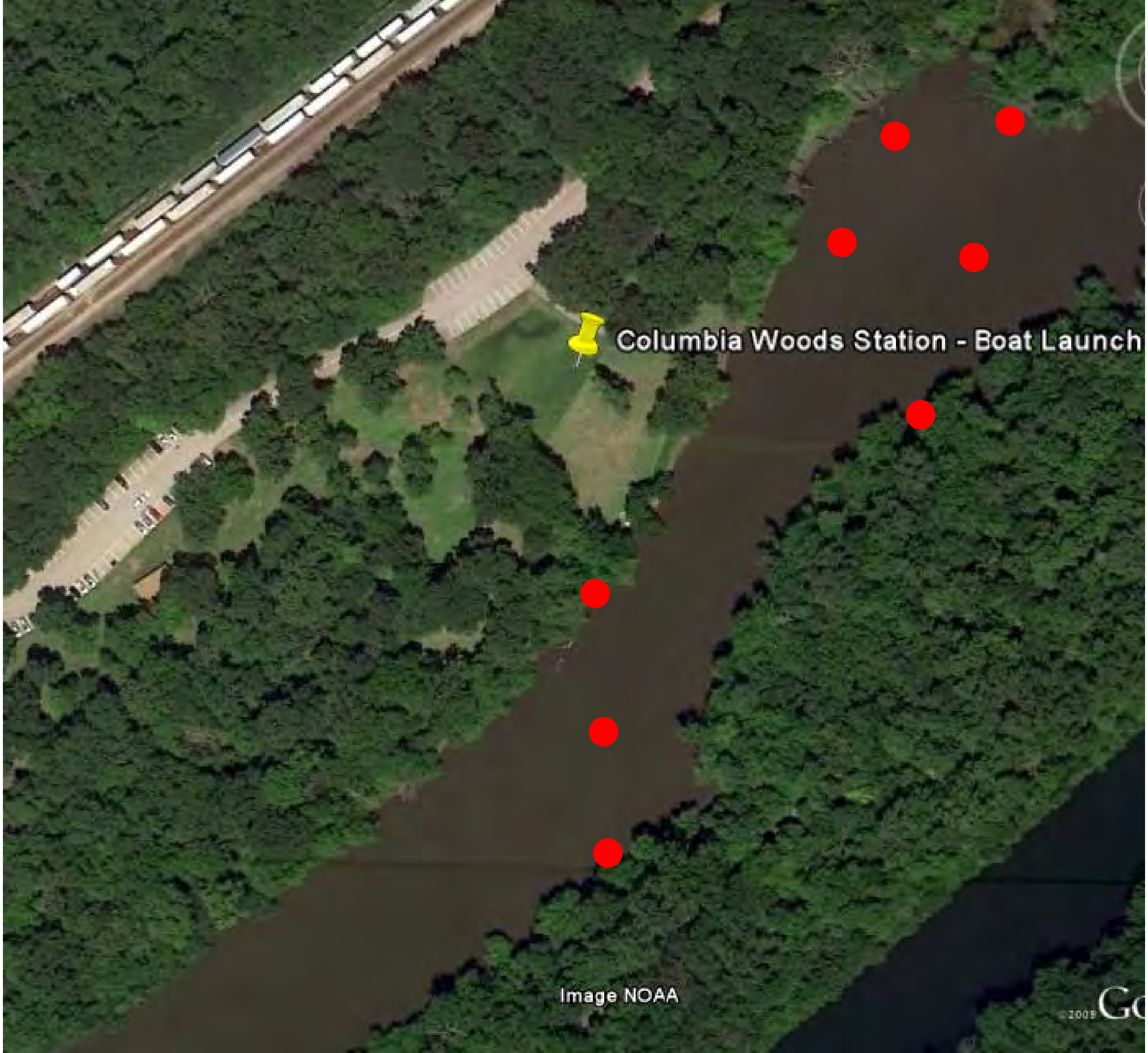
Columbia Woods Station - Boat Launch
Image NOAA
©2009

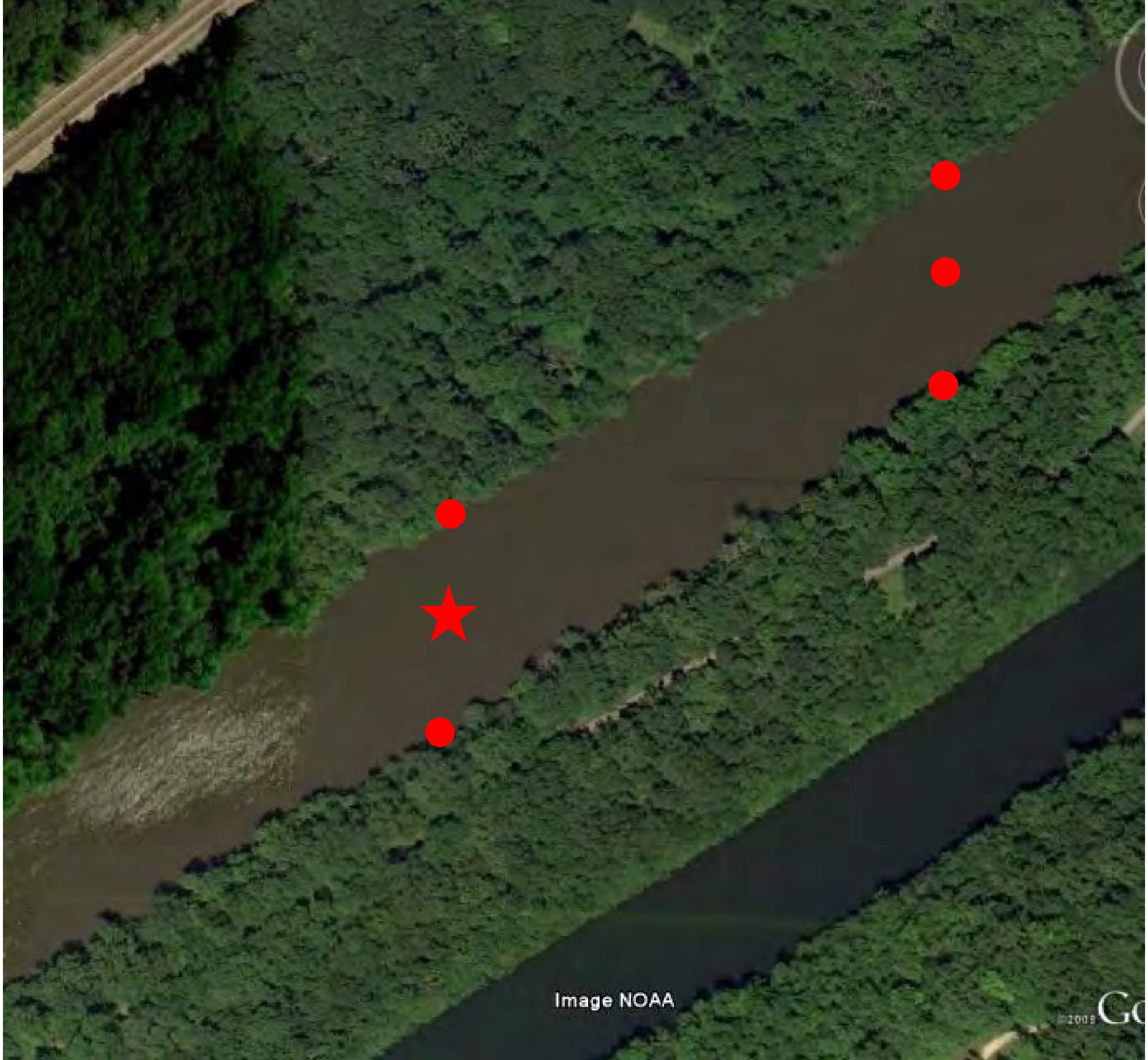
Image NOAA

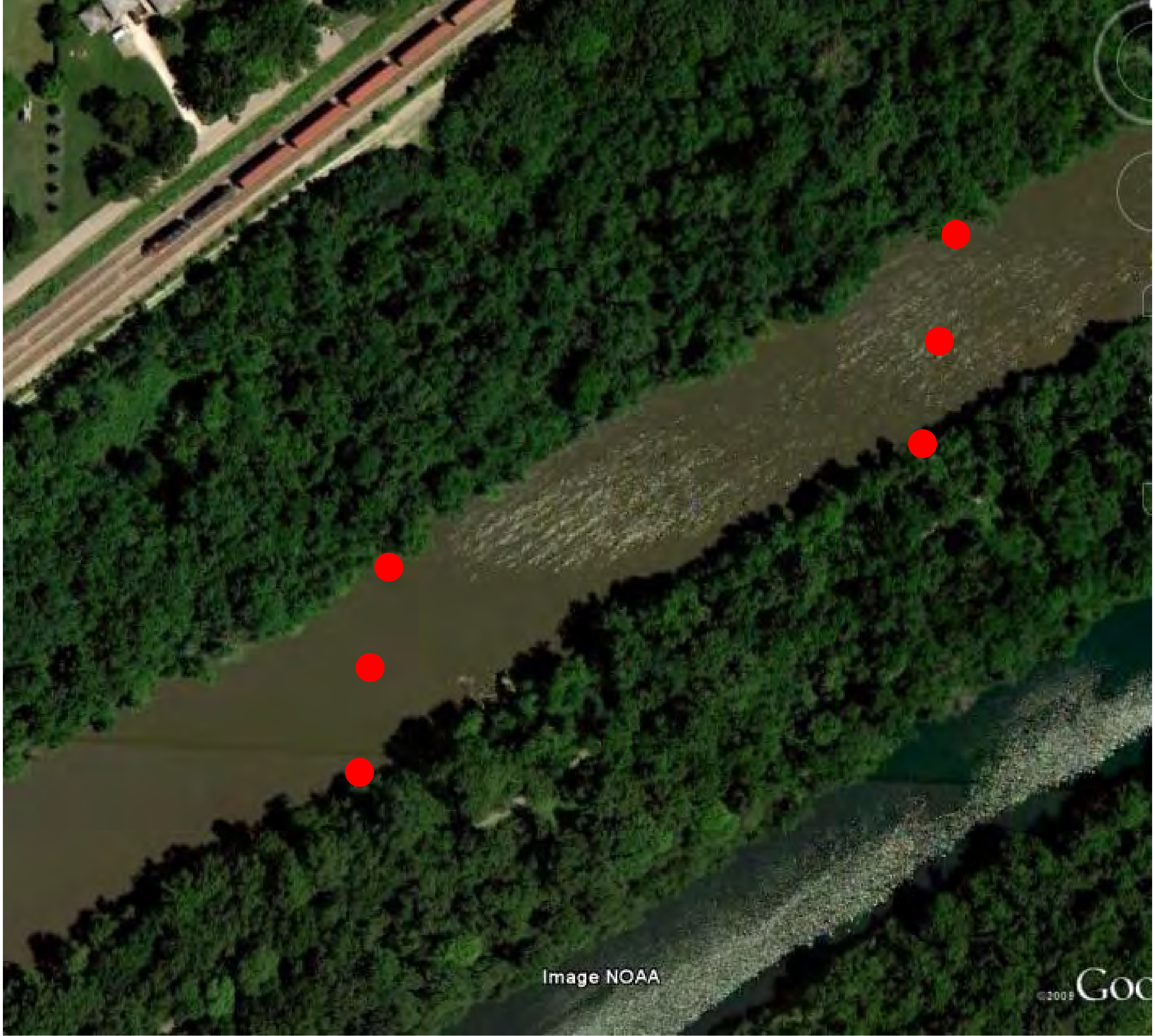
Image NOAA

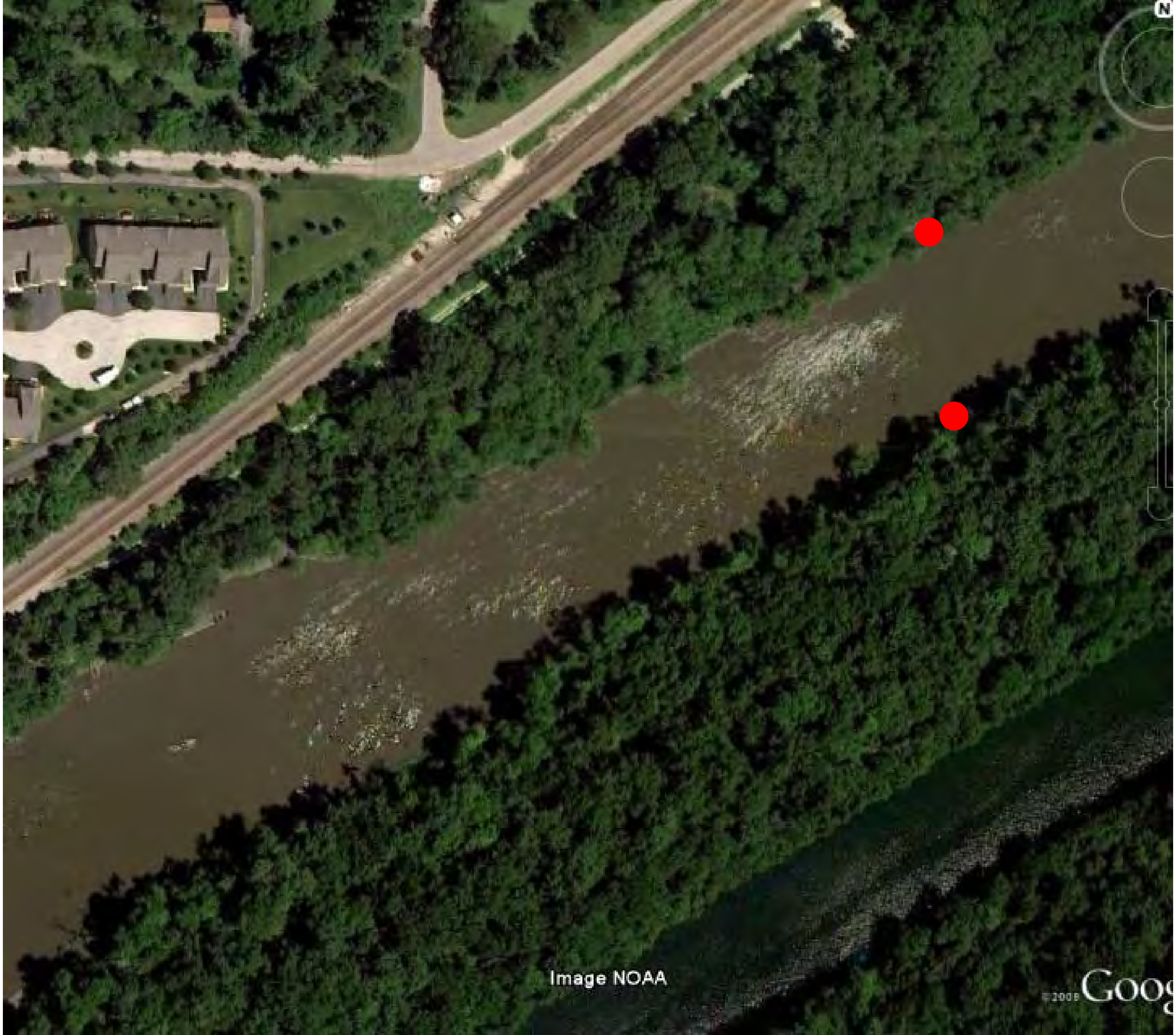
Image NOAA
©2009 Goog

Lamont Road Station

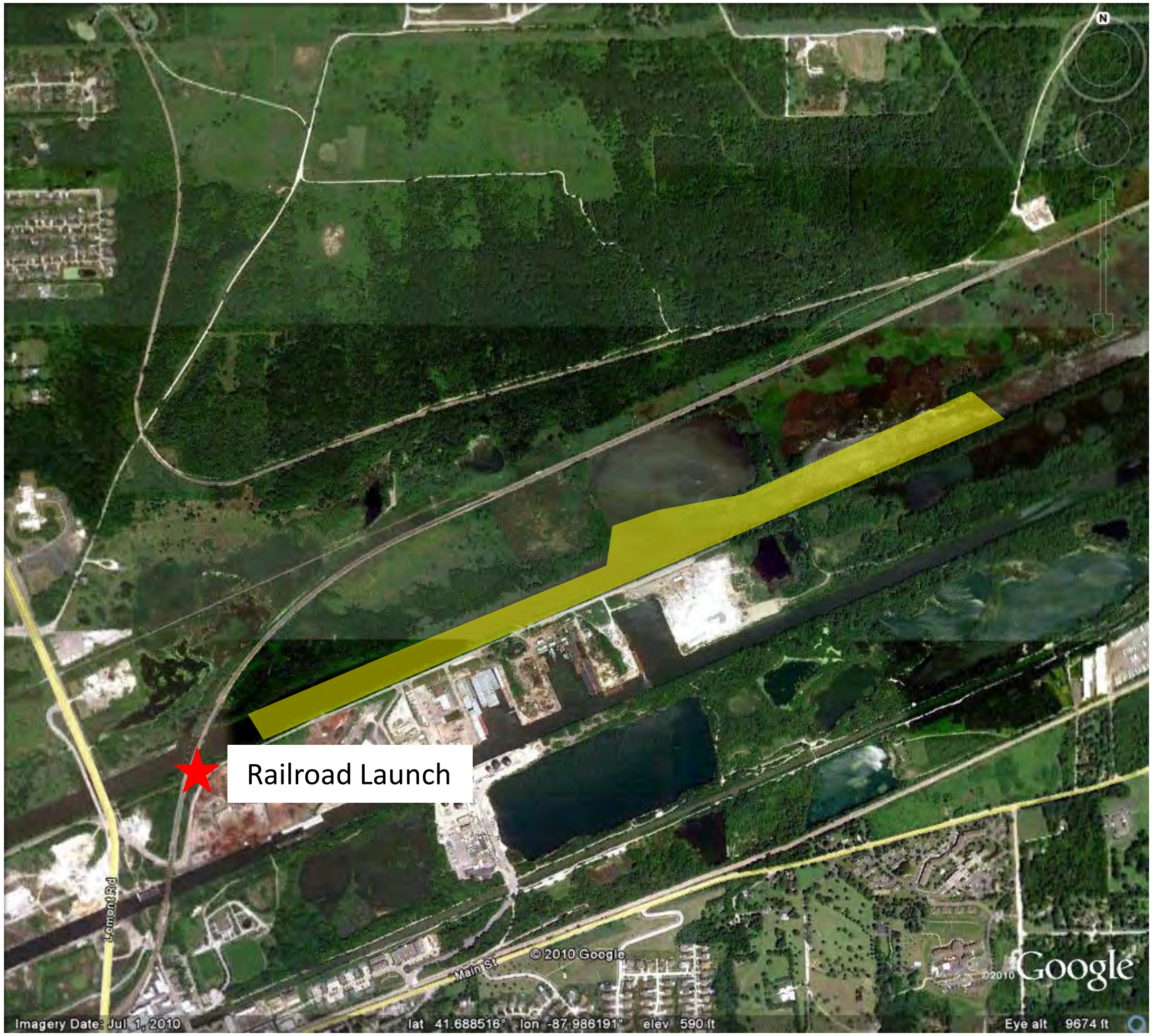

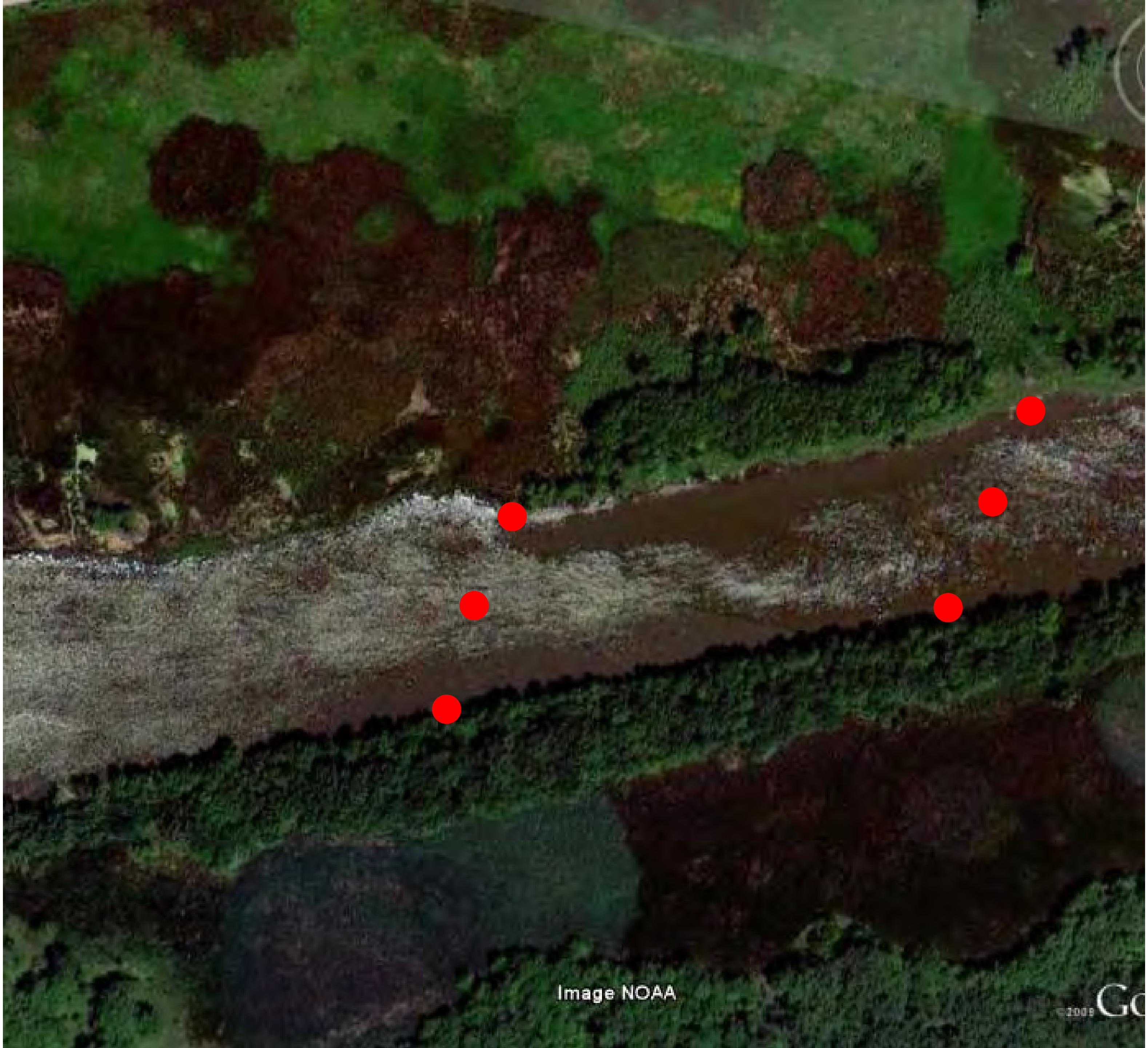
Image NOAA
©2009 Go

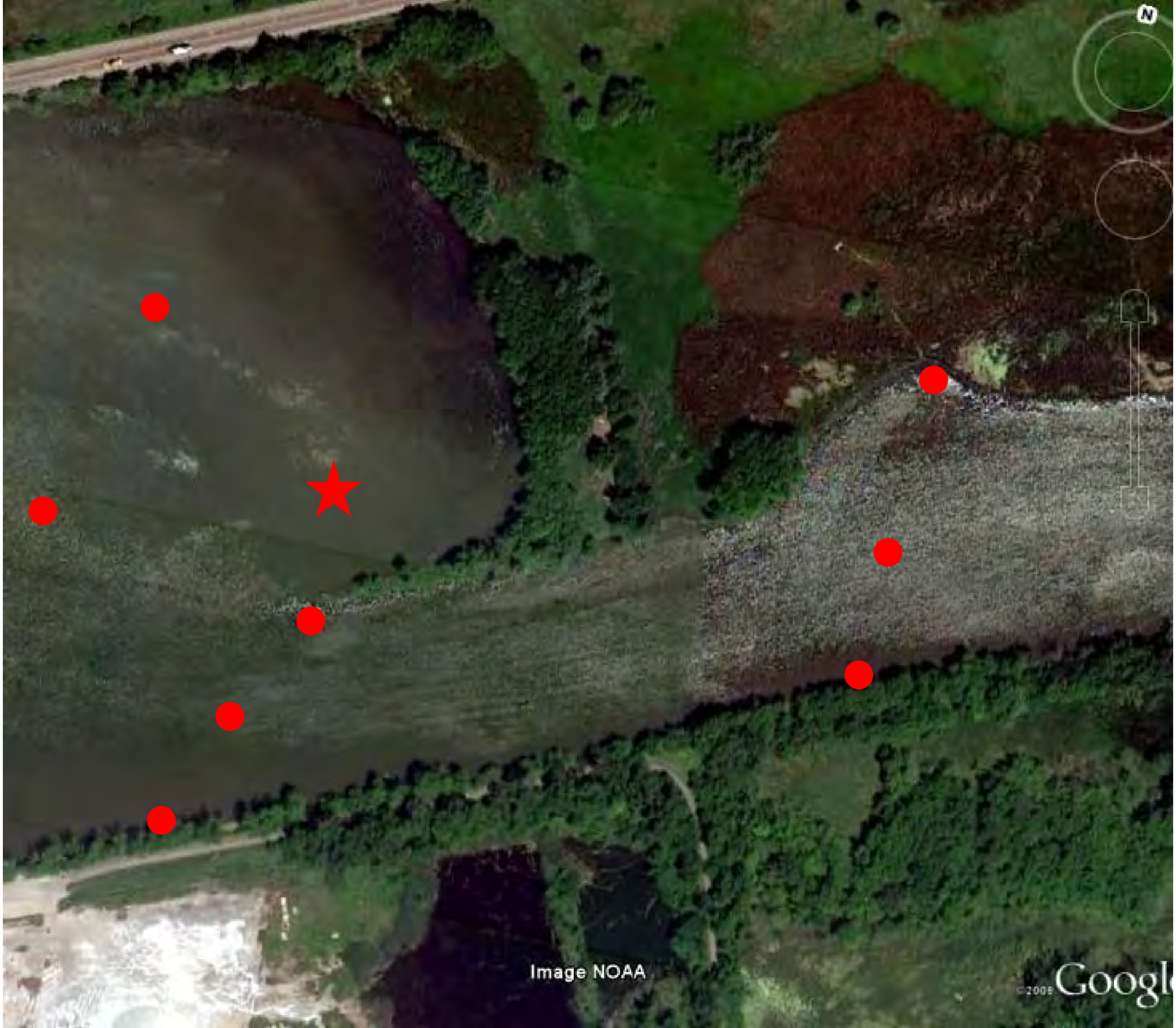
N
Image NOAA
©2009
Google

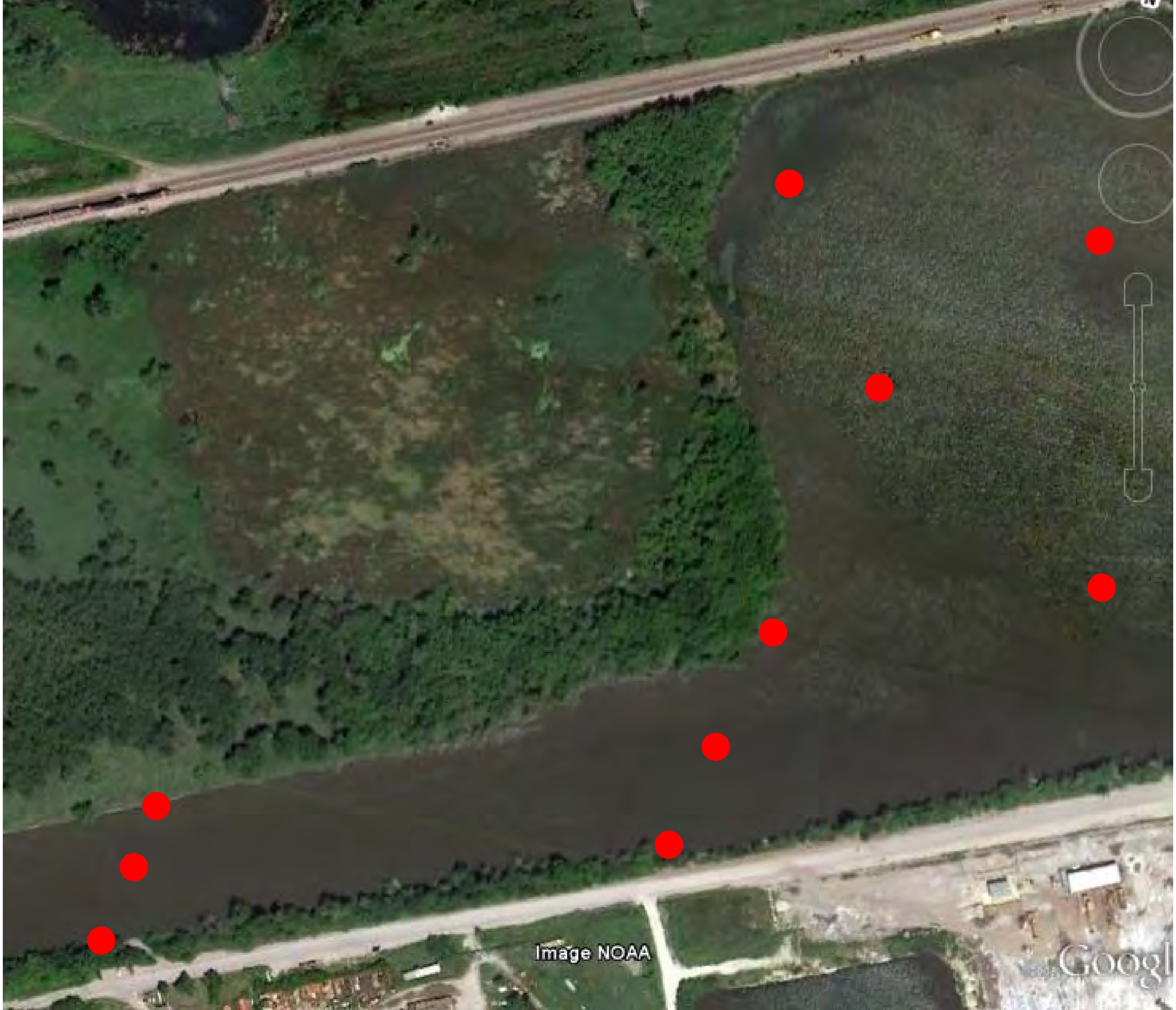
Image NOAA

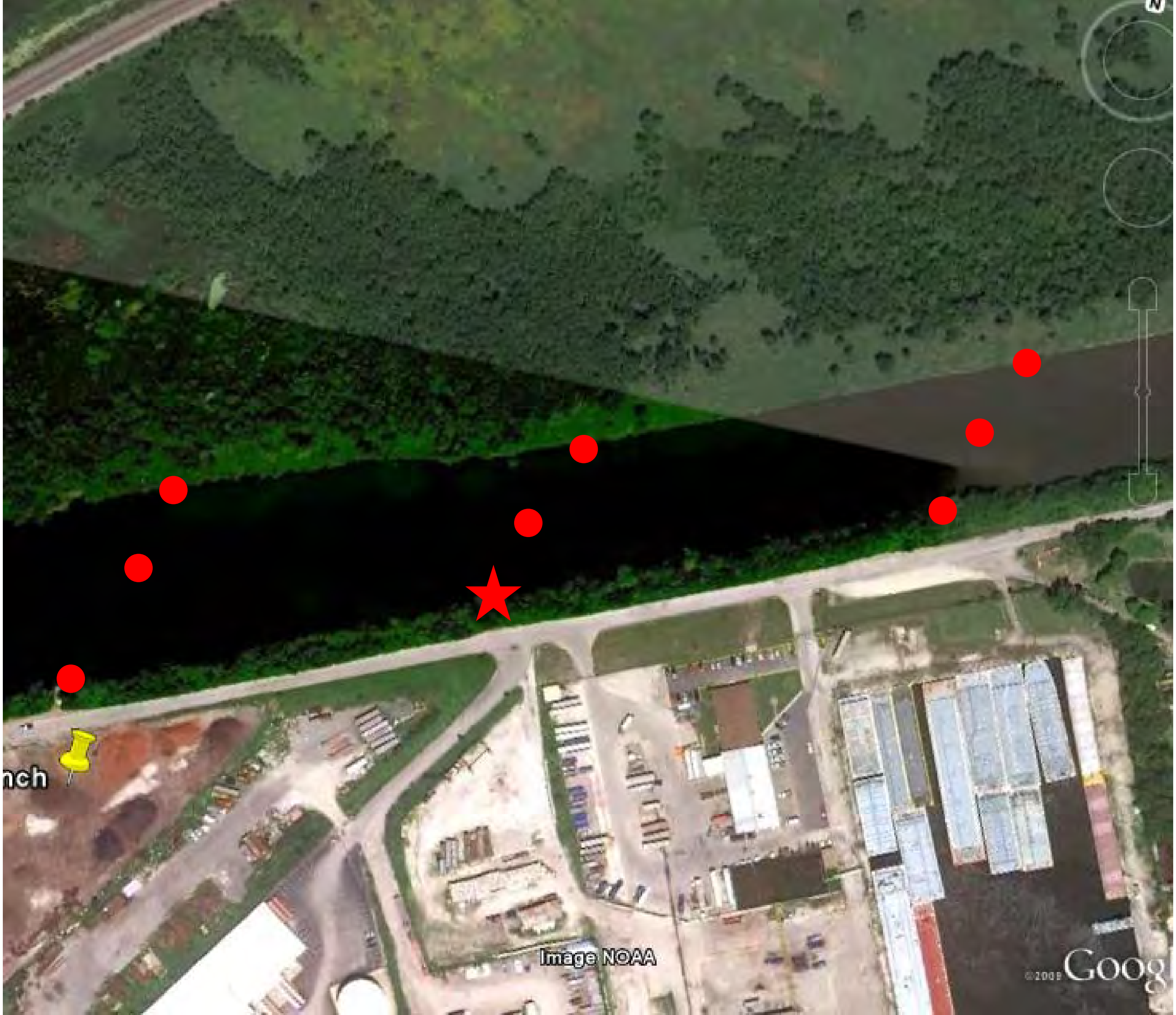
nch
Image NOAA
©2009
Goog

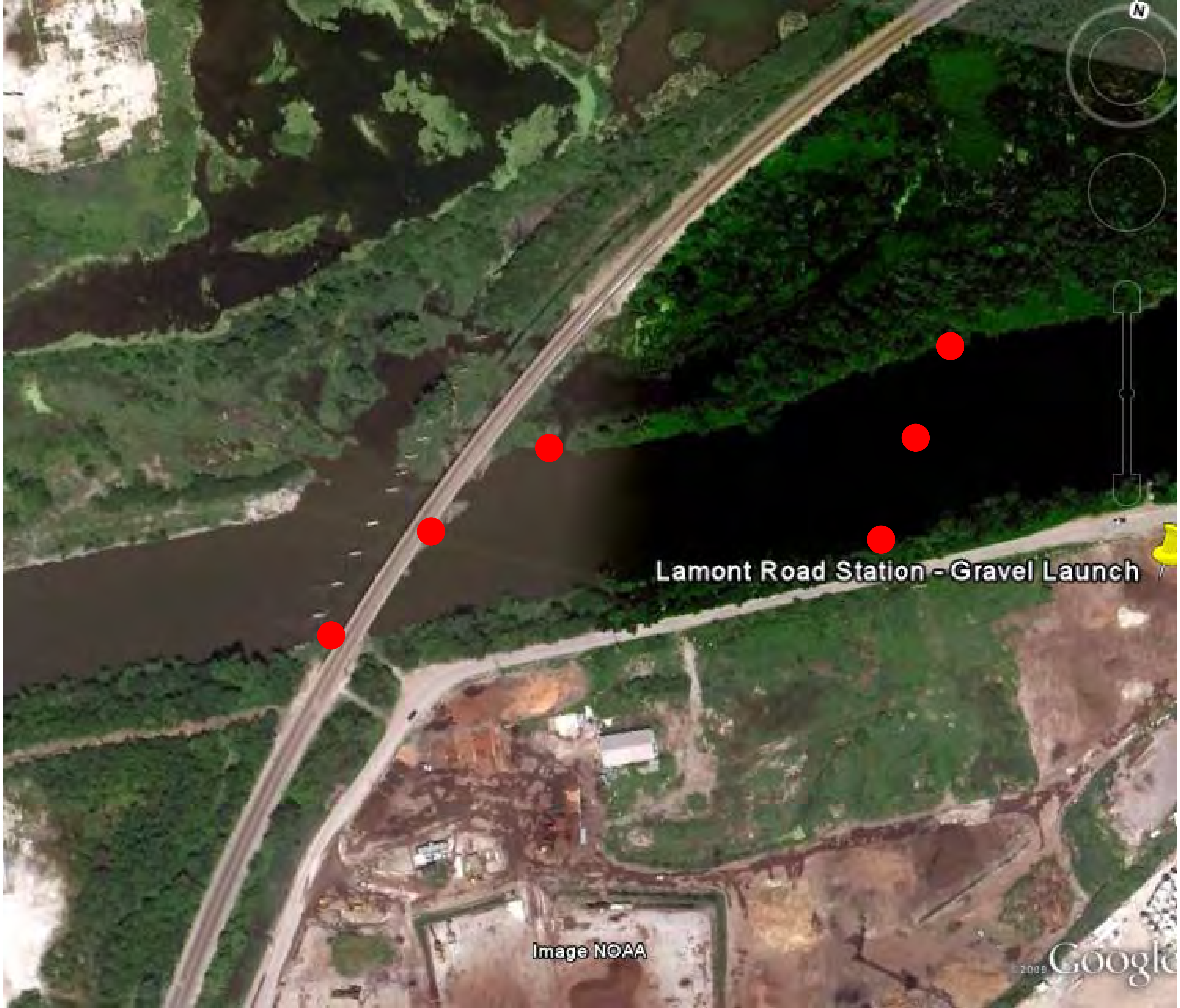
Lamont Road Station - Gravel Launch
Image NOAA
©2008 Google

APPENDIX D

DATA MANAGEMENT

D-1 Purpose

In order to keep accurate records of eDNA sample collections, personnel associated with sampling and filtering of collections, and data associated with a specific collection sample, datasheets associated with sample collections must be kept in accordance with the following protocols for quick reference and to prevent loss. This appendix describes procedures for data reporting and data management specific to USACE.

D-1.1 Data Reporting Procedure

(1) Reporting of unconfirmed positives (presumptive positives):

The eDNA Processing Leader will provide an email to the eDNA Program Manager with preliminary information on unconfirmed positives. Unconfirmed, or presumptive, positives are those samples that have returned a positive hit for silver or bighead carp DNA, yet the equipment or negative controls have not been run, and the sequencing of the DNA has not yet been conducted to confirm the results.

The eDNA Program Manager will check this preliminary information against the database, and develop a preliminary map for USACE internal viewing only. This information serves as a warning for upcoming confirmed results. The eDNA Program Manager will then provide this information to the Project Manager and USACE leadership only.

The email should contain the following information:

- Sample batch (date and collection reach)
- Sample ID with unconfirmed positive and for which species

(2) Reporting of confirmed results:

The eDNA Processing Leader will provide an email to the eDNA Program Manager with information regarding confirmed positive data as soon as it is available. The email should contain the following information:

- Sample batch (date and collection reach)
- Sample ID with confirmed positive and for which species

The eDNA Program Manager will prepare an official map within a geodatabase; the eDNA Sampling Specialist will update the table in the Excel database and send this table to the eDNA Program Manager. The eDNA Program Manager will then compile both sets of information (map and table) into one file and distribute to USACE, ACRCC co-chairs, and the MRRWG co-chairs and web posting.

(3) Monthly reports:

Monthly reports will be produced by the eDNA Processing Leader, the eDNA Program Manager, and the eDNA Sampling Specialist for synthesis of collected data and record management.

The format of the report should include:

(a) Summary of results from reporting month and context of results to previous sampling events
(b) Maps and tables generated during the reporting period

(c) News (staff or equipment notes, incident reports, etc)
(d) Results for the previous month in a tabular format:
Date/Time collected, Location (Reach, Lat-Long), Sample ID, Sampling Crew, Volume of sample, Temp, Depth, Filter Time, Filter Crew, Results (Positive or Negative) for each species (bighead or silver carp).
(e) Forthcoming sampling efforts (location, dates, and crews)

The following is a breakdown of responsibilities for each contributor:

(a) eDNA Processing Leader is responsible for ensuring accuracy of results reported. Also responsible for providing news (item b, above).
(b) eDNA Program Manager and eDNA Sampling Specialist will collaborate to complete the rest of the report. Report will be provided to the Project Leader for distribution and/or web posting.
(c) Reports will be completed at the end of each month, unless data comes in at the end of the month, which should be included in that month's report. Reports will be completed NLT 5 days after last results are received for the month.

D-1.2 Data Management Procedure

(1) Field datasheets (Exhibit Forms 1 – 3) will be filled out completely in accordance with protocols described in Sections 2.0 and 3.0.

(2) Completed field datasheets will be handed over to the Filtering Leader upon arrival to the lab. Once the samples and datasheets have been transferred from the sampling crew (i.e. FWS, IDNR, and/or EPA) to the filtering crew (i.e. USACE), the COC will be signed (Exhibit Form 3).

(3) Datasheets for a single sampling event will then be scanned in and saved as a pdf in the following format: mm_dd_yyyy_Location.pdf. For example, on March 23, 2010, 120 samples were collected from Lockport Pool. The datasheets for this single sampling event would be saved as the following pdf: 03_23_2010_Lockport Pool.pdf.

(4) Exhibit Form 3 is the COC and is mailed along with the sample collection to ERDC. Once ERDC has received the samples and has found no evidence of tampering, the COC should be signed, scanned, and emailed to the LRC point of contact (Shawna.S.Herleth-king@usace.army.mil). This document can then be attached to the appropriate sample collection pdf.

(5) The datasheet pdf containing Exhibit Forms 1-3 will then be saved via the following pathway:

<P:\PRJ-3061a Dispersal Barriers\Carp Monitoring\eDNA Monitoring\USACE eDNA Data>

(6) Hard copies of field datasheets will be maintained in the USACE – Chicago District's Planning Library. Datasheets will be filed in cabinet #2, drawer 1, Dispersal Barriers file marker, eDNA Data yyyy file folder. Datasheets will be separated by year and ordered sequentially by date within each eDNA Data year file folder.

(7) Electronic database management – Datasheets are sent in Microsoft Excel format to MVR PM GIS personnel, who proceed to save a copy of the excel sheets to:

\\mvr-netapp1\egis\Data\zz_WORK\Rivers\Illinois_River\Asian_Carp\data

Once the sheets have been copied, they are modified slightly to facilitate importing into an ESRI File Geodatabase table. The sheets are then imported into an ESRI file geodatabase table named Samples_Table located at:

\\mvr-netapp1\egis\Data\zz_WORK\Rivers\Illinois_River\Asian_Carp\data\Asian_Carp_RCC_MRRW.gdb

Once the table has been imported, it is converted to a file geodatabase feature class named Samples (replacing any older version of the feature class) and is then ready for display on map products.
