# Supplementary material for: Modeling the Sensitivity of Field Surveys for Detection of Environmental DNA (eDNA)
Source: PLoS One. 2015 Oct 28;10(10):e0141503. doi: 10.1371/journal.pone.0141503 (PMC4624909; doi:10.1371/journal.pone.0141503)
Supplement: S1 Table — (PDF) [file pone.0141503.s002.pdf]

Table S1. Fraction of PCR replicates causing fluorescence on an agarose gel.

Expected number of target marker copies	Number of PCR replicates	Fraction of PCR replicates testing positive	
		Bighead carp	Silver carp
1	60	0.217	0.283
2	30	0.700	0.300
3	30	0.800	0.300
4	30	0.800	0.567
5	60	0.817	0.850
6	30	0.933	0.633
7	30	0.967	-†
8	30	0.967	0.900
9	30	1.000	0.933
10	60	1.000	0.983
11	30	1.000	1.000
12	30	1.000	0.967
13	30	1.000	0.967
14	30	1.000	1.000
15	30	1.000	1.000
50	30	1.000	1.000
100	30	1.000	1.000
200	30	1.000	1.000
500	30	1.000	1.000
1000	30	1.000	1.000

(†) Not evaluated.
