# Supplementary material for: Modeling the Sensitivity of Field Surveys for Detection of Environmental DNA (eDNA)
Source: PLoS One. 2015 Oct 28;10(10):e0141503. doi: 10.1371/journal.pone.0141503 (PMC4624909; doi:10.1371/journal.pone.0141503)
Supplement: S2 Table — (PDF) [file pone.0141503.s003.pdf]

Table S2. Fraction of PCR replicates successfully sequenced for target markers.

Expected number of target marker copies	Target species					
	Bighead carp			Silver carp		
	Number of runs	Successful runs	Success ratio	Number of runs	Successful runs	Success ratio
1	6	0	0.000	6	6	1.000
2	21	2	0.095	9	8	0.889
3	24	0	0.000	9	9	1.000
4	25	6	0.240	17	16	0.941
5	28	4	0.143	21	20	0.952
6	29	6	0.207	19	18	0.947
7	29	13	0.448	5	5	1.000
8	30	4	0.133	27	24	0.889
9	-†	-†	-†	28	15	0.536
10	-†	-†	-†	29	29	1.000
11	-†	-†	-†	30	27	0.900
12	-†	-†	-†	29	28	0.966
13	-†	-†	-†	29	28	0.966
14	-†	-†	-†	30	30	1.000
15	30	25	0.833	30	30	1.000
25	29	13	0.448	-†	-†	-†
50	30	21	0.700	-†	-†	-†
100	29	27	0.931	-†	-†	-†

(†) Not evaluated.
